# Meta-Analysis and Gene Set Analysis of Archived Microarrays Suggest Implication of the Spliceosome in Metastatic and Hypoxic Phenotypes

**DOI:** 10.1371/journal.pone.0086699

**Published:** 2014-01-31

**Authors:** Bertrand De Meulder, Fabrice Berger, Eric Bareke, Sophie Depiereux, Carine Michiels, Eric Depiereux

**Affiliations:** 1 Microorganism Biology Research Unit -NARILIS, University of Namur, Namur, Belgium; 2 Sainte Justine University Hospital Center Research Center, University of Montreal, Montreal, Canada; 3 Environmental and Evolutional Research Unit, University of Namur, Namur, Belgium; 4 Cellular Biology Research Unit - NARILIS, University of Namur, Namur, Belgium; Harvard School of Public Health, United States of America

## Abstract

We propose to make use of the wealth of underused DNA chip data available in public repositories to study the molecular mechanisms behind the adaptation of cancer cells to hypoxic conditions leading to the metastatic phenotype. We have developed new bioinformatics tools and adapted others to identify with maximum sensitivity those genes which are expressed differentially across several experiments. The comparison of two analytical approaches, based on either Over Representation Analysis or Functional Class Scoring, by a meta-analysis-based approach, led to the retrieval of known information about the biological situation – thus validating the model – but also more importantly to the discovery of the previously unknown implication of the spliceosome, the cellular machinery responsible for mRNA splicing, in the development of metastasis.

## Introduction

### Cancer & metastasis

Despite the development of effective therapies for many cancers [1]-[3], the prevalence of cancer is growing alarmingly in aging populations [4]. Metastases are one of the main causes of death related to cancer [5]. It is therefore not surprising that a large number of labs and researchers focus on gaining a better understanding of the metastatic process [6]–[8].

Cancer is known to be a genetic disease, implying either alteration of DNA or dysregulation of gene expression [9]. In addition, the metastatic phenotype involves the combination of several factors [7], among which a hypoxic micro-environment has been reported to be a major/key parameter [10]–[12]. Several hypotheses have been proposed to explain this observation. First, a mechanism of adaptation is initiated, mediated by the HIF-1 transcription factor, to enhance cell survival [13]. Second, the cell response to hypoxic conditions also triggers the angiogenesis process [14]. Lastly, hypoxia has been reported to affect the selection of high potential metastatic cells [15]. As this manuscript focuses on the bioinformatics analysis of the data, we direct the reader to the following reviews for a more detailed discussion of the role of hypoxia in the development of metastasis [16]–[18].

### Microarrays

In the last decade, the availability of microarray datasets in public repositories has grown dramatically (i.e. ArrayExpress [19], GEO [20]...). As an example, the number of datasets in the Gene Expression Omnibus (GEO) has increased from 2,000 to more than 780,000 over the last ten years (2002–2012). Previously, most researchers focused on a small handful of probe sets spotted on the arrays, ignoring thousands of other probe sets. Despite the financial cost associated with creating large collections of public datasets (millions of euros/dollars), the incomplete and/or partial analysis of the datasets consequently suggests that a large body of underexploited information could be put to use in further analyses. Many authors has also significantly improved the performance of statistical analyses by solving methodological issues [21]–[23], and developing the alternative chip definition file (CDF) [24]. We propose to make use of this wealth of information by including several microarray datasets, from experiments studying similar/common biological issues, in a single analytical pipeline that makes use of the latest and best-performing algorithms, without preconceived biases.

### Data preparation

Datasets must be preprocessed in preparation for statistical analysis to improve the quality of the data (background correction), to allow for a fair comparison between arrays (standardization), and to summarize probe-level intensities to meaningful probe set values [25], [26]. Several benchmarks have previously been reported to assess the performances of preprocessing methods [27], [28].

The last preprocessing step, called summarization, consists of gathering probe-level information regarding the same target. The mapping of the target definition to the probe coordinates on the chips involves a chip definition file (CDF). The annotation of the human genome has improved since the first release of CDFs by the manufacturer (Affymetrix) and several authors have thus reported the need to update the definition of chip definition files [29], [30]. In 2007, Liu *et al* described the affyprobeminer as a tool to ease the mapping of current knowledge to probe sequences in Affymetrix arrays [24]. The authors reported discrepancies ranging from 30 to 50% between standard Affymetrix and remapped chip definition files. Affyprobeminer can also be used to build both transcript- and gene-consistent CDFs, meaning that a probe-set is defined to gather probes that specifically target only one transcript, or gene, respectively.

### Single gene analysis of one dataset

Microarray data can be used to track the expression profile of the transcriptome following a hierarchical strategy that involves many levels of interpretation. The first level refers to individual analyses aimed at inferring the positive/negative regulation of transcripts and/or genes, as defined in the chip definition file (probe set definition in CDF). Wet-lab biologists mainly interpret microarray experiments based on the results of this step. Additional layers of analysis are described briefly in the next subsections (meta-analysis and gene set analysis).

In previous work, we described a relationship between the number of replicates and the selection of the best performing methods [31]. The two main results are that the best method overall is the Shrinkage *t* test [22], bested solely by the Window t test [32] and Regularized *t* test [21] when only two replicates are available; the other main result is that the overall power of such an analysis is relatively low, depending on the number of replicates available. Therefore, the authors claimed that future methodological developments should focus on augmenting that power and on an appropriate filtering of the results.

### Annotation of a list of candidate genes

After the individual analyses, the list of genes detected as differentially expressed is typically annotated using over-representation analysis (ORA) methods to highlight meaningful information. In a previous work, we described the use of the DAVID webtools to perform such an analysis on the results of microarray studies [33]. The DAVID webtool analyzes the list of differentially expressed genes and returns a list of the pathways containing part of these genes, associated with an over-representation score (EASE score) [34].

### Differential expression analysis of gene sets

Small datasets with only a few replicates are still a major hindrance to statistical power in conventional analyses. Gene set analysis and meta-analysis are interesting and common ways to extract more information from the data, and to test higher-level hypotheses with a power level associated with an increased number of available values.

Gene set analysis using Functional Class Scoring methodologies (FCS) has improved the understanding of differences in expression profiles, and helped unravel the biological processes underlying experimental data in several ways. First, joint analysis of multiple genes involves a higher number of values than individual analyses, hence providing the potential for a higher power level, even when conducted on small datasets (small number of replicates). Second, computation of differential expression from multiple levels of interpretation enriches the qualitative description of biological variations between experimental conditions. The criteria used to define the gene sets consequently guide interpretation of the results (i.e. regulation element/transcription factor, metabolic pathways, pathology signatures, locus, cellular components...). By extension, the comparison of the results of individual and gene set analyses allows, as with ORA methods, to refine the list of candidate genes for further testing, thanks to the criteria-based approach (i.e. if all but one gene of a set of related genes are detected as "silenced" due to deletion, one can remove this potential false negative or screen the genome for an additional copy of the gene).

Over the last decade, various Functional Class Scoring methodologies (FCS) have been developed to analyze gene sets, including 2-step or global methods, competitive or self-contained null hypothesis and inference (gene-sampling, label-sampling...): GSEA [35], [36], SAMGS [37], GlobalTest, [38]...

Method-specific biases in the detection of gene sets are associated with methodological choices, and are due to correlations between genes, the simultaneous presence of up/down regulated genes, the level of expression and the number of genes in the set.... In order to detect all kinds of sets with an expression profile that differs between conditions, we developed FAERI, tailored from the two-way ANOVA [39]. Prior to analysis, FAERI applies a 2-step data reduction to avoid previously observed biases. The null distribution can then be evaluated from simulations or sample permutations. Performance comparisons conducted both on simulated and biological data illustrate that FAERI, evaluated using sample permutations, provides the most accurate results versus other methods, regardless of the composition of the gene sets (in terms of direction, level of expression, correlation and proportion of DEGs in the set). Mansmann and Meister similarly reported that sample permutations of microarray data should be preferred for evaluation of the null distribution in the GlobalAncova methodology, due the variability observed with real samples [40]).

### Meta-analysis

Meta-analysis is a natural extension of the dataset-based analysis conducted using individual and gene set methodologies, and examines several datasets relating to similar experimental conditions. A meta-analysis strategy was reported previously by Simpsons *et al* in 1904 [41] and has been extensively used in the field of medical sciences [42]–[44].

To identify commonly regulated genes in multiple datasets, a higher-level analysis must be defined as opposed to the dataset-specific strategies described above. The ideal meta-analysis design would consist of the joint analysis of multiple datasets following a higher-order multivariate analysis procedure. However, post-hoc strategies require less computing time than full-on transversal analyses, which still remains a major concern in the analysis of large datasets. In a previous study, we explored an intersection-based post-hoc strategy, defined as an additional analytical step performed on results generated with several dataset-specific analyses [45].

To compare the results of differential expression analyses of genes (or gene sets) across datasets, we reported use of the number of dataset-specific analyses that result in a significant detection of the gene (the number of top-lists in which each gene is present). This score, which monitors systematic differences in expression profiles across datasets, was then used as a selection criterion to define candidate genes. The reported strategy leads to three situations, depending on the strictness of the comparison across datasets: 1) the selection of genes that are detected in all (or the highest number of) datasets (intersection of all top-lists) results in a very low number of genes, which are often already well known; 2) selection of the genes detected in at least one dataset (union of all top lists) results in too many candidates for further investigation, and does not exclude false positives; 3) a balance can be reached between both situations, with an intermediate selection threshold at the number of DEGs across datasets. That intermediate situation (union of intersections between a given number of top lists) allows for inference of a workable amount of new candidates. Along these lines, several techniques have been developed to describe the intersections between lists of genes [33], [45], [46].

### Aim of this study

**Figure 1:**
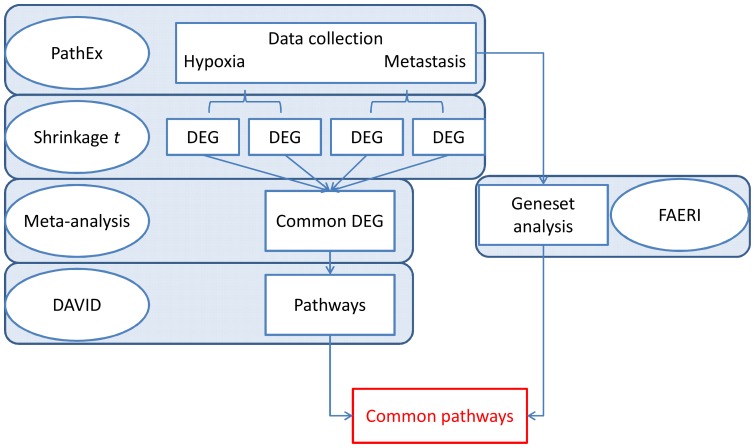
Summary of the analytical pipeline.

We propose to use a set of statistical and bioinformatics tools to reanalyze metastasis and hypoxia-related data to gain further insight into the processes involved. The comparison of two analytical pipelines (ORA and FCS) is used to detect meaningful pathways (a diagram of the analytical pipeline is shown in figure 1). Moreover, this analysis rationale could be transposed to virtually any biological situation with microarray data available.

## Results and Discussion

A major biological topic of interest in our lab is the investigation of expression profiles to describe common mechanisms between metastasis and adaptation of cells to hypoxic conditions. PathEx [47] was queried (performed on data present in PathEx in June 2012) with the keywords “hypoxia” and “metastasis” to identify datasets available from Affymetrix HGU-133a and HGU-133Plus2 arrays. We found 7 and 9 experiments focused on hypoxia and metastasis respectively. The datasets selected (16) and are listed and described in Table 1.

**Table 1:** List of the 16 datasets used in this manuscript.

Dataset Identifier	Number of replicates (Control + Tests)	Biological context
E-MEXP-445	6 (3+3)	Normoxia vs. hypoxia (monocytes)
GSE4725	6 (2+4)	Normoxia vs. hypoxia (arterial smooth muscle cells)
GSE11341	23 (6+17)	Normoxia vs. hypoxia (lung cells)
E-MEXP-1896	4 (2+2)	Normoxia vs. hypoxia (HEK293T cells)
GSE4086	4 (2+2)	Normoxia vs. hypoxia (lymphocytes B)
GSE5579	4 (2+2)	Normoxia vs. hypoxia (lymphatic endothelial cells)
GSE9234	6 (3+3)	Normoxia vs. hypoxia (HT-29 cells)
E-GEOD-1323	6 (3+3)	Primary tumor vs. metastasis (colon cancer cell lines)
E-GEOD-2280	27 (8+19)	Primary tumor vs. metastasis (lymph node cells)
GSE7929	32 (21+11)	Primary tumor vs. metastasis (A 375 metastatic cell line)
GSE7930	6 (3+3)	Primary tumor vs. metastasis (prostate subcutaneous tumors)
GSE7956	39 (29+10)	Primary tumor vs. metastasis (A 375 metastatic cell line)
GSE8401	83 (31+52)	Primary tumor vs. metastasis (melanoma cells)
GSE3325	19 (13+6)	Primary cancer vs. metastasis (prostate cancer cells)
GSE8977	22 (15+7)	Primary cancer vs. metastasis (breast cancer cells)
GSE9576	12 (9+3)	Primary cancer vs. metastasis (Midgut carcinoid liver cells)

### Meta-analysis and over-representation in pathways

**Figure 2:**
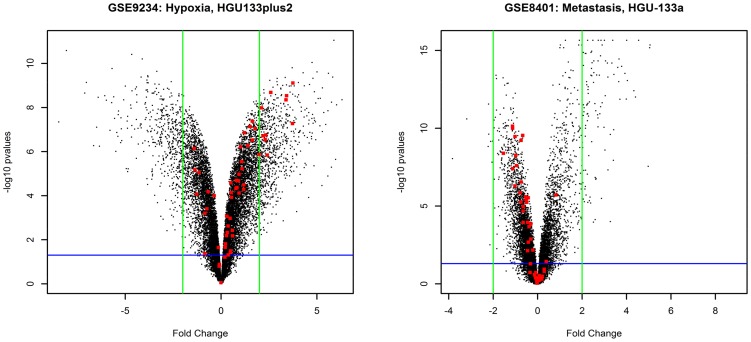
Volcano plots for two datasets. Each volcano plot is related to a single data set, chosen among the different technologies and biological group tested. The green bars represent fold change log_2_ values of +–2 and the blue bar represent a p-value threshold of 0.05. The red dots are the 1156 genes selected in the meta-analysis step.

In the first analytical step, the individual analyses of differential expression for each dataset were performed using the Shrinkage *t* methodology, which produced 16 lists of dataset-specific p-values. Volcano plots are provided in figure 2 for two of the individual datasets, to illustrate the distribution of significant values in a separate analysis. The most interesting genes are usually identified, in such graphs, in the upper left- and right-hand corners of the plot, depicting genes with low p-values (Y-axis) and high fold changes (X-axis). The meaning of the red dots is explained below.

A meta-analysis was performed in two steps to refine the list of significant genes and to define a unique top list from the 16 lists of p-values. Significant genes were first gathered from the two categories of experiments, producing two lists of detected genes (respectively specific to hypoxia and metastasis). The intersection of both lists was then performed, as described in the materials and methods section, to identify candidate genes expected to be involved in both hypoxia and metastasis, while removing potential false detections from the large lists retrieved in the first step. The meta-analysis yielded substantially different results, as shown in figure 2 by the repartition of red dots (final DEGs detected).

Table S1 provides the list of 1156 candidates identified in the meta-analysis procedure, and figure 2 shows the scattering of the candidates in volcano plots for 2 of the 16 datasets we analyzed. The wide range of values observed in figure 2 is due to the variability of the results between the 16 dataset-specific lists (p-values), and the well-known under-estimation of fold changes in microarray experiments [48]. Meta-analysis does not select the most differentially expressed genes in single experiments. As we selected the DEGs across different biological conditions, we can hypothesize that they are representative of the common components of the cellular responses to these situations, which fits well with the purpose of this study.

The list of the identifiers for the 1156 genes obtained after the meta-analysis step was then entered into the DAVID web tool. A total of 102 pathways containing at least 3 gene members of that list was generated (see Table S2). Among these pathways, only 12 of them have an EASE score under the threshold of 0.05. This number was further reduced to 3 pathways by applying a correction for multiple testing (Benjamini correction) and only one pathway (the spliceosome) was significant when applying a correction based on the false discovery rate (FDR) (see Table 2). However, the EASE score (and the corrected p-values derived from it) should be interpreted with caution, according to the biological relevance in the context studied, the wideness of the pathways stored in the Kegg maps and the obvious rate of false negatives induced by our screening. Many top list pathways, although characterized by low EASE scores, are well-known to be involved in metastatic processes and are therefore likely false negatives: MAPK and Wnt signaling pathways, focal adhesion pathway and the regulation of the actin cytoskeleton [49]–[52], which corroborates the consistency of the mapping of significant genes by our strategy. On the other hand, the robustness of the spliceosome pathway with regards to the most stringent statistical corrections supports the hypothesis of its implication in the process studied.

**Table 2:** List of pathways identified by DAVID with either a significant p-value or 14 or more genes of the 1156 DEG list detected in the map.

Pathway	Count	EASE score	Benjamini	FDR
Spliceosome	30	**2.15E-07**	**3.75E-05**	**2.64E-04**
Cell Cycle	25	**6.33E-05**	**5.49E-03**	7.75E-02
Glycolysis / Gluconeogenesis	14	**9.55E-04**	**4.07E-02**	1.16E+00
Aminoacyl-tRNA biosynthesis	10	**5.21E-03**	1.66E-01	6.19E+00
Pentose phosphate pathway	7	**1.39E-02**	2.93E-01	1.57E+01
Cysteine and methionine metabolism	8	**1.87E-02**	3.37E-01	2.07E+01
Prion diseases	8	**2.18E-02**	3.47E-01	2.37E+01
Lysosome	17	**2.94E-02**	4.05E-01	3.06E+01
One carbon pool by folate	5	**3.73E-02**	4.52E-01	3.72E+01
Antigen processing and presentation	13	**3.80E-02**	4.29E-01	3.77E+01
Pyruvate metabolism	8	**4.25E-02**	4.41E-01	4.12E+01
Purine metabolism	20	**4.51E-02**	4.37E-01	4.32E+01
Oocyte meiosis	15	6.63E-02	5.04E-01	5.68E+01
Pathways in cancer	34	1.29E-01	6.33E-01	8.16E+01
Ubiquitin mediated proteolysis	16	1.55E-01	6.63E-01	8.74E+01
Wnt signaling pathway	16	2.60E-01	7.31E-01	9.75E+01
MAPK signaling pathway	23	5.34E-01	8.58E-01	1.00E+02
Focal adhesion	15	7.85E-01	9.59E-01	1.00E+02
Regulation of actin cytoskeleton	15	8.59E-01	9.74E-01	1.00E+02

The ‘count’ column shows the number of significant genes identified within a pathway. Significant p-values (either the EASE score, the Benjamini-corrected or the FDR-corrected p-values) are shown in bold.

**Figure 3:**
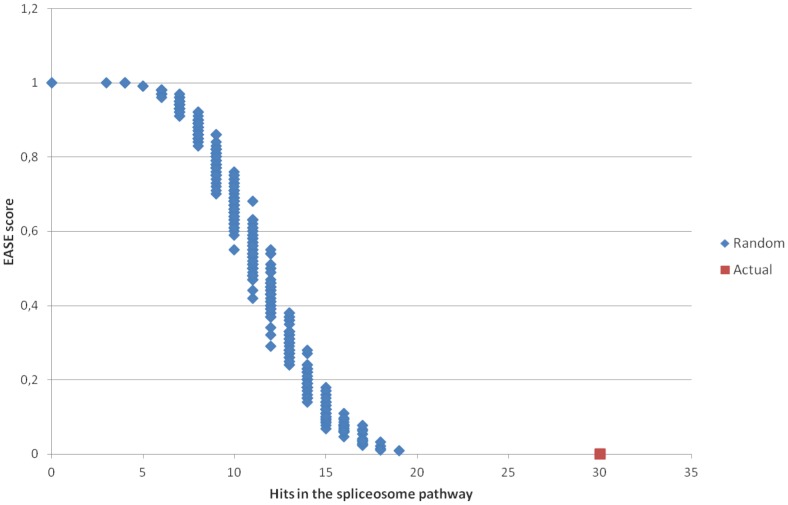
Plot of the EASE score and number of hits in the spliceosome pathway for the 500 random selections of 1156 gene identifiers (in blue), compared with the actual result of the analysis (red). This graph plots the number of hits (X-axis) against the EASE score (Y-axis). The difference between the random selection scores and the actual result score supports the assumption that the spliceosome is over-represented in our list of genes.

To further assess the significance of the spliceosome pathway in the over-representation results, we first performed 500 random selections of 1156 EntrezGeneIDs among all the identifiers present on the microarray and ran them in the DAVID tool. The EASE scores and number of hits in the spliceosome pathway were then plotted (see figure 3). The plot shows clearly the gap between the random selections (with a maximum of 19 hits and an associated EASE score of 0.0085) and the actual result (30 hits, EASE score of 2E-7). Then, we analyzed the robustness of the discovery of the spliceosome pathway by performing a more stringent selection in the meta-analysis step (see Table 3). This table shows the EASE score obtained for the spliceosome pathway when performing a meta-analysis for genes differentially expressed in two (one in each biological group), four (two in each group) or six (three in each group) of the 16 datasets. The spliceosome pathway was largely significant even in the most stringent selection (EASE score of 4E-4). These comparisons tend to support the assumption that the spliceosome pathway is actually over-represented in our meta-analysis results.

**Table 3:** Robustness analysis of the spliceosome pathway enrichment.

DEG # threshold	Count	EASE score
2 (1 and 1)	30	2.15E-07
4 (2 and 2)	23	3.40E-05
6 (3 and 3)	14	4.10E-04

The strictness of the meta-analysis step was increased (selection of genes DEGs in 2 datasets out of 16 available, then 4 datasets out of 16 and 6 datasets out of 16; the number between brackets represent the minimal number of datasets for which the genes have to be DEG to be selected per biological group, i.e. hypoxia or metastasis), the count of genes highlighted in the pathway (second column) and EASE score given by DAVID (third column).

**Figure 4:**
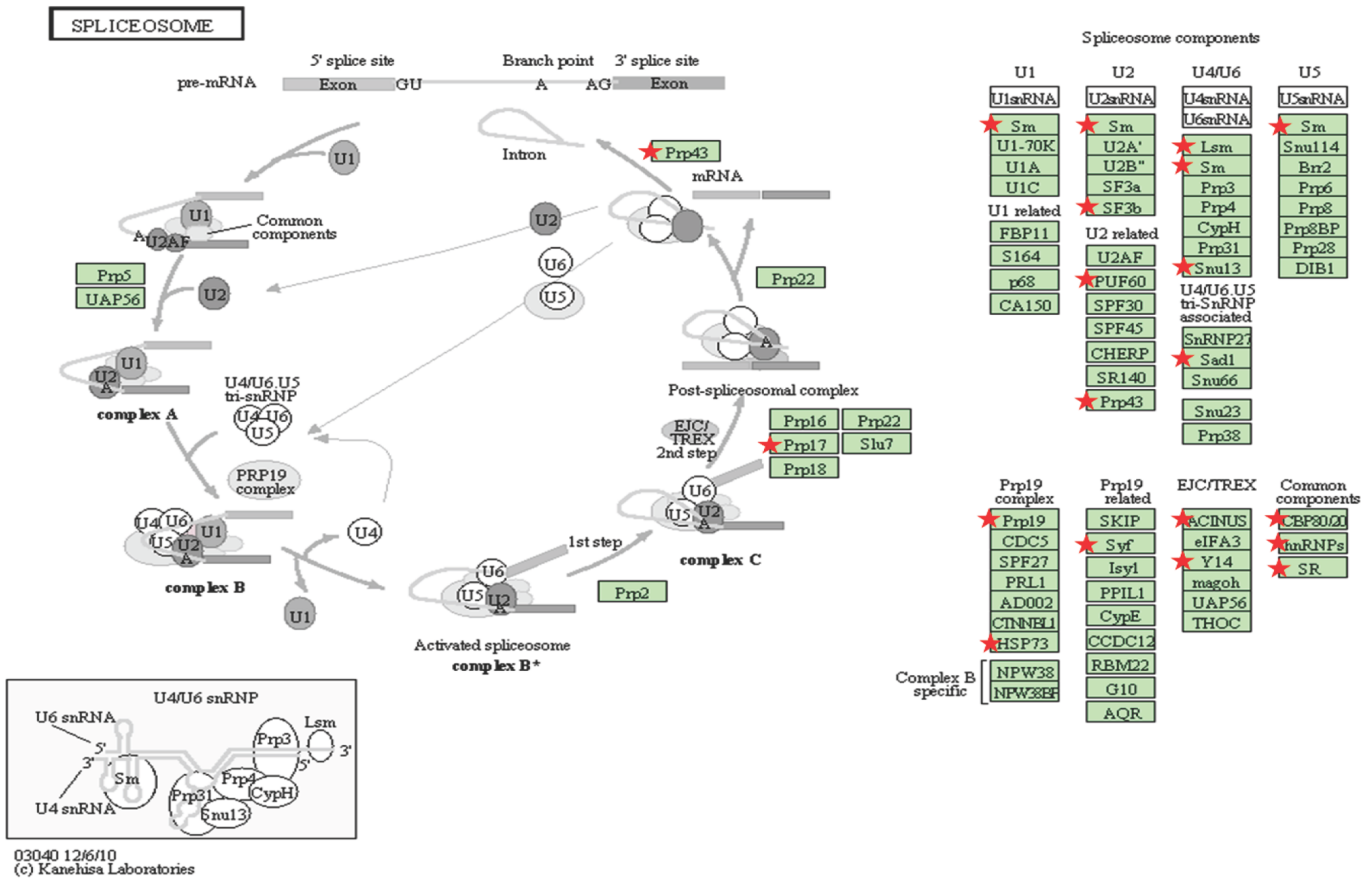
Spliceosome units. The red stars mark the genes from our list mapped on this pathway.

Moreover, the spliceosome, whose implication in cancer has been reported by several authors [53]–[55], has never been described as specifically involved in metastasis, which is not surprising based on the red dots in our volcano plots from single analyses (figure 2). The spliceosome is a complex of RNA and many protein subunits required for the splicing of pre-mRNA. It is composed of five small nuclear RNA (snRNA) and numerous associated protein factors. Proteins and snRNA form the RNA-protein complexes (snRNP), called U1, U2, U4, U5 and U6 (see figure 4). The list of genes detected as differentially expressed contains genes coding for proteins that take part in the spliceosome pathway (see Table S4). The results of our analysis identify genes in all 5 snRNPs, reinforcing the hypothesis that this pathway plays an important role in metastatic and hypoxic processes. The list of genes detected as differentially expressed and their respective p-values per dataset are presented in the Table 4.

**Table 4:** This table contains the genes identified as DEG in the spliceosome pathway, with the p-values for the respective datasets.

	Hypoxia							Metastasis									
Number of replicates	6	6	23	4	4	4	6	6	27	32	6	39	83	19	22	12	
Datasets	E-MEXP-445	GSE4725	GSE11341	E-MEXP-1896	GSE4086	GSE5579	GSE9234	E-GEOD-1323	E-GEOD-2280	GSE7929	GSE7930	GSE7956	GSE8401	GSE3325	GSE8977	GSE9576	Count
DHX 15	0,67	8,98E-01	**4,93E-02**	**1,98E-03**	**8,16E-03**	6,72E-01	**5,85E-04**	1,65E-01	2,73E-01	**2,73E-04**	3,48E-01	3,80E-01	**2,49E-02**	**3,18E-09**	3,54E-01	**1,71E-02**	8
LSM6	1,35E-01	**4,59E-02**	8,95E-01	5,69E-01	**2,06E-02**	2,57E-01	**6,51E-03**	2,16E-01	4,92E-01	**2,09E-03**	2,43E-01	3,77E-01	**1,41E-06**	**1,04E-02**	6,51E-01	1,16E-01	6
NAA38	**2,09E-02**	3,13E-01	4,17E-01	8,58E-01	7,11E-01	7,72E-01	**1,18E-04**	4,19E-01	**3,28E-02**	6,66E-02	1,27E-01	5,34E-01	**3,38E-10**	**4,34E-03**	3,60E-01	7,51E-01	5
NHP2L1	5,89E-02	7,13E-01	2,65E-01	**1,16E-02**	2,23E-01	9,53E-01	**4,75E-07**	1,89E-01	**2,16E-02**	7,66E-01	9,27E-02	1,16E-01	**7,92E-04**	**4,67E-02**	5,21E-01	4,95E-01	5
PRPF19	7,29E-01	**2,94E-02**	1,94E-01	**3,24E-02**	**6,95E-03**	4,29E-01	**3,00E-07**	5,13E-02	5,74E-01	1,24E-01	**2,27E-02**	8,74E-01	3,40E-01	5,30E-01	**1,12E-02**	1,71E-01	6
RBM8A	**2,40E-02**	7,83E-01	5,74E-01	3,66E-01	**5,26E-03**	9,17E-01	**4,89E-02**	**1,23E-02**	2,35E-01	**2,91E-02**	**2,31E-02**	6,42E-01	1,89E-01	8,44E-02	4,33E-01	**1,55E-02**	7
ACIN1	**4,05E-02**	9,92E-01	6,55E-01	**2,45E-02**	**9,74E-03**	7,11E-01	**3,20E-02**	**1,98E-04**	7,89E-01	**6,60E-05**	3,58E-01	5,65E-01	5,38E-01	**3,28E-02**	8,78E-01	1,82E-01	7
Cdc40	9,98E-01	7,88E-01	6,80E-01	3,07E-01	6,80E-02	5,20E-01	**1,07E-05**	2,98E-01	9,11E-01	**6,13E-03**	2,26E-01	7,65E-01	1,90E-01	**1,07E-07**	9,77E-01	**3,30E-02**	4
CRNKL1	**3,86E-02**	7,70E-01	**9,38E-03**	2,73E-01	**4,95E-02**	8,02E-01	**4,48E-09**	9,23E-01	2,29E-01	**1,55E-07**	1,35E-01	4,64E-01	**3,93E-08**	1,09E-01	6,34E-01	**4,83E-02**	7
HSPA1A	**4,37E-02**	9,59E-01	**4,66E-05**	**5,14E-03**	**3,13E-03**	2,63E-01	**1,15E-03**	9,60E-01	1,48E-01	**2,18E-08**	1,34E-01	**8,94E-03**	**2,58E-08**	**2,80E-06**	1,05E-01	1,61E-01	9
HSPA1B	**1,92E-02**	4,38E-01	3,65E-01	9,44E-01	3,12E-01	8,72E-01	**8,89E-06**	**1,20E-02**	5,61E-01	**6,93E-04**	3,68E-01	7,69E-01	1,57E-01	**4,47E-02**	9,09E-01	**1,00E-02**	6
HSPA8	**1,36E-03**	8,33E-01	6,60E-01	3,76E-01	6,38E-02	7,44E-01	**2,61E-02**	**9,19E-04**	1,65E-01	5,99E-01	3,84E-01	3,47E-01	**6,00E-08**	4,91E-01	8,76E-01	**2,63E-02**	5
HNRNPA1P2	2,02E-01	4,42E-01	6,08E-01	5,30E-01	1,37E-01	8,63E-01	**8,68E-07**	9,41E-01	9,27E-01	1,73E-01	2,33E-01	4,23E-01	9,38E-02	**4,20E-07**	3,42E-01	**4,48E-02**	3
HNRNPC	**1,12E-02**	**3,97E-02**	8,77E-01	**4,84E-02**	**2,12E-04**	3,44E-01	**3,52E-06**	2,76E-01	1,99E-01	**5,13E-05**	1,53E-01	9,03E-01	5,45E-02	**4,38E-02**	1,06E-01	**4,15E-02**	8
HNRNPK	7,88E-01	**2,29E-02**	7,13E-01	1,84E-01	1,54E-01	4,09E-01	5,49E-01	**4,84E-03**	2,04E-01	**1,70E-05**	2,55E-01	3,03E-01	**1,70E-04**	**4,88E-02**	**5,52E-05**	7,26E-01	6
HNRNPM	3,61E-01	**4,24E-03**	8,85E-01	1,65E-01	6,57E-02	1,00E+00	**1,73E-02**	5,15E-02	7,33E-01	2,36E-01	1,64E-01	9,32E-01	**4,87E-03**	**4,43E-02**	1,24E-01	**2,42E-02**	5
SNRPG	1,92E-01	**1,26E-02**	6,31E-01	**1,35E-02**	**3,70E-02**	2,88E-01	**5,13E-05**	8,38E-01	**3,16E-02**	1,82E-01	2,04E-01	6,29E-01	3,62E-01	**6,22E-06**	**4,60E-03**	8,42E-02	7
NCBP1	5,08E-01	8,32E-01	4,05E-01	**6,80E-04**	2,11E-01	2,35E-01	**4,33E-02**	9,02E-01	**3,87E-02**	**4,27E-02**	3,77E-01	4,40E-01	9,18E-01	**4,73E-02**	**5,94E-05**	1,52E-01	6
PUF60	4,94E-01	**2,77E-02**	3,31E-01	6,24E-02	**2,65E-03**	4,65E-01	**2,20E-02**	**5,57E-04**	2,72E-01	2,23E-01	2,56E-01	3,27E-01	4,63E-01	**2,18E-02**	1,56E-01	7,61E-01	5
SNRPD1	**8,54E-03**	7,39E-01	3,47E-01	7,28E-01	1,10E-01	1,80E-01	**6,56E-03**	3,67E-01	5,41E-01	2,50E-01	**4,22E-02**	3,55E-01	**5,27E-03**	**1,57E-03**	6,02E-01	**3,64E-02**	6
SNRPD3	7,61E-01	1,50E-01	4,52E-01	1,33E-01	9,09E-01	4,06E-01	**1,03E-08**	**3,06E-02**	**4,87E-02**	**1,67E-04**	7,68E-02	1,39E-01	**2,09E-05**	**5,97E-04**	**4,31E-02**	9,36E-01	7
SNRPEL1	8,86E-01	**2,49E-02**	3,98E-01	**1,69E-02**	**3,23E-02**	6,56E-01	**2,37E-03**	**1,81E-03**	9,32E-02	2,46E-01	**1,78E-02**	1,79E-01	4,44E-01	**5,25E-03**	7,15E-01	5,34E-01	7
SNRPF	8,75E-01	**4,83E-03**	6,61E-01	4,42E-01	**2,30E-03**	9,50E-01	**4,19E-05**	7,66E-01	5,57E-01	**1,46E-05**	7,80E-02	7,65E-01	**2,10E-03**	**4,13E-02**	2,44E-01	**3,11E-02**	7
SF3B3	5,74E-01	2,51E-01	9,73E-01	5,13E-01	1,71E-01	7,57E-01	**6,26E-04**	1,22E-01	4,35E-01	8,87E-01	4,24E-01	6,75E-01	5,96E-01	**1,67E-02**	6,29E-02	6,00E-02	2
SFRS1	2,00E-01	1,36E-01	9,40E-01	2,07E-01	1,37E-01	2,82E-01	3,64E-01	9,54E-01	7,99E-01	**3,65E-03**	**3,66E-02**	6,54E-01	6,22E-02	**7,35E-03**	5,09E-02	6,60E-02	3
SFRS3	1,87E-01	**7,79E-03**	2,18E-01	1,12E-01	4,32E-01	4,39E-01	**1,94E-07**	**3,27E-02**	3,56E-01	**1,70E-05**	**3,53E-03**	2,22E-01	**1,39E-04**	**1,12E-02**	9,77E-02	5,78E-01	7
SFRS5	4,54E-01	1,20E-01	4,66E-01	7,16E-02	6,68E-02	6,26E-01	**5,31E-08**	**6,83E-03**	**2,61E-02**	7,19E-01	7,70E-01	3,13E-01	**7,40E-03**	**7,61E-06**	**2,99E-02**	9,04E-01	6
SFRS7	**3,09E-02**	7,38E-01	6,83E-01	8,52E-01	**2,31E-03**	5,91E-01	**4,21E-02**	8,86E-01	8,82E-01	6,88E-01	6,32E-01	6,10E-01	2,92E-01	8,91E-02	8,23E-01	5,35E-02	3
SFRS9	**4,39E-03**	2,41E-01	1,02E-01	8,61E-02	8,89E-01	6,60E-01	**9,99E-05**	**5,64E-04**	2,77E-01	**1,72E-02**	**2,77E-02**	7,59E-01	**2,93E-06**	**7,74E-04**	5,24E-01	**7,95E-03**	8
TRA2B	**1,00E-02**	1,02E-01	4,29E-01	2,93E-01	**1,84E-03**	8,13E-01	**7,30E-08**	**5,67E-03**	**2,50E-02**	1,34E-01	2,50E-01	1,89E-01	**4,05E-09**	**2,67E-02**	**1,54E-02**	1,39E-01	8
USP39	1,25E-01	8,78E-01	6,01E-01	5,23E-01	1,32E-01	4,12E-01	**6,60E-05**	3,48E-01	6,97E-01	**1,07E-02**	2,63E-01	3,00E-01	2,71E-01	**2,56E-05**	1,65E-01	**4,24E-02**	4

The significant p-values are highlighted in bold. The last columns ‘count’ shows the number of times a given gene is detected as DEG through all 16 datasets.

### Gene set analysis

The second part of the analytical pipeline (figure 1) relies on the inference of differentially expressed pathways in a gene set analysis procedure (functional class scoring). Here, we used FAERI, a multivariate procedure tailored from the two-way ANOVA procedure. FAERI computes a gene set statistic from the expression data of all member genes in a single step, and avoids the loss of information inherent to 2-step procedures and the risk of false negatives due to slight differences in all member genes (that would not be individually detected in the first part of the pipeline). In addition, FAERI relies on a self-contained procedure (label sampling) that only requires the expression values of the set of member genes (and not the complete dataset). Table 5 summarizes the results obtained by individual analysis of the 16 selected datasets conducted with FAERI. These results were then used to compute, for each gene set, a ratio of discovery across all the experiments (Table 5, third column). The definition of the sets was retrieved from the C2.Kegg category of MsigDB (v3.0). The full list of p-values is provided in Table S3.

**Table 5:** Summary of FAERI results.

Gene set Name	5% Hypoxia	5% Metastasis	% Total
KEGG_PATHWAYS_IN_CANCER	7	9	100
KEGG_GLYCOLYSIS_GLUCONEOGENESIS	7	8	93.75
KEGG_PURINE_METABOLISM	7	8	93.75
KEGG_RIBOSOME	7	8	93.75
KEGG_PPAR_SIGNALING_PATHWAY	7	8	93.75
KEGG_MAPK_SIGNALING_PATHWAY	7	8	93.75
KEGG_ERBB_SIGNALING_PATHWAY	7	8	93.75
KEGG_CYTOKINE_CYTOKINE_RECEPTOR_INTERACTION	7	8	93.75
KEGG_CHEMOKINE_SIGNALING_PATHWAY	7	8	93.75
KEGG_ENDOCYTOSIS	7	8	93.75
KEGG_APOPTOSIS	7	8	93.75
KEGG_VEGF_SIGNALING_PATHWAY	7	8	93.75
KEGG_FOCAL_ADHESION	7	8	93.75
KEGG_REGULATION_OF_ACTIN_CYTOSKELETON	7	8	93.75
KEGG_ADIPOCYTOKINE_SIGNALING_PATHWAY	7	8	93.75
KEGG_HUNTINGTONS_DISEASE	7	8	93.75
KEGG_PANCREATIC_CANCER	7	8	93.75
KEGG_CHRONIC_MYELOID_LEUKEMIA	7	8	93.75
KEGG_RENAL_CELL_CARCINOMA	7	8	93.75
KEGG_CITRATE_CYCLE_TCA_CYCLE	6	8	87.5
KEGG_FRUCTOSE_AND_MANNOSE_METABOLISM	7	7	87.5
KEGG_OXIDATIVE_PHOSPHORYLATION	6	8	87.5
KEGG_VALINE_LEUCINE_AND_ISOLEUCINE_DEGRADATION	6	8	87.5
KEGG_GLUTATHIONE_METABOLISM	6	8	87.5
KEGG_AMINO_SUGAR_AND_NUCLEOTIDE_SUGAR_METABOLISM	7	7	87.5
KEGG_PYRUVATE_METABOLISM	6	8	87.5
KEGG_RNA_DEGRADATION	6	8	87.5
KEGG_SPLICEOSOME	6	8	87.5
KEGG_CALCIUM_SIGNALING_PATHWAY	6	8	87.5
KEGG_NEUROACTIVE_LIGAND_RECEPTOR_INTERACTION	6	8	87.5
KEGG_CELL_CYCLE	6	8	87.5
KEGG_OOCYTE_MEIOSIS	6	8	87.5
KEGG_P53_SIGNALING_PATHWAY	6	8	87.5
KEGG_UBIQUITIN_MEDIATED_PROTEOLYSIS	6	8	87.5
KEGG_LYSOSOME	6	8	87.5
KEGG_MTOR_SIGNALING_PATHWAY	6	8	87.5
KEGG_VASCULAR_SMOOTH_MUSCLE_CONTRACTION	6	8	87.5
KEGG_WNT_SIGNALING_PATHWAY	6	8	87.5
KEGG_AXON_GUIDANCE	6	8	87.5
KEGG_ECM_RECEPTOR_INTERACTION	6	8	87.5
KEGG_ADHERENS_JUNCTION	6	8	87.5
KEGG_TIGHT_JUNCTION	6	8	87.5
KEGG_GAP_JUNCTION	6	8	87.5
KEGG_ANTIGEN_PROCESSING_AND_PRESENTATION	6	8	87.5
KEGG_TOLL_LIKE_RECEPTOR_SIGNALING_PATHWAY	6	8	87.5
KEGG_RIG_I_LIKE_RECEPTOR_SIGNALING_PATHWAY	6	8	87.5
KEGG_JAK_STAT_SIGNALING_PATHWAY	6	8	87.5
KEGG_NATURAL_KILLER_CELL_MEDIATED_CYTOTOXICITY	6	8	87.5
KEGG_T_CELL_RECEPTOR_SIGNALING_PATHWAY	6	8	87.5
KEGG_B_CELL_RECEPTOR_SIGNALING_PATHWAY	6	8	87.5
KEGG_FC_EPSILON_RI_SIGNALING_PATHWAY	6	8	87.5
KEGG_FC_GAMMA_R_MEDIATED_PHAGOCYTOSIS	6	8	87.5
KEGG_LEUKOCYTE_TRANSENDOTHELIAL_MIGRATION	6	8	87.5
KEGG_NEUROTROPHIN_SIGNALING_PATHWAY	6	8	87.5
KEGG_LONG_TERM_DEPRESSION	6	8	87.5
KEGG_ALZHEIMERS_DISEASE	6	8	87.5
KEGG_PARKINSONS_DISEASE	6	8	87.5
KEGG_VIBRIO_CHOLERAE_INFECTION	6	8	87.5
KEGG_EPITHELIAL_CELL_SIGNALING_IN_HELICOBACTER_PYLORI_INFECTION	6	8	87.5
KEGG_LEISHMANIA_INFECTION	6	8	87.5
KEGG_COLORECTAL_CANCER	6	8	87.5
KEGG_PROSTATE_CANCER	6	8	87.5
KEGG_MELANOMA	6	8	87.5
KEGG_BLADDER_CANCER	7	7	87.5
KEGG_ACUTE_MYELOID_LEUKEMIA	6	8	87.5
KEGG_SMALL_CELL_LUNG_CANCER	6	8	87.5
KEGG_VIRAL_MYOCARDITIS	6	8	87.5

The first column presents the name of the gene sets tested, the second and third columns show the number of times each gene set was detected as differentially expressed at a threshold of 5% for the p-values, for each biological group. The last column contains the discovery rate across all experiments (7 hypoxia datasets and 9 metastasis datasets).

Table 5 summarizes the information contained in Table S3 and highlights the high number of differentially expressed sets across both categories of experiments. The pathways identified by FAERI are involved in glycolysis, neoglucogenesis, tricarboxylic cycle, oxidative phosphorylation and other sugar metabolism pathways. These results are relevant to the cell/tissue response to hypoxic conditions. Here, only one gene set was detected across all datasets: PATHWAYS_IN_CANCER. Many other cancer-related gene sets were detected in all but one experiment. Several signaling pathways were also systematically called differentially expressed, including PPAR, ERBB, MAPK, VEGF, P53, MTOR, WNT, … The pathway for the regulation of the actin cytoskeleton was also detected. The hypothesis of involvement of the spliceosome is supported by 6 out of 7 datasets related to hypoxia and 8 out of 9 datasets related to metastasis.

Both parts of the analytical pipeline described here have detected the spliceosome pathway as involved in the hypoxic and metastatic phenotypes. Among the 31 genes detected as differentially expressed in this pathway, 11 have recently been shown to be involved in the metastatic process (see Table 6). The remaining 20 genes are not yet known to be involved in these processes (see Table 6, in bold). These results suggest that abnormal alternative splicing regulation can modulate the metastatic potential of cancer cells. Indeed, it is known that the recognition of splicing sites depends on the protein composition of the spliceosome [56]. Dysregulated expression of the genes coding for these proteins could therefore change the composition of the spliceosome architecture, thus affecting the splicing process. A change in the splicing process may influence the cell at all biochemical levels, from the transcriptome to the proteome and even to the genome. The 20 genes we have identified thus hold strong potential as candidates for further studies.

**Table 6:** List of the 31 genes highlighted in the spliceosome pathway.

Gene	References in literature
***DHX 15***	NA
***LSM6***	NA
***NAA38***	NA
***NHP2L1***	NA
***PRPF19***	NA
*RBM8A*	Kim *et al*, 2008 [60] and Salicioni *et al*, 2000 [61]
*ACIN1*	Lee *et al*, 2008 [62] and Shu *et al*, 2006 [63]
***Cdc40***	NA
***CRNKL1***	NA
***HSPA1A***	NA
***HSPA1B***	NA
***HSPA8***	NA
***HNRNPA1P2***	NA
*HNRNPC*	Park *et al*, 2012 [64]
*HNRNPK*	Inoue *et al*, 2007 [65] and Li *et al*, 2011 [66]
*HNRNPM*	Palermo *et al*, 2012 [67] and Thomas *et al*, 2011 [68]
***SNRPG***	NA
***NCBP1***	NA
***PUF60***	NA
***SNRPD1***	NA
*SNRPD3*	Cunha *et al*, 2010 [69]
***SNRPEL1***	NA
***SNRPF***	NA
***SF3B3***	NA
*SFRS1*	Mukherji *et al*, 2006 [70], Hatakeyama *et al*, 2009 [71] and Meseguer *et al*, 2011 [72]
***SFRS3***	NA
*SFRS5*	Hatakeyama *et al*, 2009 [71]
*SFRS7*	Hatakeyama *et al*, 2009 [71]
*SFRS9*	Mukherji *et al*, 2006 [70]
*TRA2B*	Watermann *et al*, 2006 [73]
***USP39***	NA

Eleven of those genes are previously known in the literature to be involved in metastasis (shown in grey), the 20 other are previously unknown (shown in bold) to be involved in the metastatic process.

The results also demonstrate the potential of sensitive and specific analytical pipelines: new hypotheses can be proposed, and previously known biological features can be used as positive controls. However, comparison of the results between both parts of the analytical pipeline suggests that the two analyses behave differently: over-representation analysis of the most significant genes across datasets detects some important pathways, and the ability of gene set analysis using FAERI to detect slight cumulated differences detects more pathways. Statistical analysis with FAERI detects meaningful differences between samples, even when only small numbers of replicates are available. Nevertheless, both parts of the pipeline lead to detection of relevant information based on current knowledge, and both suggest the involvement of the spliceosome.

## Conclusion and Perspectives

We implemented a pipeline of bioinformatics tools to explore archived microarray data, from preprocessing to mapping of the results. We used that pipeline to examine metastasis and hypoxia data and found results in keeping with previous reports, as well as a new hypothesis. The combination of high-level analysis (Over Representation Analysis and Functional Category Scoring) with a meta-analysis step led to the discovery of involvement of the spliceosome in the hypoxic and metastatic processes, and the generation of a list of 20 new candidate genes.

Bioinformatics approaches will never replace bench validations; however we were able to form a plausible hypothesis just by re-analyzing available data. Biological investigations should therefore be performed to further refine the interpretation of the relationships between the pathways detected and understand how a hypoxic environment and metastasis affect both general and energetic cell metabolism. Further investigations should be conducted to clarify the results of the statistical analyses and to discriminate between causes and consequences (mechanisms of perturbations and symptoms). However, that validation is out of the scope of this methodological paper.

We think that this analytical protocol could be used successfully in many other biological contexts, wherever several datasets are available. Indeed, we have shown that single gene analysis alone yields poor results, though this is often the only step performed by wet-lab biologists. The methodology presented here allows for improved performance, comparison with previously known information and discovery of recurrent patterns (through meta-analysis), all of which were performed using freely-available resources and software packages and without the need to perform expensive *de novo* microarray experiments. We think that this work will contribute to the creation of a virtual atlas for cellular biology containing the known characteristics of cells in diverse biological conditions, which is one of the major goals of the bioinformatics community.

## Methods

### Selection and retrieval of datasets

For the purposes of the study reported here, two sets of criteria were used to retrieve datasets with PathEx (described in [47]): technological keywords to specifically retrieve Affymetrix GeneChips HGU-133a and HGU-133plus2 array models; and biological keywords to retrieve datasets that met the topics of interest in this study: hypoxia or metastasis.

Entry of these technological and biological keywords into PathEx resulted in a collection of 16 distinct datasets, as listed in Table 1: 9 datasets specific to hybridizations performed on the HGU-133a chip model, including 3 experimental designs dedicated to hypoxia and 6 dedicated to metastasis; 7 datasets obtained using the HGU-133plus2 array model, including 4 hypoxia-related and 3 metastasis-related experiments. The number or replicated measurements ranged from 2 to 52 hybridizations (see Table 1). In addition, we preferred datasets reporting *in vivo* gene expression levels and discarded data that came from *in vitro* experiments.

### Preprocessing and statistical analyses

The preprocessing of the data and the individual analyses reported in this paper were performed using R 2.7 and 2.10, available on the website of the R-Project (http://cran.r-project.org), and a set of packages available in the Bioconductor repository (http://www.bioconductor.org).

We used GCRMA to preprocess each of the 16 retrieved datasets, in accordance with the performances reported in previously reported benchmarks [26], [57], [58]. The summarization step performed by GCRMA was guided by the affyprobeminer transcript-consistent chip definition files (CDF) specific to the HGU-133a and HGU-133plus2 chip models. The probe set identifiers provided by alternative CDFs (affyprobeminer) differ from the identifiers defined by the manufacturer of the arrays (Affymetrix). Supplemental functions implemented in the affyprobeminer packages were used to convert probe set identifiers into EntrezGeneID. The identification of probe sets with EntrezGene ID identifiers allowed us to compare the gene lists between HGU-133a and HGU-133plus2 chip models, and to facilitate annotation of the results from the individual analyses.

The differential expression of individual probe sets was analyzed with the 'st' package, which implements the Shrinkage *t* methodology. This procedure was conducted on each dataset, resulting in 16 dataset-specific lists of p-values, each p-value referring to a specific probe set.

### Meta-analysis, annotation, and gene set analysis

For each dataset, we selected the list of genes detected as differentially expressed (p-value < 0.05). The 16 dataset-specific lists of the most significant genes were gathered into two groups, according to the experimental design (Hypoxia/Metastasis studies). In each group of datasets, a new list of genes was defined from the list of genes found to be differentially expressed in at least one dataset of the group. Lastly, the intersection of the list of genes from the two groups was performed by selecting genes that were detected in both groups, resulting in a list of 1156 unique gene identifiers (provided in Table S1, along with all the p-values computed for the 16 datasets, p-values ranking for each dataset and mean ranking across the 16 individual ranks).

The 1156 selected EntrezGene ID identifiers were mapped to the Kegg Pathways database using the “Functional Annotation Tools” available on the DAVID web interface [59]. Using DAVID, 102 pathways, containing at least 3 of the 1156 candidate genes, were identified (see Table S2). To avoid biases due to potential false positives, we selected for further analysis the pathways that displayed a significant p-value (see Table 2).

Alongside the selection and annotation of the most significant genes by the meta-analysis approach, differential expression analyses of gene sets were conducted on each of the 16 datasets. Gene set analyses were performed on preprocessed data in a single step using the multivariate FAERI test. Gene set definitions were retrieved from the MSigDB database (v3.0) [36]. We evaluated the differential expression on gene sets belonging to the C.2 KEGG category, composed of 186 curated pathways. Lastly, the 16 dataset-specific lists of p-values were used to compute, for each gene set, the ratio of detection as differentially expressed across all datasets. The full code for this analysis can be found in the Table S5. For more details on the FAERI methodology, see [39].

The different steps in the analytical pipelines are summarized in figure 1. The left part of the diagram contains the single gene analysis steps (Shrinkage *t* test treatment, meta-analysis and over-representation analysis (ORA) in DAVID). The right part contains the gene set analysis steps (Functional Class Scoring (FCA) by FAERI and meta-analysis of the results).

## Supporting Information

Table S1 Full list of p-values obtained for each dataset for each of the 1156 genes highlighted in the analysis.
(PDF)

Table S2 List of pathways highlighted in the over-representation analysis using DAVID.
(PDF)

Table S3 Full list of p-values obtained in the geneset analysis.
(PDF)

Table S4 Distribution of the highlighted genes in the spliceosome pathway.
(PDF)

Table S5 R code for the full analysis.
(PDF)

Checklist S1 (PDF)
