# Supplementary material for: Meta-Analysis and Gene Set Analysis of Archived Microarrays Suggest Implication of the Spliceosome in Metastatic and Hypoxic Phenotypes
Source: PLoS One. 2014 Jan 31;9(1):e86699. doi: 10.1371/journal.pone.0086699 (PMC3908947; doi:10.1371/journal.pone.0086699)
Supplement: Table S1 — Full list of p-values obtained for each dataset for each of the 1156 genes highlighted in the analysis. (PDF) [file pone.0086699.s001.pdf]

EntrezID	RefSeq MRNA	GeneSymbol	E-MEXP-445	Rank 1	GSE_4725	Rank 2	GSE_11341	Rank 3	E-GEOD_1323	Rank 4	E-GEOD_2280	Rank 5	GSE_7929	Rank 6	GSE_7930	Rank 7	GSE_7956	Rank 8	GSE_8401	Rank 9	Compte	E-MEXP_1896	Rank 10	GSE_4086	Rank 11	GSE_5579	Rank 12	GSE_9234	Rank 13	GSE_3325	Rank 14	GSE_8977	Rank 15	GSE_9576	Rank 16	Compte	Compte Total	Mean Rank Tota
205	NM_013410	AK3L2	1.16E-02	186	3.01E-04	22	2.28E-02	90	8.65E-05	76	5.90E-02	113	5.45E-04	390	5.00E-02	271	3.24E-01	640	1.35E-01	774	5	9.90E-04	47	1.30E-05	24	3.20E-01	324	4.52E-06	291	1.18E-03	434	1.08E-05	21	7.95E-02	399	5	10	256.38
8974	NM_001017974	P4HA2	4.39E-03	104	5.65E-03	112	2.47E-01	349	1.12E-07	3	4.63E-02	95	1.45E-06	179	3.54E-02	222	4.72E-02	263	7.11E-01	1050	7	1.21E-05	4	3.17E-03	269	3.64E-01	400	6.20E-11	2	7.00E-12	3	2.30E-01	619	2.28E-01	632	4	11	269.13
5033	NM_006191	PA2G4	9.05E-05	17	9.84E-05	15	3.62E-02	126	1.33E-03	185	6.01E-01	750	7.02E-02	745	5.16E-01	893	2.14E-01	539	8.00E-08	74	5	6.93E-04	43	2.70E-05	35	2.60E-01	230	4.13E-10	5	4.31E-10	17	7.62E-02	438	7.63E-02	391	4	9	281.44
55055	NM_017994	c7orf42	1.43E-02	209	1.31E-01	570	3.80E-01	480	8.14E-04	161	2.24E-02	39	9.26E-04	423	1.69E-02	142	4.22E-02	250	1.27E-11	10	7	2.46E-04	15	1.47E-05	28	6.53E-01	780	1.41E-08	30	1.07E-06	120	8.98E-02	462	5.08E-01	842	4	11	285.06
3419	NM_001166550	ids	8.19E-01	1003	4.11E-02	429	2.02E-02	76	5.10E-02	513	3.41E-02	70	3.05E-13	18	8.10E-03	102	8.34E-06	5	3.84E-07	99	7	5.77E-02	300	1.16E-04	80	8.76E-01	1005	9.68E-07	202	6.13E-07	99	1.76E-01	565	5.10E-02	322	3	10	305.50
2821	NM_002082	grk6	2.37E-04	29	1.16E-02	180	6.23E-01	758	2.94E-07	5	3.63E-01	479	2.40E-03	474	1.82E-01	552	2.81E-03	60	4.27E-02	661	6	2.78E-02	216	2.06E-06	8	1.14E-01	62	2.22E-09	11	4.65E-07	92	2.36E-01	627	4.56E-01	808	4	10	313.88
908	NM_001009186	cct6a	1.97E-01	607	2.94E-03	73	1.30E-01	233	5.41E-01	909	4.00E-01	518	3.97E-08	98	1.84E-01	557	2.48E-02	182	1.11E-03	372	4	1.73E-03	56	1.33E-03	207	3.06E-01	304	7.65E-08	77	2.08E-02	671	1.29E-04	55	1.11E-02	117	6	10	314.75
29078	NM_014165	Ndufaf4	6.59E-01	889	4.10E-01	769	2.62E-02	101	5.47E-03	275	1.64E-01	227	3.22E-11	31	1.72E-02	143	1.18E-02	121	1.91E-05	190	6	5.09E-04	31	9.53E-03	371	3.87E-01	442	5.69E-08	63	8.85E-03	586	1.33E-03	102	5.05E-01	840	5	11	323.81
79833	NM_024789	TMEM180	5.28E-01	829	5.40E-03	109	1.34E-02	67	9.09E-03	321	2.47E-02	46	6.23E-06	210	7.69E-01	1032	2.11E-03	53	4.39E-05	222	7	7.74E-03	119	2.80E-02	486	3.30E-01	342	1.01E-06	206	1.50E-05	210	1.09E-03	96	5.25E-01	858	5	12	325.38
10184	NM_005779	LHFPL2	1.15E-03	50	5.88E-01	872	4.24E-01	522	7.45E-03	307	3.80E-01	501	4.39E-09	64	5.27E-01	905	1.51E-01	454	1.29E-03	380	4	1.55E-03	55	7.73E-05	65	8.82E-02	42	2.74E-06	265	8.50E-06	190	1.48E-02	229	4.90E-02	310	6	10	325.69
6513	NM_006931	SLC2A3	5.96E-02	484	9.89E-06	8	3.14E-03	26	1.96E-05	44	6.41E-01	800	2.87E-01	924	1.20E-02	117	6.55E-02	305	2.79E-13	3	5	1.02E-03	48	4.23E-04	139	2.81E-01	261	4.06E-07	152	3.74E-06	164	5.42E-01	865	6.77E-01	952	4	9	330.75
5352	NM_182676	Pltp	7.45E-02	501	3.20E-03	77	1.00E-02	61	3.32E-02	447	1.00E-01	167	5.66E-05	292	6.17E-02	324	7.79E-01	991	5.74E-06	159	5	6.18E-04	36	2.24E-01	789	2.10E-01	175	7.65E-09	22	6.50E-04	396	6.90E-04	86	7.03E-01	974	4	9	343.56
133	NM_001124	ADM	2.93E-06	4	1.71E-06	5	1.57E-01	256	2.63E-01	743	6.28E-01	783	1.41E-04	331	2.73E-01	685	2.25E-04	22	9.91E-04	364	5	4.71E-04	26	2.88E-02	487	4.10E-01	476	3.26E-09	14	6.70E-06	182	1.57E-02	241	5.64E-01	883	5	10	343.88
9111	NM_004688	nmi	1.60E-02	222	1.41E-01	577	1.45E-01	242	2.72E-05	54	3.51E-01	466	6.44E-14	12	3.62E-03	63	4.63E-02	260	3.62E-01	917	5	4.76E-01	793	1.65E-02	416	4.86E-01	588	4.06E-08	47	2.29E-03	470	4.78E-02	372	1.57E-03	23	5	10	345.13
6237	NM_002957	RXRA	4.32E-02	432	5.32E-02	504	5.03E-01	615	3.37E-05	57	8.37E-01	990	1.21E-08	80	4.32E-03	73	7.14E-02	318	5.79E-04	323	5	3.33E-01	664	4.24E-02	538	3.57E-01	388	6.84E-07	179	3.20E-07	84	1.37E-02	216	6.94E-03	75	5	10	346.00
2012	NM_001428	eno1	2.45E-02	302	5.49E-03	110	3.85E-01	484	1.36E-02	355	1.77E-01	241	7.49E-10	51	1.37E-03	33	1.05E-02	113	3.53E-02	638	7	8.97E-01	1082	5.23E-02	559	5.31E-01	647	2.03E-08	33	4.41E-03	526	4.75E-05	32	5.87E-02	356	3	10	347.63
4601	NM_015675	Gadd45b	2.15E-03	73	6.63E-04	32	1.40E-01	240	3.15E-02	435	7.36E-01	892	1.64E-10	39	7.44E-02	359	8.77E-02	345	2.30E-01	844	4	2.85E-04	17	7.91E-05	66	4.24E-01	496	4.40E-08	52	5.47E-01	1039	1.62E-04	57	2.58E-01	656	4	8	352.63
57228	NM_020675	spc25	1.88E-01	598	4.62E-02	465	1.29E-01	231	9.62E-03	327	6.12E-02	119	2.29E-12	22	5.78E-02	306	4.76E-02	265	1.15E-07	79	5	2.79E-02	217	3.47E-01	924	6.34E-02	31	4.73E-05	464	3.06E-01	956	3.90E-05	31	2.09E-01	612	3	8	352.94
440	NM_001673	asnS	6.08E-01	862	7.21E-02	523	1.01E-02	62	5.42E-06	22	1.87E-01	253	2.39E-03	473	1.10E-01	438	6.40E-01	885	3.83E-04	305	4	1.70E-02	167	1.14E-01	661	3.08E-01	306	1.56E-08	31	8.61E-12	4	1.49E-03	108	1.66E-01	555	4	8	353.44
6515	NM_001134367	slc6a6	9.21E-05	18	1.16E-05	9	4.91E-03	35	2.03E-06	14	2.75E-01	379	4.86E-05	285	7.83E-02	376	4.71E-03	76	9.43E-01	1134	6	2.17E-03	64	4.88E-05	49	2.77E-01	257	3.78E-01	1043	3.09E-02	720	1.32E-01	517	3.95E-01	766	3	9	358.88
10808	NM_006644	HSPH1	7.95E-01	979	1.05E-02	170	4.53E-02	151	7.40E-06	29	5.67E-01	710	1.19E-05	227	4.21E-01	823	2.02E-01	526	6.58E-06	164	5	2.31E-02	193	2.96E-08	2	2.99E-01	291	3.76E-06	286	1.33E-05	205	7.78E-01	1015	5.03E-05	2	5	10	360.81
665	NM_004331	BNIP3L	1.26E-04	19	8.08E-04	37	2.00E-02	75	2.18E-02	393	5.89E-01	735	6.30E-01	1058	1.37E-01	480	2.54E-01	579	4.73E-03	472	5	6.69E-04	39	1.28E-05	23	4.29E-01	506	5.73E-09	19	6.90E-07	103	3.15E-01	698	1.98E-01	598	4	9	364.63
3418	NM_005530	idh3a	1.42E-01	572	3.06E-02	353	9.40E-01	1110	5.20E-03	269	1.73E-01	236	3.02E-11	30	2.90E-02	199	4.14E-02	246	2.75E-02	607	6	4.54E-02	275	9.49E-03	370	2.39E-01	203	4.99E-06	299	1.95E-02	664	1.39E-02	220	2.54E-02	201	6	12	365.88
3475	NM_001551	IGBP1	6.37E-01	872	3.62E-01	743	3.00E-02	110	1.03E-02	333	6.04E-02	115	5.80E-03	525	2.85E-02	197	2.67E-02	189	3.23E-06	139	6	1.43E-02	150	1.20E-03	201	3.83E-01	436	8.83E-08	82	1.30E-02	623	5.78E-02	401	3.63E-01	743	4	10	366.19
11130	NM_007057	ZWINT	1.22E-01	555	4.96E-02	494	9.86E-02	203	3.12E-04	102	5.87E-02	112	5.73E-04	394	1.17E-03	28	1.37E-01	427	1.12E-06	113	5	9.87E-01	1147	3.55E-05	39	4.83E-01	585	5.53E-08	62	5.59E-05	278	1.47E-02	228	8.93E-01	1095	4	9	366.38
6281	NM_006513	SARS	2.09E-02	266	1.50E-02	211	1.70E-01	274	3.63E-02	459	9.31E-01	1080	1.49E-08	84	3.98E-02	237	1.11E-03	38	4.27E-08	68	7	1.53E-02	156	4.90E-05	50	7.38E-01	864	7.24E-04	653	1.60E-09	21	7.04E-01	970	1.36E-01	505	4	11	371.00
51444	NM_016271	RNF138	2.34E-02	292	1.16E-01	556	1.21E-01	223	2.72E-03	221	2.34E-03	8	2.41E-02	644	1.94E-02	159	4.43E-01	742	1.05E-07	77	6	2.19E-01	531	6.97E-03	341	4.87E-01	591	4.68E-05	462	5.07E-03	540	1.70E-02	246	5.66E-02	351	4	10	374.00
10397	NM_001135242	ndrg1	1.48E-03	60	7.16E-03	130	8.60E-04	17	1.93E-02	380	3.74E-01	492	7.07E-05	305	4.22E-01	824	2.84E-02	197	6.70E-08	73	7	5.10E-02	286	1.38E-01	700	4.52E-01	549	1.69E-10	3	9.05E-01	1130	1.55E-02	240	2.97E-01	694	2	9	380.00
3939	NM_002300	ldhb	1.06E-03	48	1.96E-03	61	4.15E-02	140	1.17E-02	345	7.80E-01	935	7.33E-04	411	2.46E-01	649	1.12E-01	388	7.68E-01	1067	5	2.62E-05	7	5.44E-04	150	1.35E-01	83	4.22E-08	50	3.32E-05	242	8.55E-01	1057	1.02E-01	450	4	9	380.19
55818	NM_021729	VPS11	2.48E-03	79	6.10E-05	13	1.06E-02	63	6.22E-03	285	4.95E-01	638	8.24E-01	1111	1.48E-01	494	5.34E-01	805	1.77E-01	811	4	1.23E-03	52	3.43E-04	120	4.08E-01	472	6.66E-08	70	3.71E-04	360	4.09E-02	353	7.10E-02	383	5	9	381.81
3726	NM_002267	kpna3	2.83E-01	682	2.58E-05	11	9.25E-01	1091	1.57E-05	41	4.38E-01	572	1.05E-07	118	3.02E-03	58	7.22E-03	93	3.59E-07	98	6	4.16E-02	264	5.37E-01	1025	4.08E-01	471	1.78E-05	390	1.37E-05	207	6.53E-02	414	1.82E-01	581	3	9	382.25
4783	NM_021705	NFYA	3.77E-02	395	2.53E-08	2	1.47E-01	245	3.04E-02	429	2.16E-01	292	4.13E-02	695	5.71E-02	303	4.76E-01	762	2.86E-01	875	4	6.19E-04	37	1.20E-03	200	4.15E-01	485	2.13E-06	253	5.67E-03	551	2.21E-03	120	1.17E-01	477	5	9	382.56
3251	NM_005345	HSPA1A	4.37E-02	437	9.59E-01	1128	4.66E-05	2	9.60E-01	1138	1.48E-01	213	2.18E-08	91	1.34E-01	474	8.94E-03	105	2.58E-08	61	5	5.14E-03	105	3.13E-03	267	2.63E-01	236	1.15E-03	680	2.80E-06	153	1.05E-01	482	1.61E-01	550	4	9	382.63
57122	NM_020441	CORO1B	8.58E-01	1022	1.92E-02	250	7.90E-02	193	6.68E-05	71	3.46E-01	463	4.15E-02	696	6.18E-02	325	9.76E-01	1137	7.71E-03	504	4	3.52E-02	242	6.70E-04	163	2.61E-01	232	2.68E-07	130	2.67E-08	46	1.52E-01	532	2.19E-02	183	5	9	386.81
26502	NM_012336	NARF	1.36E-02	204	6.44E-02	517	5.48E-01	675	9.46E-06	34	5.39E-01	674	5.11E-04	386	3.97E-02	234	2.60E-01	583	8.71E-02	732	4	4.57E-03	95	2.33E-03	245	4.78E-01	580	6.47E-07	176	6.05E-06	177	7.22E-03	180	3.38E-01	722	5	9	388.38
51065	NM_015920	Rps27l	2.31E-02	289	4.09E-01	768	8.74E-01	1040	9.01E-03	320	3.01E-01	410	1.55E-03	450	3.89E-02	231	7.64E-05	12	5.56E-10	27	6	5.09E-01	817	3.57E-05	40	1.49E-01	98	2.43E-02	859	6.77E-05	289	1.26E-01	511	6.57E-03	71	4	10	389.50
4616	NM_002473	MYH9	1.90E-02	246	5.18E-04	30	8.10E-01	966	1.05E-02	335	9.81E-02	159	4.36E-01	1003	3.06E-03	59	1.83E-01	501	1.05E-02	532	5	1.81E-01	490	1.30E-04	84	2.41E-01	205	1.29E-08	27	4.79E-05	266	2.27E-02	279	8.97E-01	1098	4	9	392.50
54541	NM_019058	ddit4	1.77E-02	233	4.18E-06	6	3.38E-01	442	2.42E-03	209	3.25E-01	437	4.18E-01	991	2.56E-01	665	8.91E-02	348	1.61E-01	795	3	4.98E-04	30	6.03E-07	6	6.67E-01	795	5.57E-07	163	5.26E-09	27	7.44E-01	995	1.78E-02	158	5	8	393.75
5604	NM_002760	PRKY	1.98E-02	251	2.50E-02	298	5.54E-02	167	2.13E-02	390	2.41E-01	330	1.95E-14	10	3.26E-02	215	4.07E-02	243	8.01E-02	721	6	4.20E-04	25	3.54E-06	12	7.70E-01	903	1.05E-04	520	2.03E-04	337	7.68E-01	1008	5.76E-01	889	4	10	394.94
1992	NM_001419	ELAVL1	5.31E-01	831	2.57E-01	678	2.37E-02	93	2.15E-04	90	2.42E-02	44	4.21E-11	32	1.28E-03	30	3.93E-02	235	2.51E-01	851	6	1.37E-01	433	2.38E-01	804	3.73E-01	413	4.23E-05	454	1.26E-08	39	5.73E-01	886	8.52E-02	413	2	8	395.38
5230	NM_002633	pgm1	2.11E-05	11	3.51E-02	383	3.61E-04	8	5.99E-01	944	5.60E-01	699	1.13E-03	433	6.56E-01	976	1.78E-04	20	1.03E-06	110	6	1.22E-03	51	1.61E-04	94	3.46E-01	369	1.01E-07	92	6.21E-02	799	2.41E-01	630	3.77E-01	752	3	9	398.19
10019	NM_005475	SH2B3	2.59E-01	655	2.47E-02	293	1.70E-01	273	8.74E-02	572	1.16E-01	184	5.65E-12	25	9.42E-02	410	4.86E-02	269	4.75E-08	70	4	7.14E-01	946	3.63E-04	127	2.03E-01	162	1.78E-03	702	1.93E-01	904	1.75E-02	252	1.53E-01	533	3	7	398.56
2026	NM_004436	ENSA	1.39E-04	22	1.58E-03	53	7.19E-04	14	2.66E-01	745	9.60E-01	1108	1.29E-05	228	9.02E-01	1105	1.16E-02	120	1.25E-02	550	6	1.63E-02	162	3.00E-01	884	3.53E-01	383	5.37E-07	160	2.56E-04	348	1.84E-02	258	3.31E-02	247	5	11	399.19
10054	NM_005499	UBA2	1.18E-03	54	2.13E-01	640	2.87E-01	387	1.91E-03	198	9.82E-02	160	6.13E-08	109	3.57E-01	767	1.02E-01	372	3.68E-05	213	4	2.36E-02	199	5.21E-03	317	3.27E-01	333	1.86E-03	704	7.79E-03	576	2.41E-01	629	3.62E-01	742	4	8	400.00
2941	NM_005513	GTF2E1	5.74E-01	847	2.43E-02	285	9.49E-01	1119	9.94E-01	1154	1.78E-02	32	1.35E-13	14	3.44E-04	12	3.18E-02	209	3.77E-06	147	6	1.15E-02	137	2.47E-01	816	2.39E-02	15	7.15E-06	324	6.92E-08	60	7.24E-03	181	8.63E-01	1074	5	11	401.63
10598	NM_012111	AHSA1	2.90E-01	686	2.21E-02	272	2.90E-01	391	1.41E-03	191	9.07E-01	1056	3.48E-04	376	7.11E-02	347	1.71E-03	46	8.85E-03	516	5	1.32E-02	142	5.70E-03	323	7.93E-01	928	2.58E-07	126	2.33E-05	221	1.15E-01	498	5.03E-02	320	4	9	402.44
664	NM_004052	BNIP3	2.76E-04	31	8.29E-06	7	4.47E-05	1	9.77E-01	1150	8.48E-01	1002	2.83E-04	366	7.41E-01	1018	5.35E-03	81	1.76E-01	810	5	1.66E-06	2	1.38E-04	87	2.09E-01	172	4.75E-01	1065	1.73E-04	331	1.88E-03	115	2.85E-02	223	5	10	403.81
51514	NM_016448	dtl	8.89E-01	1051	3.77E-02	404	9.33E-02	201	9.19E-03	323	3.10E-02	63	2.13E-01	876	3.33E-02	218	3.38E-01	651	1.16E-08	53	5	8.57E-04	46	8.21E-05	68	5.21E-01	637	1.26E-06	224	1.93E-06	136	1.18E-01	501	7.82E-01	1017	4	9	404.31
5066	NM_032456	PCDH7	6.43E-02	488	2.94E-02	338	2.79E-01	374	2.97E-01	762	9.84E-02	161	3.98E-08	99	9.74E-01	1148	5.84E-02	287	1.13E-02	539	3	2.43E-03	69	1.06E-05	21	4.88E-01	593	8.44E-08	80	6.73E-08	59	9.12E-01	1096	6.37E-02	366	4	7	405.00
51567	NM_016614	Ttrap	5.00E-03	118	3.70E-01	748	2.85E-01	383	4.30E-01	843	1.43E-01	207	5.98E-10	48	6.17E-01	956	2.96E-01	616	3.43E-07	97	3	6.08E-02	308	2.29E-02	459	4.11E-02	26	4.96E-01	1072	5.27E-07	95	5.97E-02	407	8.66E-03	101	4	7	405.25
55893	NM_199263	THSD1	1.02E-05	7	5.44E-02	505	4.43E-04	9	9.18E-03	322	2.86E-01	391	6.37E-01	1061	9.45E-01	1127	9.11E-02	350	3.25E-04	300	4	3.64E-04	21	3.77E-05	41	2.98E-01	290	1.79E-02	836	3.22E-01	959	5.21E-03	162	1.03E-02	110	5	9	405.69
8407	NM_003564	TAGLN2	3.72E-03	96	8.14E-02	529	5.46E-01	672	8.42E-06	33	9.58E-01	1107	1.32E-08	82	2.68E-01	681	7.46E-03	95	1.38E-07	81	5	2.94E-02	222	4.25E-02	539	3.38E-01	354	1.69E-06	236	3.04E-08	48	6.21E-01	919	4.66E-01	815	4	9	406.81
1026	NM_005195	cebpd	4.95E-01	813	9.46E-01	1121	5.94E-03	43	3.09E-01	774	3.67E-01	484	2.11E-09	58	3.24E-02	213	2.10E-01	535	9.01E-03	519	4	2.78E-01	603	2.07E-02	446	1.34E-02	8	4.72E-08	55	7.23E-03	569	4.32E-03	151	1.19E-02	118	6	10	406.88
3945	NM_006499	LGALS8	4.72E-02	457	2.16E-02	270	2.40E-01	344	1.52E-03	194	5.38E-01	669	1.41E-01	831	5.46E-02	290	1.25E-01	407	4.26E-04	312	4	4.66E-01	785	4.75E-04	145	2.11E-01	177	2.32E-05	412	1.62E-01	892	4.47E-03	153	2.11E-02	177	4	8	407.19
5289	NM_000297	Pkd2	3.61E-01	737	1.17E-02	181	4.55E-01	555	5.49E-01	916	5.06E-02	103	7.17E-02	747	1.68E-01	531	2.09E-01	533	1.20E-06	114	2	5.72E-05	8	1.73E-02	423	2.37E-01	201	4.43E-05	457	1.73E-02	652	4.01E-02	347	1.00E-03	19	6	8	407.75
2745	NM_006877	gmpr	3.48E-03	94	6.31E-01	901	6.54E-01	795	4.46E-04	126	4.79E-01	619	5.49E-09	67	4.05E-01	816	8.37E-05	14	4.25E-01	945	4	9.20E-01	1100	4.77E-03	310	3.39E-02	22	2.25E-06	255	4.02E-06	166	4.56E-03	154	1.86E-02	164	6	10	409.25
55148	NM_018109	MTPAP	1.47E-01	574	8.65E-03	149	5.22E-01	643	2.49E-01	732	1.67E-01	233	1.22E-06	176	1.67E-01	530	5.95E-02	289	1.45E-03	387	3	7.36E-02	336	1.58E-02	414	2.35E-01	197	4.54E-04	624	4.30E-03	520	6.48E-02	413	5.31E-02	334	3	6	409.44
7167	NM_001159287	TPI1	9.45E-04	46	7.80E-04	36	5.87E-02	173	2.85E-01	759	2.53E-01	351	7.66E-05	311	1.47E-02	130	1.07E-01	381	8.52E-01	1097	4	2.51E-01	572	5.74E-06	15	2.05E-01	168	3.67E-07	144	6.17E-03	558	5.93E-01	901	6.76E-01	951	3	7	412.06
1528	NM_004938	DAPK1	1.96E-01	606	7.41E-01	983	3.02E-02	112	1.51E-06	12	3.68E-01	487	3.91E-07	147	8.46E-02	389	5.86E-02	288	1.61E-05	184	4	3.31E-01	660	1.43E-03	211	6.04E-01	731	1.90E-07	114	4.32E-01	1000	1.13E-01	492	2.65E-02	209	3	7	414.06
64981	NM_032409	Pink1	4.97E-02	471	4.51E-01	797	3.29E-02	118	5.86E-04	139	2.13E-02	36	7.03E-04	410	2.32E-01	626	1.80E-03	47	6.53E-02	703	6	2.16E-01	526	1.77E-02	424	2.98E-01	289	4.09E-05	450	4.20E-02	751	1.71E-02	247	1.96E-01	593	4	10	414.19
10728	NM_006601	Ptges3	1.86E-02	242	3.38E-02	374	3.07E-01	416	4.04E-01	833	3.06E-01	416	7.40E-08	112	3.69E-03	65	6.95E-01	930	1.68E-07	84	5	2.99E-01	628	3.77E-02	524	6.23E-01	748	9.88E-05	516	9.17E-07	113	2.06E-01	602	2.08E-03	29	4	9	414.50
7045	NM_003252	Tial1	4.55E-02	446	1.55E-03	51	7.55E-01	905	4.40E-06	20	5.37E-02	107	9.77E-15	8	6.89E-02	344	4.51E-02	255	3.49E-02	635	6	7.47E-01	977	1.62E-01	716	2.04E-01	164	2.28E-04	578	5.84E-06	175	9.01E-01	1086	2.03E-02	174	3	9	415.06
29923	NM_013332	C7orf68	2.56E-06	2	5.98E-03	116	8.19E-03	53	7.58E-01	1024	5.62E-01	702	8.88E-04	421	5.84E-01	940	8.82E-03	104	6.76E-01	1041	5	5.32E-03	106	4.05E-09	1	8.79E-01	1011	1.43E-09	9	9.65E-11	9	7.62E-01	1003	8.42E-03	99	5	10	415.06
8635	NM_003730	RNASET2	5.69E-05	15	5.30E-01	840	1.80E-01	282	4.33E-05	61	8.15E-01	973	1.21E-02	582	6.87E-02	342	3.02E-01	620	3.96E-01	930	3	1.64E-01	469	6.07E-05	56	5.06E-01	620	1.06E-05	350	2.26E-08	45	1.11E-04	52	8.20E-02	406	4	7	415.19
3091	NM_005338	HIP1	6.83E-02	495	1.65E-03	57	5.21E-03	38	3.54E-02	453	1.60E-01	225	1.28E-07	120	8.19E-01	1071	5.66E-02	281	3.34E-06	143	5	7.53E-01	984	6.56E-08	3	3.52E-01	379	3.91E-04	612	6.12E-01	1056	2.69E-04	67	2.68E-01	668	3	8	415.75
30001	NM_014584	ERO1L	1.63E-05	10	2.77E-04	19	7.90E-04	16	1.24E-01	618	5.50E-01	687	1.42E-02	596	1.23E-01	458	9.82E-01	1144	4.93E-02	671	5	1.46E-01	442	1.91E-04	99	1.41E-01	90	3.35E-07	139	2.24E-07	76	9.77E-01	1151	1.01E-01	446	3	8	416.38
5236	NM_002634	Phb	5.53E-06	6	4.22E-03	90	6.55E-02	179	6.18E-01	953	5.17E-01	653	5.60E-01	1040	4.14E-01	820	2.45E-02	181	4.41E-03	467	4	4.64E-03	97	1.42E-03	210	3.13E-01	313	5.36E-08	60	9.42E-04	421	4.28E-01	789	8.17E-02	404	4	8	417.69
5163	NM_001130863	ENPP2	3.15E-07	1	8.99E-03	153	1.90E-04	6	9.77E-01	1149	4.88E-01	630	9.62E-02	784	1.37E-01	481	9.63E-02	360	3.90E-02	648	4	5.79E-07	1	4.12E-05	43	3.20E-01	322	5.65E-07	167	2.17E-03	465	3.24E-01	705	4.04E-01	773	4	8	418.00
7456	NM_001077269	WIPF1	7.94E-03	153	8.30E-01	1041	8.01E-01	955	3.86E-02	474	5.38E-01	671	6.40E-09	69	1.56E-02	136	2.73E-03	58	3.80E-03	451	6	1.77E-01	485	4.95E-03	312	2.59E-01	226	2.77E-01	1013	9.09E-06	193	6.79E-02	421	5.33E-03	62	3	9	420.00
23030	NM_015015	KDM4B	7.59E-03	149	3.68E-04	24	2.79E-01	373	1.46E-01	647	7.12E-02	132	1.11E-01	803	9.58E-01	1137	7.47E-02	322	1.16E-02	542	3	2.90E-01	613	2.14E-04	105	3.80E-01	430	2.91E-08	38	2.31E-02	684	1.06E-01	483	3.91E-02	271	4	7	422.06
3843	NM_001017402	Lamb3	1.19E-01	553	2.30E-01	649	5.97E-01	734	4.09E-04	123	6.60E-01	821	1.59E-01	839	6.87E-02	343	6.70E-02	309	2.54E-05	199	2	3.04E-02	226	1.82E-02	430	3.06E-01	305	5.84E-08	64	2.54E-02	695	1.16E-03	98	7.69E-02	392	5	7	423.75
10318	NM_006058	Tnip1	2.08E-01	612	3.66E-03	84	6.51E-01	790	4.22E-06	18	6.31E-01	790	7.83E-04	415	2.33E-03	50	4.52E-02	257	2.70E-05	201	6	1.03E-02	131	1.38E-04	86	5.51E-01	671	1.17E-07	97	2.37E-02	686	4.35E-01	795	9.30E-01	1117	4	10	425.00
51637	NM_016039	c14orf16	5.73E-02	480	4.28E-02	442	4.11E-02	138	1.66E-01	670	6.54E-01	812	4.21E-05	279	4.63E-02	262	1.50E-03	42	1.94E-01	821	5	2.35E-02	198	8.41E-05	70	1.75E-01	127	2.88E-05	432	4.45E-04	369	2.56E-01	650	8.08E-01	1026	4	9	426.13

7852	NM_003467	CXCR4	1.74E-03	67	3.00E-04	21	7.95E-01	950	2.59E-05	52	8.68E-01	1021	9.86E-02	787	5.59E-02	297	2.29E-04	23	6.15E-01	1023	4	2.45E-04	14	4.38E-05	45	5.48E-01	666	9.21E-06	339	8.17E-03	581	2.67E-03	128	4.51E-01	805	5	9	426.19
3336	NM_181353	id1	1.71E-01	588	1.00E-03	42	6.87E-01	841	3.24E-01	784	4.30E-01	560	8.15E-03	545	1.33E-01	473	1.07E-01	379	5.59E-13	4	3	6.21E-02	311	8.60E-02	622	2.52E-01	215	3.48E-06	280	6.00E-04	388	2.68E-02	300	1.24E-01	492	3	6	426.50
10007	NM_005471	gnpda1	2.01E-02	254	6.06E-02	511	8.89E-02	199	1.16E-02	344	5.22E-01	657	4.34E-02	702	2.22E-03	47	1.59E-03	43	3.40E-02	632	6	3.16E-02	228	1.29E-01	684	6.99E-03	6	5.83E-01	1092	3.05E-07	83	2.42E-01	631	3.27E-01	713	3	9	426.63
29997	NM_015710	GLTSCR2	1.73E-02	227	6.80E-01	936	6.46E-01	786	3.87E-06	17	8.91E-01	1043	9.50E-06	223	1.90E-02	155	1.71E-01	481	2.31E-09	38	5	3.17E-02	231	6.75E-03	337	6.85E-01	813	7.57E-01	1130	3.39E-06	160	9.63E-07	9	3.24E-02	241	5	10	426.69
64759	NM_194071	CREB3L2	3.11E-02	347	4.56E-02	462	9.96E-01	1156	3.43E-07	6	2.05E-01	274	2.27E-08	92	7.25E-01	1010	1.67E-01	477	1.06E-02	534	5	4.74E-03	99	5.66E-04	153	9.21E-01	1057	5.54E-03	755	6.18E-05	282	5.54E-07	7	1.22E-02	122	6	11	427.06
23338	NM_015288	Phf15	2.40E-02	296	2.52E-02	299	1.61E-01	261	1.89E-02	378	3.77E-01	497	4.30E-08	103	1.56E-03	37	1.70E-02	147	8.36E-01	1092	6	5.92E-01	874	3.63E-03	284	5.40E-01	657	3.64E-05	446	3.57E-02	735	1.85E-01	577	2.02E-02	172	4	10	428.44
4691	NM_002491	NDUFB3	2.17E-02	277	6.78E-03	125	6.89E-01	844	2.84E-04	99	3.59E-01	475	1.99E-02	626	9.14E-01	1115	1.61E-01	468	5.38E-01	993	4	3.64E-02	248	2.58E-02	477	3.83E-01	435	9.20E-07	200	9.04E-08	62	1.04E-04	50	6.80E-02	379	5	9	429.56
3927	NM_005565	lcp2	2.69E-03	82	3.54E-01	738	7.39E-02	187	1.08E-04	79	2.95E-01	402	6.05E-03	527	4.38E-02	252	1.45E-02	135	9.62E-01	1146	5	4.32E-03	91	1.12E-02	382	9.98E-01	1154	1.84E-03	703	8.81E-10	20	2.69E-01	660	4.98E-02	317	5	10	429.69
7422	NM_001025369	VEGFA	5.25E-03	122	8.80E-07	4	1.40E-02	68	9.63E-01	1142	2.86E-01	388	3.06E-01	931	1.38E-01	484	8.68E-03	102	1.00E-01	744	4	2.81E-03	72	9.01E-05	73	3.47E-01	371	7.32E-07	185	9.29E-06	195	5.47E-01	869	9.92E-01	1153	4	8	431.44
3312	NM_004134	HSPA9	4.98E-01	817	6.18E-02	513	6.55E-01	797	2.59E-02	410	6.92E-01	855	4.71E-06	202	6.67E-02	341	1.77E-02	152	2.52E-08	60	4	7.14E-02	327	3.73E-04	129	5.01E-01	614	1.39E-07	106	9.07E-04	420	1.34E-02	214	6.93E-01	965	4	8	432.63
6432	NM_004593	TRA2B	1.00E-02	172	1.02E-01	543	4.29E-01	530	5.67E-03	278	2.50E-02	48	1.34E-01	825	2.50E-01	654	1.89E-01	512	4.05E-09	43	4	2.93E-01	618	1.84E-03	228	8.13E-01	953	7.30E-08	72	2.67E-02	701	1.54E-02	236	1.39E-01	513	4	8	432.88
5705	NM_002807	PSMD1	5.11E-01	823	3.23E-02	365	5.27E-01	649	5.48E-05	68	3.21E-01	435	4.31E-06	200	1.81E-02	148	4.80E-01	766	3.96E-03	453	5	5.00E-02	284	1.59E-04	93	4.36E-01	524	2.91E-06	269	2.57E-06	149	9.09E-01	1092	2.14E-01	617	4	9	433.44
81555	NM_001136043	C15orf44	3.33E-02	359	3.15E-02	358	8.91E-01	1059	5.22E-03	270	1.75E-01	239	4.58E-01	1011	3.51E-01	764	2.14E-01	540	2.74E-09	40	4	4.35E-03	92	3.37E-03	275	5.25E-01	642	2.12E-05	404	4.94E-03	536	1.76E-02	254	1.09E-02	115	6	10	434.88
51097	NM_016002	sccpdh	9.55E-01	1109	3.34E-02	372	7.75E-01	926	5.33E-04	134	9.62E-01	1110	2.30E-04	353	2.29E-03	49	2.04E-01	527	8.89E-04	355	5	2.56E-02	209	2.31E-01	798	2.37E-01	200	2.49E-06	258	9.42E-05	302	2.30E-04	64	2.35E-02	193	5	10	434.94
3183	NM_031369	HNRNPD	2.99E-02	338	1.51E-01	588	9.50E-01	1120	7.38E-03	305	2.20E-02	38	2.65E-06	193	6.82E-01	990	7.45E-01	965	2.53E-06	134	5	2.18E-02	190	1.09E-01	656	3.88E-01	446	2.11E-05	402	1.49E-04	325	1.02E-02	199	7.34E-03	80	5	10	435.56
901	NM_004354	CCNG2	6.61E-03	140	1.20E-02	186	3.99E-01	499	8.98E-01	1099	5.52E-01	688	4.70E-02	706	3.47E-04	13	8.78E-01	1057	8.75E-03	514	5	2.30E-03	65	7.22E-04	166	7.38E-01	863	6.97E-09	20	8.31E-04	416	1.44E-02	224	4.94E-02	315	6	11	435.69
23764	NM_152878	Maff	1.48E-02	212	3.93E-04	26	1.14E-01	216	7.10E-03	302	7.47E-01	901	5.20E-05	290	4.77E-02	264	5.77E-02	286	5.26E-01	988	5	4.73E-04	27	4.54E-03	307	4.88E-01	592	1.12E-06	212	1.60E-01	887	3.10E-02	311	9.94E-01	1156	4	9	436.06
10653	NM_021102	Spint2	6.13E-03	133	2.08E-01	638	2.32E-01	338	2.18E-05	48	5.69E-02	108	4.90E-02	710	3.04E-01	716	7.50E-01	969	2.18E-11	11	4	2.24E-02	192	7.91E-02	608	3.79E-01	427	6.06E-06	313	6.07E-03	556	1.76E-01	564	2.50E-01	648	3	7	436.19
7298	NM_001071	Tyms	8.03E-01	987	3.05E-02	352	1.93E-01	296	3.90E-05	59	2.28E-01	308	5.09E-07	154	1.29E-01	468	3.50E-02	221	9.83E-08	76	5	9.66E-01	1131	1.50E-03	216	2.66E-01	240	1.10E-01	960	2.65E-04	350	2.52E-02	290	5.60E-01	879	3	8	436.69
27229	NM_014444	TUBGCP4	4.13E-01	767	1.98E-03	62	7.65E-01	914	5.63E-01	921	3.05E-01	414	9.11E-01	1137	5.58E-02	295	7.11E-02	317	1.06E-03	368	2	2.32E-03	66	6.52E-02	580	1.53E-01	102	2.63E-04	586	3.58E-06	162	9.93E-03	197	8.46E-03	100	5	7	436.75
51367	NM_015918	pop5	3.72E-01	743	2.72E-02	320	2.57E-01	359	6.46E-04	144	6.66E-02	126	9.28E-04	424	2.85E-01	697	1.02E-01	370	9.56E-01	1139	3	2.06E-01	516	1.63E-03	221	3.99E-01	463	6.15E-05	485	1.12E-06	121	4.82E-01	830	4.63E-03	55	4	7	438.31
5716	NM_002815	Psmd11	1.28E-01	561	1.09E-02	175	2.85E-02	107	2.24E-04	94	2.50E-01	347	4.18E-08	102	9.76E-02	414	6.50E-01	890	7.43E-09	50	5	5.10E-01	820	1.26E-01	680	3.90E-01	450	5.31E-05	476	2.07E-02	670	2.27E-01	618	1.72E-01	568	2	7	438.88
2597	NM_002047	Gars	3.66E-06	5	3.29E-03	80	6.05E-01	746	8.39E-04	163	2.46E-01	341	1.95E-01	866	8.53E-01	1082	1.17E-01	397	6.65E-03	492	4	2.11E-02	186	3.56E-04	123	4.16E-01	488	1.11E-05	355	2.44E-03	478	8.39E-02	447	4.11E-01	780	4	8	439.31
5202	NM_002626	PFKL	5.46E-01	838	1.89E-02	249	2.73E-02	103	2.03E-01	691	6.58E-01	817	2.76E-10	43	7.88E-01	1049	1.28E-02	127	1.28E-05	178	5	7.54E-01	986	8.80E-05	71	3.39E-01	356	5.86E-04	642	1.51E-05	211	1.01E-01	477	2.28E-02	192	4	9	439.38
54165	NM_020640	DCUN1D1	1.09E-02	177	7.78E-01	1010	8.24E-01	988	1.55E-02	363	4.18E-03	11	6.66E-01	1072	1.04E-01	429	2.72E-01	594	8.19E-06	169	4	2.68E-02	214	1.34E-02	396	3.25E-02	21	1.16E-02	810	5.43E-07	96	2.98E-01	681	1.82E-03	25	6	10	441.00
10951	NM_001127228	CBX1	1.62E-02	223	4.10E-02	426	2.06E-01	311	4.52E-01	855	3.67E-01	485	6.75E-01	1075	7.49E-02	362	6.22E-02	297	1.38E-02	558	3	6.45E-01	904	2.90E-03	263	2.69E-02	19	1.21E-07	100	1.02E-04	306	2.06E-01	603	4.34E-02	289	5	8	442.25
23129	NM_015103	Plxnd1	1.09E-02	176	1.62E-01	596	7.59E-01	909	9.16E-04	169	7.01E-02	130	4.50E-08	104	3.50E-01	762	4.91E-01	777	1.13E-01	758	3	3.33E-02	235	4.82E-01	1001	4.75E-01	577	3.46E-06	279	1.06E-06	119	5.56E-02	394	8.36E-03	97	4	7	442.69
26271	NM_001142522	Fbxo5	7.64E-01	954	1.07E-01	550	2.59E-02	99	8.14E-02	561	1.01E-02	22	4.85E-06	203	1.57E-01	511	9.39E-01	1103	6.08E-06	163	4	5.69E-02	297	6.06E-04	156	5.57E-01	677	1.40E-04	542	6.52E-05	286	9.67E-02	471	1.25E-01	493	3	7	443.00
6426	NM_003017	SFRS3	1.87E-01	597	7.79E-03	142	2.18E-01	325	3.27E-02	443	3.56E-01	471	1.70E-05	244	3.53E-03	61	2.22E-01	549	1.39E-04	268	5	1.12E-01	401	4.32E-01	980	4.39E-01	528	1.94E-07	116	1.12E-02	609	9.77E-02	473	5.78E-01	892	2	7	443.69
10576	NM_006431	CCT2	3.42E-02	365	1.32E-02	198	4.58E-01	560	1.01E-01	591	8.41E-01	994	5.83E-04	395	7.30E-02	352	2.61E-01	584	7.11E-05	238	4	2.64E-02	212	4.05E-01	971	3.73E-01	415	9.78E-08	89	7.15E-05	292	5.46E-02	391	1.07E-01	459	3	7	444.13
10908	NM_001166111	PNPLA6	3.51E-02	372	4.81E-01	815	7.96E-01	951	3.32E-04	108	6.19E-01	772	8.99E-09	75	4.69E-01	860	2.12E-02	166	1.27E-02	553	5	5.51E-04	33	1.92E-05	30	5.24E-01	641	7.81E-04	656	4.38E-05	259	7.08E-03	178	2.40E-01	641	5	10	444.38
11052	NM_007007	Cpsf6	3.24E-01	717	9.75E-01	1141	4.98E-01	608	1.11E-01	603	2.45E-02	45	4.98E-09	65	6.69E-01	984	2.94E-01	614	2.84E-05	202	3	3.07E-04	20	4.41E-03	302	3.66E-01	405	2.24E-07	122	1.40E-02	627	5.03E-02	383	4.26E-02	285	5	8	445.19
23291	NM_033644	Fbxw11	4.98E-02	472	6.09E-01	887	3.86E-01	485	4.12E-03	248	2.47E-01	342	5.55E-04	393	5.73E-02	304	1.23E-01	404	4.02E-03	455	4	7.67E-01	996	6.12E-03	328	2.11E-01	178	4.87E-04	630	1.95E-03	456	1.67E-01	551	3.30E-05	1	4	8	445.63
80010	NM_024959	SLC24A6	1.29E-04	21	6.08E-02	512	3.38E-01	443	2.46E-01	731	3.35E-03	10	1.68E-03	454	3.19E-01	724	3.40E-02	215	2.87E-07	92	5	4.29E-02	268	4.30E-04	140	4.93E-01	598	6.98E-01	1117	5.47E-07	97	7.82E-01	1018	3.10E-01	699	3	8	446.19
51081	NM_015971	MRPS7	8.91E-01	1054	9.04E-03	155	2.73E-01	368	8.00E-02	559	1.85E-01	252	5.17E-07	155	3.16E-01	721	3.03E-03	61	3.71E-04	303	4	1.35E-01	429	8.89E-05	72	4.52E-01	548	3.75E-05	448	1.42E-01	875	1.74E-02	251	5.77E-01	891	3	7	446.38
523	NM_001690	Atp6v1a	1.51E-03	62	6.08E-01	886	1.34E-01	234	2.67E-02	414	6.15E-01	766	5.49E-01	1035	1.60E-01	519	2.77E-03	59	4.00E-03	454	4	7.00E-02	325	1.77E-02	425	9.47E-02	47	2.40E-04	581	4.94E-03	535	1.47E-02	226	1.77E-01	575	4	8	446.44
4E+05	NM_172089	Tnfsf12-	9.85E-01	1140	5.75E-03	113	4.37E-01	537	1.52E-01	653	4.14E-01	536	9.52E-06	224	7.73E-02	372	4.40E-02	254	6.41E-01	1033	3	4.77E-01	796	3.53E-02	515	2.13E-01	180	1.61E-06	235	7.25E-09	30	5.90E-04	84	1.02E-01	448	4	7	446.88
6781	NM_003157	NEK4	8.70E-01	1033	8.28E-04	38	5.79E-02	171	8.69E-01	1084	2.31E-01	314	7.85E-13	19	1.40E-01	487	3.03E-03	62	2.02E-02	578	4	4.69E-06	3	3.47E-01	925	3.05E-01	301	6.08E-01	1095	3.11E-15	1	3.87E-01	761	4.47E-02	292	3	7	447.75
7280	NM_001069	Tubb2a	4.91E-03	114	9.34E-01	1114	3.37E-02	122	7.02E-06	27	4.16E-01	539	1.04E-04	322	5.97E-01	947	2.39E-01	567	2.07E-05	193	5	6.22E-01	892	1.84E-02	432	4.78E-03	3	5.08E-06	301	2.12E-01	917	5.10E-04	79	2.05E-01	607	4	9	448.50
23518	NM_015361	R3hdm1	3.36E-02	361	5.14E-02	499	9.43E-01	1113	2.03E-02	386	3.45E-01	461	1.56E-08	85	2.99E-01	709	3.89E-02	231	3.08E-08	64	5	8.69E-01	1070	4.72E-03	309	1.27E-01	72	1.02E-05	346	4.95E-01	1024	7.08E-02	427	2.99E-03	43	3	8	450.00
3914	NM_002293	LAMC1	1.71E-02	226	9.20E-01	1107	5.84E-01	716	5.78E-06	24	2.30E-01	312	1.57E-09	55	1.10E-02	111	1.05E-03	37	2.22E-16	1	6	7.40E-01	971	3.26E-01	909	2.63E-01	237	9.01E-03	790	3.47E-08	50	7.82E-01	1017	2.92E-01	691	2	8	453.38
1E+05	NM_182919	TICAM1	8.76E-01	1041	2.00E-03	64	2.97E-01	400	2.02E-02	384	5.49E-01	684	3.89E-04	382	7.11E-03	92	2.49E-02	183	9.46E-01	1136	5	1.35E-02	147	4.02E-03	296	1.76E-01	129	1.51E-05	376	1.71E-05	215	1.97E-01	590	9.63E-01	1138	4	9	453.56
10456	NM_006118	HAX1	7.41E-01	937	4.34E-01	787	4.77E-02	155	7.91E-04	156	2.48E-01	344	2.39E-07	137	8.74E-01	1087	3.98E-01	715	5.31E-02	679	3	2.72E-01	591	1.15E-01	662	1.62E-01	112	4.02E-07	151	9.23E-07	114	1.32E-01	518	1.11E-02	116	3	6	453.81
10153	NM_005760	Cebpz	7.07E-02	500	3.03E-02	349	6.66E-01	813	3.68E-03	242	5.95E-02	114	9.27E-05	318	4.42E-01	840	6.29E-03	87	1.15E-03	377	5	3.26E-01	654	8.19E-01	1113	3.90E-01	451	5.59E-07	165	7.92E-02	814	3.69E-02	337	7.86E-03	89	3	8	453.94
23171	NM_015141	GPD1L	9.16E-01	1065	1.74E-02	235	9.40E-01	1109	1.06E-03	176	5.54E-01	691	7.37E-14	13	1.05E-03	26	5.23E-01	797	3.59E-02	639	5	1.71E-01	476	8.16E-02	616	3.60E-01	393	1.22E-06	220	4.87E-06	171	5.56E-02	393	3.31E-02	246	3	8	454.13
10301	NT_024524	DLEU1	4.98E-01	816	4.81E-03	101	9.42E-02	202	4.36E-01	846	5.31E-02	106	3.60E-02	677	2.45E-01	648	5.58E-01	829	4.47E-03	468	3	3.70E-02	252	2.83E-03	258	3.15E-01	316	2.84E-05	429	9.52E-03	595	2.69E-01	658	6.38E-03	68	5	8	454.31
6676	NM_003119	SPG7	8.14E-01	996	1.91E-03	60	6.11E-03	45	9.52E-01	1132	2.58E-01	355	1.02E-01	793	8.93E-01	1095	4.39E-02	253	6.99E-01	1048	3	1.44E-02	151	3.33E-03	273	3.83E-01	433	4.00E-09	16	5.67E-05	280	2.65E-02	298	4.14E-03	52	6	9	455.00
833	NM_139273	carS	2.29E-01	631	5.02E-01	827	5.21E-03	39	2.60E-05	53	5.65E-01	706	3.17E-04	372	8.11E-02	383	3.95E-02	237	8.29E-02	726	4	5.01E-03	102	7.59E-02	602	3.80E-01	428	1.69E-04	552	1.36E-07	66	9.22E-01	1102	1.16E-01	470	3	7	456.00
5993	NM_020216	Rnpep	2.13E-02	272	3.12E-01	709	6.90E-01	845	2.27E-02	399	3.12E-01	421	3.35E-07	145	5.57E-03	82	1.50E-04	19	1.67E-01	801	5	3.37E-01	667	9.01E-03	366	3.36E-01	350	3.20E-05	438	8.06E-01	1107	1.37E-01	521	1.75E-02	156	3	8	456.13
694	NM_001731	btg1	2.19E-02	281	3.19E-01	716	1.12E-01	214	3.71E-02	463	4.88E-01	629	1.12E-06	173	4.04E-01	815	1.20E-02	122	2.84E-01	872	4	2.93E-02	221	2.97E-04	113	7.31E-01	856	7.41E-07	186	1.01E-01	834	2.45E-01	635	2.11E-02	178	4	8	456.75
5621	NM_002765	prps2	1.21E-02	190	2.93E-01	699	8.95E-01	1066	7.70E-02	553	1.30E-01	195	1.08E-04	324	2.11E-01	600	4.53E-03	75	4.44E-01	956	3	6.81E-01	922	2.19E-01	780	1.09E-01	58	5.77E-07	168	2.72E-10	14	1.19E-05	22	3.15E-01	705	3	6	457.94
7357	NM_003358	UGCG	2.17E-01	618	1.20E-03	45	6.68E-01	817	2.79E-03	225	6.79E-01	839	1.69E-01	850	3.95E-04	14	8.48E-01	1031	9.06E-03	520	4	2.42E-02	204	3.92E-05	42	3.02E-01	296	7.70E-07	191	3.22E-05	241	1.78E-01	570	4.87E-01	830	4	8	458.31
8553	NM_003670	bhlhe40	4.16E-05	14	1.39E-11	1	1.38E-01	239	6.32E-01	961	4.74E-02	97	5.80E-01	1044	7.44E-01	1020	1.58E-02	144	3.35E-01	902	4	2.33E-04	13	4.46E-05	46	2.97E-01	285	7.82E-08	78	2.71E-02	703	3.73E-01	750	8.32E-01	1042	4	8	458.69
55312	NM_018346	RSAD1	9.01E-02	525	3.03E-02	348	3.41E-01	446	2.13E-01	701	4.26E-01	553	6.73E-06	213	1.25E-01	462	9.13E-02	351	1.68E-07	83	3	7.18E-02	332	8.29E-04	174	4.25E-01	501	6.68E-05	493	4.56E-01	1008	1.73E-02	249	5.95E-01	906	3	6	459.06
4282	NM_001170931	FOXO4	1.29E-04	20	1.87E-02	247	6.51E-01	792	4.98E-01	881	2.98E-01	405	8.71E-01	1126	4.43E-01	841	1.12E-01	387	4.99E-04	320	3	4.38E-01	763	4.38E-05	44	2.43E-01	208	3.82E-06	287	8.56E-03	585	2.30E-02	281	1.79E-02	160	5	8	459.19
10313	NM_201428	RTN3	7.33E-03	145	7.72E-01	1005	2.96E-01	398	1.13E-01	606	2.95E-01	401	6.97E-08	111	2.05E-01	594	1.47E-02	136	8.86E-03	517	4	1.34E-01	427	2.63E-03	252	9.10E-01	1040	7.35E-04	655	2.12E-05	219	9.75E-03	196	2.51E-01	650	4	8	459.50
5245	NM_000293	Phkb	4.28E-01	775	4.72E-02	475	4.01E-01	502	3.47E-03	236	9.89E-01	1143	5.90E-08	108	5.23E-01	900	1.28E-02	126	2.62E-04	292	5	1.06E-02	135	7.05E-01	1079	2.77E-01	255	8.96E-07	198	2.39E-04	343	1.99E-02	267	1.47E-01	522	4	9	459.75
9	NM_001160175	NAT1	3.94E-02	404	6.09E-03	117	5.55E-01	680	3.40E-01	794	5.77E-02	110	3.04E-04	368	1.49E-01	497	1.64E-01	471	1.45E-01	783	3	2.73E-01	593	2.63E-01	833	2.00E-01	157	1.15E-04	526	3.68E-01	970	2.91E-05	29	2.27E-03	31	3	6	460.19
51031	NM_016080	GLOD4	4.11E-02	416	4.24E-01	780	4.12E-02	139	3.71E-02	464	3.59E-01	477	1.78E-02	620	8.94E-01	1096	5.38E-03	82	1.16E-01	762	5	4.02E-02	259	6.32E-04	160	3.18E-01	319	2.59E-03	714	7.45E-05	293	8.39E-02	448	5.48E-02	343	4	9	460.75
6617	NM_001039697	Snapc3	1.02E-01	531	2.79E-03	70	5.26E-01	646	1.95E-01	687	7.86E-01	945	5.32E-04	388	2.90E-02	198	5.17E-01	793	1.24E-02	549	4	2.99E-04	19	9.64E-04	182	1.32E-01	77	2.61E-03	715	2.93E-01	948	2.21E-01	617	4.26E-04	9	4	8	460.88
961	NM_198793	CD47	4.70E-02	454	6.92E-01	950	8.64E-01	1034	7.96E-04	158	3.25E-01	436	6.63E-06	212	9.56E-02	411	2.44E-02	180	8.14E-04	345	5	2.35E-01	546	1.16E-02	387	7.46E-01	873	1.95E-07	117	1.41E-05	208	3.23E-03	135	6.49E-01	934	4	9	461.25
123	NM_001122	PLIN2	2.57E-04	30	5.24E-02	503	5.98E-01	736	7.80E-09	1	2.11E-01	281	3.00E-12	24	2.74E-01	687	1.00E-02	111	9.22E-01	1122	4	9.64E-03	126	3.13E-01	898	5.80E-01	704	2.40E-07	124	6.14E-02	796	9.34E-01	1111	1.48E-02	141	3	7	462.19
6787	NM_198437	AURKA	1.60E-01	580	8.39E-03	147	1.15E-01	218	3.27E-01	786	2.70E-01	370	6.48E-04	402	2.13E-01	604	3.45E-01	659	2.37E-06	130	3	1.74E-01	480	2.83E-03	259	2.07E-01	169	1.20E-05	361	6.82E-03	563	4.38E-01	799	5.68E-01	886	3	6	463.31
9709	NM_001010990	HERPUD1	1.58E-02	221	2.34E-01	655	7.12E-01	862	2.18E-02	394	6.61E-01	822	4.21E-01	992	3.34E-01	737	1.39E-01	433	3.41E-03	441	3	1.22E-04	11	1.13E-02	385	4.12E-01	481	1.86E-06	242	5.25E-04	377	2.80E-03	130	3.02E-02	231	6	9	463.38
501	NM_001182	ALDH7A1	9.32E-01	1083	1.61E-01	594	5.81E-04	13	2.85E-05	55	6.30E-01	789	1.53E-05	236	1.19E-01	453	6.22E-01	875	1.11E-01	755	3	6.12E-02	309	1.35E-01	692	1.51E-01	101	7.59E-05	500	5.57E-10	18	8.06E-03	184	3.81E-01	758	3	6	463.44
6611	NM_005985	snai1	2.14E-01	616	1.22E-01	565	8.70E-03	55	1.57E-01	657	1.45E-01	209	1.21E-10	34	9.01E-01	1104	1.32E-01	420	1.72E-10	19	3	6.96E-01	937	3.89E-04	133	5.38E-01	654	2.76E-05	426	4.32E-02	755	2.78E-01	670	1.88E-02	165	4	7	463.69
7436	NM_001018056	vldlr	2.72E-02	323	4.49E-02	457	7.22E-02	184	5.91E-01	941	2.01E-01	266	1.33E-03	442	1.49E-01	496	2.32E-02	176	1.51E-02	567	5	1.05E-02	134	2.91E-01	874	2.78E-01	258	1.23E-07	102	2.13E-01	918	2.63E-02	297	7.30E-01	985	3	8	463.75
8780	NM_003831	Riok3	1.84E-01	595	4.20E-02	434	1.94E-01	297	4.81E-02	501	2.85E-01	387	5.28E-04	387	1.22E-01	456	2.16E-02	167	4.03E-02	653	5	5.05E-02	285	5.22E-05	51	8.79E-01	1010	5.00E-06	300	1.88E-01	902	5.83E-01	895	9.32E-03	104	3	8	464.00
5660	NM_002791	Psma6	2.35E-02	294	4.27E-01	782	2.60E-01	361	1.44E-03	192	1.55E-01	222	6.67E-05	302	1.82E-01	554	9.60E-01	1122	4.74E-02	670	4	6.81E-01	923	7.29E-04	167	1.78E-01	134	1.03E-06	207	1.76E-06	131	1.76E-01	567	4.35E-01	797	3	7	464.06
328	NM_080649	Apex1	7.31E-01	926	1.78E-02	238	4.09E-01	506	9.74E-03	329	8.14E-01	971	3.30E-04	374	1.17E-01	450	2.00E-02	158	1.63E-03	396	5	4.51E-02	274	6.04E-03	326	4.45E-01	534	1.95E-06	249	2.89E-01	945	1.28E-01	512	3.26E-02	242	4	9	464.38
2317	NM_002028	Fntb	1.53E-02	216	3.31E-02	370	6.82E-01	836	3.07E-03	230	2.02E-01	269	2.95E-05	269	1.56E-03	36	3.52E-03	68	3.96E-08	67	7	1.04E-01	388	2.22E-03	238	8.18E-01	954	4.53E-01	1061	4.64E-01	1014	9.47E-01	1124	4.75E-02	303	2	9	465.19
10963	NM_006819	stip1	7.82E-01	965	3.00E-02	345	8.58E-01	1026	9.15E-02	579	2.66E-01	367	3.86E-01	975	1.27E-02	118	1.05E-01	376	1.44E-04	269	3	6.11E-04	35	9.92E-04	186	3.01E-01	293	7.46E-02	939	4.72E-08	54	1.21E-01	505	8.90E-02	422	3	6	465.88
23212	NM_015169	rrs1	4.28E-01	774	8.89E-04	41	6.21E-02	175	4.41E-01	851	2.31E-01	315	1.81E-05	248	1.85E-01	559	4.08E-02	244	9.79E-01	1151	3	3.92E-02	258	3.58E-01	930	3.84E-01	437	2.63E-10	4	1.32E-01	870	1.42E-01	524	7.54E-03	82	3	6	466.44
6856	NM_003184	Taf2	6.76E-04	39	5.85E-01	869	5.20E-01	640	4.53E-05	63	7.47E-03	18	2.29E-01	885	3.01E-01	711	8.21E-01	1021	7.36E-06	166	4	5.10E-02	287	7.34E-02	597	8.39E-01	969	1.51E-05	375	1.07E-05	200	3.17E-02	315	4.93E-02	312	4	8	466.69
11163	NM_199040	NUDT4	4.00E-02	406	6.84E-04	34	1.30E-01	232	2.79E-01	757	4.01E-01	520	2.90E-03	489	8.58E-01	1083	1.90E-01	516	3.27E-06	140	4	9.30E-02	370	3.66E-01	939	1.54E-01	103	1.34E-06	228	1.52E-04	326	3.05E-01	686	2.44E-01	643	2	6	467.00
5937	NM_002902	RCN2	1.03E-01	536	1.95E-02	254	2.05E-01	310	1.76E-04	83	1.81E-01	247	2.23E-06	187	8.56E-02	392	3.80E-01	694	8.61E-01	1100	3	5.44E-01	846	8.91E-02	628	1.35E-01	82	3.12E-03	724	5.37E-05	275	2.07E-02	269	5.41E-01	865	3	6	468.25
1476	NM_001325	cstf2	1.80E-02	235	7.38E-01	980	4.72E-01	579	3.02E-03	229	4.20E-03	12	2.27E-02	634	9.65E-01	1141	5.76E-02	285	1.24E-04	260	5	1.10E-01	396	6.63E-02	582	6.79E-01	808	3.83E-07	148	2.23E-03	469	1.72E-02	248	1.23E-01	490	3	8	468.50
230	NM_005165	aldoc	7.73E-04	41	4.11E-02	427	8.81E-02	197	9.21E-01	1117	6.41E-01	801	3.01E-01	927	6.21E-01	959	3.88E-02	230	5.99E-02	693	3	3.15E-03	78	1.38E-04	88	2.80E-01	259	2.58E-11	1	1.79E-02	656	1.67E-02	244	4.17E-01	783	5	8	468.81
384	NM_001172	arg2	3.02E-01	705	3.83E-02	407	5.42E-01	667	4.07E-04	122	1.13E-01	182	2.30E-10	40	7.35E-02	354	4.14E-03	71	2.66E-01	860	4	9.51E-01	1123	2.27E-01	792	2.65E-01	239	5.11E-04	636	1.16E-01	854	8.91E-03	189	3.65E-02	260	3	7	468.81

8503	NM_001114172	PIK3R3	9.21E-01	1069	4.37E-02	450	1.08E-01	212	2.11E-01	698	1.51E-01	217	1.76E-07	127	1.13E-04	8	5.51E-01	823	4.25E-01	946	3	9.24E-01	1103	1.72E-02	422	2.41E-02	16	3.78E-08	44	8.10E-07	105	7.85E-01	1020	3.56E-02	255	5	8	469.69
54962	NM_017867	C4orf27	9.69E-01	1126	9.75E-03	162	1.63E-01	264	1.31E-01	630	3.50E-04	2	3.91E-06	197	2.47E-01	652	1.65E-01	473	3.70E-03	449	4	2.32E-01	542	2.48E-02	469	3.05E-01	300	5.28E-06	305	1.44E-03	449	2.87E-01	675	4.85E-01	828	3	7	470.19
64112	NM_022356	lepre1	4.90E-03	113	6.82E-01	938	4.41E-01	544	4.78E-05	64	2.93E-01	399	1.29E-01	819	2.77E-01	690	4.66E-02	261	2.80E-01	869	3	9.90E-03	129	2.22E-02	455	1.66E-01	123	6.35E-01	1102	4.54E-05	264	5.40E-05	36	3.43E-01	726	4	7	470.75
3021	NM_005326	HAGH	9.95E-02	529	1.22E-02	191	5.33E-01	657	1.73E-03	195	5.43E-01	680	2.54E-06	190	1.73E-03	41	2.40E-03	56	3.08E-03	436	6	8.10E-01	1023	2.37E-02	460	9.39E-01	1083	2.69E-05	424	7.13E-02	805	9.84E-06	20	3.75E-01	751	3	9	471.31
9522	NM_004866	SCAMP1	2.30E-01	633	4.34E-01	785	2.89E-01	389	8.39E-02	566	9.58E-02	157	1.45E-04	335	1.57E-02	137	3.33E-01	649	4.07E-04	308	3	7.53E-01	983	1.03E-01	647	6.76E-01	806	3.01E-05	434	3.26E-06	157	1.55E-01	536	1.31E-03	21	3	6	471.44
4172	NM_005915	MCM6	2.64E-01	659	3.13E-02	357	5.30E-01	651	2.18E-03	201	4.39E-01	573	3.03E-02	662	1.27E-01	464	6.12E-01	866	6.17E-05	234	4	2.42E-02	203	9.15E-04	179	3.89E-01	448	2.59E-09	12	1.04E-03	428	6.50E-01	937	2.87E-01	686	4	8	472.50
8683	NM_003769	SFRS9	4.39E-03	106	2.41E-01	660	1.02E-01	208	5.64E-04	137	2.77E-01	384	1.72E-02	616	2.77E-02	191	7.59E-01	977	2.93E-06	138	5	8.61E-02	360	8.89E-01	1138	6.60E-01	787	9.99E-05	517	7.74E-04	409	5.24E-01	849	7.95E-03	91	3	8	473.00
10561	NM_006417	IFI44	3.40E-01	726	1.18E-02	184	5.09E-01	623	5.46E-01	913	4.82E-01	623	5.79E-03	524	5.51E-02	293	2.42E-01	570	1.30E-01	772	2	6.71E-01	918	3.86E-02	527	6.70E-02	34	2.15E-07	121	1.49E-02	635	1.11E-04	53	5.23E-03	61	5	7	473.56
11319	NM_001135752	ECD	2.63E-01	658	1.17E-01	558	4.97E-03	36	3.31E-02	446	4.24E-01	551	3.69E-01	968	1.55E-03	35	2.62E-05	8	1.23E-02	548	5	5.41E-01	842	3.02E-02	501	4.89E-01	594	2.06E-02	847	2.47E-02	691	4.90E-04	77	2.81E-02	217	5	10	473.56
1719	NM_001025434	nqo1	7.94E-01	977	1.52E-01	589	2.09E-02	81	2.04E-05	47	2.90E-02	57	3.62E-04	379	5.58E-01	924	1.06E-01	377	9.79E-04	360	5	1.83E-01	495	4.44E-04	143	4.83E-01	584	2.97E-06	274	3.98E-04	364	6.55E-01	941	7.35E-01	987	3	8	473.69
10276	NM_001047160	NET1	8.08E-01	992	2.39E-01	659	2.36E-02	92	2.86E-02	422	8.96E-01	1051	0.00E+00	2	4.57E-02	260	4.36E-02	252	3.73E-06	145	6	2.87E-01	612	1.19E-01	669	1.03E-01	54	9.22E-08	86	3.38E-02	726	5.07E-01	841	3.33E-01	718	2	8	473.81
6434	NM_006930	SKP1	9.40E-01	1090	4.68E-02	472	8.71E-02	196	3.92E-02	475	3.23E-03	9	8.46E-09	73	2.69E-01	683	2.08E-01	530	2.12E-04	281	5	3.14E-01	642	1.00E-02	374	4.86E-01	587	8.94E-08	83	1.06E-01	836	2.72E-02	301	6.80E-01	954	3	8	474.13
55299	NM_018339	RFK	3.44E-01	728	3.93E-02	416	3.55E-01	460	8.55E-04	164	9.04E-01	1055	7.81E-06	216	1.07E-01	433	1.55E-02	139	4.54E-04	315	5	5.84E-01	870	3.79E-01	950	7.72E-01	905	2.33E-08	36	9.84E-05	305	1.70E-01	557	2.69E-03	37	3	8	474.13
26092	NM_015602	TOR1AIP1	2.75E-03	85	7.44E-01	988	6.69E-01	818	3.43E-02	449	3.52E-01	468	1.39E-08	83	3.13E-01	720	1.42E-01	439	8.38E-04	348	4	4.30E-01	755	8.79E-03	364	8.06E-01	941	2.16E-06	254	5.00E-05	270	1.02E-01	478	1.33E-02	130	4	8	474.38
4200	NM_005587	MEF2A	2.18E-01	621	4.66E-01	807	7.71E-01	921	7.24E-04	150	8.66E-01	1019	1.90E-08	89	4.07E-02	241	4.50E-01	748	3.05E-11	12	4	9.32E-01	1111	3.35E-05	37	3.47E-01	374	2.02E-05	399	2.60E-03	482	6.99E-03	177	8.14E-02	403	4	8	474.44
51075	NM_015959	TMX2	9.23E-01	1072	2.79E-02	326	2.37E-01	341	2.37E-03	207	6.48E-01	807	1.01E-01	790	3.65E-02	224	5.40E-01	809	3.68E-02	641	4	2.33E-02	195	8.05E-07	7	4.35E-01	521	1.97E-05	396	6.46E-05	284	1.29E-02	208	4.00E-01	771	5	9	474.94
56683	NM_020154	C15orf24	1.46E-01	573	1.85E-02	246	1.86E-01	289	6.18E-01	952	3.35E-01	450	4.74E-05	283	5.18E-02	279	7.83E-03	99	1.64E-01	797	3	2.24E-01	532	7.68E-04	171	2.85E-01	266	6.62E-08	69	1.84E-02	658	9.15E-01	1101	5.04E-01	839	3	6	475.25
51622	NM_198097	C7orf28A	1.30E-02	200	4.54E-01	801	7.33E-01	885	1.28E-02	349	2.49E-01	345	3.34E-08	95	7.44E-01	1019	8.19E-03	101	1.01E-02	529	5	8.33E-03	122	5.50E-03	321	4.63E-01	567	1.88E-08	32	3.66E-03	505	6.70E-01	951	4.19E-01	784	4	9	475.38
5218	NM_002631	PGD	5.71E-03	128	4.53E-01	800	5.94E-01	730	3.75E-01	817	8.84E-01	1035	2.12E-11	28	5.90E-03	85	5.75E-01	837	2.88E-03	430	4	6.08E-01	884	3.41E-06	11	6.57E-03	5	7.29E-01	1124	3.51E-05	246	1.19E-02	205	3.29E-02	244	5	9	475.56
55901	NM_001006600	Erbb2ip	8.42E-01	1015	2.83E-04	20	6.50E-02	178	5.13E-01	891	6.31E-02	122	3.11E-03	494	7.96E-02	380	4.28E-03	72	3.96E-01	929	3	1.87E-01	500	3.76E-01	946	1.49E-01	99	8.42E-05	508	3.69E-03	507	4.62E-01	819	1.42E-02	138	3	6	476.13
79009	NM_001143843	TMEM106C	3.72E-02	392	8.20E-02	530	1.71E-01	277	4.29E-06	19	6.87E-01	850	1.26E-01	817	1.07E-03	27	1.56E-02	140	1.06E-02	535	5	2.43E-02	205	8.51E-01	1120	3.98E-01	461	6.07E-03	759	6.48E-03	559	4.27E-01	787	1.46E-02	140	4	9	476.13
10714	NM_006591	pold3	6.25E-01	869	3.07E-02	354	3.05E-01	411	3.51E-04	112	4.23E-02	85	2.02E-03	462	2.42E-01	642	7.06E-02	315	3.74E-02	644	5	4.15E-01	744	3.84E-04	131	2.55E-01	219	1.43E-02	825	7.27E-03	570	1.20E-01	502	3.57E-01	737	3	8	476.38
3628	NM_005539	INPP5A	9.53E-01	1104	2.06E-02	261	4.07E-01	505	7.91E-02	557	2.87E-01	392	1.91E-01	864	1.73E-02	145	1.66E-01	475	1.01E-01	746	2	1.15E-01	407	2.62E-01	832	3.44E-02	23	5.49E-05	479	2.04E-06	139	1.01E-04	49	2.45E-01	645	4	6	476.44
3241	NM_000194	HPRT1	5.46E-02	477	1.25E-01	566	9.96E-01	1157	3.78E-04	116	6.57E-01	816	2.12E-07	134	4.29E-01	830	1.96E-03	49	4.37E-01	951	3	3.32E-01	661	9.12E-05	74	3.52E-01	377	9.22E-05	513	2.22E-06	141	5.01E-02	382	7.17E-02	384	3	6	476.75
5984	NM_007370	RFC5	3.74E-01	746	2.31E-01	652	2.51E-02	97	6.65E-03	291	2.26E-03	7	1.70E-01	851	7.44E-02	360	8.07E-01	1011	1.72E-09	32	4	4.83E-03	100	6.41E-02	578	5.55E-01	675	2.27E-05	411	7.73E-08	61	9.39E-01	1113	2.50E-01	649	3	7	477.13
11266	NM_007240	DUSP12	2.91E-02	331	8.58E-01	1067	7.27E-01	879	3.59E-02	457	1.23E-01	189	9.51E-03	557	2.36E-01	634	5.66E-01	833	5.92E-04	324	4	1.23E-01	412	1.69E-03	224	3.42E-01	360	2.89E-06	268	2.12E-03	461	1.64E-01	546	8.20E-03	94	4	8	477.25
834	NM_033292	CASP1	8.77E-02	520	6.43E-03	120	5.47E-03	41	5.14E-01	894	5.54E-01	693	6.70E-02	739	3.44E-01	750	1.29E-01	413	1.50E-01	787	2	6.96E-01	935	2.21E-02	453	4.10E-01	475	2.94E-06	272	9.71E-09	34	3.55E-02	332	2.17E-02	180	5	7	477.38
5198	NM_012394	PFDN2	8.53E-01	1019	1.80E-03	59	6.49E-01	788	5.51E-06	23	4.91E-01	633	1.45E-02	602	7.35E-02	355	2.53E-03	57	9.12E-04	358	5	3.39E-03	82	2.02E-01	761	3.47E-01	373	1.92E-07	115	6.87E-01	1076	8.81E-01	1076	3.66E-02	261	3	8	477.38
7804	NM_001018054	LRP8	1.89E-02	245	3.65E-02	393	5.55E-01	679	2.12E-02	389	4.30E-02	87	2.36E-11	29	4.93E-02	267	1.06E-02	114	9.37E-01	1131	7	1.72E-01	477	8.58E-01	1122	7.10E-01	839	3.39E-03	731	6.02E-06	176	4.79E-01	828	1.34E-02	133	3	10	477.50
26973	NM_001144073	CHORDC1	6.66E-01	892	7.85E-02	528	4.25E-02	145	2.31E-01	717	8.21E-01	977	2.80E-10	44	3.13E-02	209	7.65E-01	984	1.71E-07	85	4	1.85E-01	498	1.14E-05	22	5.50E-01	669	1.08E-07	94	3.53E-04	358	8.34E-01	1044	7.05E-02	381	3	7	477.94
4758	NM_181828	Nf2	3.10E-02	346	6.84E-01	941	2.86E-01	385	4.59E-01	859	3.70E-01	490	2.81E-01	922	4.83E-01	867	1.53E-01	458	1.60E-05	183	2	3.70E-01	705	1.95E-02	438	3.79E-01	425	2.03E-06	251	1.51E-07	70	1.37E-02	218	8.18E-03	93	5	7	478.19
4076	NM_001135937	SMAD2	1.03E-01	534	1.83E-01	623	9.02E-01	1073	4.59E-03	257	2.32E-01	316	1.60E-02	611	5.15E-02	277	9.44E-02	357	1.47E-08	54	3	3.64E-01	696	7.70E-04	172	6.01E-01	727	1.01E-06	205	2.07E-03	458	3.98E-01	770	1.52E-01	528	3	6	478.63
5694	NM_002805	PSMC5	4.21E-02	425	1.45E-01	582	4.33E-01	533	2.51E-04	98	2.88E-01	396	8.87E-01	1130	2.08E-01	596	4.49E-03	74	4.66E-02	668	4	2.35E-03	67	1.46E-03	214	2.73E-01	250	3.13E-05	437	7.88E-03	578	3.56E-01	733	5.58E-01	877	4	8	478.63
226	NM_001127617	ALDOA	3.13E-02	349	1.32E-03	50	7.48E-01	897	2.58E-01	738	5.58E-01	697	4.98E-02	711	5.80E-01	937	1.74E-01	488	5.86E-01	1013	3	2.39E-03	68	4.56E-07	5	9.38E-02	45	1.83E-07	113	1.47E-04	324	1.32E-02	212	7.77E-01	1013	5	8	478.75
6385	NM_001007067	sdcbp	7.29E-01	923	3.97E-03	89	6.83E-01	837	2.20E-04	92	2.14E-01	286	1.77E-07	128	6.16E-02	322	2.40E-01	568	1.48E-03	390	4	7.23E-01	956	1.24E-01	675	3.25E-01	331	3.28E-07	138	7.14E-01	1081	1.82E-02	256	1.88E-01	589	2	6	478.81
9686	NM_014667	VGLL4	3.04E-03	90	5.86E-01	871	6.28E-01	763	1.06E-02	336	6.83E-02	129	1.32E-02	590	5.86E-02	309	1.60E-01	465	1.59E-01	793	3	1.52E-01	449	7.74E-03	347	2.52E-01	213	1.40E-03	690	4.80E-05	267	2.61E-02	296	8.42E-01	1053	4	7	478.81
5496	NM_002716	ppp2r1b	4.47E-02	441	5.61E-02	509	3.90E-01	487	6.26E-01	957	1.42E-01	204	8.86E-03	552	2.64E-01	674	1.39E-01	431	6.92E-02	706	2	3.46E-02	239	4.06E-04	135	5.64E-01	688	7.46E-08	74	4.96E-05	269	9.27E-01	1105	2.40E-02	195	5	7	479.13
10949	NM_006805	HNRNPA0	1.12E-02	182	5.81E-01	867	5.61E-01	688	5.13E-02	516	1.19E-01	187	1.07E-01	797	9.12E-01	1113	8.01E-01	1004	1.14E-03	375	2	1.46E-02	153	5.53E-04	152	4.59E-01	559	2.06E-07	118	8.78E-02	822	7.43E-04	89	4.01E-03	50	5	7	479.50
204	NM_001625	Ak2	2.75E-03	84	1.06E-01	547	4.39E-01	540	3.26E-02	441	1.56E-01	223	7.84E-04	416	4.54E-01	851	2.11E-01	536	4.94E-02	672	4	1.85E-01	497	2.69E-05	34	8.22E-01	958	3.35E-07	140	2.84E-01	944	2.13E-01	608	2.26E-02	191	3	7	480.13
56925	NM_020170	ncln	1.64E-03	65	8.60E-02	534	9.72E-03	58	6.91E-01	990	6.04E-01	754	1.01E-01	789	3.82E-01	793	1.34E-02	128	9.66E-05	249	4	1.59E-01	462	2.06E-02	444	9.70E-01	1131	1.03E-06	209	2.24E-06	143	3.73E-04	74	5.30E-01	859	4	8	480.13
2288	NM_001164318	FLNB	3.37E-02	362	1.69E-01	604	5.70E-01	698	8.16E-05	74	2.43E-01	337	5.67E-01	1042	7.91E-01	1051	9.70E-02	361	1.41E-02	561	3	1.89E-02	178	1.06E-03	189	3.78E-01	423	9.77E-03	796	2.21E-03	467	1.11E-01	488	4.53E-03	54	5	8	480.31
6256	NM_002966	S100A10	1.14E-03	49	3.14E-01	712	3.44E-01	450	7.94E-01	1047	3.57E-01	472	2.97E-07	140	5.62E-01	926	7.59E-03	96	1.34E-12	5	4	6.77E-01	921	9.05E-06	20	1.30E-01	76	1.11E-03	677	8.93E-01	1126	8.20E-02	445	1.55E-01	537	2	6	481.19
54529	NM_019048	Asnsd1	3.48E-01	730	1.67E-02	232	8.34E-01	996	5.23E-02	518	1.89E-01	257	1.65E-01	848	6.18E-01	957	9.34E-04	36	3.54E-05	210	3	4.03E-01	735	1.94E-02	437	7.04E-01	834	9.47E-08	88	8.55E-07	109	5.14E-02	386	5.29E-02	333	3	6	481.63
8560	NM_003676	DEGS1	1.76E-03	68	4.40E-01	791	5.33E-01	656	1.53E-02	362	3.53E-01	470	7.43E-01	1096	1.89E-03	42	4.84E-01	771	3.52E-02	636	4	5.74E-03	107	1.02E-02	376	9.48E-02	48	7.83E-06	331	8.13E-04	413	4.75E-01	823	3.34E-01	719	4	8	481.81
54665	NM_018427	RRN3	6.49E-02	489	2.24E-01	645	2.05E-02	77	4.72E-04	132	2.02E-01	268	3.22E-01	938	3.18E-01	723	7.04E-02	314	1.37E-02	557	3	9.13E-02	367	8.37E-02	618	3.52E-01	378	6.10E-06	314	4.22E-01	993	4.59E-01	815	8.77E-03	102	2	5	483.13
55647	NM_017925	DENND4C	1.58E-02	220	2.80E-01	689	1.45E-01	243	1.82E-01	681	3.06E-02	61	2.54E-01	903	2.57E-01	666	2.18E-01	545	3.37E-05	208	3	1.23E-01	411	1.54E-03	218	7.85E-01	920	7.00E-07	181	5.54E-02	786	1.75E-04	58	6.69E-01	943	3	6	483.31
38	NM_000019	ACAT1	2.07E-02	262	8.07E-01	1028	6.28E-01	762	2.36E-01	722	6.28E-01	786	7.35E-07	161	3.19E-02	211	1.47E-01	446	2.82E-02	610	4	1.07E-01	392	2.59E-02	478	1.21E-01	69	1.84E-05	393	1.26E-06	122	6.44E-01	933	6.41E-02	367	3	7	483.88
4677	NM_152298	NASP	1.00E-01	530	2.91E-01	695	1.82E-02	73	7.23E-04	149	2.93E-02	59	1.05E-02	566	8.90E-01	1093	2.61E-01	585	3.46E-03	443	5	8.06E-02	350	1.49E-02	409	9.30E-01	1070	1.45E-07	108	1.45E-02	631	2.61E-01	654	5.25E-02	332	3	8	484.19
56929	NM_020194	MFF	7.56E-02	504	4.76E-03	100	5.47E-01	673	1.74E-01	679	9.22E-01	1069	4.00E-01	981	7.10E-02	346	1.36E-01	426	9.13E-03	522	2	1.88E-03	60	1.25E-02	390	7.63E-01	896	7.89E-07	192	1.04E-03	429	1.68E-03	112	6.65E-02	373	5	7	484.50
51388	NM_016101	NIP7	7.33E-01	928	3.17E-02	360	9.93E-01	1153	6.55E-04	145	6.28E-01	784	2.12E-05	256	4.24E-01	828	8.12E-02	334	5.59E-05	231	4	1.45E-02	152	2.27E-02	457	5.93E-01	716	4.44E-08	53	1.13E-03	433	1.01E-02	198	3.41E-01	725	5	9	484.56
27338	NM_014501	Ube2s	3.29E-02	357	2.44E-02	287	5.35E-01	662	5.95E-04	140	3.31E-01	446	9.42E-04	426	4.87E-01	871	2.07E-01	529	1.90E-06	123	5	2.99E-01	629	4.51E-02	548	8.42E-01	973	4.88E-03	748	5.20E-07	94	5.32E-02	389	1.54E-01	535	3	8	484.81
3181	NM_004500	Hnrnpc	1.12E-02	181	3.97E-02	419	8.77E-01	1045	2.76E-01	753	1.99E-01	262	5.13E-05	289	1.53E-01	505	9.03E-01	1075	5.45E-02	684	3	4.84E-02	281	2.12E-04	104	3.44E-01	364	3.52E-06	281	4.38E-02	757	1.06E-01	484	4.15E-02	279	5	8	485.19
5437	NM_002703	PPAT	5.21E-03	121	3.85E-01	757	3.35E-01	440	1.07E-02	337	5.19E-02	105	2.56E-05	264	1.73E-01	540	1.70E-03	45	4.80E-03	473	5	8.12E-01	1028	1.73E-03	227	9.37E-01	1077	1.32E-02	818	1.78E-07	73	8.97E-02	460	7.54E-01	1001	3	8	485.38
29929	NM_013339	alg6	7.72E-01	960	4.71E-03	96	4.64E-01	566	3.82E-01	818	1.82E-01	249	2.28E-02	636	1.12E-01	443	4.18E-02	247	4.63E-07	105	4	6.93E-02	323	1.74E-04	97	2.22E-01	187	1.44E-03	691	2.99E-03	495	9.50E-01	1128	3.44E-01	727	3	7	485.50
55723	NM_018238	AGK	7.83E-01	970	3.01E-03	74	5.08E-01	622	1.44E-02	360	2.76E-02	54	4.56E-03	515	7.37E-02	356	8.06E-02	332	2.06E-05	192	5	7.61E-02	342	1.53E-04	90	4.97E-01	607	1.85E-02	840	8.25E-05	297	8.98E-01	1083	8.26E-01	1037	3	8	485.69
9361	NM_004793	lonp1	1.03E-03	47	3.16E-01	714	4.23E-01	521	5.47E-03	273	2.26E-01	307	2.10E-02	631	8.06E-01	1064	4.21E-04	27	4.89E-01	978	4	7.27E-02	334	1.50E-02	410	5.46E-01	663	4.58E-05	460	3.49E-11	7	8.72E-01	1066	4.21E-02	282	4	8	486.50
10892	NM_006785	MALT1	2.00E-02	253	7.42E-01	985	4.58E-01	558	6.24E-04	141	2.77E-01	385	2.81E-12	23	4.30E-02	248	9.95E-02	368	8.03E-01	1081	4	4.79E-02	280	6.36E-01	1060	6.70E-01	797	1.03E-02	800	1.61E-06	130	2.22E-02	275	8.08E-02	401	4	8	486.56
25941	NM_015476	c18orf10	4.46E-01	783	3.90E-01	759	4.87E-01	596	5.26E-03	271	1.39E-01	201	6.52E-06	211	1.47E-02	131	4.02E-01	720	9.94E-02	743	3	3.93E-01	726	3.05E-01	888	3.36E-01	352	1.83E-04	560	3.28E-06	158	2.93E-01	679	4.33E-04	10	3	6	486.75
9318	NM_001143887	COPS2	1.82E-04	26	2.90E-01	693	8.38E-01	1002	2.68E-02	415	1.16E-01	186	4.69E-07	151	1.12E-02	112	3.49E-02	220	5.40E-02	683	5	7.07E-01	942	6.98E-02	593	6.36E-01	765	3.55E-03	734	3.72E-05	250	1.92E-03	116	5.93E-01	902	3	8	486.88
30	NM_001607	Acaa1	4.72E-02	456	3.22E-01	718	5.60E-01	686	6.10E-02	535	3.59E-01	478	8.40E-08	116	5.03E-01	882	1.79E-01	496	4.45E-05	223	3	3.44E-03	83	9.13E-02	632	1.33E-01	79	6.05E-02	923	9.63E-05	304	1.52E-01	531	2.52E-01	651	2	5	487.06
580	NM_000465	BARD1	1.83E-02	238	3.58E-02	388	1.35E-01	236	1.07E-03	177	1.18E-03	5	1.22E-04	326	3.66E-01	776	7.42E-01	963	1.63E-05	185	6	3.41E-02	238	4.12E-03	297	7.38E-01	865	8.38E-01	1138	3.32E-04	356	2.04E-01	598	7.78E-01	1014	3	9	487.50
55236	NM_018244	uqcc	4.28E-02	430	1.07E-01	549	1.06E-01	211	1.66E-01	669	7.17E-01	879	1.29E-08	81	2.50E-03	53	5.32E-01	802	2.58E-04	290	4	8.80E-01	1078	5.31E-02	560	2.91E-01	277	9.68E-02	953	6.47E-05	285	3.79E-02	338	5.57E-02	346	2	6	487.56
81887	NM_031217	kif18a	6.82E-01	900	1.56E-02	219	2.97E-01	401	2.75E-03	223	3.36E-01	452	2.35E-10	41	4.73E-01	863	3.28E-01	644	5.61E-02	687	3	8.53E-02	358	6.07E-01	1049	4.26E-01	503	5.10E-04	634	5.68E-09	28	5.11E-02	385	8.80E-02	419	2	5	487.88
9788	NM_014751	mtss1	1.00E+00	1158	1.22E-02	192	4.07E-01	504	1.98E-05	45	4.18E-01	543	1.38E-05	234	1.75E-01	542	2.72E-02	192	6.45E-02	701	4	1.34E-02	145	8.00E-01	1104	3.02E-01	295	3.79E-02	898	2.70E-06	150	4.21E-02	357	3.79E-01	754	4	8	488.38
1E+05	NM_199188	Larp4	2.78E-02	325	4.08E-02	424	4.28E-01	528	2.74E-01	752	5.41E-01	678	3.79E-02	685	1.73E-01	541	7.23E-01	954	3.01E-07	95	4	1.75E-01	484	5.82E-05	54	2.70E-01	246	3.83E-07	147	5.15E-01	1029	2.53E-02	292	1.94E-01	591	3	7	489.06
3190	NM_002149	hpcal1	2.40E-02	295	3.88E-01	758	7.49E-01	898	5.88E-02	530	4.57E-02	93	4.44E-03	513	4.86E-01	870	6.57E-02	306	1.32E-03	382	4	3.50E-02	241	6.64E-02	583	2.89E-01	274	6.08E-07	172	4.82E-01	1018	3.07E-01	688	2.65E-02	208	3	7	489.31
4354	NM_000251	Msh2	4.93E-03	115	1.30E-01	568	2.10E-01	316	1.16E-06	11	9.42E-01	1090	3.29E-02	668	3.07E-02	207	1.80E-01	498	9.32E-01	1128	4	3.44E-01	674	1.59E-04	92	2.20E-01	186	6.86E-02	932	2.77E-03	485	2.38E-04	65	4.34E-01	795	3	7	489.38
142	NM_001618	parp1	6.96E-03	142	2.16E-02	269	6.37E-01	775	5.13E-01	892	9.94E-01	1149	4.68E-01	1013	4.49E-03	74	8.76E-01	1055	5.09E-09	49	4	1.54E-01	454	6.88E-03	338	4.34E-01	519	4.65E-06	294	5.51E-04	380	2.31E-02	283	1.70E-02	149	5	9	489.69
8870	NM_003897	Ier3	4.71E-01	801	4.93E-03	103	9.62E-01	1129	4.92E-05	65	9.65E-02	158	1.14E-01	806	2.71E-01	684	1.01E-01	369	6.98E-01	1047	2	1.34E-02	144	2.55E-03	250	1.63E-01	116	4.24E-08	51	1.08E-10	10	9.42E-01	1117	7.36E-01	990	4	6	490.00
28984	NM_014059	c13orf15	3.02E-02	340	3.26E-01	722	6.58E-01	803	5.05E-02	508	3.93E-01	510	1.35E-01	827	6.07E-02	317	4.61E-01	755	7.50E-04	339	2	8.22E-02	353	4.72E-06	14	1.81E-01	140	7.80E-03	780	4.83E-02	772	1.12E-01	491	2.10E-02	176	4	6	490.44
79134	NM_024312	GNPTAB	5.25E-01	828	2.91E-03	72	5.03E-01	616	4.59E-03	256	7.79E-01	933	4.67E-05	282	1.17E-01	448	5.58E-01	830	1.48E-02	565	4	6.39E-02	316	4.96E-01	1008	3.42E-01	362	1.03E-09	8	1.79E-06	133	1.49E-02	232	6.88E-01	960	3	7	490.56
6643	NM_003104	sorD	6.08E-03	132	5.00E-01	825	7.73E-02	192	6.81E-03	294	9.91E-01	1147	4.58E-02	705	3.22E-01	730	5.41E-03	83	3.17E-05	204	5	2.79E-01	605	1.66E-02	418	1.57E-01	105	5.79E-07	169	2.90E-04	352	6.20E-01	914	7.07E-01	976	3	8	490.69
516	NM_005175	ATP5G1	9.53E-01	1106	1.61E-02	223	2.74E-01	369	2.05E-01	692	3.92E-02	77	1.15E-03	434	1.01E-01	425	7.94E-03	100	6.83E-01	1043	4	6.25E-04	38	2.91E-01	873	2.00E-01	156	2.91E-02	871	2.74E-07	81	3.64E-01	740	2.20E-01	626	3	7	490.88
2997	NM_005319	Hist1h1c	7.80E-04	42	1.47E-01	586	6.75E-03	48	6.27E-04	142	5.79E-01	724	3.60E-03	504	4.29E-01	831	3.40E-03	66	6.13E-02	695	5	5.60E-01	855	3.51E-05	38	4.56E-01	554	4.00E-01	1049	1.78E-01	898	3.17E-01	701	1.30E-02	126	2	7	491.19
10857	NM_006667	PGRMC1	1.55E-02	218	3.55E-01	739	2.17E-01	324	1.29E-03	182	4.79E-01	615	1.61E-01	840	4.69E-02	263	6.14E-02	294	8.45E-01	1096	3	1.30E-01	420	2.34E-01	802	6.16E-01	742	1.65E-05	385	7.52E-03	573	3.27E-07	5	6.21E-02	363	3	6	491.31
3842	NM_002271	IPO5	5.57E-01	839	3.28E-01	726	2.20E-03	22	2.19E-05	49	7.24E-01	884	1.56E-01	837	1.18E-01	451	7.97E-01	1003	1.13E-04	257	3	4.05E-03	90	1.80E-01	732	1.97E-01	154	5.99E-07	171	4.30E-01	998	6.55E-02	415	3.05E-02	234	3	6	491.38
3516	NM_134470	il1rap	4.15E-02	420	1.43E-01	580	4.62E-02	152	4.29E-02	487	5.50E-01	686	3.11E-01	932	2.09E-01	598	3.63E-02	225	3.35E-02	630	5	1.60E-02	158	7.60E-04	170	6.63E-01	791	2.31E-08	35	2.21E-03	468	8.28E-01	1042	1.27E-01	494	4	9	491.75
8754	NM_001005845	Adam9	2.83E-01	679	1.16E-01	554	2.07E-02	80	7.98E-01	1051	9.85E-01	1136	1.03E-06	169	4.30E-02	247	1.32E-01	421	4.29E-05	220	4	3.54E-01	687	3.18E-03	271	2.31E-02	13	6.56E-01	1110	1.38E-03	445	5.64E-02	396	7.53E-02	390	3	7	491.81
7375	NM_199443	USP4	6.47E-01	881	7.18E-03	131	4.72E-03	32	6.16E-03	284	7.87E-01	946	8.53E-01	1121	5.43E-02	288	1.15E-02	119	1.89E-08	59	5	3.56E-03	86	3.16E-05	36	5.00E-01	610	1.18E-01	962	4.20E-01	991	2.50E-01	638	2.90E-01	688	2	7	492.00

**2230**	**NM_002014**	**fkbp4**	1.05E-01	538	4.39E-02	452	6.74E-01	824	5.54E-02	523	9.51E-01	1101	6.67E-01	1073	7.98E-03	100	2.17E-02	168	1.08E-04	254	**4**	6.66E-02	319	9.24E-05	75	2.68E-01	243	8.41E-05	507	1.01E-02	599	2.59E-01	651	1.02E-01	447	**3**	**7**	**492.13**
**6632**	**NM_004175**	**snrpd3**	7.61E-01	951	1.50E-01	587	4.52E-01	552	3.06E-02	431	4.87E-02	100	1.67E-04	341	7.68E-02	370	1.39E-01	430	2.09E-05	194	**4**	1.33E-01	426	9.09E-01	1141	4.06E-01	470	1.03E-08	25	5.97E-04	387	4.31E-02	362	9.36E-01	1119	**3**	**7**	**492.88**
**5985**	**NM_001025603**	**RFX5**	6.87E-01	902	5.87E-02	510	2.01E-01	304	1.63E-02	365	7.37E-02	135	1.59E-03	452	1.88E-02	154	8.20E-01	1020	9.59E-03	526	**4**	4.14E-02	261	6.14E-01	1054	2.83E-01	264	1.11E-05	353	1.68E-07	71	2.88E-01	676	5.12E-01	848	**3**	**7**	**493.44**
**56061**	**NM_019606**	**Mepce**	1.26E-02	197	4.54E-01	802	2.44E-02	95	4.05E-01	834	5.81E-01	727	1.28E-03	440	1.20E-03	29	2.34E-01	561	8.21E-03	509	**5**	1.76E-02	169	5.16E-04	147	6.94E-01	824	1.84E-01	984	9.07E-05	300	4.90E-01	833	1.00E-01	445	**3**	**8**	**493.50**
**23015**	**NM_001023567**	**GOLGA8A**	1.76E-02	231	6.15E-01	893	7.32E-05	5	6.59E-04	146	5.74E-01	718	3.78E-01	973	5.33E-02	284	1.06E-02	115	1.95E-01	822	**4**	1.94E-02	182	3.51E-03	279	2.82E-01	263	3.32E-01	1029	3.79E-02	741	7.21E-01	980	3.08E-02	236	**4**	**8**	**493.56**
**23522**	**NM_012330**	**MYST4**	2.58E-01	653	8.31E-03	146	1.01E-01	207	2.66E-02	413	4.02E-01	524	1.22E-03	436	1.31E-03	31	3.63E-01	677	1.07E-03	369	**5**	1.88E-02	177	3.30E-01	911	9.66E-01	1122	1.66E-02	831	2.67E-02	700	2.59E-01	652	5.94E-03	65	**4**	**9**	**494.63**
**1843**	**NM_004090**	**DUSP3**	8.77E-01	1043	3.85E-04	25	5.47E-01	674	4.16E-02	482	4.73E-02	96	1.53E-04	337	5.14E-01	890	2.39E-01	566	5.19E-06	155	**5**	9.47E-02	374	3.55E-01	929	2.61E-01	231	1.06E-03	674	3.92E-07	89	6.58E-01	944	8.21E-02	408	**2**	**7**	**494.81**
**55325**	**NM_018361**	**AGPAT5**	4.11E-02	415	1.73E-01	612	1.52E-01	250	6.16E-01	950	4.99E-01	640	1.29E-02	589	7.51E-01	1024	1.42E-01	438	6.48E-02	702	**2**	3.66E-02	250	8.34E-02	617	1.68E-02	9	1.23E-06	223	3.99E-08	51	2.02E-01	595	1.68E-01	560	**4**	**6**	**495.31**
**10802**	**NM_021982**	**Sec24a**	2.65E-02	320	6.50E-03	121	3.60E-01	464	7.06E-02	544	6.31E-01	792	3.41E-05	275	2.53E-01	658	1.84E-02	154	8.98E-11	15	**5**	2.34E-01	544	2.10E-01	772	7.47E-01	874	3.47E-08	41	5.76E-06	174	9.40E-01	1115	8.66E-01	1077	**2**	**7**	**496.25**
**28969**	**NM_014038**	**BZW2**	3.96E-01	760	9.02E-03	154	3.80E-01	481	4.74E-03	261	3.20E-01	432	2.16E-13	16	2.70E-03	55	5.96E-02	290	2.44E-06	132	**5**	9.12E-01	1093	5.60E-01	1032	9.18E-01	1052	4.42E-07	155	3.69E-02	740	1.37E-02	217	8.61E-01	1071	**3**	**8**	**496.31**
**9131**	**NM_001130846**	**AIFM1**	1.07E-01	541	1.45E-01	583	1.43E-02	69	2.10E-04	89	5.55E-01	694	1.25E-02	584	1.83E-02	150	6.89E-01	923	2.11E-03	411	**5**	8.72E-03	124	5.71E-02	568	7.94E-01	930	1.62E-05	383	4.39E-02	759	7.07E-01	972	1.91E-02	167	**4**	**9**	**496.69**
**202**	**NM_001624**	**AIM1**	5.07E-03	120	8.19E-01	1035	5.99E-01	737	7.72E-02	554	1.79E-01	245	6.00E-10	49	6.91E-01	994	6.22E-03	86	9.43E-04	359	**4**	8.44E-01	1048	1.18E-03	199	9.49E-01	1099	1.98E-04	564	9.63E-07	117	2.30E-02	280	1.09E-01	464	**4**	**8**	**496.88**
**9123**	**NM_001042422**	**SLC16A3**	2.94E-01	694	7.64E-04	35	8.80E-01	1051	7.49E-02	548	2.87E-01	393	3.50E-01	953	1.78E-01	546	8.20E-02	336	1.49E-02	566	**2**	8.46E-01	1051	1.18E-04	81	2.60E-01	229	5.58E-07	164	3.42E-02	729	5.18E-06	14	1.68E-01	562	**4**	**6**	**497.00**
**2224**	**NM_004109**	**FDX1**	1.20E-03	55	1.02E-01	542	8.56E-01	1023	9.76E-02	586	5.38E-03	14	1.02E-02	562	5.33E-03	79	9.11E-01	1082	4.14E-03	459	**5**	4.18E-02	265	3.81E-02	525	4.22E-01	493	9.97E-08	91	4.31E-04	367	4.81E-01	829	7.30E-01	986	**4**	**9**	**497.38**
**8996**	**NM_003946**	**NOL3**	3.76E-01	748	1.28E-01	567	5.35E-01	660	3.12E-01	777	9.22E-01	1070	4.75E-04	385	9.40E-01	1125	2.03E-03	51	7.77E-03	506	**3**	1.72E-01	478	1.67E-02	420	1.83E-02	10	1.19E-07	98	3.31E-06	159	2.75E-01	666	3.14E-02	238	**5**	**8**	**497.38**
**51202**	**NM_016355**	**DDX47**	6.77E-02	493	1.19E-02	185	6.66E-01	810	9.51E-01	1130	6.67E-01	827	1.13E-06	175	7.89E-01	1050	1.41E-02	134	1.35E-03	385	**4**	1.42E-02	149	1.03E-02	377	3.05E-01	299	2.38E-05	414	8.36E-02	816	1.31E-02	211	1.37E-01	507	**4**	**8**	**497.63**
**51118**	**NM_016037**	**UTP11L**	7.65E-01	955	4.95E-02	493	6.73E-01	823	5.45E-01	912	4.32E-02	88	4.53E-07	149	4.15E-01	822	1.19E-01	401	2.18E-02	584	**4**	8.71E-02	363	1.39E-01	702	2.62E-01	235	7.03E-07	182	1.15E-02	610	1.90E-04	60	1.92E-01	590	**3**	**7**	**498.06**
**10625**	**NM_006469**	**IVNS1ABP**	5.40E-02	475	1.64E-01	600	2.45E-01	348	4.08E-02	480	7.03E-01	873	6.13E-04	398	2.04E-04	10	3.89E-03	69	6.10E-03	490	**5**	7.27E-03	115	3.34E-04	118	9.39E-01	1081	9.26E-05	514	5.59E-03	548	3.33E-01	713	9.74E-01	1143	**4**	**9**	**498.44**
**23118**	**NM_015093**	**tab2**	1.80E-03	70	2.29E-01	648	7.73E-01	923	3.42E-01	796	4.20E-01	545	1.99E-05	254	1.30E-01	470	6.34E-02	302	1.12E-04	256	**3**	6.96E-02	324	1.94E-04	101	5.39E-01	656	8.16E-01	1136	1.30E-03	441	8.44E-02	449	2.10E-01	614	**2**	**5**	**499.06**
**29957**	**NM_013386**	**slc25a24**	9.53E-01	1105	7.85E-01	1014	6.06E-03	44	7.39E-02	546	4.61E-01	593	4.28E-07	148	8.98E-01	1101	7.62E-01	979	2.04E-05	191	**3**	6.32E-02	315	3.58E-04	125	5.63E-01	687	1.14E-06	215	1.77E-02	654	3.79E-03	145	1.34E-02	132	**5**	**8**	**499.63**
**7168**	**NM_001018004**	**TPM1**	8.20E-01	1004	2.99E-02	344	8.46E-01	1009	4.65E-02	498	4.02E-01	522	3.70E-02	680	1.19E-01	452	6.12E-02	293	5.72E-02	690	**3**	5.28E-01	833	5.27E-01	1021	3.73E-01	414	1.08E-07	95	1.03E-08	37	1.20E-03	101	1.33E-04	3	**4**	**7**	**499.75**
**6637**	**NM_003100**	**snx2**	7.30E-01	925	3.89E-03	87	9.24E-01	1090	2.26E-01	713	2.09E-02	35	1.41E-07	121	1.95E-01	575	8.29E-02	338	9.87E-11	17	**4**	4.26E-01	750	4.69E-01	996	2.56E-01	221	7.20E-05	498	2.45E-04	347	1.36E-01	520	4.11E-01	781	**2**	**6**	**500.88**
**55161**	**NM_018129**	**Pnpo**	4.90E-02	465	1.04E-01	545	3.33E-01	438	2.17E-01	708	2.88E-01	397	8.70E-04	419	9.72E-01	1145	1.57E-01	462	2.65E-08	62	**3**	8.38E-02	357	6.45E-04	162	7.52E-01	879	7.82E-06	330	8.42E-04	417	9.77E-01	1149	7.61E-03	84	**4**	**7**	**501.19**
**1176**	**NM_001833**	**CLTA**	4.87E-02	463	3.04E-02	350	2.27E-01	335	7.97E-04	159	2.47E-01	343	3.96E-01	978	1.07E-04	7	4.17E-01	729	4.83E-01	974	**4**	3.14E-01	644	4.71E-05	47	9.95E-01	1153	6.54E-07	177	3.15E-03	497	5.00E-02	381	4.20E-01	785	**4**	**8**	**501.38**
**79022**	**NM_024057**	**NUP37**	7.59E-01	949	8.84E-03	151	9.45E-01	1115	1.79E-04	85	1.06E-01	173	7.42E-03	541	7.10E-01	1002	5.78E-01	838	4.65E-06	149	**4**	2.57E-01	574	1.30E-02	393	1.08E-01	56	7.58E-01	1131	3.74E-05	251	5.40E-02	390	2.93E-02	227	**3**	**7**	**501.56**
**22890**	**NM_014950**	**ZBTB1**	6.93E-04	40	3.15E-01	713	2.12E-01	319	4.97E-01	880	7.63E-01	914	7.59E-04	413	7.87E-01	1047	3.05E-01	624	9.02E-05	248	**3**	9.80E-02	377	1.97E-04	102	6.99E-02	35	9.72E-04	667	2.25E-02	680	3.84E-01	757	2.66E-02	210	**4**	**7**	**501.63**
**51232**	**NM_016441**	**Crim1**	2.18E-02	279	4.30E-02	445	2.38E-01	342	8.74E-01	1086	5.54E-01	692	1.66E-05	242	5.33E-03	78	4.11E-01	725	3.90E-02	649	**5**	3.25E-01	651	6.12E-04	157	3.87E-01	441	3.28E-03	728	6.43E-08	57	2.35E-01	625	4.86E-01	829	**3**	**8**	**501.63**
**53**	**NM_001131064**	**ACP2**	1.47E-03	59	1.34E-01	573	6.43E-01	782	2.45E-02	405	2.64E-01	364	1.70E-02	615	9.56E-01	1134	2.31E-04	24	8.66E-01	1104	**4**	5.18E-01	826	2.89E-01	868	7.03E-01	831	2.71E-07	132	3.57E-07	87	6.71E-05	42	2.18E-02	181	**4**	**8**	**501.69**
**9857**	**NM_014810**	**CEP350**	7.18E-01	919	9.45E-03	160	7.25E-01	877	9.40E-02	582	2.60E-01	356	1.50E-10	37	5.87E-03	84	2.27E-02	170	1.85E-02	575	**5**	5.75E-01	866	2.50E-02	470	7.18E-01	846	1.25E-03	684	4.70E-02	770	2.24E-03	122	1.39E-01	512	**4**	**9**	**501.88**
**8886**	**NM_006773**	**DDX18**	3.48E-02	371	2.98E-01	701	5.07E-01	621	2.61E-01	742	1.88E-01	256	3.30E-02	669	2.69E-01	682	3.06E-03	63	8.23E-05	246	**4**	7.84E-04	44	4.32E-02	541	4.27E-01	504	1.58E-06	234	6.65E-01	1065	3.69E-01	746	1.66E-01	557	**3**	**7**	**502.63**
**990**	**NM_001255**	**cdc20**	8.33E-01	1011	3.93E-02	418	3.62E-01	466	1.61E-01	662	9.97E-02	166	2.42E-01	893	7.78E-02	374	1.31E-01	418	8.07E-06	167	**2**	7.86E-01	1007	3.31E-04	117	4.47E-01	540	7.62E-06	328	6.25E-07	100	4.93E-01	835	1.64E-01	554	**3**	**5**	**503.50**
**25813**	**NM_015380**	**Samm50**	3.89E-01	753	3.83E-03	86	4.40E-01	543	7.84E-01	1040	2.39E-01	326	2.43E-01	894	9.27E-01	1119	6.95E-02	311	7.42E-03	501	**2**	3.35E-02	236	4.98E-01	1010	8.97E-02	44	1.12E-05	356	2.35E-06	146	9.95E-02	476	2.83E-02	219	**4**	**6**	**503.75**
**10957**	**NM_006813**	**PNRC1**	9.00E-01	1059	6.03E-04	31	4.47E-01	549	1.61E-01	664	2.41E-01	331	2.31E-03	471	1.89E-01	567	6.64E-01	900	4.84E-03	474	**3**	3.80E-02	256	1.21E-01	674	5.06E-01	622	7.04E-08	71	6.21E-06	178	6.10E-03	169	8.33E-01	1045	**4**	**7**	**503.81**
**65260**	**NM_024033**	**C7orf49**	9.37E-01	1088	4.62E-02	466	1.56E-01	255	2.84E-01	758	4.71E-01	607	3.72E-02	681	7.67E-03	97	8.51E-01	1032	8.21E-01	1088	**3**	1.60E-01	463	3.89E-01	957	1.17E-01	65	3.07E-07	136	2.11E-07	74	1.57E-03	109	2.24E-02	190	**4**	**7**	**504.13**
**3556**	**NM_001560**	**IL13RA1**	1.16E-02	185	8.27E-05	14	1.17E-01	220	8.12E-01	1059	8.22E-01	979	2.98E-03	491	3.17E-03	60	2.10E-03	52	3.98E-04	306	**6**	7.02E-01	940	8.66E-02	623	2.24E-01	188	1.60E-02	829	7.20E-01	1084	7.16E-02	428	2.06E-01	609	**1**	**7**	**504.19**
**51517**	**NM_016453**	**nckipsd**	7.52E-02	502	5.34E-01	843	2.77E-03	25	1.31E-01	629	7.18E-01	881	3.35E-03	499	9.28E-02	406	2.22E-01	551	9.78E-01	1150	**2**	4.62E-02	277	5.84E-05	55	5.09E-01	627	9.40E-07	201	3.55E-05	247	5.33E-01	858	5.01E-02	319	**4**	**6**	**504.38**
**22877**	**NM_014938**	**MLXIP**	4.80E-01	805	1.49E-02	210	2.74E-01	370	4.20E-02	485	9.31E-02	155	4.99E-02	713	4.58E-02	261	5.06E-01	785	3.07E-03	435	**5**	6.41E-02	317	4.65E-01	995	4.51E-01	546	2.47E-01	1002	3.87E-03	512	3.53E-02	328	1.73E-02	152	**3**	**8**	**504.44**
**80135**	**NM_025106**	**SPSB1**	7.39E-01	936	2.30E-02	278	2.40E-01	345	1.01E-01	589	6.50E-04	3	1.38E-06	178	1.94E-01	572	6.43E-03	89	2.90E-03	431	**5**	4.45E-01	769	2.92E-03	264	6.10E-01	737	6.63E-05	491	7.94E-03	579	3.40E-01	722	8.91E-01	1091	**3**	**8**	**504.63**
**55164**	**NM_018132**	**Cenpq**	3.73E-02	393	8.50E-04	40	8.35E-01	999	6.99E-03	299	1.01E-01	168	3.38E-01	946	8.87E-03	103	5.60E-02	278	7.24E-04	334	**5**	2.48E-02	207	4.88E-01	1004	5.25E-01	644	4.87E-05	468	6.35E-04	393	3.93E-01	766	8.22E-01	1034	**3**	**8**	**504.75**
**58478**	**NM_021821**	**MRPS35**	4.21E-02	424	1.79E-01	619	8.93E-01	1062	5.25E-01	904	2.38E-01	325	4.24E-14	11	7.79E-03	99	9.62E-01	1124	4.06E-04	307	**4**	4.74E-04	28	4.18E-04	138	5.64E-01	689	1.98E-04	563	1.33E-06	123	1.31E-01	515	9.96E-01	1158	**4**	**8**	**505.56**
**10575**	**NM_006430**	**cct4**	8.89E-01	1052	7.57E-03	138	1.71E-01	276	3.17E-04	104	6.88E-01	851	9.56E-02	783	7.06E-01	1001	9.21E-01	1092	9.38E-09	52	**3**	4.35E-01	760	1.66E-02	419	1.76E-01	131	2.61E-06	260	5.47E-02	784	5.89E-05	38	5.67E-02	352	**3**	**6**	**505.81**
**7334**	**NM_003348**	**UBE2N**	2.36E-01	637	2.40E-02	284	8.98E-01	1068	3.81E-02	468	2.29E-01	309	8.02E-03	543	3.69E-03	66	6.78E-02	310	4.01E-07	101	**5**	2.84E-03	73	2.56E-02	475	6.80E-01	809	4.58E-06	292	3.40E-02	727	5.32E-01	856	8.66E-01	1079	**4**	**9**	**506.06**
**7520**	**NM_021141**	**XRCC5**	4.43E-01	781	1.58E-02	221	7.80E-01	933	1.42E-05	39	2.24E-01	303	2.92E-05	268	2.54E-01	659	6.95E-01	931	7.88E-04	343	**4**	9.65E-03	127	2.90E-01	871	4.78E-01	581	4.15E-05	451	6.98E-03	565	2.66E-01	655	6.51E-02	369	**3**	**7**	**506.06**
**6950**	**NM_006286**	**tfdp2**	6.68E-01	894	2.58E-01	680	6.74E-01	825	2.25E-02	397	1.05E-01	172	1.40E-03	445	1.05E-01	430	2.02E-01	525	1.42E-01	779	**2**	4.42E-03	93	2.47E-01	815	4.61E-01	565	4.11E-08	49	1.20E-04	317	7.28E-05	43	8.60E-01	1070	**4**	**6**	**506.19**
**6636**	**NM_003096**	**Snrpg**	1.92E-01	604	1.26E-02	195	6.31E-01	766	8.38E-01	1069	3.16E-02	64	1.82E-01	856	2.04E-01	591	6.29E-01	878	3.62E-01	918	**2**	1.35E-02	146	3.70E-02	523	2.88E-01	270	5.13E-05	474	6.22E-06	179	4.60E-03	156	8.42E-02	412	**5**	**7**	**506.31**
**3313**	**NM_007355**	**HSP90AB1**	2.52E-01	649	1.06E-01	548	7.90E-01	943	1.13E-02	342	7.89E-01	948	1.02E-01	792	9.35E-02	407	5.92E-01	854	4.29E-03	464	**2**	9.51E-03	125	5.56E-05	52	4.17E-01	490	7.64E-08	76	2.19E-03	466	4.82E-02	375	1.74E-01	572	**5**	**7**	**506.44**
**80232**	**NM_025196**	**GRPEL1**	7.00E-01	908	2.66E-02	315	4.11E-01	509	2.22E-01	711	9.76E-01	1128	5.12E-06	206	5.85E-04	18	1.71E-01	482	1.42E-03	386	**4**	1.00E-02	130	6.14E-03	329	9.48E-01	1097	1.26E-07	104	4.98E-03	538	9.65E-01	1137	1.01E-02	108	**5**	**9**	**506.63**
**64785**	**NM_022780**	**Rmnd5a**	9.43E-01	1094	8.46E-04	39	4.75E-01	583	3.64E-03	241	2.80E-02	55	5.40E-02	721	1.35E-02	122	6.91E-01	926	2.18E-01	839	**4**	2.55E-03	71	1.31E-01	688	3.70E-01	409	7.16E-06	325	1.43E-04	321	3.13E-01	695	7.22E-01	981	**3**	**7**	**506.88**
**81533**	**NM_030793**	**FBXO38**	4.03E-02	408	3.73E-02	401	5.94E-01	726	1.05E-01	595	8.62E-01	1017	1.87E-02	624	3.68E-01	780	2.45E-01	574	7.01E-03	496	**4**	1.95E-02	184	1.17E-02	388	6.25E-01	751	4.01E-07	150	1.57E-05	213	4.04E-02	350	1.04E-01	455	**5**	**9**	**507.00**
**23268**	**NM_015221**	**DNMBP**	1.55E-01	576	4.23E-05	12	6.68E-01	816	2.61E-03	217	5.91E-01	738	1.37E-12	21	1.50E-01	501	1.83E-01	504	4.87E-01	976	**3**	5.95E-01	878	9.77E-01	1155	3.76E-01	420	1.87E-05	394	1.95E-02	663	5.11E-03	161	7.82E-03	87	**4**	**7**	**507.44**
**54881**	**NM_198881**	**TBC1D8B**	1.07E-01	540	7.23E-03	132	3.70E-01	473	5.41E-01	910	3.18E-01	430	2.79E-01	920	9.94E-02	419	5.05E-01	782	4.62E-02	665	**2**	9.19E-01	1098	1.72E-03	226	2.42E-01	206	4.47E-06	290	1.89E-04	334	2.65E-03	127	1.74E-01	571	**4**	**6**	**507.69**
**10797**	**NM_006636**	**mthfd2**	8.37E-03	158	2.44E-01	662	8.34E-02	195	2.43E-02	404	3.41E-01	458	2.29E-06	189	4.87E-01	873	6.87E-01	922	1.12E-07	78	**4**	6.43E-01	902	3.35E-02	510	9.74E-01	1133	1.73E-05	388	7.26E-06	185	7.76E-01	1014	5.12E-03	60	**4**	**8**	**508.19**
**29980**	**NM_017613**	**DONSON**	4.98E-03	116	9.68E-02	538	1.01E-01	206	3.84E-02	471	2.31E-02	42	7.56E-01	1098	1.35E-01	475	9.82E-01	1143	2.66E-06	135	**4**	4.16E-01	745	3.26E-02	508	4.58E-01	558	6.67E-03	770	3.17E-09	24	6.64E-03	171	9.56E-01	1133	**4**	**8**	**508.31**
**7978**	**NM_006980**	**MTERF**	2.47E-01	643	2.15E-02	268	1.16E-01	219	7.83E-01	1037	2.72E-02	52	2.53E-01	902	4.44E-01	843	4.21E-02	248	4.50E-03	469	**4**	7.27E-01	960	2.45E-01	813	4.73E-02	30	1.00E-05	345	1.91E-02	661	8.01E-02	443	2.58E-02	202	**4**	**8**	**508.44**
**55589**	**NM_017735**	**TTC27**	2.51E-01	648	2.47E-02	292	5.18E-01	639	3.18E-02	437	7.14E-01	878	4.54E-01	1006	4.25E-03	72	8.33E-01	1027	1.75E-01	809	**3**	4.61E-03	96	2.24E-01	787	1.82E-01	142	4.70E-05	463	4.10E-03	518	8.55E-03	187	1.51E-02	142	**5**	**8**	**508.94**
**51379**	**NM_015986**	**CRLF3**	1.79E-02	234	6.93E-01	951	1.17E-01	221	4.67E-03	258	9.76E-01	1126	1.01E-08	77	3.61E-01	771	7.85E-01	994	2.34E-02	593	**4**	4.41E-02	272	4.33E-01	981	2.74E-01	252	2.93E-04	591	8.85E-06	192	1.40E-03	104	1.53E-01	534	**4**	**8**	**509.44**
**813**	**NM_001219**	**CALU**	9.24E-01	1074	8.94E-03	152	5.83E-01	715	1.06E-01	596	3.66E-01	482	2.24E-03	470	8.02E-02	381	8.77E-01	1056	8.01E-09	51	**3**	5.78E-01	867	2.43E-03	247	1.11E-01	61	2.24E-05	407	9.22E-06	194	2.71E-01	662	3.66E-01	745	**3**	**6**	**510.00**
**10799**	**NM_006638**	**RPP40**	9.96E-01	1148	2.89E-02	333	1.21E-01	222	1.23E-03	180	2.86E-01	389	3.28E-01	942	2.64E-01	672	2.37E-01	564	5.80E-02	692	**2**	3.01E-03	75	5.82E-01	1045	7.88E-02	39	4.77E-08	56	6.01E-01	1054	1.82E-01	574	2.15E-02	179	**3**	**5**	**510.25**
**55847**	**NM_018660**	**ZNF395**	8.16E-01	998	4.71E-01	812	4.41E-03	30	3.90E-04	119	2.61E-01	361	1.68E-08	87	7.63E-03	95	7.69E-03	97	1.27E-02	554	**6**	1.32E-01	423	2.74E-01	849	8.92E-01	1021	9.59E-04	665	3.78E-01	973	2.11E-02	270	4.63E-01	812	**2**	**8**	**510.38**
**2091**	**NM_004730**	**etf1**	2.01E-01	609	2.09E-02	264	9.68E-01	1136	5.31E-05	67	3.54E-02	71	1.66E-08	86	5.93E-01	942	1.65E-01	474	2.90E-01	879	**4**	6.09E-01	885	4.83E-03	311	2.89E-01	273	7.61E-05	501	9.26E-01	1141	1.30E-07	3	1.52E-01	530	**3**	**7**	**510.75**
**3029**	**NM_005327**	**hadh**	3.90E-03	99	6.19E-01	895	8.39E-01	1003	3.49E-02	451	5.88E-01	734	8.65E-05	315	8.05E-01	1063	1.48E-01	447	8.82E-04	354	**4**	2.31E-01	540	2.15E-01	777	1.46E-01	95	1.98E-04	562	7.71E-07	104	2.20E-02	273	1.07E-01	460	**3**	**7**	**510.75**
**9590**	**NM_144497**	**AKAP12**	3.72E-01	742	1.10E-04	16	2.21E-01	328	4.46E-04	127	3.92E-01	508	2.58E-01	906	4.40E-02	254	1.33E-01	423	9.02E-04	357	**4**	6.76E-01	920	8.57E-02	621	2.95E-01	283	3.04E-02	874	5.05E-01	1027	5.80E-03	168	2.23E-01	628	**2**	**6**	**511.38**
**81930**	**NM_031299**	**Cdca3**	9.85E-01	1136	8.62E-03	148	2.78E-02	105	6.80E-02	541	7.28E-02	133	7.39E-03	540	6.11E-01	953	4.35E-01	737	7.31E-02	710	**3**	1.87E-01	499	2.21E-02	454	2.77E-01	256	1.56E-05	380	5.38E-05	276	1.81E-01	573	3.71E-01	749	**3**	**6**	**511.88**
**3669**	**NM_002228**	**Jun**	2.41E-01	640	4.76E-02	478	5.40E-01	665	5.12E-02	514	2.49E-02	47	6.46E-12	26	5.25E-01	901	1.36E-02	130	3.32E-02	629	**5**	7.40E-01	972	2.24E-03	239	8.45E-01	978	1.36E-02	822	8.44E-04	418	3.11E-02	312	8.81E-02	420	**4**	**9**	**511.94**
**1615**	**NM_001141970**	**DAXX**	8.66E-04	45	7.47E-03	137	5.69E-02	169	2.45E-04	97	7.81E-01	937	2.18E-01	879	9.94E-01	1156	1.70E-01	479	7.07E-04	333	**4**	9.15E-02	368	1.48E-03	215	3.78E-01	422	1.32E-02	819	6.05E-04	389	7.63E-01	1004	3.78E-01	753	**3**	**7**	**512.63**
**10430**	**NM_032635**	**Tmem147**	2.12E-01	615	4.47E-02	455	2.09E-01	314	2.90E-05	56	3.37E-02	69	9.96E-07	168	5.26E-02	282	6.60E-01	898	2.21E-01	841	**4**	4.78E-02	279	2.17E-01	779	6.58E-01	785	4.68E-03	746	3.01E-01	953	2.75E-01	667	4.55E-02	295	**3**	**7**	**512.63**
**444**	**NM_001164752**	**asph**	7.24E-01	921	6.46E-01	912	4.22E-02	143	1.02E-01	593	3.79E-01	500	7.70E-09	72	6.21E-04	19	1.49E-01	451	5.70E-03	485	**4**	5.57E-02	295	3.60E-01	933	6.17E-01	743	5.14E-06	302	3.84E-03	511	1.93E-02	264	8.52E-01	1064	**3**	**7**	**513.00**
**4175**	**NM_005916**	**MCM7**	5.43E-02	476	1.21E-02	187	9.36E-01	1103	2.49E-03	212	5.97E-03	15	8.27E-02	768	4.48E-01	846	9.55E-01	1118	1.47E-09	31	**4**	1.24E-01	413	2.05E-03	233	7.15E-01	844	1.38E-06	229	4.95E-05	268	6.94E-02	424	8.33E-01	1044	**3**	**7**	**513.19**
**79677**	**NM_024644**	**C14orf16**	3.27E-02	356	7.33E-01	976	5.17E-01	635	5.99E-02	532	3.06E-02	62	1.77E-06	181	2.15E-01	610	5.47E-01	819	8.58E-03	512	**4**	4.43E-01	768	3.65E-02	522	8.18E-01	955	7.16E-05	496	4.31E-08	52	5.92E-05	39	3.05E-01	696	**4**	**8**	**513.19**
**54589**	**NM_018364**	**RSBN1**	1.57E-05	8	3.02E-03	75	2.24E-01	331	8.06E-01	1056	9.69E-01	1117	7.42E-02	752	5.62E-02	300	2.09E-02	162	2.84E-03	428	**4**	9.21E-01	1102	1.98E-01	755	4.29E-01	509	8.98E-08	84	2.25E-10	12	4.85E-02	376	9.83E-01	1147	**3**	**7**	**513.38**
**65018**	**NM_022914**	**acd**	4.65E-03	112	3.05E-01	706	6.58E-01	802	8.87E-04	167	6.03E-01	752	4.00E-08	100	1.99E-02	161	2.76E-01	598	3.12E-07	96	**5**	9.73E-01	1139	2.24E-01	788	8.53E-01	982	1.17E-07	96	8.43E-02	818	4.46E-01	803	9.33E-03	105	**2**	**7**	**514.06**
**26020**	**NM_014045**	**LRP10**	4.07E-02	411	5.70E-01	861	1.84E-01	286	1.10E-01	599	8.26E-01	984	1.85E-02	622	4.03E-01	812	2.12E-01	537	3.73E-03	450	**3**	2.96E-01	624	1.43E-05	27	3.11E-01	309	7.73E-03	778	5.21E-05	272	2.15E-01	612	3.84E-03	48	**4**	**7**	**514.50**
**4176**	**NM_001168335**	**ME2**	6.04E-01	860	4.84E-02	486	7.97E-01	952	3.96E-03	246	3.68E-02	74	7.13E-01	1088	5.52E-01	922	7.90E-01	996	1.85E-08	58	**4**	3.64E-02	247	6.73E-03	336	6.56E-01	783	7.89E-08	79	1.05E-04	309	3.70E-03	142	2.59E-01	657	**5**	**9**	**514.69**
**9943**	**NM_005109**	**OXSR1**	3.30E-03	92	9.45E-02	537	2.37E-01	340	2.02E-01	689	2.60E-01	357	1.79E-04	343	9.72E-04	24	9.19E-02	353	3.08E-01	889	**3**	3.52E-01	683	6.39E-04	161	6.75E-01	805	1.05E-02	802	1.04E-02	604	2.20E-01	615	6.70E-01	944	**3**	**6**	**514.88**
**51318**	**NM_016622**	**MRPL35**	9.83E-01	1135	1.63E-02	227	5.30E-01	652	4.17E-04	124	6.77E-02	127	2.50E-03	476	2.97E-01	707	6.36E-02	303	2.09E-09	36	**4**	1.61E-02	160	7.92E-02	609	9.31E-01	1073	1.31E-04	535	3.01E-01	954	1.97E-01	589	1.53E-01	532	**2**	**6**	**514.94**
**91300**	**NM_178812**	**MTDH**	4.71E-02	455	3.27E-01	724	8.12E-01	970	8.58E-03	316	5.13E-01	649	3.65E-09	62	8.27E-01	1075	1.27E-02	125	6.70E-05	237	**5**	7.05E-03	114	6.98E-01	1073	4.30E-01	511	6.37E-04	651	1.53E-02	640	1.76E-01	566	7.02E-03	77	**4**	**9**	**515.31**
**3417**	**NM_002168**	**IDH2**	1.78E-03	69	2.00E-01	629	3.56E-01	462	1.04E-04	77	1.13E-01	181	1.35E-04	330	6.47E-01	971	1.27E-01	409	7.98E-02	720	**3**	7.20E-01	951	6.64E-03	335	9.56E-01	1111	9.33E-04	662	8.25E-04	415	1.17E-01	500	9.04E-02	424	**3**	**6**	**515.38**
**3632**	**NM_001025243**	**Irak1**	1.71E-01	586	5.05E-01	829	2.47E-02	96	3.71E-02	465	8.26E-01	983	1.28E-02	588	8.16E-01	1067	1.97E-01	521	9.68E-02	742	**3**	3.54E-02	243	1.30E-03	204	7.18E-01	847	3.65E-06	284	5.34E-05	274	8.14E-02	444	6.88E-03	74	**5**	**8**	**515.44**
**23210**	**NM_001081461**	**JMJD6**	1.06E-01	539	3.12E-02	356	8.21E-01	984	5.09E-01	886	3.52E-01	469	1.30E-05	230	2.35E-01	631	7.97E-01	1002	5.64E-03	483	**3**	2.72E-01	590	6.23E-03	331	1.76E-01	130	1.73E-07	110	1.20E-08	38	2.71E-01	661	4.73E-01	821	**3**	**6**	**516.31**

**4627**	**NM_005966**	**NAB1**	6.47E-01	882	4.84E-02	487	8.57E-01	1025	1.78E-04	84	1.53E-01	219	1.12E-06	174	7.22E-01	1009	3.69E-01	681	3.60E-03	446	**4**	3.16E-02	230	1.05E-01	649	1.32E-01	78	2.96E-04	592	9.55E-01	1146	1.17E-03	99	1.08E-01	461	**3**	**7**	**516.38**
**1786**	**NM_001380**	**DOCK1**	1.11E-01	545	2.93E-02	336	4.82E-01	589	6.48E-01	967	5.72E-01	716	1.93E-04	346	4.85E-01	868	3.68E-01	680	7.87E-05	244	**3**	5.18E-01	825	3.54E-03	280	4.96E-01	603	2.06E-04	565	6.91E-06	184	4.35E-02	364	1.78E-02	157	**5**	**8**	**516.81**
**28977**	**NM_014050**	**MRPL42**	1.70E-01	585	9.06E-03	156	7.73E-01	924	2.09E-01	697	4.16E-02	83	1.25E-01	814	4.57E-02	259	1.24E-01	405	4.75E-10	24	**4**	9.69E-03	128	9.37E-04	180	7.09E-01	838	8.64E-06	337	6.84E-02	803	6.75E-01	955	8.70E-01	1081	**3**	**7**	**516.81**
**2023**	**NM_001975**	**eno2**	2.76E-06	3	1.10E-01	551	9.47E-01	1117	1.07E-04	78	8.11E-01	970	4.32E-01	1001	9.55E-01	1133	4.43E-01	743	1.11E-06	112	**3**	4.15E-04	24	1.14E-04	79	2.50E-01	210	1.55E-04	546	1.10E-02	607	2.10E-01	604	1.27E-01	495	**4**	**7**	**517.06**
**7453**	**NM_213645**	**wars**	4.41E-01	778	8.02E-01	1024	2.09E-01	313	3.39E-04	111	7.84E-01	943	2.59E-06	191	1.93E-02	157	9.81E-01	1142	1.97E-04	277	**4**	5.75E-01	865	7.70E-06	17	3.68E-01	408	2.47E-07	125	1.43E-03	448	8.07E-01	1034	9.66E-02	440	**3**	**7**	**517.06**
**51182**	**NM_016299**	**HSPA14**	2.82E-01	677	1.39E-02	201	6.00E-01	739	7.54E-01	1020	2.81E-02	56	2.37E-04	356	5.11E-01	888	8.49E-02	341	1.11E-02	538	**4**	9.53E-01	1124	3.00E-02	499	3.44E-01	363	8.03E-06	333	3.91E-01	978	5.22E-05	35	1.31E-02	128	**4**	**8**	**517.25**
**830**	**NM_006136**	**Capza2**	2.00E-02	252	9.64E-01	1134	5.30E-02	165	9.73E-01	1146	2.08E-02	34	5.45E-09	66	4.57E-01	853	1.88E-01	510	2.51E-09	39	**4**	6.05E-01	883	1.86E-02	742	8.83E-01	1013	1.87E-03	705	1.01E-08	35	9.06E-02	463	3.19E-02	239	**3**	**7**	**517.44**
**3329**	**NM_002157**	**HSPE1**	2.98E-01	700	1.10E-02	176	6.41E-01	779	5.89E-01	939	4.39E-01	574	1.46E-02	603	5.51E-02	292	7.41E-02	321	5.32E-12	8	**3**	2.47E-01	565	6.00E-02	571	8.44E-01	975	5.64E-07	166	5.63E-05	279	6.01E-02	408	6.32E-01	928	**2**	**5**	**517.75**
**3397**	**NM_002167**	**Id3**	9.80E-01	1130	7.30E-03	135	6.31E-01	767	2.52E-01	734	9.52E-01	1103	3.10E-07	142	6.49E-03	89	7.09E-02	316	2.87E-10	21	**4**	6.04E-01	882	3.10E-01	895	6.27E-01	753	3.99E-08	46	9.17E-05	301	4.24E-02	360	2.13E-01	616	**3**	**7**	**518.13**
**2029**	**NM_001136018**	**ephX1**	8.74E-02	519	5.11E-02	498	7.25E-01	876	1.34E-01	631	2.24E-03	6	1.23E-06	177	1.59E-01	515	7.90E-01	997	3.84E-09	42	**3**	2.91E-01	615	7.17E-01	1082	2.54E-02	18	7.87E-06	332	9.47E-03	591	7.30E-02	430	6.95E-01	967	**3**	**6**	**518.50**
**51499**	**NM_016399**	**TRIAP1**	9.39E-01	1089	2.11E-02	267	3.67E-02	129	3.51E-03	237	4.55E-01	586	8.56E-02	772	5.93E-01	944	4.84E-01	772	1.57E-01	791	**3**	2.37E-02	200	1.74E-01	727	7.72E-02	37	3.31E-08	40	6.88E-04	402	2.02E-01	592	3.32E-01	716	**3**	**6**	**518.81**
**10618**	**NM_006464**	**TGOLN2**	5.81E-01	851	2.20E-01	642	5.88E-02	174	9.05E-01	1106	1.43E-01	206	1.57E-07	124	3.77E-01	788	1.53E-01	457	7.40E-04	337	**2**	2.68E-01	587	1.04E-03	188	1.27E-02	7	1.54E-02	827	8.49E-04	419	4.38E-01	800	4.35E-01	796	**4**	**6**	**519.31**
**10431**	**NM_006327**	**TIMM23B**	5.97E-03	131	8.70E-02	535	4.25E-02	144	5.05E-05	66	1.57E-02	29	9.25E-01	1140	6.39E-01	969	1.08E-02	116	2.15E-01	836	**5**	7.58E-02	340	1.36E-01	695	6.85E-01	812	1.08E-04	522	4.12E-02	749	1.84E-01	576	2.56E-01	654	**2**	**7**	**519.63**
**5955**	**NM_181558**	**RFC3**	2.36E-01	636	1.11E-01	552	2.42E-01	346	4.57E-01	858	3.93E-02	78	1.11E-01	804	8.85E-02	395	3.14E-01	634	4.28E-07	102	**2**	2.65E-01	586	3.67E-04	128	5.63E-01	686	6.84E-03	772	2.05E-01	910	4.63E-01	820	6.17E-04	12	**3**	**5**	**519.94**
**4809**	**NM_005600**	**NIT1**	4.52E-01	787	3.82E-02	406	3.21E-01	427	3.99E-02	477	7.72E-01	926	8.43E-01	1116	1.22E-01	455	7.69E-03	98	3.75E-06	146	**4**	5.33E-02	294	2.83E-01	858	1.65E-01	121	4.63E-05	461	4.21E-01	992	2.85E-02	304	1.05E-01	456	**2**	**6**	**520.25**
**7184**	**NM_003299**	**Hsp90b1**	4.08E-02	412	8.84E-01	1085	2.10E-01	317	5.12E-01	890	5.68E-01	712	2.69E-07	138	5.09E-03	77	2.66E-01	588	1.27E-13	2	**4**	6.70E-04	40	1.28E-02	391	5.69E-01	694	1.92E-03	708	4.69E-02	769	6.85E-01	963	1.56E-01	539	**4**	**8**	**520.31**
**23002**	**NM_014992**	**DAAM1**	3.00E-01	704	4.04E-01	767	4.63E-02	153	3.90E-01	823	2.72E-01	375	1.26E-03	438	6.76E-05	5	8.75E-02	344	2.64E-02	604	**4**	2.31E-01	539	1.39E-04	89	7.32E-01	857	6.66E-05	492	8.46E-01	1115	7.26E-01	984	3.00E-03	44	**3**	**7**	**520.81**
**5687**	**NM_002798**	**PSMB6**	3.88E-01	751	7.13E-01	959	3.65E-02	128	5.05E-01	883	3.26E-01	438	5.34E-07	158	9.82E-02	416	2.34E-02	177	7.76E-03	505	**4**	7.40E-01	970	5.87E-03	325	1.34E-01	81	1.90E-01	985	4.96E-02	776	3.04E-02	309	1.19E-01	481	**3**	**7**	**521.38**
**22822**	**NM_007350**	**phlda1**	1.37E-01	567	8.38E-02	531	3.96E-01	494	8.53E-02	569	9.87E-01	1137	1.10E-09	52	2.07E-03	45	3.41E-02	217	1.07E-04	253	**4**	4.28E-01	752	1.88E-01	745	7.07E-01	836	5.16E-08	57	2.66E-02	697	3.40E-01	720	2.69E-01	672	**2**	**6**	**521.50**
**1508**	**NM_001913**	**CUX1**	1.39E-02	207	7.13E-01	962	9.56E-01	1124	2.66E-07	4	8.56E-02	148	1.27E-02	585	3.60E-03	62	8.68E-03	103	2.08E-01	833	**5**	1.39E-01	438	4.84E-01	1003	8.76E-01	1003	6.89E-02	933	2.06E-04	338	7.98E-06	17	1.86E-01	585	**2**	**7**	**521.56**
**51018**	**NM_016052**	**RRP15**	2.98E-01	699	2.65E-02	314	5.76E-01	708	1.43E-01	643	2.24E-01	302	5.87E-05	293	3.59E-01	769	1.08E-01	382	1.82E-03	403	**3**	4.50E-01	771	6.27E-02	575	9.50E-01	1100	1.02E-05	349	1.35E-03	443	1.58E-01	542	4.25E-03	53	**3**	**6**	**521.63**
**51192**	**NM_181641**	**Cklf**	3.48E-02	370	7.24E-03	134	1.79E-01	280	7.93E-04	157	8.66E-01	1018	2.07E-01	871	1.93E-02	156	4.57E-01	753	1.04E-01	748	**4**	6.19E-01	891	2.84E-01	859	8.32E-02	40	3.38E-04	600	8.97E-01	1128	2.36E-03	124	2.84E-02	221	**3**	**7**	**521.88**
**334**	**NM_001642**	**aplp2**	1.11E-02	178	9.42E-01	1117	4.90E-01	601	7.02E-03	301	4.59E-01	591	6.90E-04	407	5.03E-03	76	1.50E-01	453	3.65E-01	922	**4**	8.78E-01	1075	2.93E-02	493	3.35E-01	349	1.22E-05	364	5.59E-02	787	5.73E-03	167	1.16E-01	472	**3**	**7**	**522.06**
**6E+05**	**NM_033529**	**CDK11A**	5.43E-01	836	1.76E-01	616	3.68E-01	471	8.05E-01	1054	3.11E-06	1	2.41E-02	643	2.12E-03	46	6.29E-05	10	**6.29E-05**	235	**4**	2.81E-01	607	3.07E-01	890	3.42E-01	361	1.02E-04	518	2.14E-02	676	9.40E-01	1116	4.05E-02	274	**3**	**7**	**522.13**
**10628**	**NM_006472**	**TXNIP**	2.86E-03	86	4.93E-01	822	3.65E-01	469	7.98E-04	160	7.99E-01	960	2.25E-07	135	1.57E-01	512	4.51E-02	256	8.17E-06	168	**5**	3.86E-01	716	8.51E-03	359	7.53E-01	882	5.01E-05	471	8.65E-01	1120	9.04E-01	1087	1.74E-02	154	**3**	**8**	**522.31**
**3460**	**NM_001550**	**ifrd1**	2.12E-02	269	3.16E-02	359	6.44E-01	783	3.64E-02	460	7.79E-01	932	2.27E-02	635	2.13E-01	607	3.90E-02	232	1.84E-01	817	**5**	3.33E-01	663	5.39E-03	320	9.19E-01	1055	1.11E-05	354	1.24E-02	619	1.51E-02	235	8.74E-04	18	**5**	**10**	**522.38**
**1649**	**NM_001358**	**DHX15**	6.65E-01	891	8.98E-01	1092	4.93E-02	160	1.65E-01	666	2.73E-01	378	2.73E-04	363	3.48E-01	754	3.80E-01	696	2.49E-02	598	**3**	1.98E-03	62	8.16E-03	353	6.72E-01	800	5.85E-04	641	3.18E-09	25	3.54E-01	731	1.71E-02	150	**5**	**8**	**522.50**
**79568**	**NM_024598**	**C16orf57**	3.22E-01	713	1.05E-02	172	9.26E-01	1093	8.23E-06	32	2.36E-01	323	2.52E-01	901	1.82E-01	553	2.82E-01	601	1.64E-05	186	**3**	1.01E-01	384	3.00E-01	883	2.33E-01	194	4.86E-06	298	1.39E-04	319	7.80E-01	1016	3.07E-01	697	**2**	**5**	**522.81**
**84669**	**NM_001032291**	**Psrc1**	1.71E-01	587	9.17E-02	536	3.05E-01	413	3.30E-02	445	1.02E-01	169	8.37E-02	771	5.88E-02	310	5.89E-01	850	1.10E-03	371	**2**	8.57E-01	1061	2.75E-01	850	2.60E-01	228	2.88E-06	267	2.49E-05	223	5.74E-01	887	8.13E-02	402	**2**	**4**	**523.13**
**2181**	**NM_004462**	**FDFT1**	5.33E-03	123	5.72E-01	863	7.50E-01	899	3.61E-02	458	8.24E-01	981	2.31E-07	136	1.65E-02	138	5.68E-01	835	2.40E-10	20	**5**	3.51E-01	680	2.48E-01	817	4.16E-01	486	2.86E-05	430	6.92E-05	291	2.77E-02	303	6.04E-01	913	**3**	**8**	**523.31**
**55508**	**NM_017512**	**ENOSF1**	2.10E-03	72	8.79E-01	1082	2.81E-01	379	3.31E-01	791	9.21E-02	153	1.19E-01	808	9.10E-01	1109	9.99E-01	1158	3.18E-05	206	**2**	3.34E-01	665	5.75E-05	53	2.05E-01	167	8.67E-05	509	5.39E-03	547	2.53E-01	644	2.21E-03	30	**4**	**6**	**523.31**
**6897**	**NM_001008897**	**TCP1**	6.98E-01	906	8.32E-01	1044	2.20E-02	87	3.42E-05	58	4.15E-01	538	6.63E-01	1071	4.94E-04	16	4.59E-02	259	7.15E-05	239	**5**	9.93E-01	1152	9.97E-01	1157	7.48E-01	875	5.88E-06	310	3.65E-10	16	3.98E-02	345	4.72E-02	302	**4**	**9**	**523.44**
**51728**	**NM_016310**	**POLR3K**	4.51E-02	443	1.21E-03	47	4.33E-02	146	2.37E-03	208	1.10E-01	179	4.01E-01	982	3.99E-02	238	5.14E-01	791	5.65E-08	71	**6**	6.23E-01	894	1.06E-02	379	8.20E-01	956	5.27E-02	915	1.47E-02	634	7.95E-02	441	8.43E-01	1056	**2**	**8**	**523.75**
**55186**	**NM_018170**	**Rprd1a**	1.37E-01	568	1.25E-02	194	1.15E-01	217	6.42E-03	287	6.80E-01	840	9.72E-01	1147	4.96E-02	268	3.96E-01	710	5.82E-05	232	**4**	3.43E-01	673	1.20E-04	82	4.78E-01	578	1.34E-05	370	2.46E-06	148	8.52E-01	1055	7.77E-01	1012	**3**	**7**	**523.81**
**6728**	**NM_006947**	**SRP72**	1.52E-02	215	5.11E-01	835	8.16E-01	977	4.69E-04	131	6.10E-01	760	1.67E-07	126	1.00E-01	422	5.96E-01	855	3.10E-04	299	**4**	5.44E-01	844	3.52E-04	121	5.92E-01	715	2.09E-07	119	1.97E-01	906	6.51E-01	938	1.20E-02	119	**3**	**7**	**523.88**
**5036**	**NM_030979**	**PABPC3**	4.03E-01	764	2.07E-02	262	3.70E-01	472	4.48E-02	492	2.42E-01	335	5.19E-02	717	9.50E-01	1131	1.53E-01	456	1.01E-01	745	**2**	1.79E-02	171	9.89E-01	1156	3.12E-01	311	1.14E-06	216	3.60E-02	737	2.40E-04	66	5.74E-02	353	**4**	**6**	**524.00**
**2052**	**NM_001436**	**FBL**	3.76E-01	747	1.01E-02	166	3.96E-01	495	4.65E-01	863	4.66E-01	598	1.19E-02	579	2.51E-01	655	3.47E-02	219	2.30E-02	591	**4**	6.02E-01	881	4.21E-01	979	1.61E-01	110	8.07E-06	334	1.53E-02	641	3.54E-02	329	2.47E-02	200	**4**	**8**	**524.19**
**10813**	**NM_006649**	**Utp14a**	4.43E-01	780	4.73E-03	97	4.88E-01	598	8.94E-01	1096	4.04E-01	526	6.07E-01	1052	2.72E-02	188	6.64E-04	33	9.89E-01	1155	**3**	5.81E-02	301	1.15E-02	386	5.00E-01	611	5.33E-07	159	2.37E-04	342	3.91E-01	765	4.70E-02	298	**4**	**7**	**524.19**
**4259**	**NM_000247**	**micA**	3.09E-02	344	2.76E-01	686	6.10E-01	753	1.09E-02	339	6.17E-01	771	2.26E-06	188	5.72E-01	932	6.14E-01	868	4.19E-01	942	**3**	3.71E-02	253	1.66E-04	95	4.64E-01	569	5.40E-08	61	2.26E-02	681	1.72E-01	559	5.61E-02	348	**4**	**7**	**524.31**
**5351**	**NM_182943**	**PLOD2**	9.85E-01	1137	4.35E-02	448	6.30E-01	765	8.49E-01	1075	1.76E-01	240	2.88E-03	487	8.90E-02	397	7.84E-02	325	5.89E-03	487	**3**	4.34E-01	759	3.17E-03	270	2.87E-01	268	7.57E-07	189	1.34E-05	206	1.70E-01	558	4.08E-01	778	**3**	**6**	**524.31**
**10105**	**NM_005729**	**PPIF**	2.37E-04	28	8.34E-01	1048	1.56E-01	254	5.64E-01	922	5.32E-01	662	3.62E-01	960	4.84E-05	4	1.89E-01	513	3.45E-03	442	**3**	7.88E-03	120	4.38E-03	301	6.96E-01	825	2.71E-06	264	2.76E-08	47	5.51E-01	873	9.49E-01	1127	**4**	**7**	**524.38**
**55316**	**NM_018356**	**C5orf22**	1.03E-01	533	1.33E-01	571	3.57E-03	28	2.69E-02	416	2.76E-01	381	3.50E-03	502	7.20E-01	1008	3.63E-01	678	1.96E-01	823	**3**	2.93E-03	74	3.05E-01	889	2.65E-01	238	1.59E-03	696	7.84E-06	188	7.87E-01	1021	5.53E-02	344	**3**	**6**	**524.38**
**80324**	**NM_025232**	**REEP4**	3.60E-01	734	1.32E-03	49	3.55E-01	459	9.49E-02	584	8.06E-01	964	2.84E-02	658	4.62E-01	858	6.77E-01	914	4.08E-01	936	**2**	1.09E-01	395	1.47E-02	406	4.04E-01	465	8.51E-07	195	2.90E-05	233	8.54E-02	451	8.14E-03	92	**4**	**6**	**524.56**
**23787**	**NM_014341**	**MTCH1**	2.15E-02	275	4.67E-01	808	8.60E-01	1030	9.35E-02	581	8.16E-01	974	1.63E-03	453	9.92E-02	418	1.38E-01	429	1.55E-04	272	**3**	7.04E-01	941	5.73E-04	154	2.95E-02	20	3.18E-06	278	2.28E-02	683	6.67E-02	419	2.64E-01	661	**4**	**7**	**524.75**
**7905**	**NM_005669**	**REEP5**	4.64E-02	450	3.95E-01	762	1.50E-01	247	5.78E-02	527	7.55E-01	908	1.17E-07	119	3.88E-03	68	1.26E-01	408	2.49E-03	418	**4**	4.52E-03	94	8.72E-02	624	6.42E-01	772	8.56E-01	1141	3.25E-02	723	2.56E-01	649	1.28E-01	496	**2**	**6**	**525.38**
**63826**	**NM_022064**	**RNF123**	6.55E-01	884	6.85E-03	127	1.25E-01	227	2.67E-03	218	7.75E-01	928	3.62E-01	961	4.75E-01	864	9.72E-02	363	1.37E-04	266	**3**	1.55E-01	457	1.38E-02	400	3.28E-01	337	1.91E-06	245	2.51E-01	934	6.75E-03	174	7.90E-01	1021	**3**	**6**	**525.38**
**4141**	**NM_001042539**	**MAZ**	1.04E-01	537	8.93E-01	1090	7.06E-03	50	4.76E-01	869	9.09E-01	1058	1.05E-02	567	3.41E-01	745	1.84E-01	506	2.30E-06	129	**3**	4.38E-01	762	8.64E-03	361	2.89E-01	272	4.30E-07	154	3.57E-02	734	5.93E-02	405	2.00E-02	170	**4**	**7**	**525.56**
**55707**	**NM_018154**	**ASF1B**	6.48E-03	138	2.12E-01	639	7.82E-01	934	1.45E-01	646	4.84E-01	625	8.81E-05	316	4.97E-01	876	1.62E-01	469	5.74E-01	1007	**2**	5.71E-04	34	7.56E-04	169	4.46E-01	536	4.05E-04	613	2.61E-01	936	3.84E-03	149	5.15E-02	325	**4**	**6**	**525.75**
**26355**	**NM_014367**	**Fam162a**	3.45E-05	13	7.08E-01	957	4.96E-05	3	2.87E-03	227	4.20E-02	84	3.51E-02	675	5.68E-01	930	6.58E-03	90	2.10E-03	410	**7**	2.33E-02	197	5.47E-04	151	9.42E-01	1087	1.03E-02	799	9.16E-01	1136	9.28E-01	1106	1.62E-01	551	**3**	**10**	**526.00**
**7514**	**NM_003400**	**xpo1**	2.99E-01	702	3.60E-03	83	7.06E-01	856	1.86E-02	377	4.10E-01	533	1.25E-01	813	5.82E-01	939	9.74E-01	1134	2.25E-09	37	**3**	9.26E-01	1104	5.72E-03	324	2.54E-01	217	4.16E-04	614	2.41E-10	13	2.38E-02	287	7.22E-02	385	**4**	**7**	**526.13**
**7559**	**NM_016265**	**ZNF12**	5.01E-01	819	2.11E-02	266	3.49E-01	456	1.44E-01	644	6.77E-01	837	2.86E-02	659	2.83E-02	195	3.71E-01	686	2.42E-06	131	**4**	7.95E-02	348	9.66E-04	183	2.34E-01	195	1.90E-01	987	6.79E-01	1070	3.48E-01	728	2.78E-02	216	**2**	**6**	**526.25**
**55326**	**NM_018421**	**TBC1D2**	3.31E-03	93	7.30E-01	973	4.90E-03	34	9.74E-01	1147	1.46E-01	211	5.02E-06	205	5.02E-01	881	4.40E-01	740	1.34E-04	265	**4**	4.85E-02	282	4.57E-01	991	3.24E-01	327	2.74E-05	425	8.00E-01	1106	7.23E-04	88	2.55E-01	653	**3**	**7**	**526.31**
**822**	**NM_001747**	**capG**	1.54E-03	63	9.80E-01	1143	8.60E-01	1029	3.06E-01	771	6.57E-01	814	0.00E+00	1	3.87E-02	230	6.55E-01	892	3.27E-04	301	**4**	3.76E-01	709	3.25E-03	272	9.87E-01	1144	1.76E-04	557	4.57E-06	169	2.87E-07	4	5.13E-02	323	**4**	**8**	**526.38**
**9100**	**NM_005153**	**USP10**	2.30E-02	288	1.35E-01	574	2.04E-01	307	3.49E-01	804	3.06E-01	415	3.98E-01	980	8.18E-01	1069	6.66E-02	307	3.22E-02	625	**2**	6.13E-03	109	6.19E-04	159	7.00E-01	828	6.57E-03	766	2.05E-03	457	7.55E-02	436	4.72E-02	301	**5**	**7**	**526.56**
**51439**	**NM_016255**	**FAM8A1**	4.86E-02	461	7.82E-01	1011	4.83E-01	591	3.82E-02	470	4.38E-01	570	1.07E-02	571	4.20E-02	245	3.99E-01	717	4.73E-02	669	**5**	1.30E-01	418	2.55E-01	822	7.62E-01	894	3.56E-07	142	4.96E-07	93	5.11E-03	160	1.96E-01	596	**3**	**8**	**526.88**
**5935**	**NM_002897**	**RBMS1**	1.23E-02	193	8.79E-01	1083	5.75E-01	707	7.42E-03	306	4.29E-01	559	1.10E-01	801	2.14E-01	608	5.55E-01	826	6.21E-01	1026	**2**	1.74E-03	57	1.44E-03	212	8.06E-01	942	1.85E-06	241	3.52E-07	86	5.81E-02	403	7.44E-02	389	**4**	**6**	**527.44**
**5211**	**NM_012395**	**cdk14**	2.94E-01	693	1.11E-02	178	9.54E-01	1123	4.82E-03	262	2.45E-01	340	3.01E-01	928	7.95E-01	1053	9.74E-01	1135	7.71E-02	716	**2**	2.09E-03	63	8.65E-06	18	3.36E-01	351	2.34E-08	37	6.08E-03	557	5.07E-01	842	1.52E-02	144	**5**	**7**	**527.50**
**51491**	**NM_016391**	**NOP16**	5.97E-01	859	7.50E-01	992	4.74E-01	581	2.12E-02	388	1.64E-01	228	6.73E-04	405	2.38E-01	637	1.75E-01	491	5.46E-02	685	**2**	7.65E-03	118	8.88E-01	1136	4.47E-01	539	1.23E-07	101	5.61E-02	788	5.53E-03	165	5.34E-02	336	**3**	**5**	**528.06**
**92140**	**NM_033418**	**c1orf156**	1.16E-01	551	2.70E-02	319	1.53E-01	251	3.62E-01	810	6.66E-01	826	6.23E-03	530	1.85E-01	560	5.43E-01	814	4.96E-03	476	**3**	2.10E-01	522	3.41E-01	919	1.66E-01	122	6.50E-04	652	7.32E-03	571	7.73E-04	90	9.60E-02	437	**3**	**6**	**528.13**
**56851**	**NM_001038707**	**CDC42SE1**	4.16E-02	422	5.19E-01	838	4.79E-01	585	1.08E-02	338	1.92E-02	33	8.14E-05	313	1.91E-01	568	3.08E-03	64	1.02E-01	747	**5**	7.66E-01	994	5.81E-02	569	1.03E-01	53	2.26E-05	409	4.06E-02	748	4.10E-01	776	7.53E-01	1000	**2**	**7**	**528.56**
**2512**	**NM_199335**	**FYB**	3.92E-02	403	2.46E-01	664	2.79E-01	375	2.17E-04	91	2.41E-01	332	1.50E-03	447	7.17E-01	1007	2.73E-01	596	4.95E-01	979	**3**	7.50E-01	980	9.00E-03	365	4.99E-01	609	6.12E-05	484	4.39E-02	760	1.84E-04	59	4.89E-02	309	**5**	**8**	**528.75**
**7110**	**NM_022648**	**TNS1**	8.21E-01	1006	3.51E-02	384	1.01E-01	205	7.28E-01	1004	6.10E-01	757	3.30E-05	274	8.85E-01	1092	4.53E-01	750	5.03E-05	226	**3**	2.96E-02	223	1.57E-05	29	1.50E-01	100	1.41E-04	543	5.00E-03	539	2.85E-01	673	2.57E-01	655	**4**	**7**	**528.75**
**11261**	**NM_007236**	**Chp**	1.07E-02	175	7.53E-01	995	4.67E-01	574	1.48E-01	649	2.29E-01	311	2.34E-05	262	7.94E-01	1052	9.89E-02	366	5.45E-04	321	**3**	2.07E-01	517	1.68E-04	96	9.66E-01	1120	2.80E-05	428	4.39E-02	758	2.51E-01	640	2.45E-02	198	**4**	**7**	**528.88**
**55173**	**NM_018146**	**RNMTL1**	2.42E-03	78	7.02E-01	956	1.50E-01	249	1.73E-01	676	4.37E-01	568	5.27E-03	521	1.53E-01	506	3.23E-02	210	2.24E-01	843	**3**	3.37E-01	666	1.40E-02	402	5.43E-01	662	6.22E-07	173	1.15E-02	611	1.56E-01	537	4.60E-01	809	**3**	**6**	**529.19**
**2107**	**NM_001014763**	**etfB**	8.04E-01	989	4.66E-01	806	5.11E-03	37	7.07E-04	148	1.81E-01	248	2.89E-03	488	9.67E-01	1143	2.20E-02	169	9.02E-01	1115	**4**	2.14E-02	187	1.92E-01	751	4.31E-01	513	2.68E-05	423	7.49E-02	810	1.48E-02	230	8.64E-02	415	**3**	**7**	**529.50**
**51287**	**NM_016565**	**CHCHD8**	3.05E-01	706	5.00E-03	105	1.67E-01	268	2.88E-02	423	8.06E-01	965	5.43E-04	389	2.33E-02	179	5.28E-01	800	1.15E-01	760	**4**	3.15E-01	645	2.03E-01	764	3.99E-01	462	6.66E-07	178	4.22E-01	994	1.89E-01	581	3.81E-02	267	**2**	**6**	**530.38**
**9541**	**NM_004882**	**cir1**	3.83E-03	97	8.30E-01	1043	4.16E-01	513	4.36E-01	847	7.01E-01	865	1.28E-02	587	5.11E-02	275	6.20E-01	873	2.43E-05	196	**3**	3.51E-01	682	6.91E-01	1071	1.01E-01	52	2.07E-05	401	2.05E-08	44	1.93E-01	583	5.94E-02	359	**2**	**5**	**530.50**
**79081**	**NM_024112**	**C9orf16**	2.18E-01	619	3.93E-02	417	2.68E-01	364	9.60E-01	1139	2.53E-01	350	4.44E-03	514	1.30E-01	469	3.65E-01	679	3.60E-01	915	**2**	5.63E-01	857	6.86E-04	164	3.76E-01	419	2.13E-07	120	2.62E-01	937	7.18E-03	179	5.59E-02	347	**3**	**5**	**530.56**
**51340**	**NM_016652**	**CRNKL1**	3.86E-02	401	7.70E-01	1001	9.38E-03	57	9.23E-01	1120	2.29E-01	310	1.55E-07	123	1.35E-01	478	4.64E-01	756	3.93E-08	66	**4**	2.73E-01	592	4.95E-02	555	8.02E-01	940	4.48E-09	18	1.09E-01	839	6.34E-01	928	4.83E-02	308	**3**	**7**	**530.75**
**10109**	**NM_152862**	**arpc2**	2.41E-02	299	3.47E-01	735	7.91E-01	944	7.85E-04	155	3.81E-01	502	7.76E-07	164	7.92E-02	379	5.11E-02	273	1.20E-02	544	**4**	9.38E-01	1114	1.65E-02	417	6.74E-01	802	1.39E-01	969	1.54E-02	642	4.76E-03	157	8.07E-02	400	**3**	**7**	**531.00**
**29028**	**NM_014109**	**ATAD2**	7.79E-01	963	5.36E-03	108	2.56E-01	358	4.50E-01	854	5.15E-01	651	7.41E-05	307	2.20E-02	174	1.44E-01	442	1.14E-04	258	**4**	4.04E-01	736	1.38E-01	699	7.54E-01	883	5.81E-01	1091	6.66E-08	58	4.98E-01	836	7.44E-03	81	**2**	**6**	**531.19**
**8795**	**NM_147187**	**tnfrsf10**	2.79E-01	670	1.30E-03	48	1.71E-01	278	1.30E-01	628	9.73E-01	1121	1.80E-03	457	5.59E-02	296	1.55E-01	459	1.08E-03	370	**3**	1.57E-01	459	4.18E-01	978	2.98E-01	288	1.52E-01	974	6.03E-02	795	2.27E-02	278	8.19E-02	405	**1**	**4**	**531.50**
**1488**	**NM_147782**	**CTSB**	2.74E-01	663	5.92E-01	875	6.93E-01	849	4.97E-03	266	1.43E-01	205	1.94E-05	253	9.00E-02	399	4.36E-03	73	8.93E-01	1112	**3**	3.66E-01	699	3.69E-01	943	1.23E-01	70	5.42E-05	478	7.37E-02	809	1.29E-02	209	2.01E-01	604	**2**	**5**	**531.69**
**2960**	**NM_002097**	**GTF3A**	1.44E-02	210	5.19E-02	500	8.64E-01	1033	2.01E-02	383	6.00E-01	749	2.22E-04	351	6.84E-01	992	2.25E-01	554	1.71E-08	57	**4**	7.75E-01	1003	2.52E-01	821	3.88E-01	444	1.60E-05	382	5.24E-02	780	4.93E-04	78	2.09E-02	175	**3**	**7**	**532.00**
**8450**	**NM_003588**	**CUL4B**	3.93E-01	757	5.42E-01	849	4.60E-01	563	2.17E-01	706	5.40E-01	675	3.74E-01	971	4.07E-03	69	3.31E-01	646	8.72E-06	171	**2**	2.52E-03	70	8.01E-03	352	2.37E-01	202	1.02E-05	348	1.95E-02	665	2.99E-01	682	4.21E-01	786	**4**	**6**	**532.00**
**2222**	**NM_002004**	**FDPS**	1.47E-02	211	1.73E-01	610	9.13E-01	1080	2.93E-02	425	1.21E-02	23	1.12E-02	572	7.77E-01	1041	2.91E-01	611	1.10E-01	754	**4**	1.26E-01	415	3.87E-04	132	4.47E-01	538	4.11E-09	17	5.72E-01	1044	2.58E-02	295	3.69E-01	748	**3**	**7**	**532.25**
**5721**	**NM_000963**	**PTGS2**	1.13E-01	549	4.88E-02	488	1.96E-01	301	8.28E-01	1066	1.07E-01	174	7.41E-04	412	7.48E-03	94	6.91E-01	925	8.80E-01	1108	**3**	1.14E-01	405	5.40E-04	149	2.12E-01	179	1.33E-04	537	4.34E-01	1001	3.32E-02	319	4.61E-01	810	**3**	**6**	**532.31**
**10062**	**NM_005693**	**NR1H3**	2.10E-02	267	9.45E-01	1120	8.15E-01	974	1.15E-01	609	3.75E-01	493	2.82E-01	656	7.54E-01	1028	3.18E-03	65	2.38E-03	416	**4**	7.17E-02	331	4.39E-01	985	5.69E-03	4	2.47E-04	583	7.52E-09	31	5.46E-01	868	7.91E-03	90	**4**	**8**	**532.50**
**2589**	**XM_002347288**	**GAPDH**	2.29E-03	75	7.38E-01	981	1.69E-01	270	1.86E-01	682	2.23E-01	301	4.94E-05	286	4.88E-01	874	3.31E-02	212	5.04E-02	673	**3**	6.88E-01	928	4.00E-01	967	5.73E-01	698	7.07E-05	495	2.16E-01	919	1.53E-05	25	1.44E-02	139	**3**	**6**	**532.81**

2E+05	NM_138288	c14orf14	2.47E-01	644	3.97E-02	420	1.23E-01	224	4.80E-01	871	7.49E-01	904	7.94E-08	115	3.20E-01	726	1.73E-02	149	7.72E-04	341	4	2.50E-04	16	3.77E-01	948	5.77E-01	700	3.38E-02	887	6.53E-04	397	3.84E-01	758	9.13E-02	427	3	7	532.94
5550	NM_002736	prkar2b	4.52E-01	786	1.09E-03	44	1.69E-01	271	3.32E-01	792	6.28E-01	785	1.80E-03	458	2.12E-01	602	3.07E-01	625	4.05E-02	654	3	1.06E-01	391	1.16E-01	664	2.05E-01	166	3.30E-04	597	2.69E-01	939	2.10E-01	606	1.73E-02	153	2	5	533.31
79023	NM_024063	SPATA5L1	1.15E-01	550	1.43E-02	204	8.90E-01	1058	1.48E-01	650	5.05E-02	102	6.19E-10	50	1.89E-01	566	3.10E-01	630	4.93E-09	47	3	4.86E-01	804	2.97E-03	266	2.87E-01	269	1.52E-02	826	3.14E-02	722	5.71E-01	884	6.03E-01	911	3	6	533.69
80127	NM_025065	RPF1	3.57E-01	733	2.53E-02	301	5.06E-01	619	7.80E-02	555	8.48E-01	1003	1.26E-10	35	3.51E-01	763	2.29E-01	557	1.53E-02	568	3	7.14E-02	328	4.57E-02	549	7.42E-01	868	2.07E-04	566	2.87E-02	711	3.77E-03	143	3.60E-02	256	5	8	534.69
9913	NM_014860	SUPT7L	5.66E-01	841	2.99E-02	343	8.66E-01	1035	3.08E-03	231	2.39E-01	327	1.28E-01	818	2.44E-01	647	6.62E-01	899	2.98E-07	94	3	1.38E-01	436	4.13E-04	137	2.30E-01	192	1.35E-04	538	1.00E-02	597	6.64E-01	949	1.18E-01	478	3	6	535.13
1841	NM_004417	DUSP1	9.82E-01	1133	4.24E-02	436	5.01E-01	611	2.53E-03	214	7.67E-01	920	2.67E-04	360	1.24E-01	460	2.89E-04	26	6.33E-02	698	4	2.95E-01	622	1.43E-02	404	1.81E-01	139	1.28E-06	225	5.80E-04	383	8.71E-01	1064	5.45E-01	870	3	7	535.31
7378	NM_003364	upp1	1.02E-02	173	6.24E-02	514	1.67E-01	269	1.11E-02	340	8.39E-01	993	6.99E-01	1083	8.93E-03	104	8.23E-05	13	5.67E-03	484	5	2.79E-01	604	3.91E-01	958	3.79E-01	426	9.55E-04	663	3.27E-03	501	2.92E-01	677	3.96E-01	767	2	7	535.56
5707	NM_002814	PSMD10	2.60E-01	656	2.70E-02	318	2.76E-02	104	3.59E-03	239	9.77E-01	1132	1.72E-05	246	1.72E-01	538	2.60E-02	188	8.14E-01	1083	5	1.52E-01	448	3.23E-01	906	3.05E-01	302	1.26E-04	532	4.84E-03	533	4.44E-01	802	1.59E-01	546	2	7	535.81
55823	NM_018447	Tmem111	3.77E-02	396	3.26E-01	723	1.61E-01	260	7.52E-01	1018	1.25E-01	193	3.00E-03	492	1.12E-01	442	3.31E-02	213	5.67E-01	1002	3	5.21E-02	288	4.12E-02	536	1.18E-01	66	6.51E-01	1107	3.97E-04	363	3.63E-01	738	3.59E-01	738	2	5	535.94
25865	NM_001079880	PRKD2	1.40E-01	569	7.11E-01	958	5.92E-01	723	4.52E-04	128	9.74E-01	1124	1.17E-02	577	3.97E-02	236	2.13E-01	538	5.17E-02	675	3	1.12E-04	9	4.98E-04	146	2.44E-02	17	1.65E-05	384	1.60E-01	888	9.14E-01	1099	1.36E-01	506	4	7	536.06
27346	NM_014573	TMEM97	9.17E-01	1066	1.54E-02	217	9.33E-01	1101	1.54E-01	656	8.29E-02	145	1.62E-14	9	5.10E-01	886	1.18E-03	40	5.20E-01	985	3	2.77E-01	598	3.63E-04	126	9.85E-01	1139	3.55E-08	42	8.23E-04	414	2.99E-02	308	5.10E-01	845	4	7	536.06
7803	NM_003463	ptp4a1	6.44E-01	877	6.46E-02	518	6.98E-01	852	2.18E-01	709	4.61E-03	13	6.52E-01	1065	6.20E-02	326	1.94E-03	48	8.50E-06	170	3	3.55E-02	244	1.21E-01	671	4.68E-01	572	1.06E-02	803	1.31E-01	869	7.33E-02	431	8.39E-02	411	2	5	536.19
55272	NM_018293	ZNF654	3.93E-01	756	2.32E-02	279	7.52E-02	189	1.60E-02	364	4.80E-02	98	1.63E-01	845	2.91E-02	200	4.79E-02	268	7.38E-01	1057	5	1.55E-01	456	1.42E-02	403	8.81E-01	1012	7.94E-07	193	3.14E-06	156	9.77E-01	1148	9.94E-01	1155	3	8	536.19
7812	NM_007158	CSDE1	6.23E-01	868	6.84E-01	940	2.43E-02	94	1.35E-03	186	5.25E-01	659	6.21E-04	400	2.95E-01	706	8.01E-04	35	3.69E-01	923	4	7.31E-01	962	4.10E-01	975	4.26E-01	502	3.75E-04	609	1.35E-06	125	8.67E-04	91	1.37E-01	508	3	7	536.44
54969	NM_001039140	C20orf27	5.29E-02	474	2.97E-02	342	2.05E-01	309	1.13E-01	607	2.39E-01	328	1.58E-03	451	3.87E-01	797	5.16E-01	792	1.07E-01	752	2	3.22E-03	80	1.98E-02	441	9.53E-01	1105	7.71E-06	329	1.13E-01	849	3.90E-01	764	1.82E-02	163	4	6	536.44
23760	NM_012399	PITPNB	9.54E-01	1107	4.28E-02	444	5.56E-01	681	4.20E-02	486	7.58E-01	910	2.74E-04	365	1.27E-01	465	5.91E-01	852	1.40E-02	560	4	3.62E-02	246	2.73E-03	255	6.45E-01	775	4.25E-05	455	7.28E-04	406	1.10E-02	202	6.74E-02	375	5	9	536.50
2629	NM_013976	gcdH	9.63E-03	168	3.81E-01	755	4.42E-01	546	1.96E-01	688	5.23E-01	658	4.87E-02	708	8.98E-02	398	3.34E-02	214	1.05E-01	750	3	8.59E-01	1064	2.01E-01	760	1.29E-01	75	6.23E-07	174	5.70E-04	382	3.95E-02	344	5.88E-01	901	3	6	536.56
56257	NM_021254	C21orf59	2.25E-01	628	3.90E-02	413	8.76E-01	1044	2.75E-06	15	1.77E-01	242	7.83E-02	754	1.50E-01	500	2.29E-01	558	4.11E-01	940	2	5.75E-02	299	2.00E-01	759	5.10E-01	628	1.71E-04	553	6.59E-01	1062	4.57E-03	155	2.75E-03	39	3	5	536.81
60496	NM_021933	MIIP	4.70E-02	453	1.63E-01	599	1.13E-01	215	8.67E-01	1082	4.84E-01	626	1.17E-05	226	2.03E-02	165	3.31E-02	211	4.72E-04	317	5	9.86E-01	1146	9.69E-01	1153	4.51E-01	547	1.92E-06	247	4.43E-05	261	6.99E-01	967	6.74E-02	374	2	7	536.81
1877	NM_001168319	EDN1	1.12E-01	547	1.47E-02	208	4.56E-01	556	3.07E-01	772	3.46E-01	462	6.62E-04	403	6.21E-02	328	1.48E-01	449	1.35E-06	117	3	5.86E-01	872	3.57E-04	124	3.37E-01	353	1.25E-04	530	2.36E-02	685	9.83E-01	1154	8.20E-01	1033	3	6	537.06
102	NM_001110	ADAM10	5.01E-01	818	5.46E-02	506	3.02E-01	407	4.37E-01	848	8.72E-01	1023	8.05E-02	760	3.00E-02	202	4.48E-01	746	2.34E-07	89	2	3.84E-01	713	7.35E-01	1088	1.94E-01	150	1.74E-02	835	1.48E-05	209	9.14E-04	94	1.04E-02	112	4	6	537.50
9833	NM_014791	Melk	8.01E-01	983	3.13E-01	710	3.43E-02	124	1.41E-02	359	1.16E-01	185	1.39E-01	830	4.69E-01	861	9.38E-01	1102	4.97E-10	25	3	2.98E-01	625	4.27E-02	540	4.92E-01	597	4.29E-04	616	8.11E-07	107	1.45E-02	225	3.24E-01	712	4	7	537.56
9611	NM_006311	NCOR1	2.13E-02	271	3.62E-01	742	5.89E-01	720	2.71E-03	220	7.01E-01	866	3.49E-02	674	8.04E-01	1061	6.19E-03	85	9.20E-01	1121	4	2.09E-01	521	4.59E-03	308	2.27E-01	190	3.24E-07	137	2.48E-02	692	2.10E-01	605	7.91E-02	396	3	7	538.06
25979	NM_015510	DHRS7B	4.96E-02	469	5.01E-02	497	8.02E-01	958	1.59E-01	660	7.92E-01	951	1.53E-03	449	7.97E-01	1055	2.63E-04	25	2.13E-02	582	4	3.75E-02	254	3.13E-01	899	2.42E-01	207	3.65E-06	283	5.48E-02	785	3.66E-02	336	2.46E-02	199	4	8	538.06
10549	NM_006406	Prdx4	3.49E-04	34	4.34E-01	786	5.15E-01	631	1.01E-01	592	3.73E-01	491	5.08E-08	107	2.71E-02	187	9.38E-01	1101	4.36E-05	221	4	4.92E-01	808	3.96E-04	134	6.49E-01	777	3.21E-03	727	2.33E-03	471	6.91E-02	423	9.41E-01	1124	3	7	538.38
54927	NM_017840	mrpl16	3.72E-02	390	4.34E-01	784	5.28E-02	164	4.19E-02	483	2.73E-01	376	1.30E-03	441	7.76E-01	1039	2.79E-02	195	1.09E-09	29	5	1.89E-01	502	9.69E-01	1154	6.04E-01	730	9.65E-06	342	6.20E-04	390	4.38E-01	798	4.44E-01	801	2	7	538.63
10507	NM_001142287	SEMA4D	7.21E-05	16	1.43E-01	581	5.77E-01	709	9.04E-04	168	4.56E-01	587	2.29E-02	638	3.78E-01	790	7.08E-01	942	5.78E-02	691	3	2.88E-02	219	1.35E-02	398	4.99E-01	608	7.02E-05	494	4.24E-01	995	3.62E-03	141	2.49E-01	647	4	7	539.00
2932	NM_001512	GSTA4	6.87E-01	901	1.62E-01	595	1.22E-02	65	1.34E-02	352	5.16E-01	652	6.08E-01	1053	9.47E-01	1129	3.43E-01	657	2.88E-07	93	3	4.78E-01	798	6.12E-01	1053	1.20E-01	67	1.80E-06	239	1.89E-05	216	1.68E-01	553	2.60E-02	203	3	6	539.13
4709	NM_005004	Ndufb8	1.26E-01	559	3.71E-02	399	7.08E-01	857	2.52E-02	407	6.28E-02	121	2.16E-01	877	4.01E-01	810	2.67E-01	589	1.90E-09	35	3	1.48E-01	444	1.94E-04	100	1.74E-01	125	5.24E-02	913	8.21E-01	1108	3.13E-02	313	7.01E-01	970	2	5	539.19
51076	NM_015960	cutC	2.15E-02	273	1.40E-02	202	8.33E-01	994	3.34E-03	235	2.73E-01	377	1.29E-05	229	3.66E-01	777	2.20E-01	547	9.98E-03	528	5	5.27E-01	832	3.04E-02	502	3.14E-01	315	1.18E-03	682	8.58E-05	298	6.18E-01	911	6.28E-01	926	3	8	539.25
51428	NM_016222	DDX41	4.28E-02	431	2.47E-01	666	7.17E-01	868	1.91E-02	379	2.13E-01	285	3.24E-01	939	3.63E-02	223	2.73E-02	193	8.17E-01	1086	4	7.51E-02	337	3.70E-03	289	4.70E-01	573	4.43E-01	1057	1.11E-04	314	7.24E-01	983	3.22E-04	5	3	7	539.25
1434	NM_001025105	csnk1a1	7.28E-03	144	4.59E-03	94	6.86E-02	182	8.74E-04	166	7.84E-01	944	2.85E-03	485	4.31E-01	832	9.43E-01	1105	4.31E-07	103	5	8.79E-01	1076	4.01E-01	968	5.15E-01	632	7.24E-07	183	4.26E-05	258	9.56E-01	1129	5.22E-02	331	2	7	539.50
25800	NM_001099406	SLC39A6	1.67E-03	66	5.01E-01	826	5.23E-01	644	4.39E-03	251	6.76E-01	835	1.97E-07	132	2.01E-02	164	4.15E-01	726	8.55E-01	1098	4	1.04E-01	387	2.62E-03	251	9.07E-01	1036	2.83E-03	718	9.34E-02	827	5.78E-02	402	3.83E-02	269	3	7	539.50
1478	NM_001329	CTBP2	4.87E-01	808	4.89E-02	489	1.04E-01	210	1.02E-01	594	6.37E-01	794	7.12E-03	538	9.25E-03	105	6.67E-02	308	2.98E-02	614	4	1.68E-01	473	1.00E-01	644	4.73E-01	575	6.31E-06	317	5.38E-03	546	9.33E-01	1109	1.48E-01	523	2	6	540.44
64757	NM_022748	TNS3	6.12E-01	864	2.92E-02	334	7.41E-01	890	8.28E-04	162	2.50E-01	346	1.64E-04	338	1.13E-02	113	4.17E-01	730	1.82E-01	815	4	3.90E-04	22	5.09E-02	556	3.61E-01	395	5.87E-02	921	9.47E-03	592	3.27E-01	710	5.30E-01	860	2	6	540.50
8493	NM_003620	PPM1D	2.64E-02	319	4.12E-01	772	9.66E-01	1134	2.00E-02	382	1.29E-02	24	6.59E-01	1068	1.94E-01	574	1.97E-03	50	5.08E-05	227	5	3.84E-01	714	4.77E-01	999	1.99E-02	11	1.20E-05	362	2.28E-01	926	1.97E-05	28	8.55E-01	1066	3	8	541.00
10213	NM_005805	PSMD14	2.18E-01	622	4.24E-02	437	2.50E-01	355	2.08E-01	695	3.28E-01	443	4.21E-09	63	8.08E-01	1065	1.74E-02	150	4.45E-04	314	4	7.20E-01	952	5.05E-01	1013	4.60E-01	561	1.14E-06	214	2.59E-03	481	3.36E-02	321	7.01E-01	971	3	7	541.06
85377	NM_138773	SLC25A46	4.22E-01	771	4.79E-01	814	1.49E-02	71	6.08E-01	948	2.67E-01	368	3.48E-07	146	6.38E-01	966	3.97E-01	711	3.32E-05	207	3	7.06E-02	326	2.81E-06	9	4.33E-01	1089	1.17E-06	218	4.92E-02	774	7.75E-01	1012	3.04E-02	233	4	7	541.44
29087	NM_199297	THYN1	3.68E-02	385	3.30E-01	727	5.93E-01	724	1.72E-01	674	3.30E-01	445	7.99E-02	757	1.99E-02	163	4.73E-03	77	2.70E-01	861	3	5.85E-01	871	2.11E-02	449	1.97E-01	153	4.31E-04	617	6.76E-07	102	3.80E-01	755	5.98E-01	908	3	6	541.75
10237	NM_005827	SLC35B1	2.42E-02	300	1.57E-02	220	4.54E-01	553	1.57E-01	658	2.76E-01	382	4.95E-05	287	4.23E-01	825	5.63E-04	31	2.96E-01	883	4	1.32E-01	422	1.98E-01	756	5.22E-01	639	5.51E-04	639	1.56E-02	643	1.03E-01	480	6.79E-01	953	2	6	541.94
5810	NM_002856	PVRL2	5.44E-01	837	8.12E-03	145	1.88E-01	291	1.20E-01	612	3.10E-01	419	1.18E-04	325	6.66E-02	340	8.59E-01	1041	3.19E-02	624	3	1.11E-02	136	1.13E-01	659	9.43E-01	1090	3.12E-01	1025	9.47E-07	116	6.21E-01	916	8.32E-03	96	3	6	542.00
47	NM_001096	ACLY	3.78E-02	398	7.95E-01	1020	5.33E-01	658	1.25E-01	619	7.77E-01	931	5.51E-03	523	1.25E-01	463	1.49E-01	450	1.31E-03	381	3	4.15E-02	262	4.11E-02	535	8.25E-01	959	4.45E-08	54	6.67E-01	1066	3.87E-02	342	8.36E-04	17	5	8	542.38
51307	NM_001135647	FAM53C	3.21E-02	353	4.75E-01	813	3.11E-01	420	9.30E-02	580	4.08E-02	82	3.42E-01	948	7.73E-01	1037	2.43E-02	179	1.23E-01	768	3	6.86E-01	926	7.64E-02	603	2.76E-01	254	6.63E-06	320	3.84E-01	975	1.57E-03	110	4.93E-02	311	3	6	542.44
55226	NM_018227	UBA6	7.85E-02	510	2.94E-02	337	6.45E-01	784	3.17E-03	233	1.71E-01	234	6.19E-01	1055	2.20E-01	616	5.76E-03	84	4.28E-01	948	3	9.80E-01	1142	3.05E-04	114	6.92E-01	823	9.50E-06	340	6.93E-04	403	1.67E-01	552	1.36E-01	504	3	6	542.44
8826	NM_003870	IQGAP1	6.52E-01	883	4.55E-02	460	4.96E-01	606	2.92E-04	100	3.16E-01	427	2.76E-05	265	1.00E-01	421	1.18E-01	399	9.51E-02	738	3	2.61E-02	211	8.46E-01	1119	7.73E-01	906	1.33E-04	536	9.90E-03	596	8.24E-03	186	4.82E-01	827	4	7	542.50
9391	NM_004804	CIAO1	2.10E-01	614	4.71E-02	474	5.39E-01	664	1.12E-01	604	3.14E-01	424	1.53E-02	607	4.99E-02	270	1.18E-04	16	4.44E-07	104	5	7.27E-01	959	2.39E-01	806	4.29E-01	508	6.56E-02	929	6.62E-04	399	3.13E-01	694	2.05E-01	608	1	6	542.50
7153	NM_001067	TOP2A	9.54E-01	1108	4.38E-02	451	1.62E-01	263	9.63E-03	328	2.02E-01	270	2.58E-01	905	1.52E-01	504	1.66E-01	476	5.01E-09	48	3	5.06E-01	814	6.47E-01	1063	3.47E-01	375	4.47E-03	743	1.88E-09	22	1.04E-02	201	9.28E-01	1111	3	6	542.63
60488	NM_015423	AASDHPPT	8.21E-01	1005	7.83E-02	527	2.55E-02	98	6.04E-03	279	6.38E-01	795	4.89E-07	153	1.21E-01	454	9.19E-02	354	1.86E-04	276	4	8.21E-01	1032	3.68E-01	942	4.24E-01	499	6.95E-07	180	1.12E-03	431	1.95E-01	585	8.65E-01	1076	2	6	542.88
754	NM_004339	Pttg1ip	5.71E-01	846	7.24E-03	133	2.99E-01	404	1.39E-01	635	3.26E-01	440	3.86E-02	687	7.52E-01	1026	5.88E-01	849	1.25E-01	770	2	6.87E-02	322	1.96E-02	439	2.94E-01	281	1.41E-02	824	2.38E-01	930	1.38E-04	56	3.44E-03	45	4	6	542.94
7265	NM_003314	TTC1	2.32E-01	634	4.81E-02	484	1.50E-01	248	3.25E-03	234	6.48E-01	806	8.98E-03	553	2.42E-01	643	1.13E-03	39	3.63E-01	919	4	7.45E-01	976	8.76E-02	625	1.64E-01	119	1.50E-05	374	9.00E-01	1129	5.83E-01	896	3.51E-04	8	2	6	542.94
6583	NM_003074	SMARCC1	1.79E-04	24	2.58E-02	306	5.95E-01	731	8.22E-02	564	8.89E-01	1041	1.04E-08	78	1.98E-01	582	3.90E-02	234	1.15E-01	759	4	8.59E-01	1063	1.25E-01	676	6.70E-01	798	6.05E-03	758	8.82E-09	32	2.31E-01	621	9.18E-02	429	2	6	543.50
58190	NM_021204	ENOPH1	9.48E-01	1099	2.46E-02	291	5.01E-01	612	6.94E-03	297	4.82E-02	99	1.14E-05	225	1.85E-01	561	4.01E-01	719	6.12E-07	108	5	4.78E-01	799	4.84E-01	1002	5.13E-01	630	9.78E-05	515	2.88E-07	82	8.80E-01	1074	1.82E-01	583	2	7	543.50
8836	NM_003878	GGH	1.32E-02	201	5.42E-01	848	5.53E-02	166	7.55E-04	152	9.93E-01	1148	7.74E-01	1102	1.16E-01	447	1.81E-01	500	3.07E-02	619	3	1.10E-03	50	7.55E-02	601	3.31E-01	343	3.83E-01	1044	5.99E-07	98	4.28E-02	361	7.87E-01	1020	3	6	543.75
54502	NM_019027	RBM47	6.59E-03	139	9.08E-01	1099	4.69E-01	575	8.46E-05	75	8.18E-01	976	1.96E-06	183	2.13E-01	605	5.04E-02	271	6.08E-01	1020	3	2.81E-01	608	3.43E-03	276	9.63E-01	1116	5.05E-01	1074	5.26E-06	173	8.76E-02	455	4.80E-03	57	3	6	543.88
10146	NM_198395	G3BP1	2.80E-01	673	6.42E-02	516	7.67E-01	917	1.90E-04	86	6.39E-01	797	8.28E-07	166	2.10E-02	172	7.93E-02	330	3.53E-02	637	4	2.26E-01	535	1.55E-01	712	8.07E-01	944	8.08E-06	335	1.67E-01	894	7.45E-01	996	2.29E-04	4	2	6	544.63
3033	NM_000521	Hexb	9.98E-01	1156	3.74E-02	402	8.02E-01	960	3.57E-02	456	7.51E-02	136	4.29E-01	998	1.82E-01	556	1.13E-01	391	5.38E-02	682	2	7.56E-02	339	6.29E-05	58	5.20E-01	635	7.02E-06	323	1.10E-01	846	2.67E-01	656	1.22E-02	121	3	5	544.69
9601	NM_004911	Pdia4	1.56E-03	64	6.58E-01	922	9.51E-01	1122	7.10E-06	28	7.82E-01	938	4.26E-06	198	5.83E-02	308	7.24E-01	956	9.81E-08	75	4	1.83E-01	494	2.93E-02	491	9.27E-01	1065	7.34E-06	326	2.52E-02	693	1.58E-03	111	6.19E-01	924	4	8	544.69
50999	NM_001167830	Tmed5	6.74E-02	492	3.35E-01	731	2.12E-01	320	1.94E-01	685	5.99E-01	747	1.41E-02	593	7.53E-01	1027	3.83E-02	227	3.48E-06	144	3	8.48E-01	1053	8.64E-03	362	1.36E-01	86	1.82E-02	839	1.12E-05	202	1.73E-02	250	6.84E-01	958	4	7	544.75
7351	NM_003355	UCP2	5.57E-03	127	8.39E-01	1054	7.76E-01	929	1.07E-06	9	7.95E-01	953	7.24E-01	1093	3.73E-02	227	8.94E-03	106	9.80E-01	1152	4	9.81E-01	1143	1.47E-02	408	2.51E-01	212	1.03E-06	208	1.98E-08	42	8.24E-05	45	7.76E-01	1011	4	8	544.94
3654	NM_002198	Irf1	1.97E-02	249	2.42E-01	661	2.11E-01	318	9.51E-01	1131	4.80E-01	621	6.83E-04	406	7.46E-01	1023	1.62E-02	145	1.42E-01	781	3	4.16E-02	263	4.05E-02	533	8.28E-01	961	5.97E-06	312	1.92E-02	662	3.07E-03	134	1.45E-01	520	5	8	545.00
8705	NM_003782	B3GALT4	7.68E-02	505	1.14E-01	553	6.15E-03	46	3.86E-02	472	5.31E-01	661	3.45E-01	950	3.46E-01	751	5.33E-01	803	5.24E-06	156	3	8.11E-01	1025	1.27E-01	682	6.83E-01	811	5.94E-06	311	5.43E-04	374	2.78E-04	68	1.59E-01	547	3	6	545.00
9675	NM_014657	KIAA0406	7.95E-01	978	3.87E-02	411	1.91E-01	295	1.44E-01	645	4.06E-01	529	9.50E-06	222	2.79E-01	691	8.17E-01	1019	1.64E-04	273	3	8.89E-01	1081	2.20E-01	781	1.62E-01	114	3.07E-04	595	4.19E-04	365	2.73E-01	665	5.72E-03	64	3	6	545.50
1687	NM_000791	Dhfr	3.93E-03	102	4.12E-01	771	6.53E-01	794	4.33E-01	845	8.45E-01	997	3.11E-15	6	8.01E-03	101	3.14E-02	207	7.76E-01	1070	4	1.60E-01	464	2.08E-05	31	5.00E-01	612	6.14E-01	1096	1.40E-01	872	1.64E-01	547	2.69E-02	214	2	6	545.56
54892	NM_017801	CMTM6	2.22E-01	625	3.53E-01	737	2.14E-02	86	3.07E-02	432	8.65E-02	150	2.09E-01	873	3.22E-01	729	8.29E-02	339	9.05E-06	173	3	1.62E-01	465	2.10E-07	4	7.81E-01	913	6.21E-02	925	1.04E-04	308	8.89E-01	1078	5.87E-01	900	2	5	546.06
23788	NM_014342	mtch2	8.23E-01	1007	2.07E-02	263	5.61E-01	687	6.95E-03	298	2.61E-01	359	1.02E-02	563	2.02E-03	43	1.35E-02	129	2.51E-01	852	5	2.94E-01	621	5.60E-01	1033	5.98E-01	720	6.29E-06	316	7.37E-01	1091	2.13E-01	609	1.60E-02	147	2	7	546.13
22948	NM_012073	cct5	4.71E-01	800	2.48E-02	295	9.94E-03	60	5.06E-01	884	9.67E-01	1114	4.66E-01	1012	3.26E-01	732	4.49E-01	747	2.37E-03	415	3	6.06E-02	307	8.64E-01	1126	2.80E-01	260	3.92E-07	149	1.46E-04	322	5.59E-02	395	1.26E-02	124	3	6	546.38
4E+05	NM_203305	FAM102A	8.12E-01	994	6.41E-03	119	7.59E-01	910	1.95E-01	686	9.95E-02	165	2.33E-01	886	1.59E-01	516	7.43E-01	964	5.37E-03	479	2	1.78E-03	58	6.10E-03	327	2.75E-01	253	3.61E-05	444	8.33E-07	108	5.28E-01	854	7.21E-01	980	4	6	546.44
6876	NM_005642	taf7	7.33E-01	929	1.73E-01	613	1.95E-01	300	2.92E-01	760	1.25E-01	192	6.68E-02	738	7.55E-01	1029	5.42E-01	812	3.27E-03	439	1	2.48E-01	567	2.43E-01	810	3.01E-01	294	6.93E-02	934	1.09E-11	5	4.82E-04	76	3.33E-02	248	3	4	546.63
22848	NM_014911	aak1	1.12E-01	548	4.16E-02	433	9.25E-01	1092	7.38E-01	1011	3.13E-01	423	1.51E-02	605	3.46E-02	220	8.08E-02	333	2.44E-01	848	3	3.53E-01	684	4.19E-03	299	3.28E-01	335	2.06E-02	848	4.80E-03	531	5.54E-04	81	1.06E-01	458	4	7	546.81
27339	NM_014502	prpf19	7.29E-01	924	2.94E-02	339	1.94E-01	299	5.13E-02	515	5.74E-01	717	1.24E-01	812	2.27E-02	176	8.74E-01	1051	3.40E-01	905	2	3.24E-02	234	6.95E-03	339	4.29E-01	507	3.00E-07	135	5.30E-01	1032	1.12E-02	203	1.71E-01	567	4	6	547.19
64783	NM_001126130	gins3	3.65E-01	740	3.39E-02	376	4.17E-01	514	5.70E-01	932	5.18E-02	104	1.88E-04	345	1.94E-02	158	5.43E-01	813	5.02E-12	7	4	6.65E-02	318	3.11E-01	896	4.43E-01	530	8.19E-07	194	1.01E-01	833	9.45E-01	1122	5.55E-01	875	1	5	547.31
51495	NM_016395	ptplad1	8.72E-01	1036	2.25E-03	65	7.67E-01	918	4.30E-03	249	4.01E-01	519	1.00E-01	788	1.52E-02	133	9.95E-01	1154	2.70E-06	136	4	5.03E-01	811	3.89E-02	528	3.82E-01	432	1.50E-05	373	3.01E-05	235	5.48E-01	870	1.40E-01	517	3	7	547.75
55256	NM_018270	c20orf20	2.04E-02	257	6.83E-01	939	8.75E-01	1041	7.94E-01	1046	5.80E-01	725	8.19E-03	546	5.73E-03	83	2.85E-01	603	6.71E-01	1037	3	1.78E-04	12	1.88E-02	436	8.64E-01	990	1.29E-05	368	5.50E-05	277	3.52E-02	327	7.19E-03	78	6	9	547.81
23479	NM_213595	iscU	4.64E-03	111	1.60E-03	54	2.24E-01	329	1.68E-01	673	5.46E-01	682	1.97E-06	184	6.67E-01	982	9.29E-01	1097	8.44E-01	1095	3	8.20E-02	352	2.74E-03	256	1.57E-01	106	3.62E-03	735	2.70E-02	702	8.67E-02	453	6.80E-01	955	3	6	547.88
3597	NM_153464	ilf3	1.29E-01	562	1.91E-01	626	4.18E-01	517	4.46E-01	853	4.08E-01	532	8.79E-02	774	2.39E-03	51	2.89E-01	609	5.94E-01	1016	1	4.43E-02	273	4.37E-02	543	4.05E-01	467	1.26E-04	531	4.48E-03	527	1.44E-02	223	2.64E-01	663	5	6	547.94
55689	NM_018090	NECAP2	2.98E-02	337	1.87E-01	625	5.77E-02	170	6.49E-01	968	6.20E-01	775	3.96E-01	979	8.48E-02	390	3.02E-01	619	3.23E-02	626	2	3.12E-03	77	5.21E-04	148	7.04E-01	833	8.71E-05	510	1.46E-07	68	9.81E-01	1153	1.35E-01	502	4	6	548.75
55163	NM_018130	Shq1	9.23E-01	1071	8.79E-03	150	3.84E-02	132	2.82E-02	421	4.34E-01	564	6.15E-01	1054	9.58E-01	1138	1.41E-01	437	3.18E-01	894	3	1.90E-01	504	4.48E-03	304	3.45E-01	366	8.94E-09	24	5.09E-03	541	3.30E-01	711	1.17E-01	476	3	6	549.19
306	NM_005139	anxa3	9.01E-01	1060	2.32E-01	653	4.42E-02	148	1.19E-01	610	7.42E-01	896	1.22E-01	811	1.68E-02	140	2.76E-01	599	3.12E-01	890	2	2.37E-01	552	1.86E-01	741	5.06E-01	623	2.65E-07	128	3.92E-04	362	5.95E-02	406	2.03E-02	173	3	5	549.50
8882	NM_003904	ZNF259	7.46E-01	945	1.44E-02	205	2.13E-01	322	9.32E-01	1123	8.26E-01	982	1.23E-03	437	1.48E-01	495	8.96E-03	107	2.69E-03	425	4	3.79E-02	255	3.62E-03	282	8.60E-01	986	3.62E-07	143	1.53E-01	882	9.61E-03	194	7.72E-01	1009	4	8	549.50

10298	NM_001014834	PAK4	4.45E-03	107	7.67E-01	999	3.48E-01	454	1.63E-05	42	4.90E-01	632	3.57E-04	378	9.30E-01	1121	1.27E-01	410	3.08E-01	888	3	1.19E-01	409	2.37E-01	803	4.62E-01	566	1.23E-05	365	2.63E-01	938	1.60E-01	544	1.42E-02	137	2	5	549.56
1105	NM_001281	TBCB	6.42E-01	875	5.98E-01	878	4.03E-02	135	2.44E-03	210	6.26E-01	781	7.65E-01	1099	3.97E-01	807	3.90E-02	233	2.14E-02	583	4	6.91E-01	932	1.16E-03	197	9.54E-02	49	1.66E-05	386	2.87E-03	490	5.44E-01	867	3.91E-02	272	4	8	549.63
55357	NM_018518	Mcm10	1.75E-02	230	6.74E-01	930	8.54E-01	1019	4.82E-01	874	6.99E-01	864	9.67E-02	786	4.12E-02	242	7.00E-02	312	1.97E-03	407	3	2.78E-01	602	3.25E-01	908	4.73E-02	29	2.97E-02	872	1.03E-02	603	2.90E-04	70	3.92E-03	49	5	8	549.81
11257	NR_015381.1	TP53TG1	4.53E-01	788	9.60E-01	1130	8.34E-01	998	4.36E-02	488	4.36E-02	91	3.17E-07	143	1.53E-02	134	6.37E-01	883	1.31E-01	773	4	3.50E-01	678	9.60E-04	181	8.40E-01	970	2.11E-06	252	3.62E-07	88	4.70E-02	369	4.93E-01	834	4	8	550.00
4436	NM_002439	msh3	8.94E-01	1056	1.62E-02	225	3.07E-01	415	5.91E-01	940	7.95E-02	141	7.06E-01	1085	3.81E-02	229	3.70E-01	683	3.58E-10	23	3	1.36E-01	431	2.90E-02	488	6.00E-01	723	1.24E-07	103	2.39E-04	344	7.72E-01	1010	5.97E-01	907	3	6	550.19
26578	NM_012383	ostf1	5.41E-01	835	3.55E-01	740	2.95E-04	7	1.20E-01	613	9.27E-04	4	6.91E-01	1080	5.06E-02	274	2.09E-01	532	5.21E-02	676	2	1.79E-01	488	2.07E-01	768	6.25E-01	750	1.44E-06	232	4.00E-01	980	3.20E-04	72	3.80E-01	755	2	4	550.38
55030	NM_017952	Ptcd3	3.90E-01	754	1.50E-02	212	8.15E-01	973	4.03E-02	478	4.83E-01	624	8.91E-02	776	1.10E-01	436	4.77E-02	266	8.66E-01	1105	3	1.32E-01	424	1.54E-04	91	6.87E-01	815	4.54E-07	156	1.89E-02	660	4.16E-01	780	4.04E-02	273	4	7	551.44
55003	NM_017914	C19orf24	9.94E-01	1146	3.57E-02	387	4.88E-01	600	7.81E-02	556	2.66E-01	365	5.82E-03	526	1.40E-01	486	9.98E-03	110	9.51E-01	1138	3	1.54E-01	455	2.53E-02	473	4.09E-01	474	1.19E-07	99	4.22E-05	257	9.68E-01	1140	2.12E-01	615	3	6	551.69
4548	NM_002454	MTRR	5.13E-01	824	6.48E-01	914	5.66E-03	42	9.00E-02	577	3.98E-01	514	4.47E-05	281	3.95E-01	805	5.98E-01	857	4.28E-03	463	3	7.83E-02	346	4.40E-04	141	7.95E-01	931	8.18E-04	657	1.61E-03	453	2.54E-01	646	6.77E-02	377	3	6	551.75
29796	NM_013387	Uqcr10	9.64E-01	1119	4.76E-02	479	1.57E-01	258	2.57E-02	409	8.04E-03	19	6.36E-01	1059	5.36E-01	912	6.97E-01	934	1.33E-04	264	4	7.27E-02	335	9.81E-04	185	3.29E-01	340	3.20E-03	726	9.12E-01	1135	2.36E-02	286	6.45E-02	368	3	7	551.75
80273	NM_025214	CCDC68	6.17E-03	135	1.72E-01	608	4.10E-01	508	3.98E-01	828	2.23E-01	300	4.11E-02	694	2.14E-01	609	1.05E-01	375	5.27E-01	989	2	3.90E-01	721	3.16E-04	116	7.93E-01	927	1.85E-06	240	5.21E-01	1031	1.69E-03	113	2.29E-01	634	3	5	551.75
26589	NM_022163	mrpl46	9.69E-01	1125	1.65E-02	229	3.23E-02	115	5.60E-01	919	8.07E-03	20	1.19E-02	578	8.52E-02	391	6.58E-01	896	1.27E-02	552	5	1.18E-01	408	8.78E-01	1132	1.62E-01	113	9.08E-08	85	6.75E-04	401	5.19E-01	847	7.84E-01	1018	2	7	551.81
55756	NM_001136223	Rcor3	2.48E-01	645	1.21E-02	188	2.90E-01	390	2.67E-01	746	5.62E-01	703	5.93E-05	295	7.14E-01	1006	5.34E-04	29	8.54E-03	510	4	3.05E-01	637	2.43E-02	465	2.57E-01	222	8.62E-02	948	2.90E-02	713	3.72E-01	747	1.87E-01	586	2	6	551.88
10243	NM_001024218	GPHN	6.82E-02	494	3.93E-02	415	1.86E-01	288	2.73E-01	751	5.13E-01	650	1.10E-01	802	5.46E-02	289	6.56E-01	893	2.51E-03	419	2	8.24E-02	354	3.38E-02	512	4.49E-01	541	7.35E-01	1125	1.78E-01	899	5.40E-04	80	5.21E-02	329	2	4	552.56
9804	NM_014765	TOMM20	9.18E-03	165	4.76E-02	477	6.70E-02	181	1.50E-02	361	8.21E-01	978	6.84E-05	303	4.97E-01	877	6.92E-01	927	1.04E-01	749	4	6.23E-02	312	1.88E-02	434	8.53E-01	981	5.09E-05	473	3.79E-06	165	1.49E-01	527	6.53E-01	936	3	7	552.88
55750	NM_001145159	Ints9	4.61E-01	793	2.54E-02	302	9.27E-01	1094	2.21E-02	396	9.77E-01	1130	1.92E-03	460	2.94E-01	705	7.20E-03	92	2.44E-05	197	5	1.26E-01	414	1.87E-02	433	8.30E-01	964	8.68E-08	81	6.62E-01	1063	4.00E-02	346	6.75E-02	376	3	8	552.88
6428	NM_006925	Sfrs5	4.54E-01	789	1.20E-01	562	4.66E-01	571	6.83E-03	296	2.61E-02	50	7.19E-01	1091	7.70E-01	1035	3.13E-01	633	7.40E-03	500	3	7.16E-02	330	6.68E-02	586	6.26E-01	752	5.31E-08	59	7.61E-06	186	2.99E-02	307	9.04E-01	1104	3	6	553.19
4276	NM_005931	micB	4.56E-02	447	5.32E-01	842	7.54E-01	903	6.59E-02	539	6.14E-01	765	1.81E-05	247	1.66E-01	528	9.61E-05	15	1.82E-03	404	4	4.60E-01	777	3.63E-01	936	5.93E-01	717	7.27E-09	21	1.67E-04	329	3.04E-01	685	3.10E-01	698	2	6	553.31
701	NM_001211	BUB1B	8.74E-01	1038	1.16E-01	555	3.63E-02	127	5.31E-02	519	6.26E-02	120	2.97E-03	490	7.57E-02	364	6.32E-01	879	3.26E-09	41	3	8.40E-01	1046	2.71E-02	483	3.62E-01	398	2.45E-01	1001	9.83E-08	63	4.88E-01	831	5.87E-01	899	2	5	553.38
9837	NM_021067	Gins1	8.58E-01	1023	1.34E-02	200	7.32E-01	883	4.19E-02	484	2.31E-01	313	1.82E-01	855	2.41E-02	181	2.92E-01	612	5.03E-07	107	4	5.93E-01	876	2.19E-03	236	5.67E-01	693	4.81E-05	467	3.71E-05	249	5.39E-01	862	4.64E-01	813	3	7	553.38
79697	NM_024775	GEMIN6	1.81E-02	236	4.75E-03	98	7.66E-01	916	2.81E-03	226	4.76E-01	610	3.24E-03	496	5.01E-01	880	1.74E-01	487	2.25E-02	588	5	7.56E-01	988	2.48E-02	468	3.85E-01	439	1.79E-04	559	6.59E-05	287	3.02E-01	683	5.82E-01	895	3	8	553.50
2872	NM_201397	GPX1	2.75E-01	668	5.81E-01	868	2.09E-02	82	2.49E-02	406	2.57E-02	49	2.21E-01	880	5.14E-01	891	4.01E-02	240	1.79E-06	121	5	1.77E-01	486	3.51E-01	927	9.44E-01	1091	5.50E-07	161	3.60E-05	248	7.02E-01	969	4.03E-01	772	2	7	553.69
25797	NM_012413	QPCT	1.68E-02	224	5.36E-01	846	6.20E-01	756	3.30E-01	789	5.67E-01	711	1.41E-02	592	3.94E-02	233	3.40E-01	656	1.14E-03	374	4	2.77E-01	600	4.44E-02	545	4.60E-01	562	2.82E-06	266	4.02E-03	515	4.58E-01	814	6.97E-02	380	3	7	553.94
3725	NM_002229	junb	2.11E-02	268	4.22E-02	435	3.43E-01	447	3.81E-02	469	1.73E-01	237	1.27E-03	439	2.19E-01	613	9.13E-01	1084	1.84E-09	34	5	2.45E-05	6	2.64E-01	836	5.58E-01	678	4.74E-05	465	1.53E-01	884	8.71E-01	1065	6.04E-01	912	2	7	554.50
55178	NM_018155	SLC25A36	4.49E-01	785	1.92E-02	251	1.55E-01	253	1.33E-03	184	1.40E-01	202	3.41E-01	947	1.53E-01	508	1.37E-02	132	5.94E-01	1015	3	4.22E-02	267	8.48E-02	620	5.42E-01	660	9.72E-03	795	1.15E-02	612	1.96E-01	588	8.43E-01	1057	3	6	554.75
9140	NM_004707	Atg12	8.20E-02	514	8.37E-01	1052	3.36E-02	120	8.91E-03	318	7.01E-01	868	3.05E-05	272	1.28E-01	467	6.64E-01	901	2.13E-03	412	4	7.53E-02	338	1.49E-01	709	1.65E-01	120	7.92E-05	503	1.20E-04	318	5.02E-01	837	9.52E-01	1128	2	6	554.81
8760	NM_003818	Cds2	1.23E-02	194	4.24E-01	781	4.97E-01	607	2.23E-04	93	3.99E-01	517	1.38E-03	443	4.78E-01	866	8.91E-01	1070	3.67E-05	212	4	7.63E-01	992	8.49E-04	176	3.40E-01	357	6.27E-02	927	1.68E-02	648	3.77E-01	752	3.55E-02	253	3	7	555.50
10171	NM_005772	RCL1	8.49E-01	1017	2.85E-02	329	6.50E-01	789	3.23E-02	440	2.05E-01	273	4.33E-02	701	1.23E-01	457	3.60E-01	676	4.48E-01	958	3	8.70E-02	362	2.05E-02	442	3.28E-01	334	3.36E-05	442	1.18E-01	857	1.52E-01	530	4.18E-02	281	3	6	555.50
84722	NM_032704	TUBA1C	8.40E-01	1014	4.47E-02	454	4.86E-01	595	5.84E-04	138	3.40E-01	457	1.78E-06	182	7.44E-01	1021	7.92E-02	329	6.43E-04	330	4	2.36E-01	550	2.46E-02	466	4.66E-01	571	1.13E-02	808	6.64E-04	400	4.61E-01	816	3.82E-01	759	3	7	555.63
55556	NM_017593	bmp2k	3.19E-02	351	4.00E-01	766	5.17E-01	634	6.87E-01	987	7.83E-01	941	3.63E-01	963	5.15E-02	278	2.38E-01	565	5.74E-03	486	2	1.30E-01	419	6.79E-02	590	4.07E-02	25	3.93E-06	289	6.45E-06	181	3.49E-01	730	2.90E-01	689	3	5	555.88
2731	NM_006708	GLO1	9.56E-01	1110	3.38E-03	81	4.33E-01	535	8.23E-01	1064	3.13E-01	422	5.22E-11	33	6.10E-01	952	6.67E-05	11	8.39E-01	1094	3	2.80E-01	606	7.32E-05	63	1.45E-01	94	2.32E-01	998	4.57E-05	265	3.35E-01	716	5.18E-01	853	2	5	556.06
4839	NM_000270	pnp	3.34E-01	723	2.82E-02	327	4.81E-02	157	1.77E-02	374	9.63E-01	1111	7.95E-02	756	1.99E-02	160	8.26E-01	1024	9.58E-01	1140	4	3.17E-01	647	5.15E-03	316	6.91E-01	822	1.28E-08	26	1.18E-01	856	7.69E-02	440	1.39E-03	22	3	7	556.31
1211	NM_001295	CCR1	2.96E-02	336	1.20E-01	563	1.12E-03	19	5.68E-01	929	4.42E-02	92	5.80E-02	727	6.41E-03	88	9.88E-01	1147	3.55E-01	914	4	3.04E-01	634	1.02E-01	646	2.01E-01	159	2.23E-04	574	2.34E-03	472	3.24E-01	706	5.82E-01	896	2	6	556.38
8543	NM_006769	Lmo4	8.34E-01	1012	7.29E-01	972	4.12E-01	510	5.72E-01	934	3.17E-01	428	2.14E-06	186	3.50E-01	761	6.99E-01	936	8.46E-02	729	1	5.05E-01	812	4.43E-03	303	8.97E-02	43	1.63E-07	109	2.44E-03	477	4.10E-02	354	5.41E-02	341	4	5	556.69
55037	NM_017975	ZWILCH	2.94E-01	691	6.61E-03	124	2.80E-01	377	7.84E-01	1039	4.16E-01	540	1.25E-04	328	3.06E-02	205	3.97E-01	712	4.72E-06	150	4	8.44E-01	1050	2.88E-01	866	5.38E-01	655	1.31E-06	226	1.02E-01	835	8.24E-01	1040	7.55E-03	83	2	6	557.56
6879	NM_134323	TARBP2	4.16E-02	421	9.94E-01	1155	3.32E-01	437	8.77E-02	573	2.99E-01	409	9.12E-01	1138	1.58E-03	39	3.93E-02	236	3.30E-06	142	4	9.32E-01	1110	3.64E-03	286	7.67E-01	899	3.59E-01	1037	1.41E-01	873	2.22E-03	121	3.50E-03	46	3	7	557.63
5387	NM_006232	POLR2H	1.34E-01	563	9.56E-01	1127	2.13E-01	321	6.38E-03	286	9.19E-01	1065	9.31E-08	117	1.55E-01	509	3.44E-01	658	1.43E-02	563	3	2.12E-01	523	2.93E-02	492	3.64E-01	401	2.23E-04	575	1.28E-01	865	2.36E-01	626	3.02E-02	232	3	6	557.69
26275	NM_198047	HIBCH	4.97E-01	815	2.56E-04	18	4.90E-02	159	1.72E-02	370	4.40E-01	575	5.08E-01	1023	1.64E-01	525	2.24E-01	552	4.34E-01	950	3	9.28E-02	369	1.83E-02	431	3.88E-01	445	2.30E-03	711	5.98E-02	794	3.31E-01	712	1.17E-01	474	2	5	557.69
50486	NM_015714	G0s2	1.18E-02	188	9.92E-01	1152	6.11E-01	754	7.03E-05	72	1.65E-01	229	5.62E-01	1041	2.78E-03	56	2.26E-01	555	7.38E-01	1058	3	3.59E-02	245	3.36E-01	916	3.54E-01	385	2.63E-07	127	1.00E-03	425	2.78E-01	669	8.42E-01	1054	3	6	557.88
10926	NM_006716	Dbf4	2.81E-01	676	3.20E-02	363	3.23E-01	429	1.40E-02	358	2.84E-01	386	1.59E-07	125	6.38E-01	967	2.63E-01	586	1.20E-06	115	4	9.00E-01	1084	2.88E-03	261	3.74E-01	416	3.08E-03	723	5.86E-03	555	7.50E-01	1000	5.68E-01	887	3	7	558.19
57102	NM_020401	NUP107	9.97E-01	1152	5.00E-03	104	9.48E-01	1118	2.46E-01	729	3.26E-01	441	5.88E-01	1046	1.55E-01	510	1.68E-01	478	2.42E-04	286	2	5.74E-02	298	1.69E-03	223	5.41E-01	658	2.68E-06	262	6.29E-05	283	1.45E-01	526	4.69E-01	817	3	5	558.19
29015	NM_199329	Slc43a3	2.70E-03	83	6.32E-02	515	2.82E-01	380	2.31E-04	96	4.32E-01	562	1.03E-02	565	1.66E-02	139	9.06E-01	1080	1.41E-01	778	4	8.27E-01	1040	1.17E-03	198	2.98E-01	287	5.50E-01	1083	1.18E-01	858	1.78E-01	569	3.19E-01	708	1	5	558.81
60672	NM_021947	srr	2.97E-01	696	2.93E-01	698	3.26E-02	117	1.02E-03	174	2.15E-01	290	1.60E-05	239	9.42E-01	1126	1.61E-01	467	8.88E-01	1110	3	4.65E-01	783	1.24E-02	389	2.96E-01	284	4.48E-04	623	2.78E-03	486	4.33E-01	794	2.65E-01	666	3	6	558.88
8562	NM_003677	DENR	5.19E-01	827	2.45E-02	288	8.78E-01	1049	5.10E-02	511	2.98E-01	406	8.63E-06	219	3.61E-01	772	8.85E-02	347	6.53E-05	236	3	8.04E-01	1016	3.52E-02	514	6.30E-01	756	6.51E-08	67	7.90E-01	1104	4.51E-01	805	1.86E-03	26	3	6	558.94
51094	NM_015999	adipor1	1.40E-02	208	4.18E-01	777	5.15E-01	633	5.21E-01	900	1.32E-01	198	1.88E-02	625	2.99E-03	57	9.34E-06	6	5.84E-01	1010	4	5.15E-01	824	1.65E-01	718	9.43E-01	1088	1.75E-07	111	1.32E-02	624	7.05E-02	426	3.62E-01	741	2	6	559.13
56947	NM_020214	parp6	1.75E-02	228	8.85E-01	1086	1.27E-01	229	5.70E-01	931	7.83E-01	940	2.04E-05	255	1.10E-01	437	3.40E-02	216	2.94E-01	882	3	7.08E-01	943	1.89E-01	747	1.46E-01	97	6.36E-08	66	2.40E-02	687	5.82E-02	404	4.38E-01	798	2	5	559.13
9662	NM_025009	CEP135	9.49E-01	1101	7.49E-01	991	2.07E-02	79	1.40E-01	638	6.22E-01	778	2.75E-02	651	3.86E-01	796	1.12E-01	390	4.76E-05	224	3	9.69E-01	1136	2.83E-03	257	1.46E-01	96	5.91E-02	922	1.80E-06	134	3.54E-02	331	9.67E-02	441	3	6	560.31
467	NM_001674	atf3	6.37E-01	871	1.65E-02	230	7.21E-01	872	3.43E-01	799	2.00E-01	264	1.90E-01	862	2.23E-03	48	3.52E-01	671	6.18E-01	1025	2	2.17E-02	189	1.79E-02	428	3.12E-01	312	6.28E-01	1099	9.37E-07	115	2.98E-03	131	8.38E-01	1050	4	6	560.38
9793	NM_001008938	ckap5	2.53E-02	310	6.72E-02	520	6.29E-01	764	8.93E-02	576	6.26E-01	780	1.57E-02	610	1.84E-02	152	2.29E-02	173	8.63E-01	1102	4	1.65E-01	470	6.03E-04	155	6.10E-01	738	3.03E-01	1022	2.02E-04	336	1.55E-01	534	3.40E-01	724	2	6	560.38
3184	NM_012207	Hnrnph3	7.35E-01	932	5.54E-02	507	7.94E-01	949	2.19E-05	50	2.21E-01	298	4.44E-16	4	1.82E-01	555	7.51E-01	971	1.72E-01	806	2	1.47E-01	443	1.09E-01	655	3.70E-01	410	2.24E-05	408	2.10E-01	914	3.46E-02	324	3.64E-01	744	2	4	560.63
51014	NM_021649	Ticam2	7.16E-01	917	8.66E-01	1070	2.25E-01	332	4.35E-03	250	2.00E-01	263	3.57E-02	676	2.65E-01	675	1.72E-01	486	4.75E-04	318	3	4.85E-04	29	6.33E-01	1058	8.20E-01	957	1.97E-02	846	7.83E-04	410	2.26E-02	277	8.20E-02	407	4	7	560.69
2580	NM_020474	GALNT1	4.86E-02	462	2.48E-01	668	6.48E-01	787	2.49E-03	213	5.43E-01	681	7.48E-08	113	2.11E-01	599	1.79E-01	494	5.51E-02	686	3	9.68E-01	1134	4.11E-04	136	6.49E-01	778	5.80E-03	757	2.12E-04	339	3.94E-01	767	5.92E-02	358	3	6	560.75
10528	NM_006392	Nop56	8.72E-02	518	6.70E-04	33	3.30E-02	119	5.17E-03	268	9.43E-01	1091	9.26E-01	1141	9.50E-01	1130	4.75E-01	761	2.67E-02	605	4	5.73E-01	863	1.30E-01	685	4.13E-01	482	7.36E-10	6	2.56E-01	935	1.44E-03	106	3.20E-02	240	3	7	561.44
29074	NM_014161	MRPL18	7.34E-01	931	1.62E-03	55	9.64E-01	1130	1.39E-02	357	2.03E-01	271	1.51E-07	122	1.62E-01	522	3.95E-01	708	3.50E-01	912	3	7.58E-02	341	1.65E-01	719	6.09E-01	735	5.99E-04	643	2.24E-04	341	9.28E-02	465	3.54E-01	735	2	5	561.69
826	NM_001003962	capns1	3.27E-01	718	3.68E-02	395	5.42E-01	666	3.88E-04	118	9.27E-02	154	7.49E-02	753	4.48E-01	847	7.59E-01	976	7.10E-03	497	3	8.26E-02	356	5.57E-01	1030	6.35E-01	761	4.79E-06	296	4.52E-05	263	7.12E-01	974	2.20E-02	185	3	6	561.81
79077	NM_024099	c11orf48	4.69E-01	798	2.52E-02	300	6.77E-01	827	7.53E-02	549	5.75E-01	720	5.69E-01	1043	7.31E-01	1011	2.45E-01	573	2.86E-04	295	2	9.33E-02	371	1.35E-01	694	4.43E-01	531	3.71E-09	15	5.10E-04	375	2.14E-01	611	4.18E-02	280	3	5	562.06
25821	NM_133645	Mto1	7.06E-01	913	1.58E-01	593	3.35E-03	27	4.72E-01	868	1.43E-02	26	2.73E-03	480	5.27E-01	906	1.40E-01	435	2.95E-04	296	4	9.15E-01	1096	6.88E-04	165	8.73E-02	41	5.90E-01	1094	3.57E-02	733	5.25E-01	852	1.17E-01	475	2	6	562.50
10749	NM_006612	Kif1c	1.27E-02	198	6.76E-01	931	5.82E-01	714	1.34E-02	354	9.89E-01	1142	1.09E-03	432	2.85E-02	196	6.82E-01	919	1.46E-12	6	5	1.57E-01	460	1.06E-01	652	1.10E-01	59	2.81E-02	867	7.18E-01	1083	7.15E-01	976	7.73E-04	16	2	7	562.81
3189	NM_002140	Hnrnpk	7.88E-01	973	2.29E-02	277	7.13E-01	864	4.84E-03	263	2.04E-01	272	1.70E-05	245	2.55E-01	662	3.03E-01	621	1.70E-04	275	4	1.84E-01	496	1.54E-01	711	4.09E-01	473	5.49E-01	1081	4.88E-02	773	5.52E-05	37	7.26E-01	983	2	6	562.88
1906	NM_004427	PHC2	2.97E-01	695	6.82E-03	126	1.91E-01	294	4.40E-01	850	7.61E-01	912	1.45E-01	832	3.87E-05	3	4.74E-01	760	2.49E-05	198	3	7.49E-01	978	5.02E-01	1012	1.36E-01	85	2.99E-04	594	1.47E-02	633	3.30E-02	318	3.32E-01	717	3	6	562.94
2876	NM_001018075	NR3C1	4.92E-01	809	4.94E-02	492	5.31E-01	653	8.07E-01	1057	7.03E-01	874	1.53E-05	237	8.92E-01	1094	1.30E-04	17	2.59E-03	421	4	1.63E-01	466	3.76E-01	947	4.24E-01	497	1.16E-05	360	3.00E-03	496	1.38E-05	24	1.71E-01	566	3	7	563.13
84790	NM_033386	MICALL1	8.09E-03	155	2.27E-01	647	3.64E-01	468	2.01E-03	200	9.35E-01	1084	3.88E-05	278	5.39E-01	913	2.21E-01	548	5.85E-01	1011	3	1.47E-02	154	5.28E-01	1022	5.77E-01	701	9.58E-06	341	1.43E-06	128	5.75E-01	888	1.18E-01	480	3	6	563.63
56965	NM_020317	C1orf63	8.74E-03	161	6.96E-02	521	2.40E-01	343	9.87E-04	172	7.57E-01	909	1.13E-02	574	4.65E-01	859	2.86E-01	605	2.31E-02	592	4	2.91E-01	616	8.06E-01	1110	3.47E-01	372	6.35E-04	650	1.33E-02	625	3.16E-02	314	2.01E-01	602	3	7	564.06
100	NM_000022	ada	7.70E-03	151	2.62E-02	310	9.38E-01	1106	2.98E-04	101	3.08E-01	417	2.55E-03	478	1.86E-01	563	8.46E-01	1030	3.03E-03	434	5	1.53E-01	450	1.44E-03	213	7.45E-01	871	6.15E-01	1097	3.53E-02	731	1.47E-02	227	5.13E-01	849	3	8	564.25
11329	NM_007271	stk38	2.08E-02	263	4.97E-01	823	7.88E-01	940	9.78E-02	587	8.76E-01	1027	1.53E-02	608	2.07E-02	168	2.92E-02	201	8.79E-02	733	4	2.43E-01	560	2.28E-01	794	5.95E-01	718	3.77E-05	449	6.76E-05	288	3.88E-01	763	9.66E-03	106	3	7	564.25
22845	NM_014908	DOLK	8.17E-01	1000	4.19E-01	778	4.47E-02	150	2.60E-02	411	2.37E-01	324	3.08E-03	493	3.50E-01	758	4.60E-01	754	9.90E-01	1156	3	1.04E-02	132	7.20E-02	595	3.25E-01	330	2.01E-06	250	2.02E-01	909	3.12E-01	693	4.66E-02	296	3	6	564.31
51762	NM_016530	RAB8B	5.55E-03	126	1.56E-03	52	8.77E-01	1047	6.80E-01	984	5.66E-01	709	2.77E-01	917	6.61E-02	338	3.71E-01	685	1.14E-02	540	3	1.77E-01	487	1.90E-01	748	3.19E-01	321	1.07E-02	804	2.59E-05	226	3.72E-01	749	4.71E-02	299	3	6	564.50
3074	NM_139009	HFE	7.88E-03	152	5.34E-01	844	8.21E-01	982	3.16E-04	103	1.91E-01	260	9.38E-05	319	5.48E-02	291	5.85E-01	846	1.17E-03	379	4	5.83E-02	302	6.81E-01	1069	8.50E-01	979	3.33E-04	598	7.67E-06	187	2.34E-01	622	9.01E-01	1100	2	6	564.56
55667	NM_018023	YEATS2	7.69E-02	506	2.86E-02	330	2.48E-01	350	9.55E-01	1135	5.91E-01	740	5.91E-04	396	1.77E-02	146	2.28E-01	556	2.93E-01	881	3	9.93E-02	380	8.59E-01	1123	6.42E-01	773	1.52E-05	378	4.65E-04	372	3.83E-01	756	2.67E-02	211	3	6	564.56
27292	NM_014473	Dimt1l	2.21E-01	624	1.03E-01	544	6.40E-01	778	1.14E-01	608	3.37E-01	453	8.25E-01	1112	3.40E-01	742	5.98E-01	856	6.67E-06	165	1	1.43E-05	5	2.06E-01	767	9.59E-01	1113	7.27E-07	184	3.15E-08	49	7.48E-02	435	1.99E-01	600	3	4	564.69
4739	NM_000434	Neu1	2.97E-01	697	4.95E-04	28	2.08E-01	312	9.09E-01	1107	3.11E-01	420	3.11E-01	934	7.33E-06	1	9.50E-01	1115	4.65E-02	667	3	4.69E-01	787	8.63E-03	360	2.50E-01	211	8.98E-05	512	2.43E-04	346	6.57E-01	942	1.98E-01	597	3	6	564.75
26471	NM_012385	nupr1	2.37E-01	639	1.83E-01	622	4.98E-02	161	2.71E-01	749	7.42E-01	897	2.07E-07	133	7.66E-01	1031	1.97E-01	520	2.52E-04	289	3	6.76E-01	919	2.57E-01	825	5.67E-01	692	1.56E-06	233	4.10E-07	90	8.35E-02	446	4.47E-01	803	2	5	565.56
9552	NM_004890	SPAG7	1.22E-02	191	3.33E-01	729	3.40E-01	445	2.77E-02	419	2.70E-01	371	8.60E-03	550	9.16E-01	1116	2.18E-01	544	3.50E-05	209	4	6.66E-01	913	2.04E-01	765	5.72E-01	697	3.02E-03	722	3.20E-07	85	4.54E-02	368	6.33E-01	929	3	7	565.81
25983	NM_015514	ngdn	4.96E-02	470	3.19E-01	717	9.28E-01	1096	3.86E-01	821	1.16E-01	183	6.52E-05	298	2.86E-01	698	1.43E-01	441	3.36E-02	631	3	2.33E-02	196	1.10E-02	380	8.31E-01	965	5.00E-05	470	7.32E-01	1088	6.66E-02	418	2.24E-02	188	4	7	566.25
55257	NM_018285	IMP3	2.08E-02	264	2.98E-01	702	2.80E-01	378	1.59E-01	659	6.80E-01	841	2.39E-10	42	9.23E-01	1118	1.64E-01	472	6.28E-02	697	2	3.25E-01	650	3.51E-02	513	6.51E-01	779	5.44E-01	1079	4.28E-06	168	6.76E-03	175	1.49E-01	524	3	5	566.31
5360	NM_016265	ZNF12	9.44E-01	1095	2.40E-03	68	5.27E-01	648	8.23E-02	565	2.88E-01	395	9.73E-05	321	2.30E-01	625	2.22E-09	1	1.16E-03	378	4	6.64E-01	912	2.49E-01	818	6.35E-01	763	2.59E-03	713	7.86E-05	295	7.24E-02	429	8.28E-01	1039	2	6	566.56
9329	NM_012204	GTF3C4	1.64E-01	583	6.61E-03	123	8.18E-01	980	8.43E-02	567	7.72E-01	925	4.04E-01	984	2.05E-02	167	5.01E-01	780	3.83E-04	304	3	5.88E-02	304	7.90E-01	1100	3.65E-01	402	1.96E-02	844	6.27E-04	392	1.79E-02	255	5.83E-02	355	3	6	566.56
2109	NM_203372	ACSL3	7.63E-03	150	6.13E-01	891	3.21E-01	428	1.23E-05	37	8.86E-01	1037	1.83E-01	858	3.95E-01	804	1.71E-01	483	2.21E-02	585	3	1.82E-01	491	5.72E-01	1038	3.91E-01	453	2.18E-04	571	3.20E-04	355	4.39E-02	366	1.43E-01	519	3	6	566.63
991	NM_078467	CDKN1A	9.49E-01	1100	4.31E-02	446	5.58E-01	685	2.16E-01	704	2.68E-01	369	7.60E-04	414	4.58E-01	855	6.00E-01	858	8.61E-04	350	3	2.90E-01	614	1.04E-04	78	3.71E-01	412	1.10E-04	525	2.03E-06	137	6.33E-01	926	2.04E-01	606	3	6	567.44

22950	NM_018158	SLC4A1AP	2.06E-02	261	4.01E-04	27	6.25E-01	760	1.67E-01	671	5.80E-02	111	2.44E-04	358	3.00E-02	203	6.01E-01	859	7.25E-02	709	4	9.28E-01	1105	6.43E-02	579	4.23E-01	495	3.00E-03	721	1.78E-02	655	9.14E-01	1100	1.16E-01	469	2	6	567.69
54468	NM_019005	MIOS	3.93E-03	101	7.42E-01	986	4.11E-02	137	1.32E-04	81	8.81E-01	1029	1.87E-04	344	2.24E-01	619	8.01E-02	331	5.10E-05	228	5	9.69E-01	1135	1.11E-03	192	8.97E-01	1026	2.04E-03	709	1.14E-01	852	8.36E-01	1046	3.81E-02	268	3	8	567.75
483	NM_001679	Atp1b3	1.82E-02	237	1.29E-02	196	1.94E-01	298	5.65E-01	923	9.30E-01	1079	4.37E-01	1004	5.99E-02	315	2.94E-01	615	1.67E-04	274	3	4.74E-01	792	3.14E-01	900	4.60E-01	560	2.89E-02	869	7.15E-04	405	8.93E-02	459	1.82E-02	161	3	6	567.94
5792	NM_002853	RAD1	9.81E-01	1132	4.66E-03	95	2.10E-01	315	8.20E-02	563	5.40E-01	677	6.88E-01	1076	1.88E-01	565	3.56E-02	224	1.49E-10	18	3	4.28E-01	753	9.11E-01	1142	1.75E-01	128	1.88E-06	243	4.81E-01	1017	8.46E-01	1053	7.72E-03	86	2	5	567.94
26511	NM_012110	Chic2	6.86E-02	496	2.35E-02	280	4.27E-01	527	3.20E-01	780	5.85E-01	731	2.76E-09	59	1.96E-01	577	7.58E-01	975	5.34E-02	680	2	8.02E-01	1014	8.91E-02	629	1.89E-01	147	1.70E-03	700	4.10E-01	987	1.41E-02	221	4.25E-02	284	3	5	567.94
7257	NM_005999	Tsnax	2.34E-01	635	1.92E-02	252	9.28E-01	1095	2.15E-02	391	3.04E-01	412	2.11E-03	465	5.27E-01	904	5.30E-02	274	4.06E-06	148	4	9.95E-01	1153	7.67E-03	346	3.93E-01	456	1.53E-03	695	3.72E-04	361	1.04E-01	481	8.09E-01	1027	3	7	568.44
4005	NM_198315	vwa5a	7.81E-02	509	9.21E-03	158	8.55E-01	1022	1.27E-01	623	1.87E-01	254	1.12E-02	573	6.52E-02	334	8.75E-01	1053	7.84E-02	718	2	5.27E-01	831	8.57E-04	177	2.14E-01	183	3.58E-01	1036	8.07E-05	296	8.96E-01	1082	3.47E-02	250	3	5	568.69
8899	NM_003913	prpf4b	8.56E-02	517	6.60E-02	519	3.16E-01	424	5.47E-01	914	8.72E-01	1022	1.52E-01	834	1.65E-01	527	3.50E-01	668	7.82E-05	243	1	6.12E-02	310	3.98E-01	965	1.11E-01	60	3.62E-05	445	2.51E-05	224	1.75E-01	563	5.42E-01	866	2	3	568.81
26509	NM_133337	Myof	4.65E-01	795	4.82E-02	485	6.38E-01	776	4.69E-06	21	5.18E-01	654	5.66E-02	725	7.12E-01	1004	3.77E-01	691	2.45E-04	287	3	9.45E-01	1119	2.87E-03	260	2.91E-01	278	1.93E-01	988	2.75E-11	6	7.06E-01	971	2.94E-03	42	3	6	568.88
143	NM_006437	PARP4	3.65E-02	384	3.78E-01	754	2.89E-02	108	9.00E-03	319	9.76E-01	1125	1.74E-04	342	2.28E-01	623	1.51E-02	137	7.52E-02	712	5	3.14E-01	641	4.81E-01	1000	6.12E-01	740	4.34E-04	618	5.78E-01	1049	2.53E-02	291	3.67E-02	262	3	8	569.06
6733	NM_003153	STAT6	3.12E-02	348	9.55E-01	1125	7.51E-01	902	2.32E-03	204	5.63E-01	704	2.17E-01	878	2.66E-01	679	5.85E-01	847	2.72E-03	426	3	6.01E-02	306	2.51E-02	472	1.87E-01	145	1.56E-01	975	6.44E-04	394	4.08E-02	352	5.63E-02	349	3	6	569.13
23043	NM_001161563	TNIK	4.31E-03	103	1.87E-01	624	3.32E-01	436	7.67E-02	552	3.98E-01	515	3.13E-01	935	2.85E-01	696	9.57E-01	1121	6.11E-03	491	2	2.77E-01	599	2.65E-01	837	4.03E-01	464	6.53E-05	488	3.66E-02	739	4.81E-02	374	1.52E-02	143	4	6	569.81
57180	NM_020533	mcoln1	7.34E-01	930	3.39E-02	375	9.15E-01	1081	3.18E-02	436	5.61E-01	700	8.91E-06	220	5.39E-01	914	6.76E-01	912	2.97E-02	613	4	7.45E-01	975	8.68E-03	363	3.67E-01	406	2.07E-05	400	5.60E-03	549	3.05E-03	133	1.08E-02	113	5	9	570.00
8533	NM_003653	Cops3	4.80E-01	804	1.81E-02	243	8.51E-01	1013	1.13E-02	341	6.08E-02	118	2.61E-13	17	1.51E-01	503	1.83E-01	503	3.94E-02	652	4	7.69E-01	999	9.71E-02	639	5.15E-01	633	3.90E-06	288	9.08E-01	1131	8.15E-03	185	8.42E-01	1052	2	6	570.06
8887	NM_006024	Tax1bp1	3.78E-02	397	9.51E-01	1122	6.81E-01	835	1.19E-03	178	7.49E-01	903	2.26E-02	633	4.18E-03	71	6.34E-02	301	1.90E-03	405	5	1.53E-03	54	2.39E-01	807	5.12E-01	629	2.09E-02	849	2.43E-03	476	2.53E-01	645	4.71E-01	819	3	8	570.25
6301	NM_000332	ATXN1	5.87E-01	854	5.66E-01	860	4.76E-03	33	6.38E-04	143	2.63E-01	363	2.17E-04	350	4.85E-01	869	3.90E-03	70	4.42E-01	954	4	2.19E-01	530	7.55E-03	345	9.88E-01	1146	1.40E-05	372	2.63E-02	696	1.43E-01	525	7.80E-01	1015	3	7	570.31
55129	NM_001100417	ubr7	2.63E-02	317	7.53E-01	994	4.91E-01	603	5.63E-01	920	4.02E-01	523	1.48E-02	604	3.78E-01	791	4.16E-01	728	1.45E-09	30	3	4.62E-01	781	1.25E-01	679	8.06E-01	943	3.75E-06	285	2.03E-06	138	3.67E-01	744	5.03E-03	59	3	6	571.19
9735	NM_014708	KNTC1	7.42E-01	938	4.68E-02	471	1.40E-01	241	1.10E-01	598	4.40E-01	576	4.78E-01	1016	2.84E-01	694	9.99E-01	1157	3.91E-05	216	2	5.04E-03	103	2.41E-02	463	9.44E-01	1092	1.30E-02	814	1.40E-06	127	1.23E-02	206	9.15E-02	428	5	7	571.25
54476	NM_207111	rnf216	2.93E-01	690	1.18E-01	560	3.59E-01	463	2.43E-01	727	3.32E-01	447	2.69E-04	362	2.51E-01	656	1.22E-01	403	8.84E-05	247	2	2.73E-01	594	1.35E-02	397	9.14E-01	1043	2.12E-04	569	5.84E-02	791	1.13E-01	493	3.11E-01	701	2	4	571.44
22872	NM_016211	SEC31A	8.99E-01	1057	3.76E-02	403	4.52E-01	550	5.35E-01	908	2.55E-01	352	8.60E-09	74	1.43E-01	489	6.68E-01	904	3.15E-01	892	2	4.39E-02	270	4.82E-02	554	2.26E-01	189	3.59E-06	282	2.98E-01	950	4.14E-02	356	6.05E-01	914	4	6	571.50
10099	NM_005724	TSPAN3	4.29E-01	776	1.64E-03	56	5.64E-01	691	3.81E-04	117	5.09E-01	646	9.53E-04	428	4.32E-01	833	4.35E-01	736	5.11E-05	229	4	9.96E-01	1154	1.23E-03	203	9.35E-01	1076	3.37E-05	443	1.39E-02	626	4.15E-01	779	5.81E-02	354	3	7	571.69
29079	NM_014166	med4	1.86E-02	241	8.36E-01	1051	4.21E-01	519	1.74E-01	678	6.45E-01	803	1.42E-02	597	7.37E-02	357	5.79E-01	841	1.36E-01	776	2	3.08E-03	76	5.79E-01	1042	1.80E-01	136	1.48E-03	692	1.33E-03	442	4.01E-01	771	1.28E-02	125	4	6	571.69
4999	NM_002553	ORC5L	6.18E-01	866	1.09E-03	43	8.94E-01	1063	5.67E-01	928	6.32E-02	123	2.82E-07	139	6.58E-01	977	4.57E-02	258	2.42E-04	285	4	2.08E-01	519	2.83E-01	857	3.83E-01	434	4.48E-04	622	1.77E-04	332	3.79E-01	754	6.74E-01	948	2	6	571.75
8914	NM_003920	TIMELESS	5.63E-02	479	3.32E-02	371	8.05E-01	964	2.07E-04	88	8.94E-01	1047	9.15E-03	554	1.69E-01	535	5.49E-01	820	1.71E-03	402	4	8.50E-01	1054	1.21E-01	673	6.00E-01	721	1.97E-05	398	2.79E-06	152	4.47E-01	804	7.84E-03	88	3	7	571.88
6778	NM_003155	stc1	4.59E-03	110	9.62E-01	1132	8.11E-01	968	4.50E-05	62	4.44E-01	577	2.97E-01	926	1.08E-01	434	9.34E-02	356	2.34E-02	594	3	3.69E-01	703	3.58E-01	931	8.79E-01	1009	4.60E-02	907	1.12E-05	201	1.43E-03	105	1.41E-02	136	4	7	571.94
55631	NM_017817	RAB20	6.39E-01	873	5.76E-01	865	2.27E-02	88	8.85E-01	1090	1.11E-01	180	6.67E-03	533	7.83E-01	1044	8.83E-01	1064	5.55E-05	230	3	1.51E-01	447	4.45E-02	546	3.64E-01	399	4.74E-06	295	1.73E-02	653	4.08E-01	774	6.50E-03	70	4	7	571.94
8743	NM_003810	TNFSF10	4.13E-01	766	2.77E-02	325	4.87E-01	597	2.79E-03	224	5.86E-01	732	8.80E-02	775	5.93E-02	313	7.21E-01	952	4.61E-04	316	3	6.99E-01	939	9.00E-01	1140	1.95E-01	151	9.80E-04	668	1.46E-07	69	7.12E-01	973	2.67E-02	212	3	6	572.00
26521	XM_001720982	TIMM8B	9.96E-01	1150	7.04E-03	128	1.37E-01	237	8.17E-01	1061	6.50E-02	125	3.32E-04	375	7.40E-04	21	6.81E-01	918	2.66E-01	859	3	1.11E-01	397	5.18E-01	1017	1.41E-01	91	3.07E-05	435	2.29E-01	927	3.23E-01	704	3.24E-01	711	1	4	572.25
481	NM_001677	Atp1b1	8.58E-01	1024	6.00E-01	881	3.25E-02	116	5.23E-01	901	5.93E-01	741	1.59E-05	238	4.50E-04	15	4.92E-01	778	7.62E-02	713	3	3.89E-01	718	3.78E-04	130	5.48E-01	665	5.49E-06	306	4.44E-05	262	2.03E-01	596	8.64E-01	1075	3	6	572.44
3428	NM_005534	IFNGR2	1.91E-01	602	1.69E-02	233	4.71E-01	578	7.29E-01	1005	3.86E-01	505	1.04E-06	171	5.03E-02	273	8.24E-01	1022	4.83E-01	971	2	9.19E-01	1097	2.68E-01	840	2.55E-01	220	9.92E-08	90	4.61E-03	528	1.78E-01	568	1.06E-01	457	2	4	572.50
5257	NM_002643	pigf	8.14E-03	156	7.85E-01	1016	4.73E-01	580	7.71E-04	154	4.54E-01	584	2.68E-08	93	8.83E-01	1090	5.06E-01	784	2.16E-04	283	4	4.92E-01	807	1.58E-02	413	5.79E-01	703	1.31E-02	815	2.92E-01	947	2.76E-01	668	6.38E-03	67	3	7	572.50
10765	NM_006618	KDM5B	3.95E-01	758	1.14E-02	179	4.46E-01	548	4.96E-03	265	8.47E-01	1001	8.75E-01	1127	7.69E-02	371	3.78E-01	693	8.04E-02	722	2	3.91E-02	257	1.82E-01	737	3.60E-01	392	1.07E-06	210	4.82E-03	532	3.64E-01	741	2.25E-01	629	3	5	572.63
617	NM_004328	BCS1L	2.27E-01	630	9.75E-03	163	2.27E-01	336	5.47E-03	274	9.76E-01	1127	4.18E-01	990	8.34E-01	1077	1.20E-02	123	7.93E-01	1078	3	3.88E-01	717	9.96E-02	642	2.07E-01	170	9.37E-08	87	1.51E-01	881	4.57E-01	813	4.75E-03	56	2	5	572.75
5142	NM_002610	PDK1	4.13E-02	418	6.73E-01	927	3.37E-02	123	8.04E-01	1053	6.77E-01	836	6.84E-08	110	3.30E-01	733	1.28E-01	411	7.84E-02	717	3	9.39E-01	1115	1.36E-02	399	4.71E-02	28	5.74E-01	1089	2.97E-04	353	9.29E-02	466	7.28E-02	387	3	6	572.81
64778	NM_022768	RBM15	5.95E-01	857	2.94E-02	340	7.43E-01	892	1.38E-01	634	3.52E-01	467	2.36E-01	889	2.19E-01	612	5.32E-01	801	5.40E-06	157	2	1.14E-01	404	3.31E-01	912	4.39E-01	527	9.98E-06	344	1.64E-04	328	4.66E-01	821	2.21E-02	186	3	5	573.19
54386	NM_018975	Terf2ip	3.55E-02	376	5.88E-01	873	2.93E-01	394	8.88E-07	8	8.56E-01	1012	1.46E-09	53	2.20E-01	614	1.30E-01	415	7.56E-01	1065	3	3.55E-01	689	1.13E-03	194	6.14E-01	741	8.89E-05	511	5.92E-04	386	5.24E-01	850	7.36E-01	991	3	6	573.25
22944	NM_012311	kin	8.01E-01	984	4.36E-02	449	8.23E-01	987	7.33E-02	545	3.68E-01	488	2.74E-03	481	6.38E-02	330	8.39E-01	1029	8.76E-04	352	3	7.21E-01	954	8.89E-06	19	5.05E-01	617	2.53E-06	259	2.08E-05	218	2.43E-01	632	4.93E-01	833	3	6	573.56
9991	NM_005156	ROD1	2.60E-02	314	4.28E-02	443	6.04E-01	744	7.88E-01	1045	2.11E-01	280	7.02E-01	1084	1.84E-02	151	6.27E-02	298	7.79E-01	1073	3	2.58E-01	575	5.61E-02	567	2.28E-01	191	2.38E-05	413	5.33E-04	378	7.50E-01	999	2.19E-01	624	2	5	573.69
25847	NM_015391	ANAPC13	2.82E-01	678	1.34E-02	199	3.64E-01	467	7.34E-05	73	9.45E-01	1095	5.10E-01	1024	1.47E-02	129	5.10E-03	80	5.70E-01	1005	4	1.27E-01	416	7.45E-04	168	6.54E-01	782	6.85E-02	931	3.94E-02	746	6.63E-01	948	9.85E-02	443	2	6	574.00
518	NM_001689	ATP5G3	4.23E-01	772	4.63E-02	467	2.86E-01	386	6.82E-03	295	7.71E-02	137	9.38E-05	320	5.40E-01	915	5.82E-01	845	7.99E-05	245	4	3.91E-04	23	8.07E-01	1111	6.34E-01	760	5.69E-01	1087	7.78E-03	575	9.62E-02	470	4.09E-01	779	2	6	574.19
3964	NM_002310	Lifr	1.61E-05	9	6.23E-01	899	6.23E-01	759	9.45E-02	583	6.90E-01	853	1.31E-01	822	5.34E-02	285	5.04E-08	2	5.50E-03	480	3	1.88E-01	501	1.31E-01	687	5.67E-01	690	4.30E-03	742	4.95E-03	537	8.84E-02	458	5.62E-01	880	2	5	574.19
51067	NM_001040436	Yars2	4.79E-02	460	1.73E-01	611	3.71E-01	475	7.87E-01	1042	9.25E-01	1074	2.06E-02	630	1.53E-03	34	1.19E-01	400	2.78E-01	867	3	1.96E-01	511	2.91E-02	490	5.50E-01	670	3.13E-06	276	9.00E-07	111	3.03E-01	684	5.34E-01	862	3	6	574.81
9130	NM_004699	Fam50a	4.69E-02	452	4.68E-01	809	3.52E-01	458	2.07E-01	693	3.39E-01	456	2.61E-01	907	3.75E-05	2	1.31E-01	416	8.94E-01	1113	2	5.35E-01	839	7.75E-03	349	6.38E-01	768	1.71E-02	833	1.01E-08	36	4.44E-03	152	6.09E-01	917	4	6	575.00
8209	NM_004649	C21orf33	7.56E-01	947	4.97E-02	495	9.32E-01	1100	2.23E-03	202	6.08E-02	117	4.11E-02	693	7.47E-02	361	9.28E-01	1096	2.82E-03	427	4	7.19E-01	949	3.09E-02	503	3.20E-01	323	8.10E-05	505	5.09E-05	271	2.13E-01	610	2.03E-01	605	3	7	575.25
79031	NM_024072	ddx54	1.11E-02	179	8.12E-03	144	5.11E-01	628	7.61E-02	550	1.78E-01	243	3.67E-04	380	7.88E-01	1048	6.80E-04	34	1.05E-02	531	5	8.99E-01	1083	8.40E-01	1118	6.90E-01	819	4.21E-05	453	9.69E-04	422	4.17E-01	781	4.32E-01	793	2	7	575.38
10535	NM_006397	RNASEH2A	2.16E-01	617	1.43E-02	203	9.90E-01	1152	4.49E-02	493	3.39E-01	454	4.04E-04	383	9.34E-01	1123	1.56E-01	461	8.69E-03	513	4	5.47E-01	851	7.07E-01	1080	3.96E-01	460	1.48E-04	544	2.46E-07	80	5.68E-02	399	7.95E-02	398	2	6	575.69
11103	NM_007043	KRR1	2.80E-01	672	1.54E-01	591	6.00E-01	741	7.50E-01	1016	1.66E-01	231	1.33E-01	824	4.13E-01	819	1.60E-01	464	2.22E-02	586	1	1.89E-01	503	2.41E-02	462	4.61E-01	563	2.69E-07	131	4.26E-01	997	1.96E-01	587	2.28E-03	32	3	4	576.19
4E+05	NM_001012991	c16orf88	9.46E-03	167	8.44E-01	1058	4.83E-01	590	1.11E-01	601	3.95E-01	512	6.27E-04	401	2.00E-01	583	5.54E-02	277	4.18E-03	461	3	4.12E-01	739	7.33E-01	1086	3.59E-01	391	2.39E-04	580	2.36E-06	147	1.25E-01	510	9.41E-01	1123	2	5	576.63
56920	NM_020169	LXN	9.67E-01	1123	1.80E-02	241	5.52E-01	678	7.47E-01	1015	5.71E-01	715	2.33E-05	261	8.31E-02	387	9.79E-02	364	1.67E-03	398	3	1.75E-01	483	4.26E-03	300	4.51E-01	544	4.87E-01	1068	5.61E-01	1041	1.69E-02	245	6.36E-02	365	2	5	576.75
7398	NM_001017415	usp1	7.81E-01	964	1.71E-01	607	2.09E-02	83	2.27E-01	714	8.08E-03	21	5.89E-01	1047	3.65E-01	774	2.32E-01	560	4.38E-04	313	3	9.47E-01	1121	3.43E-03	277	7.79E-01	912	9.12E-07	199	1.27E-03	438	1.51E-02	233	6.98E-01	968	4	7	576.94
4869	NM_001101662	NRD1	4.10E-02	413	7.15E-02	522	5.72E-01	702	8.20E-01	1062	8.90E-01	1042	1.30E-01	821	5.76E-01	936	2.83E-02	196	4.63E-02	666	3	8.65E-01	1067	6.64E-05	59	4.27E-01	505	2.86E-07	134	2.10E-02	674	9.21E-04	95	5.38E-02	339	4	7	577.06
10483	NM_032985	SEC23B	1.07E-01	542	7.14E-01	964	4.32E-01	532	8.14E-01	1060	7.27E-01	885	9.08E-04	422	7.21E-02	351	1.14E-01	392	6.54E-04	331	2	2.99E-01	630	1.58E-03	219	2.13E-01	181	2.86E-05	431	9.74E-01	1151	3.27E-01	709	9.55E-02	435	2	4	577.19
5743	NM_002840	ptprf	6.16E-02	485	5.19E-02	501	2.27E-02	89	7.05E-01	997	9.13E-01	1063	8.12E-06	217	7.77E-03	98	1.33E-01	424	3.20E-01	896	3	6.85E-01	925	2.51E-01	820	3.74E-01	417	6.10E-03	760	4.43E-11	8	5.66E-02	397	9.66E-01	1140	2	5	577.31
5901	NM_002882	RanBP1	8.61E-01	1027	2.23E-02	273	2.99E-01	403	1.23E-02	348	6.84E-01	846	3.30E-01	943	1.82E-02	149	4.00E-01	718	6.03E-03	489	4	9.01E-02	366	4.60E-01	993	4.41E-01	529	1.09E-06	211	3.77E-01	972	1.25E-02	207	3.95E-01	765	2	6	577.44
6634	XM_001726340	SNRPE	8.86E-01	1048	2.49E-02	297	3.98E-01	497	1.81E-03	197	9.32E-02	156	2.46E-01	899	1.78E-02	147	1.79E-01	493	4.44E-01	955	3	1.69E-02	165	3.23E-02	507	6.56E-01	784	2.37E-03	712	5.25E-03	544	7.15E-01	977	5.34E-01	861	4	7	577.44
1E+05	NM_052966	FAM129A	1.88E-02	243	4.89E-01	818	4.27E-01	526	2.42E-01	726	7.83E-01	939	7.37E-07	162	8.17E-01	1068	7.97E-01	1001	1.29E-07	80	3	1.21E-04	10	6.14E-02	574	3.16E-01	317	4.11E-03	741	5.65E-03	550	5.78E-01	890	1.96E-01	594	3	6	577.44
29919	NM_013326	C18orf8	8.37E-03	159	9.04E-01	1094	7.76E-01	927	1.12E-01	605	4.71E-01	606	1.69E-09	57	5.34E-01	910	2.00E-01	524	9.34E-02	736	2	3.55E-03	85	7.13E-05	62	5.59E-01	682	6.42E-01	1103	5.33E-05	273	9.61E-01	1133	4.27E-02	286	4	6	577.63
10492	NM_001159674	SYNCRIP	4.64E-02	449	2.00E-01	630	5.94E-01	729	3.19E-04	107	4.29E-01	557	7.55E-09	71	8.20E-02	384	5.38E-01	808	1.87E-05	189	4	2.87E-04	18	8.76E-01	1129	7.73E-01	908	4.81E-05	466	5.81E-01	1050	5.62E-01	879	5.44E-01	869	2	6	577.69
3977	NM_013402	Fads1	9.50E-01	1102	2.60E-02	308	4.64E-01	567	7.88E-01	1043	8.83E-01	1032	1.65E-05	240	5.20E-02	280	3.46E-02	218	4.73E-01	969	3	2.56E-02	210	1.05E-01	650	3.12E-01	310	2.52E-01	1005	1.26E-01	864	3.82E-03	147	4.72E-02	300	3	6	577.81
821	NM_001746	Canx	5.33E-01	832	9.13E-01	1104	4.92E-01	604	3.69E-04	115	2.99E-01	408	3.72E-03	506	9.03E-04	22	1.86E-01	508	1.30E-04	262	4	2.65E-01	585	8.34E-03	356	6.01E-01	726	5.35E-05	477	8.78E-01	1122	7.98E-01	1030	1.96E-01	592	2	6	578.06
26097	NM_015607	c1orf77	1.12E-01	546	2.18E-02	271	2.69E-02	102	1.75E-02	373	6.40E-03	16	1.63E-02	613	4.27E-01	829	9.30E-02	355	1.58E-03	393	6	7.17E-01	947	8.86E-01	1133	8.69E-01	996	1.66E-03	698	3.18E-03	498	6.55E-01	940	1.57E-01	542	2	8	578.25
10611	NM_006457	PDLIM5	8.30E-01	1010	2.04E-01	635	8.52E-01	1016	6.97E-01	992	5.90E-01	737	4.39E-03	512	1.57E-03	38	3.97E-02	238	2.87E-01	876	3	6.97E-03	113	1.02E-04	77	2.86E-01	267	1.01E-02	798	2.98E-01	949	1.09E-01	486	1.38E-01	511	3	6	578.44
63891	NM_022065	Thada	2.06E-02	259	4.89E-01	819	1.04E-01	209	9.90E-02	588	8.30E-02	146	7.88E-01	1106	4.98E-02	269	1.99E-01	523	6.38E-04	329	3	4.90E-01	806	4.49E-03	305	6.98E-01	827	8.61E-01	1142	1.53E-02	639	9.46E-01	1123	1.99E-02	169	3	6	578.69
8522	NM_201432	gas7	1.36E-02	205	5.55E-01	853	7.66E-01	915	8.45E-01	1071	2.24E-01	304	2.22E-16	3	1.39E-01	485	8.79E-02	346	3.53E-03	445	3	8.25E-01	1036	3.54E-02	516	9.69E-01	1129	2.18E-01	995	1.50E-02	636	1.30E-02	210	1.24E-02	123	4	7	579.50
4552	NM_130439	MXI1	7.44E-01	941	1.10E-02	177	7.13E-01	865	2.33E-01	720	9.01E-01	1052	2.89E-05	267	6.01E-01	948	5.72E-02	283	1.54E-07	82	3	5.37E-01	840	2.53E-04	107	9.56E-01	1109	3.37E-07	141	1.02E-02	600	2.15E-01	613	1.53E-01	531	3	6	579.75
10480	NM_006360	EIF3M	1.35E-03	57	2.47E-01	665	7.79E-01	930	3.01E-02	428	2.51E-01	348	1.00E-02	561	2.38E-01	636	8.61E-01	1043	2.09E-02	581	4	9.66E-01	1132	2.90E-02	489	2.68E-01	244	2.08E-04	567	2.12E-01	916	1.34E-02	213	1.14E-01	467	3	7	579.81
55422	NM_018656	slc35e3	3.42E-02	367	6.87E-01	944	6.78E-01	830	3.70E-02	462	3.34E-02	68	1.79E-03	456	8.25E-01	1073	6.97E-01	933	3.36E-01	903	4	1.74E-01	479	4.02E-03	295	3.88E-01	447	3.18E-02	880	1.21E-07	65	2.35E-01	623	1.03E-01	453	3	7	579.88
5471	NM_000942	ppiB	8.03E-01	986	1.64E-02	228	4.42E-01	547	3.92E-01	825	5.06E-01	644	2.51E-03	477	4.07E-01	817	1.35E-01	425	2.88E-08	63	3	7.88E-02	347	7.80E-01	1096	6.66E-01	792	2.44E-05	416	7.80E-03	577	1.20E-01	503	1.59E-01	544	2	5	580.44
4087	NM_004990	mars	9.64E-01	1120	2.24E-02	276	9.01E-01	1072	6.22E-05	70	2.52E-01	349	4.59E-07	150	6.28E-01	961	3.48E-01	667	8.81E-03	515	4	7.39E-01	966	9.72E-02	640	3.71E-01	411	3.13E-01	1026	1.06E-04	310	2.84E-01	672	8.38E-03	98	2	6	581.44
3006	XM_930111	h3f3a	7.97E-03	154	6.92E-01	949	4.64E-01	568	3.42E-01	797	9.94E-01	1151	2.73E-04	364	1.07E-01	432	2.36E-01	563	5.37E-02	681	2	3.07E-02	227	5.09E-03	313	2.94E-01	280	6.23E-03	762	4.50E-02	765	6.99E-02	425	5.48E-01	873	4	6	581.50
6051	NM_001024662	rpl6	1.70E-02	225	3.84E-01	756	4.31E-01	531	4.42E-04	125	8.85E-01	1036	3.21E-01	937	7.32E-01	1013	2.35E-02	178	6.61E-01	1035	3	6.31E-01	898	1.32E-03	205	3.45E-01	368	2.13E-05	405	9.49E-02	828	2.54E-02	293	1.23E-01	491	3	6	582.75
64397	NM_001029991	METT11D1	1.18E-02	187	7.86E-01	1017	7.92E-01	947	5.07E-01	885	3.03E-01	411	1.58E-10	38	4.87E-01	872	3.32E-01	648	9.18E-01	1118	2	1.61E-02	161	2.37E-02	461	4.44E-01	533	1.44E-01	971	4.04E-03	516	2.64E-03	126	9.51E-02	434	4	6	582.81
7314	NM_018955	UBB	7.83E-01	967	9.87E-03	165	1.27E-01	230	3.34E-04	109	9.40E-01	1089	8.33E-01	1114	2.04E-01	590	9.77E-03	109	1.68E-01	802	3	5.24E-01	830	8.37E-05	69	4.14E-01	484	1.72E-04	555	1.18E-01	859	4.04E-02	351	7.63E-01	1005	3	6	583.00
79665	NM_024613	plekhf2	3.44E-02	369	2.95E-01	700	4.19E-02	141	5.56E-01	918	5.98E-01	746	1.69E-05	243	3.97E-01	806	1.37E-04	18	8.28E-01	1090	4	1.12E-01	400	6.15E-04	158	7.65E-01	897	5.37E-03	752	4.01E-01	981	1.86E-02	259	5.17E-01	852	3	7	583.13
7091	NM_003272	GPR137B	2.64E-02	318	8.43E-01	1057	7.59E-01	907	4.81E-01	873	2.75E-02	53	2.13E-05	257	1.03E-01	428	6.37E-03	88	3.07E-01	887	4	4.37E-02	269	2.11E-01	774	5.37E-01	653	2.12E-01	993	2.53E-02	694	1.74E-01	561	1.47E-01	521	2	6	583.31
4714	NM_005006	NDUFS1	5.37E-01	833	4.34E-02	447	7.74E-01	925	7.51E-03	309	3.20E-02	66	2.64E-01	908	7.39E-01	1015	4.93E-01	779	3.84E-01	925	3	4.41E-02	271	6.71E-02	587	3.32E-01	346	4.37E-05	456	2.97E-09	23	2.35E-01	624	4.77E-01	824	3	6	583.63
8992	NM_003945	ATP6V0E1	3.54E-01	731	1.75E-01	615	4.35E-01	536	7.23E-01	1003	9.96E-01	1152	1.75E-02	619	1.33E-02	121	4.85E-01	773	2.44E-02	596	3	2.00E-02	185	2.25E-03	242	7.30E-01	855	8.73E-03	787	3.58E-01	968	2.68E-03	129	1.93E-03	27	5	8	583.69
9134	NM_057749	Ccne2	8.73E-01	1037	1.37E-01	576	3.02E-01	406	3.05E-01	769	1.49E-01	215	4.91E-01	1019	8.90E-02	396	1.88E-01	509	3.43E-02	634	1	3.99E-01	731	2.60E-05	33	5.43E-01	661	9.48E-02	951	3.12E-05	239	4.19E-01	783	7.41E-02	388	2	3	584.19
899	NM_001761	CCNF	9.99E-01	1157	2.48E-02	296	5.07E-01	620	1.31E-04	80	8.66E-02	151	8.48E-01	1119	2.55E-02	185	3.46E-01	660	7.05E-01	1049	3	1.08E-01	393	2.08E-03	234	4.38E-01	526	8.38E-01	1137	3.76E-05	253	7.65E-01	1006	1.20E-01	485	2	5	584.44

9236	NM_004748	CCPG1	4.46E-03	108	2.45E-01	663	9.75E-01	1141	4.84E-01	875	5.13E-01	648	8.78E-04	420	9.57E-01	1136	1.82E-04	21	8.73E-04	351	4	2.96E-01	623	6.03E-02	572	1.64E-01	118	4.20E-02	902	1.25E-01	863	1.76E-02	253	2.62E-01	660	2	6	584.63
4860	XM_496355	Npm1	2.25E-02	286	4.73E-02	476	6.27E-01	761	3.83E-01	819	4.68E-01	601	2.96E-10	45	7.69E-01	1033	1.58E-02	143	6.92E-01	1044	4	1.99E-01	514	1.62E-01	717	5.67E-01	691	2.90E-03	719	6.41E-07	101	3.07E-01	687	3.35E-01	720	2	6	584.81
4898	NM_006184	NUCB1	6.85E-03	141	2.01E-01	631	8.22E-01	986	3.11E-03	232	4.79E-01	618	7.40E-05	306	9.95E-02	420	2.87E-02	198	5.16E-02	674	4	2.33E-02	194	2.38E-01	805	7.50E-01	876	4.52E-01	1060	5.60E-01	1040	4.19E-01	782	7.81E-02	394	1	5	584.81
7873	NM_006010	manf	4.67E-02	451	1.75E-01	614	4.61E-01	564	1.07E-01	597	2.13E-01	284	8.17E-02	763	1.43E-02	128	7.74E-01	989	2.44E-01	847	2	4.74E-01	791	4.72E-02	552	5.83E-01	708	7.61E-05	502	1.17E-02	613	3.35E-02	320	2.37E-01	638	4	6	585.06
10095	XM_936251	ARPC1B	2.62E-02	316	8.40E-01	1055	4.59E-01	562	1.43E-01	642	7.96E-01	954	4.22E-01	993	1.03E-02	107	2.87E-01	606	6.93E-04	332	3	3.54E-01	686	7.48E-05	64	8.61E-01	987	3.65E-03	736	3.61E-02	738	4.76E-05	33	1.68E-01	561	4	7	585.75
51651	NM_016077	ptrh2	7.06E-02	499	3.85E-02	409	2.90E-02	109	9.81E-03	330	2.58E-01	354	1.75E-01	853	4.32E-01	834	9.65E-01	1125	1.61E-03	395	4	1.69E-01	474	2.72E-01	843	5.92E-01	714	3.54E-02	892	7.96E-01	1105	1.97E-02	265	2.02E-02	171	3	7	585.75
311	NM_145869	anxa11	2.51E-02	307	7.33E-01	978	2.24E-01	330	2.70E-03	219	2.01E-01	267	6.59E-01	1067	2.47E-01	651	9.49E-02	359	1.18E-04	259	3	5.66E-01	860	1.07E-01	654	9.41E-01	1085	5.68E-04	640	1.00E-02	598	3.10E-01	689	8.67E-02	416	2	5	586.19
27429	NM_013247	HTRA2	6.17E-01	865	6.44E-01	910	8.01E-01	957	9.27E-03	325	7.23E-01	883	6.59E-05	299	3.04E-01	717	9.08E-02	349	1.93E-03	406	3	4.52E-01	774	6.65E-06	16	3.32E-01	345	5.86E-06	309	1.71E-04	330	4.57E-01	812	8.80E-01	1086	3	6	586.44
6310	NM_002999	Sdc4	8.83E-02	522	4.78E-02	481	4.83E-01	592	1.74E-02	372	5.70E-01	713	9.05E-06	221	8.79E-01	1088	1.05E-01	374	9.77E-01	1149	3	2.99E-01	627	3.04E-01	887	7.88E-01	923	6.34E-04	649	1.61E-04	327	3.17E-04	71	7.79E-02	393	3	6	586.81
4925	NM_139132	NUP98	1.50E-03	61	8.09E-01	1030	5.69E-01	694	2.30E-05	51	2.91E-02	58	1.62E-01	842	8.23E-01	1072	6.11E-01	865	2.98E-04	297	4	4.98E-01	809	2.95E-02	496	7.35E-01	861	1.10E-04	524	1.28E-03	440	5.52E-01	875	8.76E-02	418	3	7	587.06
8030	NM_005436	CCDC6	2.24E-01	627	2.86E-02	331	6.35E-01	773	1.29E-01	626	4.14E-01	537	6.18E-05	297	1.38E-04	9	1.16E-01	394	8.37E-01	1093	3	6.72E-03	111	7.02E-01	1076	5.06E-01	621	2.91E-04	590	7.79E-01	1100	6.68E-01	950	3.63E-02	258	3	6	587.06
54821	NM_017742	ZCCHC2	9.30E-01	1080	1.18E-02	183	2.67E-01	363	4.98E-02	506	5.89E-01	736	2.87E-05	266	1.32E-01	472	3.22E-01	639	8.95E-03	518	4	1.00E-01	382	2.39E-01	808	3.66E-01	403	1.94E-02	843	4.39E-05	260	9.75E-01	1147	4.21E-01	787	2	6	587.06
10048	NM_005493	RANBP9	4.89E-02	464	7.78E-01	1008	3.85E-01	483	2.27E-01	715	3.78E-01	499	6.18E-04	399	7.16E-02	349	8.02E-01	1006	3.99E-05	217	3	3.32E-03	81	6.34E-01	1059	4.57E-01	556	6.88E-01	1116	5.10E-02	777	8.04E-03	183	1.20E-01	483	2	5	587.19
3659	NM_002201	ISG20	2.83E-01	680	3.91E-01	760	7.02E-03	49	1.29E-01	625	8.05E-01	963	3.24E-03	497	3.22E-02	212	3.60E-01	675	3.85E-01	926	3	3.68E-01	702	7.32E-02	596	7.85E-01	921	4.80E-04	628	6.83E-01	1072	9.29E-05	47	3.61E-03	47	3	6	587.50
4800	NM_005008	NHP2L1	5.89E-02	482	7.13E-01	960	2.65E-01	362	1.89E-01	683	2.16E-02	37	7.66E-01	1100	9.27E-02	405	1.16E-01	393	7.92E-04	344	2	1.16E-02	138	2.23E-01	786	9.53E-01	1106	4.75E-07	157	4.67E-02	768	5.21E-01	848	4.95E-01	835	3	5	587.75
9093	NM_005147	DNAJA3	8.52E-01	1018	4.24E-02	438	9.93E-03	59	5.01E-02	507	4.07E-01	531	2.34E-01	887	5.05E-01	884	4.73E-02	264	7.62E-04	340	4	7.74E-01	1002	4.14E-01	976	4.94E-01	601	7.46E-08	75	3.59E-06	163	5.66E-01	881	4.12E-01	782	2	6	588.00
1052	NM_001814	CTSC	9.29E-01	1078	5.15E-04	29	1.34E-01	235	7.71E-01	1030	6.27E-01	782	3.21E-02	666	5.28E-02	283	4.74E-01	759	1.15E-11	9	3	2.17E-01	527	2.87E-01	863	1.59E-01	108	2.11E-02	850	9.18E-01	1137	1.92E-04	61	7.38E-01	993	2	5	588.13
56902	NM_020163	SEMA3G	3.17E-01	709	1.70E-03	58	8.32E-01	993	2.45E-03	211	6.50E-01	810	6.60E-01	1069	6.67E-01	983	8.64E-01	1047	4.55E-09	46	3	2.26E-01	534	3.62E-03	283	7.55E-01	886	1.29E-08	28	7.43E-01	1093	5.51E-03	163	1.30E-01	497	3	6	588.13
5889	NM_006325	Ran	9.26E-01	1075	3.23E-02	364	6.58E-01	801	4.40E-02	489	6.34E-02	124	8.52E-01	1120	1.03E-01	426	3.78E-01	692	2.15E-04	282	3	2.86E-01	610	1.65E-03	222	6.37E-01	767	1.09E-04	523	3.26E-03	500	9.86E-01	1155	3.73E-02	265	4	7	588.44
26574	NM_012138	AATF	8.55E-01	1020	2.84E-03	71	4.18E-01	516	6.46E-03	288	7.09E-01	876	2.83E-03	484	2.00E-01	584	5.21E-01	796	4.41E-01	953	3	1.69E-02	166	9.58E-01	1152	5.07E-01	624	1.68E-03	699	2.07E-06	140	1.12E-01	490	1.66E-01	558	3	6	588.56
2342	NM_000146	FTL	2.94E-01	692	2.67E-02	316	7.45E-01	894	8.11E-01	1058	8.84E-01	1033	3.14E-01	936	5.22E-02	281	3.17E-02	208	4.17E-09	44	3	1.09E-01	394	7.85E-02	606	2.14E-01	182	7.84E-03	781	7.67E-01	1099	3.56E-01	734	1.89E-02	166	2	5	589.00
5226	NM_000291	PGK1	4.45E-02	440	1.46E-01	585	3.99E-01	500	5.58E-03	276	1.23E-01	190	3.49E-01	951	6.02E-03	86	9.79E-01	1140	4.52E-01	961	3	9.97E-02	381	7.03E-01	1077	3.61E-01	394	4.89E-04	632	7.44E-01	1094	6.41E-03	170	1.64E-01	553	2	5	589.38
54802	NM_017669	ERCC6L	5.78E-03	129	3.97E-01	764	6.24E-02	177	5.69E-01	930	6.90E-01	852	2.31E-05	260	7.70E-01	1036	2.16E-01	542	5.71E-02	689	2	1.31E-01	421	3.60E-02	519	7.43E-01	869	5.18E-06	304	1.18E-03	435	2.05E-01	600	5.98E-01	909	3	5	589.75
10592	NM_001042550	SMC2	4.16E-01	768	1.80E-01	620	3.80E-02	131	3.31E-01	790	2.29E-02	41	2.30E-02	640	1.42E-01	488	7.17E-01	945	1.59E-03	394	4	1.86E-02	176	6.18E-03	330	8.91E-01	1020	1.18E-01	961	3.54E-04	359	5.51E-01	874	5.94E-01	904	3	7	590.06
9702	NM_014679	Cep57	5.18E-01	825	7.31E-01	975	2.11E-02	85	9.17E-01	1113	2.98E-01	404	1.03E-02	564	1.05E-01	431	1.04E-02	112	5.03E-06	154	4	1.64E-01	468	3.22E-02	506	9.90E-01	1150	2.16E-03	710	7.15E-02	806	9.62E-01	1134	3.25E-04	6	3	7	590.19
22827	NM_001136033	PUF60	4.94E-01	812	2.77E-02	324	3.31E-01	434	5.57E-04	136	2.72E-01	374	2.23E-01	881	2.56E-01	664	3.27E-01	643	4.63E-01	967	2	6.24E-02	313	2.65E-03	253	4.65E-01	570	2.20E-02	853	2.18E-02	679	1.56E-01	538	7.61E-01	1004	3	5	590.31
23560	NM_012341	GTPBP4	9.60E-01	1113	1.52E-02	213	9.75E-01	1140	7.07E-04	147	6.70E-01	831	1.54E-06	180	5.96E-01	945	1.08E-01	383	1.13E-03	373	4	8.22E-01	1033	4.93E-01	1006	5.78E-01	702	7.56E-07	188	5.01E-01	1026	4.83E-03	158	7.25E-04	13	3	7	590.69
7084	NM_007005	Tle4	2.83E-01	681	2.91E-01	694	5.37E-03	40	4.10E-02	481	2.71E-01	372	6.31E-02	734	5.91E-02	312	4.27E-01	734	8.35E-07	109	3	3.19E-02	232	3.20E-01	903	9.15E-01	1046	4.29E-02	905	7.83E-04	411	7.87E-01	1022	4.07E-01	777	3	6	590.81
93349	NM_022754	SFXN1	7.60E-01	950	2.72E-02	321	1.64E-01	265	9.26E-01	1121	5.49E-01	683	4.35E-05	280	8.04E-01	1062	4.42E-01	741	3.64E-03	448	3	8.34E-01	1045	1.25E-01	677	1.58E-01	107	1.03E-03	671	1.09E-04	312	3.68E-01	745	2.61E-03	36	3	6	591.50
79188	NM_030763	HMGN5	1.73E-04	23	1.05E-02	171	9.22E-01	1088	2.39E-01	723	3.10E-01	418	5.53E-01	1037	5.09E-01	885	5.73E-02	284	7.36E-05	241	3	6.14E-01	889	4.72E-01	997	6.05E-01	732	2.96E-06	273	2.43E-05	222	1.23E-01	507	7.27E-01	984	2	5	592.13
5048	NM_138766	pam	5.02E-03	119	3.97E-01	763	9.84E-01	1147	8.01E-06	30	9.73E-01	1122	7.34E-02	750	3.30E-01	734	8.62E-02	342	3.63E-01	920	2	1.38E-01	435	2.43E-02	464	5.35E-01	651	7.63E-07	190	4.26E-03	519	3.35E-01	714	1.80E-01	578	3	5	592.38
9592	NM_004907	ier2	8.00E-01	981	2.35E-03	67	6.57E-01	800	3.58E-04	113	6.80E-01	842	9.59E-04	429	9.08E-01	1108	5.45E-01	815	9.86E-04	363	4	1.03E-01	386	1.16E-01	663	5.53E-01	673	3.71E-05	447	2.28E-06	145	1.57E-01	539	9.29E-01	1115	2	6	592.88
10376	NM_006082	TUBA1B	1.90E-01	601	4.55E-01	803	4.88E-01	599	5.34E-04	135	8.96E-01	1050	2.12E-01	875	5.17E-01	894	9.18E-02	352	1.50E-03	391	2	3.16E-02	229	8.05E-01	1107	2.57E-01	223	2.24E-04	577	8.76E-06	191	3.72E-01	748	3.30E-01	714	3	5	593.06
832	NM_004930	CAPZB	6.45E-03	137	6.58E-01	921	8.68E-01	1037	5.18E-01	896	2.61E-01	358	2.35E-05	263	6.32E-01	964	6.23E-01	876	9.79E-04	361	3	3.39E-01	668	7.37E-02	598	8.10E-01	950	5.95E-05	481	1.17E-01	855	7.03E-04	87	2.70E-03	38	3	6	593.13
23521	NM_012423	Rpl13a	2.37E-01	638	9.95E-01	1156	7.29E-01	881	2.33E-03	205	5.79E-01	723	9.85E-01	1153	3.92E-02	232	7.61E-01	978	5.23E-02	677	2	3.13E-01	640	2.08E-02	447	2.90E-01	276	1.91E-06	244	9.40E-09	33	1.54E-02	238	7.01E-01	969	4	6	593.13
162	NM_145730	AP1B1	2.99E-03	88	7.87E-01	1018	5.98E-01	735	3.86E-02	473	2.17E-01	294	3.60E-02	678	3.65E-01	775	1.46E-01	445	2.19E-01	840	3	6.80E-02	321	8.91E-02	627	4.95E-01	602	3.21E-05	439	3.55E-01	966	3.47E-03	138	8.43E-01	1055	2	5	593.38
5825	NM_000320	QDPR	2.49E-02	305	6.90E-01	946	3.05E-01	412	4.79E-02	500	2.26E-02	40	1.26E-01	816	4.56E-02	258	3.93E-01	706	2.72E-01	863	4	4.20E-02	266	1.92E-01	750	7.85E-01	919	3.41E-01	1032	2.70E-03	483	6.65E-02	417	4.22E-01	788	2	6	593.81
2E+05	NM_001080412	ZBTB38	2.77E-01	669	2.61E-03	69	7.49E-02	188	5.66E-02	525	9.12E-01	1062	1.62E-01	843	3.94E-01	803	2.58E-01	581	9.81E-04	362	2	1.53E-01	452	4.61E-02	551	5.07E-01	625	2.11E-05	403	7.25E-02	808	8.62E-02	452	9.18E-01	1109	2	4	593.88
23370	NM_001130955	ARHGEF18	8.76E-03	163	9.22E-01	1109	8.82E-01	1052	3.56E-02	454	3.16E-01	426	2.19E-03	468	2.29E-02	177	8.59E-01	1042	4.20E-01	944	4	9.59E-01	1128	3.11E-02	505	6.99E-02	36	1.66E-05	387	3.46E-01	965	2.43E-03	125	1.49E-01	525	3	7	594.13
8031	NM_001145260	NCOA4	3.60E-03	95	9.31E-01	1111	9.17E-01	1084	2.29E-03	203	4.02E-01	525	3.13E-05	273	3.05E-01	718	8.05E-01	1009	3.99E-01	933	3	9.31E-01	1109	1.22E-04	83	5.25E-01	643	1.03E-03	672	1.32E-05	204	1.50E-01	529	4.98E-02	316	4	7	594.19
25820	NM_005744	ARIH1	4.42E-02	439	8.10E-01	1031	9.90E-01	1151	5.43E-03	272	4.56E-01	588	1.01E-01	791	4.86E-02	266	6.08E-01	863	4.96E-06	153	4	3.15E-03	79	2.06E-01	766	6.67E-01	794	1.54E-05	379	4.12E-01	988	1.39E-02	219	3.47E-01	728	3	7	594.19
9367	NM_004251	RAB9A	7.93E-01	976	7.15E-01	965	8.14E-01	971	8.49E-01	1074	1.45E-01	210	2.19E-05	258	3.56E-01	766	7.73E-02	324	1.79E-01	814	1	7.79E-02	345	1.62E-03	220	7.51E-01	878	1.76E-06	237	3.78E-03	510	4.54E-01	809	1.72E-02	151	4	5	594.25
6619	XM_001725324	SNRPD1	8.54E-03	160	7.39E-01	982	3.47E-01	453	3.67E-01	814	5.41E-01	679	2.50E-01	900	4.22E-02	246	3.55E-01	672	5.27E-03	477	3	7.28E-01	961	1.10E-01	658	1.80E-01	135	6.56E-03	765	1.57E-03	451	6.02E-01	903	3.64E-02	259	3	6	594.69
5168	NM_012393	PFAS	2.32E-03	76	5.85E-01	870	7.31E-01	882	8.51E-01	1076	4.22E-01	548	4.44E-15	7	7.04E-02	345	6.40E-01	886	5.99E-03	488	3	7.62E-01	989	4.47E-02	547	8.67E-01	993	7.21E-05	499	1.43E-02	629	8.71E-06	18	2.65E-01	665	4	7	594.88
55154	NM_018126	tmem33	3.92E-01	755	7.82E-01	1012	3.10E-02	113	9.60E-01	1141	6.58E-01	818	1.44E-04	333	1.53E-01	507	4.03E-02	241	4.11E-01	938	3	1.27E-03	53	1.44E-01	706	3.75E-01	418	2.24E-04	576	1.85E-01	901	4.76E-01	824	2.18E-02	182	3	6	594.88
6717	NM_003134	SRP14	2.33E-02	291	4.92E-01	820	8.87E-01	1057	1.09E-05	35	9.82E-01	1133	2.36E-01	890	5.73E-01	933	1.70E-01	480	3.90E-07	100	3	5.55E-01	853	1.38E-03	209	4.10E-01	477	9.19E-01	1152	1.87E-06	135	9.60E-02	469	1.21E-01	487	2	5	595.06
92342	NM_138387	G6pc3	8.12E-01	995	3.52E-02	385	2.51E-01	356	8.46E-01	1072	2.07E-01	277	6.61E-01	1070	3.33E-01	735	1.91E-01	517	2.63E-03	424	2	1.82E-02	173	8.55E-01	1121	1.96E-01	152	6.44E-07	175	4.55E-01	1007	8.26E-01	1041	2.59E-03	35	3	5	595.94
5577	NM_006256	pkn2	8.17E-01	1001	2.75E-02	322	4.85E-01	594	6.66E-01	974	4.47E-01	580	7.97E-01	1108	1.35E-01	476	2.55E-02	185	6.99E-03	495	3	7.40E-01	969	1.15E-03	195	2.33E-01	193	1.95E-01	989	1.07E-01	837	1.46E-06	10	2.09E-01	613	2	5	596.31
51167	NM_016230	CYB5R4	2.22E-03	74	1.19E-01	561	5.87E-01	719	4.66E-01	864	5.40E-01	676	6.94E-05	304	2.66E-01	677	6.77E-01	913	3.04E-02	616	3	5.45E-01	848	9.32E-03	369	2.40E-01	204	6.85E-03	773	9.70E-01	1149	2.19E-03	119	3.01E-01	695	3	6	597.56
4928	NM_006190	ORC2L	2.32E-02	290	9.73E-02	539	4.74E-01	582	1.39E-03	190	6.92E-01	856	1.05E-01	796	6.02E-01	949	4.95E-02	270	4.59E-01	965	3	5.95E-01	877	2.30E-01	797	3.42E-01	359	5.75E-01	1090	2.42E-04	345	5.52E-03	164	1.31E-01	499	2	5	598.00
81689	NM_030963	RNF146	3.20E-01	711	2.58E-02	305	5.56E-01	683	6.25E-02	536	1.05E-01	171	4.08E-01	987	2.55E-01	660	9.11E-01	1081	2.27E-03	414	2	2.68E-01	588	7.33E-03	344	1.61E-01	109	3.57E-02	895	1.19E-02	616	1.94E-01	584	5.68E-01	885	3	5	598.06
26156	NM_015659	Rsl1d1	3.90E-03	100	1.77E-01	617	3.29E-01	433	3.01E-01	767	4.73E-01	609	8.08E-02	761	5.18E-01	897	1.80E-01	497	7.55E-01	1064	1	9.55E-01	1125	2.56E-02	476	1.88E-01	146	5.89E-07	170	1.94E-01	905	2.86E-04	69	6.54E-01	937	3	4	598.31
11044	NM_006999	papd7	1.17E-03	52	7.68E-01	1000	2.77E-01	372	4.49E-03	254	5.37E-01	668	9.86E-01	1154	1.09E-01	435	8.04E-01	1008	8.07E-01	1082	2	1.05E-02	133	8.46E-04	175	9.39E-01	1080	1.18E-02	811	2.22E-06	142	6.57E-01	943	3.74E-02	266	5	7	598.44
8763	NM_001142402	CD164	7.59E-03	148	9.16E-01	1106	8.95E-01	1067	4.04E-02	479	7.90E-01	950	7.19E-02	748	1.06E-02	108	1.31E-01	417	1.75E-09	33	4	1.90E-01	506	2.00E-01	758	7.62E-01	893	1.97E-05	397	3.06E-02	718	3.86E-01	760	1.22E-01	488	2	6	598.50
29103	NM_013238	DNAJC15	8.93E-01	1055	3.66E-01	745	1.08E-03	18	1.57E-06	13	1.91E-01	259	1.42E-02	595	1.45E-01	491	1.49E-01	452	5.60E-01	998	3	6.91E-01	931	8.30E-01	1116	6.02E-01	728	1.65E-04	549	5.83E-04	384	7.96E-01	1028	2.77E-02	215	3	6	598.56
6845	NM_006754	SYPL1	4.52E-02	444	7.00E-01	953	6.10E-01	752	1.24E-01	617	9.64E-01	1112	3.87E-02	688	1.85E-01	558	1.55E-01	460	5.29E-02	678	2	7.73E-02	344	2.46E-02	467	2.18E-01	184	5.82E-06	307	3.42E-02	730	4.76E-01	825	1.09E-01	463	3	5	598.88
4E+05	NM_001013699	h3f3c	1.10E-01	543	6.40E-01	907	9.89E-01	1150	1.81E-03	196	6.68E-01	829	6.83E-02	743	2.64E-01	673	1.45E-01	444	5.70E-01	1004	1	8.46E-04	45	7.27E-03	342	1.81E-01	138	1.28E-03	686	2.86E-02	710	4.53E-01	807	6.55E-02	371	4	5	599.25
10574	NM_001009570	Cct7	4.23E-01	773	2.64E-02	312	3.99E-01	498	6.05E-03	280	9.45E-01	1093	2.78E-01	919	5.18E-01	895	3.82E-01	700	3.02E-03	433	3	1.13E-01	402	8.11E-02	615	9.37E-01	1078	1.17E-06	217	4.62E-01	1012	1.55E-02	239	1.30E-02	127	3	6	599.56
7965	NM_001013739	AIMP2	7.42E-01	939	5.14E-03	106	1.45E-03	21	1.01E-02	332	5.80E-01	726	2.98E-02	661	1.69E-02	141	7.70E-02	323	3.48E-03	444	6	9.84E-01	1145	2.43E-01	811	9.86E-01	1142	2.64E-06	261	7.56E-03	574	8.61E-01	1060	6.02E-01	910	2	8	599.75
10463	NM_006345	SLC30A9	8.15E-01	997	8.67E-01	1071	2.84E-02	106	4.20E-01	841	9.88E-02	163	1.08E-03	431	3.04E-01	715	7.67E-01	985	2.47E-04	288	3	7.60E-03	117	1.69E-01	721	5.71E-01	696	2.09E-04	568	1.47E-06	129	9.63E-01	1135	2.28E-01	633	3	6	599.75
5329	NM_000302	PLOD1	1.10E-01	544	6.76E-01	932	3.96E-01	496	7.47E-01	1014	3.66E-01	481	3.21E-10	47	1.51E-02	132	2.68E-02	191	9.44E-01	1135	3	7.83E-01	1005	2.88E-01	867	2.59E-01	227	2.77E-05	427	8.89E-01	1123	2.86E-01	674	4.81E-02	305	2	5	600.00
6198	NM_006270	Rras	6.43E-02	487	2.31E-01	651	8.26E-01	991	1.69E-02	369	3.89E-01	506	9.99E-01	1157	3.03E-01	714	3.39E-01	654	1.95E-02	576	2	2.67E-02	213	6.97E-03	340	4.33E-01	518	4.39E-04	619	1.50E-08	40	8.60E-01	1059	3.21E-01	710	4	6	600.25
5869	NM_004582	RABGGTB	4.22E-02	427	9.33E-01	1113	1.26E-01	228	3.88E-01	822	2.63E-02	51	1.41E-02	594	8.01E-01	1059	9.04E-01	1077	1.68E-01	804	3	8.12E-01	1027	2.09E-04	103	2.03E-01	163	6.37E-05	486	8.74E-05	299	1.66E-01	549	4.51E-01	804	3	6	600.38
203	NM_000476	ak1	9.24E-01	1073	4.75E-03	99	3.15E-01	423	7.30E-01	1007	2.43E-01	336	7.45E-05	308	2.67E-01	680	6.21E-01	874	1.55E-05	182	3	1.53E-01	453	2.24E-01	790	3.31E-01	344	2.45E-04	582	5.90E-02	792	8.68E-01	1062	2.07E-01	610	1	4	600.94
5495	NM_002707	Ppm1g	5.38E-01	834	5.56E-01	855	2.82E-01	381	9.98E-01	1158	5.77E-02	109	6.91E-01	1079	6.53E-03	90	1.09E-01	385	5.88E-01	1014	1	1.23E-02	140	1.01E-04	76	3.93E-01	455	2.73E-02	865	4.07E-01	984	7.97E-01	1029	1.94E-02	168	4	5	601.38
23219	NM_015176	Fbxo28	1.91E-02	247	6.01E-01	882	5.12E-01	629	3.20E-02	438	5.19E-01	655	5.80E-02	728	7.37E-04	20	9.89E-01	1150	3.89E-08	65	4	4.04E-01	737	3.66E-01	940	6.04E-01	729	2.96E-09	13	2.15E-03	464	6.28E-01	924	7.59E-01	1002	2	6	601.44
50618	NM_019595	Itsn2	9.10E-01	1061	6.77E-01	933	4.00E-02	134	7.20E-01	1001	6.79E-02	128	1.31E-05	231	9.42E-03	106	2.31E-02	174	1.48E-01	785	4	7.65E-01	993	1.16E-03	196	8.95E-01	1024	7.82E-02	942	2.14E-02	677	3.48E-01	729	1.38E-01	510	2	6	601.50
3304	NM_002154	HSPA4	1.38E-02	206	1.45E-01	584	3.07E-01	417	1.44E-05	40	4.98E-01	639	9.40E-04	425	6.60E-01	979	7.94E-01	999	3.69E-02	642	4	6.68E-01	915	5.91E-01	1047	6.37E-01	766	6.63E-06	319	1.28E-02	620	7.35E-01	990	2.78E-03	40	3	7	601.75
23612	NM_012396	Phlda3	8.84E-01	1047	4.97E-02	496	4.25E-01	523	1.53E-01	655	3.26E-01	439	3.37E-02	671	5.36E-02	286	1.76E-02	151	5.23E-01	986	3	3.67E-01	700	2.28E-01	793	4.04E-01	466	1.66E-03	697	2.45E-02	689	5.10E-03	159	5.46E-01	871	3	6	601.81
1312	NM_004074	COX8A	1.56E-01	578	3.25E-02	367	2.71E-01	367	2.71E-01	748	6.29E-01	787	6.59E-05	300	8.98E-01	1102	8.38E-02	340	1.27E-01	771	2	9.40E-01	1117	2.10E-01	773	2.50E-01	209	1.13E-02	806	2.97E-04	354	6.60E-01	946	6.67E-03	72	3	5	602.31
5325	NM_002659	PLAUR	9.65E-01	1121	1.04E-01	546	2.29E-02	91	5.01E-01	882	9.77E-01	1129	1.85E-08	88	1.36E-02	123	2.88E-02	200	2.88E-01	877	4	1.73E-02	168	7.82E-01	1097	9.93E-01	1151	3.87E-01	1046	4.45E-09	26	5.89E-07	8	8.81E-01	1087	3	7	602.50
1E+05	NM_025077	toe1	3.39E-02	363	2.36E-01	657	8.54E-01	1017	1.14E-02	343	2.16E-01	291	3.30E-06	196	9.68E-02	412	9.40E-01	1104	1.83E-07	86	4	4.33E-01	757	3.96E-01	964	4.50E-01	543	1.34E-02	821	2.95E-02	715	2.22E-02	276	9.04E-01	1105	3	7	603.13
5819	NM_001122674	abcd3	8.36E-03	157	6.18E-01	894	7.16E-01	867	1.43E-01	641	9.89E-01	1140	1.43E-02	599	5.52E-01	920	3.54E-02	223	5.83E-06	162	4	2.97E-02	224	3.42E-01	921	5.82E-01	706	2.39E-05	415	4.19E-05	256	3.24E-01	707	4.76E-01	822	3	7	603.38
8497	NM_015053	Ppfia4	2.49E-01	646	8.71E-01	1073	3.78E-02	130	7.55E-01	1021	3.89E-01	507	6.17E-02	732	1.58E-01	514	7.00E-03	91	8.65E-01	1103	2	1.38E-02	148	4.59E-06	13	8.64E-01	991	3.11E-01	1024	2.82E-05	232	7.43E-01	993	9.55E-02	436	3	5	603.38
9689	NM_014670	BZW1	3.61E-02	382	2.55E-01	677	1.88E-01	290	3.59E-03	240	7.35E-01	890	6.06E-07	160	2.39E-01	639	5.11E-01	788	3.29E-06	141	4	5.63E-01	858	2.66E-01	838	8.95E-01	1025	2.20E-02	852	2.08E-03	459	7.35E-02	432	7.37E-01	992	2	6	603.94
11157	NM_007080	lsm6	1.35E-01	565	4.59E-02	464	8.95E-01	1065	2.16E-01	705	4.92E-01	636	2.09E-03	464	2.43E-01	645	3.77E-01	690	1.41E-06	119	3	5.69E-01	862	2.06E-02	445	2.57E-01	224	6.51E-03	764	1.04E-02	605	6.51E-01	939	1.16E-01	471	3	6	603.94
10209	XM_001132642	Eif1	1.56E-02	219	3.91E-02	414	7.98E-01	953	7.01E-01	995	3.29E-01	444	7.71E-07	163	2.91E-01	702	2.71E-01	593	8.62E-01	1101	3	2.74E-01	595	4.02E-02	531	6.32E-01	758	7.25E-04	654	2.50E-01	933	2.26E-04	63	1.61E-01	548	3	6	604.13
10667	NM_006567	FARS2	7.69E-01	957	2.32E-03	66	2.03E-01	306	5.08E-02	509	2.34E-01	320	2.42E-02	645	3.48E-01	752	5.18E-01	794	1.22E-01	767	2	7.92E-03	121	4.44E-01	987	4.19E-01	492	1.13E-02	807	2.79E-03	488	7.39E-01	992	2.80E-01	681	3	5	605.25
488	NM_001135765	ATP2A2	1.78E-01	592	6.40E-01	908	2.50E-01	354	2.58E-01	737	5.04E-01	643	2.63E-02	649	7.04E-03	91	8.33E-01	1026	1.06E-01	751	2	9.57E-02	375	1.96E-02	440	7.68E-01	901	1.80E-05	391	8.91E-01	1125	2.15E-02	271	9.43E-02	433	3	5	605.44

**1965**	**NM_001130678**	**eif4e**	3.28E-01	720	2.10E-02	265	8.91E-01	1060	3.67E-04	114	1.67E-01	232	2.77E-03	483	1.00E+00	1158	1.57E-02	141	3.67E-02	640	**5**	7.62E-01	990	1.18E-01	668	9.18E-01	1050	1.77E-07	112	9.86E-01	1153	8.76E-04	92	4.72E-01	820	**2**	**7**	**606.13**
**1457**	**NM_000100**	**Cstb**	3.42E-02	366	6.32E-01	903	3.31E-01	435	7.45E-01	1013	4.67E-01	599	5.47E-04	391	1.18E-02	115	2.18E-01	543	1.31E-05	180	**4**	3.03E-01	631	5.11E-01	1015	7.81E-01	916	6.74E-06	322	4.00E-02	747	3.45E-01	725	4.44E-01	800	**2**	**6**	**606.31**
**8402**	**NM_003562**	**Slc25a11**	5.77E-02	481	1.06E-02	173	5.02E-01	613	8.31E-03	313	2.05E-01	275	4.37E-01	1005	1.86E-01	562	6.15E-01	870	3.48E-01	911	**2**	1.01E-01	383	8.10E-04	173	7.96E-01	934	1.98E-01	991	5.26E-02	781	9.04E-01	1088	1.78E-02	159	**2**	**4**	**607.00**
**80221**	**NM_001115113**	**WDR26**	9.33E-01	1084	2.52E-01	671	1.82E-02	74	5.24E-01	902	3.94E-01	511	2.75E-03	482	2.44E-01	646	8.74E-02	343	6.14E-01	1022	**2**	3.93E-01	727	3.44E-01	923	3.24E-01	328	7.36E-08	73	1.17E-02	614	1.95E-01	586	3.50E-01	731	**2**	**4**	**607.31**
**5860**	**NM_002868**	**rab5b**	6.92E-01	903	7.70E-01	1003	1.82E-01	284	2.72E-02	417	6.17E-01	768	5.25E-07	157	3.11E-02	208	2.09E-02	163	4.31E-01	949	**4**	8.44E-01	1049	8.43E-02	619	6.91E-01	821	8.61E-03	786	1.41E-04	320	4.92E-02	377	5.85E-01	898	**3**	**7**	**607.63**
**54957**	**NM_017858**	**TIPIN**	2.29E-01	632	2.62E-01	681	4.19E-02	142	1.72E-04	82	6.32E-01	793	5.12E-01	1025	5.21E-01	899	2.89E-01	608	2.62E-02	602	**3**	3.79E-01	711	2.24E-01	791	4.32E-01	514	2.40E-06	257	9.79E-01	1152	6.88E-03	176	2.59E-01	658	**2**	**5**	**607.69**
**79158**	**NM_024334**	**Tmem43**	4.98E-03	117	8.57E-02	533	5.35E-01	661	5.21E-01	899	4.91E-01	635	1.93E-05	251	6.48E-01	973	3.07E-01	626	3.80E-02	645	**3**	1.45E-01	441	5.65E-01	1035	7.00E-01	829	1.51E-05	377	2.09E-02	673	1.10E-04	51	7.17E-01	979	**3**	**6**	**607.81**
**661**	**NM_001722**	**Polr3d**	9.31E-01	1081	2.04E-01	636	1.39E-03	20	5.67E-01	927	7.01E-01	867	6.36E-03	531	8.18E-01	1070	4.21E-02	249	2.44E-06	133	**4**	3.92E-01	724	1.33E-05	26	4.83E-01	583	8.10E-02	944	2.46E-02	690	6.72E-01	954	4.51E-02	294	**3**	**7**	**608.06**
**8799**	**NM_003846**	**PEX11B**	2.06E-02	258	5.55E-01	854	3.55E-01	461	2.42E-01	725	4.04E-02	81	9.56E-03	559	6.70E-01	986	2.47E-01	575	1.16E-01	761	**3**	7.88E-01	1008	2.60E-02	479	3.62E-01	397	5.77E-05	480	1.39E-03	446	2.19E-01	614	8.35E-01	1047	**3**	**6**	**608.19**
**397**	**NM_001175**	**arhgdib**	4.13E-02	417	6.45E-01	911	8.63E-01	1032	2.82E-02	420	9.33E-01	1082	1.94E-05	252	1.81E-01	549	1.39E-02	133	8.16E-02	724	**4**	7.44E-01	974	8.88E-01	1137	5.69E-01	695	1.18E-06	219	3.53E-04	357	3.21E-04	73	3.81E-01	757	**3**	**7**	**608.25**
**81619**	**NM_030935**	**TSC22D4**	1.89E-02	244	5.04E-01	828	6.94E-01	851	2.63E-02	412	1.32E-01	197	6.72E-02	740	2.84E-01	695	5.29E-04	28	2.96E-03	432	**4**	2.02E-01	515	2.33E-01	799	7.11E-01	840	4.62E-02	908	5.70E-02	790	3.28E-02	316	9.62E-01	1137	**2**	**6**	**608.25**
**1454**	**NM_001895**	**CSNK2A1P**	7.70E-01	958	1.17E-02	182	5.20E-01	641	2.09E-01	696	3.59E-01	476	4.80E-05	284	4.00E-01	809	9.46E-01	1110	8.69E-02	731	**2**	1.43E-01	439	5.48E-01	1028	3.23E-01	325	1.05E-04	521	9.53E-02	829	3.55E-03	140	1.70E-01	564	**2**	**4**	**608.31**
**9255**	**NM_001142415**	**AIMP1**	1.35E-01	564	1.63E-01	598	2.19E-01	326	1.21E-05	36	1.90E-01	258	5.99E-02	730	7.44E-01	1022	1.99E-01	522	2.25E-06	128	**2**	8.11E-01	1026	2.81E-01	854	9.14E-01	1042	1.83E-05	392	9.74E-01	1150	1.83E-01	575	1.40E-01	516	**1**	**3**	**608.69**
**92579**	**NM_138402**	**SP140L**	2.61E-01	657	2.36E-02	281	6.71E-01	820	1.25E-03	181	4.79E-01	617	1.35E-01	828	3.44E-01	749	2.59E-02	187	8.35E-01	1091	**3**	6.83E-04	42	2.61E-01	831	5.19E-01	634	1.61E-04	548	4.21E-02	752	5.10E-01	845	2.76E-01	677	**3**	**6**	**608.75**
**51009**	**NM_016041**	**Derl2**	4.62E-01	794	4.27E-02	441	3.45E-01	452	1.40E-01	639	4.07E-01	530	2.35E-04	355	1.31E-02	119	5.40E-01	810	2.77E-01	866	**3**	8.08E-01	1019	4.99E-01	1011	2.71E-01	248	1.04E-02	801	3.25E-01	961	2.73E-02	302	7.95E-02	397	**2**	**5**	**609.06**
**5880**	**NM_058216**	**RAD51C**	4.59E-02	448	5.07E-01	832	9.51E-01	1121	3.46E-02	450	9.74E-01	1123	4.11E-08	101	2.12E-01	603	5.05E-01	783	5.34E-01	992	**3**	1.00E+00	1157	8.39E-03	358	2.57E-01	225	5.17E-06	303	2.43E-02	688	8.85E-05	46	1.51E-01	527	**4**	**7**	**609.81**
**9467**	**NM_001018009**	**SH3BP5**	2.56E-02	311	3.26E-01	721	6.66E-01	814	7.35E-01	1008	7.96E-01	955	3.67E-01	967	1.87E-02	153	1.79E-02	153	9.63E-01	1147	**3**	2.72E-02	215	3.36E-04	119	6.01E-01	725	2.67E-01	1010	1.16E-04	315	2.01E-02	268	5.57E-01	876	**4**	**7**	**609.81**
**10902**	**NM_001164326**	**BRD8**	4.68E-01	796	1.08E-02	174	8.72E-01	1038	6.85E-02	542	9.90E-01	1146	8.93E-01	1131	2.03E-02	166	5.64E-02	279	4.10E-01	937	**2**	2.70E-01	589	3.47E-03	278	1.44E-01	93	4.74E-04	626	3.34E-01	962	3.78E-01	753	3.53E-02	251	**3**	**5**	**610.06**
**54520**	**NM_019044**	**Ccdc93**	2.93E-02	335	2.52E-01	670	6.77E-01	826	1.38E-01	633	3.62E-02	73	8.27E-02	769	6.94E-01	996	2.08E-02	161	1.42E-01	782	**3**	4.14E-01	742	2.44E-01	812	4.34E-01	520	1.29E-01	967	2.75E-05	231	3.26E-01	708	1.56E-01	540	**1**	**4**	**610.31**
**55166**	**NM_018141**	**mrps10**	8.25E-01	1008	3.28E-01	725	3.00E-02	111	1.53E-01	654	2.42E-01	334	2.02E-03	461	8.01E-01	1057	7.03E-01	937	2.01E-01	830	**2**	3.59E-03	87	2.94E-02	495	4.11E-01	478	1.17E-03	681	9.96E-04	424	9.70E-01	1144	5.64E-02	350	**4**	**6**	**611.00**
**8611**	**NM_176895**	**PPAP2A**	4.92E-01	810	9.22E-01	1108	7.45E-03	51	6.54E-01	971	2.45E-01	338	5.57E-02	724	5.47E-03	80	1.07E-01	380	1.18E-01	763	**2**	6.52E-01	905	8.06E-01	1108	1.61E-01	111	1.31E-06	227	3.05E-02	717	5.42E-01	863	2.22E-01	627	**2**	**4**	**611.44**
**3308**	**NM_005347**	**HSPA5**	9.59E-01	1112	1.53E-01	590	4.33E-02	147	6.15E-06	26	8.34E-01	989	3.78E-02	684	8.28E-02	386	3.15E-01	635	1.79E-02	573	**4**	1.90E-01	505	2.93E-01	878	7.56E-01	888	5.69E-03	756	6.41E-01	1061	4.53E-02	367	2.24E-02	189	**3**	**7**	**611.63**
**54780**	**NM_017646**	**TRIT1**	7.22E-03	143	1.43E-01	579	9.74E-01	1139	2.16E-01	703	3.20E-01	433	1.65E-04	339	5.34E-01	909	5.55E-01	827	6.28E-01	1028	**2**	5.05E-01	813	1.41E-01	704	1.38E-01	89	2.27E-05	410	5.49E-08	55	9.57E-01	1132	1.20E-01	484	**2**	**4**	**611.69**
**2617**	**NM_175085**	**GART**	1.13E-02	183	3.37E-01	732	2.51E-01	357	4.96E-01	879	7.59E-01	911	6.48E-01	1064	1.10E-02	110	7.55E-01	974	9.32E-03	524	**3**	3.31E-01	659	9.50E-02	637	6.35E-01	762	8.20E-06	336	2.65E-05	229	4.56E-01	811	2.16E-01	621	**2**	**5**	**611.81**
**79004**	**NM_024045**	**DDX50**	2.57E-02	312	2.57E-01	679	7.11E-01	861	2.74E-02	418	4.63E-01	595	3.29E-04	373	1.73E-01	539	6.82E-01	920	9.62E-01	1145	**3**	1.37E-01	434	2.56E-05	32	8.09E-01	948	6.22E-02	926	2.10E-02	675	2.40E-01	628	4.75E-02	304	**3**	**6**	**611.81**
**23327**	**NM_001144969**	**Nedd4l**	5.42E-03	124	6.50E-01	917	3.07E-01	418	4.73E-03	260	8.62E-02	149	2.81E-02	655	1.12E-01	441	3.11E-01	632	1.25E-05	176	**4**	9.87E-01	1148	1.93E-01	753	7.82E-01	917	6.21E-04	647	2.81E-01	941	1.38E-01	522	8.88E-01	1090	**1**	**5**	**611.88**
**81**	**NM_004924**	**actn4**	2.23E-01	626	1.84E-02	245	8.10E-01	967	1.74E-02	371	6.86E-01	849	1.85E-05	249	1.46E-01	493	3.47E-01	663	1.48E-01	784	**3**	3.69E-03	88	2.80E-01	853	2.73E-01	251	7.52E-01	1129	7.24E-01	1086	7.15E-01	975	1.82E-02	162	**2**	**5**	**611.94**
**79666**	**NM_024624**	**SMC6**	8.17E-01	999	1.97E-02	256	3.00E-01	405	2.51E-01	733	8.28E-01	987	7.18E-06	214	9.64E-01	1140	2.60E-01	582	2.06E-06	126	**3**	4.27E-01	751	2.39E-03	246	7.32E-01	858	7.00E-02	935	6.76E-01	1069	5.25E-02	387	9.22E-03	103	**2**	**5**	**611.94**
**4053**	**NM_203364**	**Caprin1**	2.12E-02	270	4.60E-01	804	9.15E-01	1082	5.82E-02	529	1.55E-01	221	9.28E-07	167	8.74E-05	6	4.85E-01	774	2.60E-04	291	**4**	8.55E-01	1058	2.90E-01	869	7.41E-01	866	1.42E-01	970	3.55E-01	967	2.99E-05	30	5.76E-01	890	**1**	**5**	**612.13**
**26230**	**NM_001010927**	**TIAM2**	8.28E-01	1009	8.04E-03	143	2.94E-01	395	1.21E-01	614	2.15E-01	289	5.43E-02	722	3.10E-01	719	3.85E-02	228	2.07E-02	579	**3**	2.85E-01	609	5.37E-01	1026	7.73E-01	907	6.32E-01	1101	2.83E-01	942	4.96E-02	379	1.35E-02	134	**2**	**5**	**612.25**
**1793**	**NM_001165031**	**dtymk**	9.62E-01	1116	5.81E-03	114	4.22E-01	520	3.82E-03	243	4.25E-01	552	5.89E-05	294	6.15E-02	320	6.18E-01	872	7.41E-01	1059	**3**	5.87E-01	873	3.61E-01	935	1.92E-01	148	3.44E-02	889	2.35E-03	473	1.59E-01	543	5.10E-01	846	**2**	**5**	**612.31**
**9867**	**NM_014819**	**pja2**	3.72E-01	744	9.77E-02	540	4.42E-03	31	9.10E-01	1109	6.71E-01	833	2.44E-01	895	3.21E-01	728	7.05E-01	940	7.51E-03	502	**2**	4.76E-01	795	3.85E-01	954	4.51E-01	545	1.44E-06	231	8.85E-07	110	2.67E-01	657	2.21E-02	187	**3**	**5**	**612.56**
**55127**	**NM_018075**	**Ano10**	4.48E-02	442	2.87E-01	692	8.05E-01	963	2.53E-02	408	2.00E-01	265	3.66E-01	966	5.11E-01	887	4.77E-02	267	2.53E-02	599	**4**	6.70E-03	110	2.23E-01	784	5.59E-01	683	2.71E-07	133	7.58E-01	1097	6.72E-01	953	1.63E-01	552	**2**	**6**	**612.56**
**55975**	**NM_019116**	**ubfd1**	2.74E-01	667	3.00E-01	703	4.99E-02	162	2.33E-02	401	3.67E-01	483	7.10E-01	1087	8.00E-01	1056	5.07E-01	786	7.82E-04	342	**3**	3.80E-01	712	1.17E-01	666	8.72E-01	1000	8.03E-05	504	2.62E-05	228	1.63E-02	243	3.89E-01	763	**3**	**6**	**612.69**
**1912**	**NM_182685**	**EFNA1**	1.22E-03	56	9.07E-01	1097	5.15E-01	632	1.14E-06	10	5.52E-01	689	1.45E-10	36	9.63E-01	1139	1.48E-01	448	9.50E-01	1137	**3**	4.68E-01	786	4.38E-01	984	5.49E-01	667	1.21E-05	363	2.72E-05	230	7.98E-01	1031	1.33E-01	501	**2**	**5**	**612.88**
**1371**	**NM_134442**	**CREB1**	2.56E-01	651	3.30E-02	368	7.60E-02	191	1.60E-01	661	8.75E-01	1026	9.36E-03	555	8.02E-01	1060	9.82E-01	1145	5.80E-06	161	**3**	4.72E-01	789	1.88E-03	229	3.84E-01	438	5.84E-06	308	4.57E-01	1009	5.74E-04	83	9.58E-01	1134	**3**	**6**	**613.00**
**3624**	**NM_002194**	**INPP1**	2.66E-03	81	1.54E-01	592	3.94E-01	492	7.15E-01	999	2.16E-01	293	3.44E-01	949	5.52E-03	81	7.20E-01	950	8.93E-02	734	**2**	8.29E-01	1042	1.22E-03	202	3.91E-01	452	5.16E-02	912	1.19E-01	860	1.48E-03	107	8.50E-01	1062	**2**	**4**	**613.00**
**9183**	**NM_004724**	**ZW10**	2.43E-01	642	1.22E-02	190	6.19E-03	47	6.38E-01	963	9.40E-01	1087	7.26E-02	749	4.54E-01	850	9.70E-02	362	3.18E-05	205	**3**	8.10E-01	1022	2.22E-01	783	4.41E-02	27	4.85E-04	629	9.27E-03	587	6.29E-01	925	3.68E-01	747	**3**	**6**	**613.44**
**1994**	**NM_005229**	**elk1**	8.62E-01	1028	1.96E-02	255	2.19E-01	327	4.14E-01	839	4.95E-01	637	7.31E-03	539	5.42E-02	287	5.63E-01	831	4.52E-08	69	**3**	3.49E-02	240	2.99E-01	881	7.29E-01	854	9.15E-01	1151	5.22E-04	376	6.58E-01	945	1.70E-01	563	**2**	**5**	**613.88**
**9878**	**NM_014828**	**Tox4**	5.68E-01	843	1.67E-01	603	5.61E-02	168	8.17E-02	562	6.56E-01	813	9.50E-02	782	9.73E-02	413	1.04E-01	373	1.22E-02	547	**1**	2.49E-01	569	1.60E-01	713	5.85E-01	709	7.48E-01	1128	1.65E-01	893	4.51E-04	75	2.26E-01	631	**1**	**2**	**613.88**
**7320**	**NM_003337**	**ube2b**	3.22E-01	714	1.33E-01	572	8.63E-01	1031	1.64E-01	665	8.75E-02	152	3.81E-08	97	7.23E-03	93	1.11E-01	386	3.94E-02	651	**3**	3.75E-01	707	3.99E-02	530	7.63E-01	895	1.27E-01	965	2.11E-01	915	6.68E-02	420	8.23E-01	1035	**1**	**4**	**614.25**
**22907**	**NM_138615**	**DHX30**	1.41E-01	571	1.62E-01	597	4.63E-01	565	2.22E-01	710	8.01E-01	961	4.62E-03	516	2.32E-01	627	3.46E-01	662	3.81E-02	647	**2**	1.58E-01	461	1.38E-02	401	5.60E-01	684	6.59E-03	767	2.95E-03	494	4.51E-01	806	6.10E-02	362	**3**	**5**	**614.44**
**7319**	**NM_181777**	**UBE2A**	2.37E-03	77	8.74E-01	1078	9.04E-01	1075	1.11E-01	602	5.93E-01	742	1.65E-05	241	9.13E-01	1114	1.92E-01	519	4.34E-03	465	**3**	3.65E-02	249	3.81E-03	290	4.29E-01	510	9.28E-01	1154	5.22E-02	779	4.32E-01	791	1.70E-02	148	**3**	**6**	**614.63**
**3609**	**NM_005537**	**ing1**	2.53E-02	309	1.81E-02	242	9.09E-01	1078	1.65E-01	668	5.99E-01	748	1.13E-01	805	1.25E-01	461	3.21E-01	638	4.47E-09	45	**3**	3.14E-01	643	1.72E-02	421	9.82E-01	1138	1.27E-03	685	4.87E-01	1020	3.35E-01	715	2.86E-02	224	**3**	**6**	**615.00**
**6709**	**NM_003130**	**SRI**	2.15E-02	274	3.66E-01	746	7.47E-01	896	9.85E-03	331	6.41E-01	799	2.25E-01	882	2.07E-02	169	7.19E-01	949	1.63E-06	120	**4**	3.51E-01	679	1.70E-01	722	4.97E-01	606	4.59E-03	744	2.76E-02	705	1.55E-01	535	2.85E-01	684	**2**	**6**	**615.06**
**81847**	**NM_031206**	**LAS1L**	8.90E-01	1053	1.55E-02	218	7.18E-01	869	4.12E-01	838	9.08E-01	1057	6.69E-03	534	6.16E-02	321	1.43E-01	440	1.37E-01	777	**2**	7.21E-02	333	4.07E-02	534	7.52E-01	880	1.37E-04	539	1.35E-06	124	1.68E-01	555	3.99E-01	770	**3**	**5**	**615.13**
**5111**	**NM_002600**	**Pde4b**	8.39E-01	1013	2.60E-02	307	6.00E-01	738	4.57E-01	857	2.39E-01	329	3.89E-02	689	5.28E-01	907	9.23E-01	1094	5.27E-10	26	**3**	5.59E-02	296	5.12E-03	315	6.01E-01	724	9.83E-06	343	2.43E-03	474	2.73E-01	663	8.62E-01	1072	**3**	**6**	**615.44**
**51760**	**NM_016524**	**Syt7**	2.48E-02	303	5.34E-01	845	6.46E-01	785	8.47E-03	315	2.15E-01	288	9.08E-02	779	1.97E-01	580	3.10E-01	631	2.99E-01	884	**2**	4.02E-01	733	5.72E-01	1037	9.48E-01	1096	9.79E-07	203	1.42E-03	447	4.22E-02	358	1.70E-01	565	**3**	**5**	**615.56**
**8614**	**NM_003714**	**STC2**	9.67E-01	1124	3.01E-02	346	8.77E-01	1048	7.49E-03	308	4.78E-01	613	9.95E-01	1156	4.17E-02	243	5.86E-01	848	9.33E-01	1129	**3**	5.95E-03	108	2.06E-02	443	2.02E-01	161	5.17E-01	1076	4.62E-08	53	3.12E-01	692	1.35E-01	503	**3**	**6**	**615.69**
**91137**	**NM_138774**	**c19orf22**	2.02E-02	255	4.93E-01	821	9.06E-01	1077	6.22E-01	956	5.65E-01	707	7.22E-06	215	7.00E-01	998	3.47E-03	67	8.58E-03	511	**4**	6.98E-01	938	6.29E-02	576	5.05E-01	618	1.94E-06	248	2.31E-05	220	2.20E-01	616	8.15E-01	1030	**2**	**6**	**615.81**
**6635**	**NM_003095**	**SNRPF**	8.75E-01	1040	4.83E-03	102	6.61E-01	806	7.66E-01	1028	5.57E-01	696	1.46E-05	235	7.80E-02	375	7.65E-01	982	2.10E-03	409	**3**	4.42E-01	767	2.30E-03	243	9.50E-01	1102	4.19E-05	452	4.13E-02	750	2.44E-01	633	3.11E-02	237	**4**	**7**	**616.06**
**4205**	**NM_004528**	**Mgst3**	4.71E-01	799	5.55E-03	111	2.14E-01	323	6.85E-01	986	7.41E-01	895	8.68E-01	1124	5.42E-01	916	4.81E-01	769	1.29E-02	555	**2**	4.04E-02	260	2.54E-04	108	4.96E-01	605	2.16E-05	406	2.78E-02	706	6.33E-02	411	5.65E-01	884	**4**	**6**	**616.13**
**2971**	**NM_001161587**	**GYS1**	2.02E-01	611	9.23E-03	159	7.39E-01	888	1.39E-03	189	9.89E-01	1141	4.28E-04	384	5.62E-01	927	2.88E-01	607	6.16E-01	1024	**3**	5.45E-01	850	5.50E-01	1029	1.07E-01	55	4.86E-06	297	6.73E-01	1067	9.57E-03	193	9.66E-02	439	**2**	**5**	**616.25**
**64175**	**NM_176871**	**PDLIM2**	6.93E-01	904	1.73E-01	609	4.44E-02	149	6.97E-01	993	7.62E-01	913	3.03E-05	271	1.17E-01	449	5.20E-01	795	2.75E-02	606	**3**	2.87E-02	218	2.03E-01	763	6.12E-01	739	2.30E-02	857	9.45E-03	589	1.11E-01	487	1.42E-01	518	**3**	**6**	**616.25**
**57175**	**NM_020338**	**Zmiz1**	2.21E-02	283	9.07E-01	1098	6.65E-01	809	7.37E-03	304	8.15E-01	972	1.21E-01	810	3.49E-01	757	1.69E-02	146	1.70E-03	401	**4**	3.56E-01	690	7.53E-01	1092	5.76E-01	699	2.35E-06	256	4.08E-01	985	2.44E-02	289	3.88E-02	270	**3**	**7**	**616.31**
**10236**	**NM_001102399**	**HNRNPR**	2.99E-01	701	4.65E-02	470	4.84E-01	593	8.56E-02	570	6.63E-01	825	8.47E-05	314	2.48E-01	653	6.75E-01	908	1.12E-05	174	**3**	2.54E-02	208	7.47E-02	599	4.30E-01	512	1.35E-05	371	7.60E-01	1098	9.14E-01	1098	3.96E-01	768	**2**	**5**	**616.38**
**8775**	**NM_003827**	**napA**	4.95E-02	467	9.78E-02	541	5.45E-01	671	1.37E-02	356	7.74E-02	139	4.78E-01	1015	9.30E-01	1120	8.07E-01	1012	5.92E-05	233	**3**	3.46E-03	84	2.54E-03	249	6.23E-01	749	7.83E-01	1134	1.19E-02	617	3.36E-01	717	3.84E-01	761	**3**	**6**	**616.56**
**7266**	**NM_003315**	**DNAJC7**	3.69E-02	386	7.22E-01	970	9.30E-01	1099	7.37E-01	1010	7.22E-03	17	4.98E-06	204	9.24E-02	404	4.04E-01	721	1.78E-01	813	**3**	4.51E-01	773	3.92E-01	959	1.78E-01	133	8.13E-03	783	2.05E-01	911	4.33E-02	363	5.13E-02	324	**2**	**5**	**616.88**
**10577**	**NM_006432**	**npc2**	1.35E-02	202	6.60E-01	923	9.70E-01	1137	2.33E-03	206	8.91E-01	1044	1.42E-04	332	3.66E-03	64	6.13E-01	867	6.38E-01	1030	**4**	8.74E-01	1072	9.74E-04	184	4.94E-01	600	3.65E-08	43	7.30E-01	1087	1.04E-02	200	5.63E-01	881	**3**	**7**	**617.00**
**7750**	**NM_197968**	**zmym2**	6.57E-01	886	2.47E-02	294	7.23E-02	185	6.64E-03	290	8.08E-01	967	3.59E-01	958	9.03E-02	401	7.36E-02	320	3.64E-01	921	**2**	2.25E-01	533	1.76E-01	729	8.45E-01	977	5.02E-03	749	3.87E-01	977	2.33E-02	285	8.25E-02	409	**2**	**4**	**617.56**
**8459**	**NM_001008566**	**TPST2**	2.22E-02	284	3.76E-01	752	6.54E-01	796	8.63E-01	1081	7.68E-01	921	1.30E-01	820	6.16E-02	323	5.78E-01	839	2.20E-05	195	**2**	3.92E-01	725	2.68E-03	254	5.35E-01	649	1.07E-01	957	6.82E-03	564	9.46E-06	19	3.18E-01	707	**3**	**5**	**617.88**
**26586**	**NM_001098525**	**Ckap2**	2.18E-01	620	3.47E-02	382	1.61E-01	262	6.63E-01	973	3.19E-01	431	2.94E-01	925	2.66E-01	678	1.83E-01	505	9.04E-06	172	**2**	7.10E-01	944	9.53E-02	638	4.70E-01	574	4.22E-04	615	3.70E-03	508	1.80E-01	571	8.86E-01	1089	**2**	**4**	**617.94**
**6790**	**NM_005990**	**STK10**	9.20E-01	1068	3.30E-02	369	7.37E-01	886	8.23E-01	1063	5.36E-01	667	1.07E-06	172	3.25E-02	214	8.70E-01	1049	3.52E-04	302	**4**	3.47E-01	677	1.29E-02	392	5.05E-01	619	6.41E-06	318	1.36E-06	126	6.35E-01	929	8.29E-01	1040	**3**	**7**	**618.19**
**1075**	**NM_001270**	**CHD1**	3.00E-03	89	3.46E-01	734	8.42E-01	1006	2.00E-05	46	7.39E-01	894	4.14E-03	510	3.41E-01	743	6.42E-01	888	3.61E-01	916	**3**	3.23E-02	233	2.74E-01	848	4.18E-01	491	1.82E-02	837	6.91E-01	1078	4.20E-06	12	1.73E-01	569	**3**	**6**	**618.38**
**3326**	**NM_199440**	**HSPD1**	9.56E-01	1111	2.01E-02	259	3.39E-01	444	8.88E-01	1094	1.49E-02	27	1.87E-03	459	2.61E-01	669	9.49E-01	1114	7.39E-04	336	**4**	6.88E-03	112	8.78E-01	1131	6.54E-01	781	1.30E-01	968	1.44E-01	877	3.82E-03	148	6.31E-02	364	**2**	**6**	**618.38**
**10559**	**NM_001168398**	**SLC35A1**	2.60E-02	315	2.78E-01	688	9.65E-01	1133	9.46E-01	1128	2.76E-01	383	6.57E-01	1066	3.55E-01	765	9.46E-01	1109	3.93E-02	650	**2**	5.13E-03	104	5.12E-02	557	4.17E-01	489	1.36E-07	105	1.76E-06	132	6.88E-01	964	4.94E-02	314	**4**	**6**	**618.88**
**7204**	**NM_007118**	**TRIO**	1.65E-01	584	1.45E-02	206	4.39E-01	541	8.71E-01	1085	1.48E-01	214	3.11E-01	933	2.41E-01	641	1.40E-01	436	2.59E-01	855	**1**	2.42E-01	559	3.52E-01	928	3.06E-01	303	8.79E-03	788	1.00E-05	197	2.79E-01	671	6.90E-01	963	**2**	**3**	**619.00**
**7040**	**NM_000358**	**tgfbi**	7.39E-01	935	2.64E-02	313	8.25E-01	989	1.28E-01	624	2.18E-01	296	1.86E-07	129	5.76E-01	935	4.79E-03	79	9.25E-01	1123	**3**	3.62E-01	694	1.96E-03	230	8.85E-01	1016	1.39E-04	541	1.42E-01	874	1.18E-04	54	8.62E-01	1073	**3**	**6**	**619.06**
**4817**	**NM_006170**	**nop2**	1.55E-01	577	5.51E-01	852	7.85E-01	935	9.01E-02	578	1.96E-01	261	8.54E-06	218	5.89E-01	941	6.94E-01	928	2.78E-02	609	**2**	1.12E-01	398	1.50E-03	217	7.81E-01	914	5.84E-01	1093	2.82E-02	708	2.05E-01	601	6.94E-03	76	**3**	**5**	**619.13**
**80346**	**NM_030790**	**ITFG1**	7.77E-02	507	1.62E-02	226	7.61E-01	912	1.39E-01	637	2.08E-01	279	2.36E-02	642	3.41E-01	746	5.11E-01	787	1.22E-02	546	**3**	4.61E-01	779	1.61E-01	715	9.88E-01	1145	8.80E-04	660	1.68E-08	41	1.19E-02	204	8.67E-01	1080	**3**	**6**	**619.13**
**5717**	**NM_002818**	**PSME2**	2.67E-01	661	3.19E-03	76	2.76E-01	371	3.75E-01	816	5.39E-01	673	1.67E-04	340	4.14E-01	821	5.52E-01	825	3.04E-01	885	**2**	1.90E-02	181	3.09E-01	894	4.92E-01	596	1.16E-04	527	2.82E-03	489	5.70E-01	882	5.75E-01	888	**3**	**5**	**620.31**
**10865**	**NM_212481**	**Arid5a**	8.72E-01	1035	1.77E-02	237	6.22E-01	757	3.13E-01	778	7.08E-02	131	5.37E-01	1029	2.08E-01	595	8.75E-01	1052	4.20E-03	462	**2**	5.09E-01	818	1.34E-03	208	1.86E-01	144	7.37E-03	774	6.79E-01	1071	8.91E-03	190	2.44E-01	644	**3**	**5**	**620.31**
**26984**	**NM_012430**	**SEC22A**	1.47E-01	575	3.19E-02	362	3.49E-01	455	7.53E-01	1019	5.32E-01	663	6.19E-02	733	6.66E-01	981	2.43E-01	571	9.22E-03	523	**2**	4.12E-01	741	7.02E-01	1075	9.95E-02	50	2.82E-04	588	4.50E-04	371	9.69E-03	195	8.06E-01	1025	**3**	**5**	**620.44**
**51154**	**NM_016183**	**mrto4**	3.22E-01	715	1.94E-02	253	3.45E-01	451	1.65E-02	367	3.36E-01	451	3.32E-02	670	6.79E-01	988	7.11E-01	943	9.58E-01	1141	**3**	4.89E-01	805	1.07E-01	653	5.60E-01	685	2.20E-08	34	4.17E-01	989	2.60E-01	653	1.31E-02	129	**2**	**5**	**620.44**
**6687**	**NM_003127**	**SPTAN1**	4.43E-01	782	2.57E-02	304	4.66E-01	572	1.30E-03	183	1.24E-01	191	1.43E-02	600	8.61E-01	1085	3.58E-01	674	2.81E-01	871	**3**	1.84E-02	175	5.87E-02	570	9.22E-01	1058	5.31E-02	916	1.53E-05	212	6.77E-01	956	4.27E-01	789	**2**	**5**	**621.13**
**8729**	**NM_004193**	**GBF1**	2.35E-02	293	3.74E-01	750	5.79E-01	710	4.07E-03	247	1.72E-01	235	8.00E-02	758	2.87E-01	699	7.46E-01	966	4.51E-01	959	**2**	1.74E-01	481	3.66E-03	288	7.12E-01	842	2.58E-01	1008	4.65E-02	767	3.39E-01	718	2.82E-02	218	**3**	**5**	**621.19**
**54069**	**NM_018944**	**C21orf45**	3.87E-01	750	1.76E-02	236	5.95E-01	732	9.30E-01	1122	4.33E-02	89	3.95E-01	977	6.31E-03	87	1.74E-01	489	2.88E-03	429	**4**	5.93E-01	875	1.81E-01	736	4.56E-01	555	1.30E-03	687	6.64E-03	560	5.64E-01	880	3.59E-01	739	**2**	**6**	**621.44**
**4154**	**NM_002388**	**MCM3**	1.95E-01	605	2.53E-01	673	5.44E-01	670	1.64E-02	366	9.68E-01	1115	7.93E-08	114	6.60E-02	336	9.19E-01	1089	9.85E-01	1154	**2**	1.89E-02	179	3.08E-01	891	7.19E-01	849	9.24E-03	793	1.69E-03	454	3.50E-02	326	5.22E-02	330	**4**	**6**	**621.50**
**5526**	**NM_002720**	**PPP4C**	4.57E-01	791	1.94E-01	628	1.23E-02	66	5.13E-01	893	3.58E-01	474	8.30E-01	1113	2.55E-01	661	5.56E-01	828	7.16E-03	498	**2**	8.88E-02	364	6.11E-01	1052	1.72E-01	124	1.88E-02	841	6.25E-09	29	6.94E-01	966	2.15E-01	620	**2**	**4**	**621.75**

3399	NM_005896	IDH1	1.25E-01	558	1.98E-03	63	5.71E-01	700	4.68E-01	866	5.66E-01	708	2.32E-02	641	1.32E-01	471	3.70E-01	682	2.01E-01	827	2	5.09E-01	819	8.20E-05	67	5.59E-01	680	4.92E-01	1070	2.97E-06	154	7.92E-01	1026	2.14E-01	618	2	4	621.88
2E+05	NM_002936	RNASEH1	2.50E-01	647	9.03E-01	1093	5.71E-04	12	8.86E-01	1092	9.99E-01	1157	3.05E-10	46	3.59E-01	770	2.38E-03	55	4.13E-02	656	4	2.44E-01	561	6.56E-01	1067	6.43E-01	774	9.26E-01	1153	4.74E-03	530	2.21E-02	274	6.45E-03	69	3	7	622.25
9908	NM_012297	G3BP2	1.78E-01	593	4.63E-01	805	9.42E-01	1112	3.42E-01	795	6.11E-01	761	1.07E-02	569	2.95E-02	201	5.66E-01	834	1.51E-04	271	3	3.89E-01	719	6.25E-03	332	9.26E-01	1064	1.92E-06	246	5.03E-04	374	2.96E-01	680	2.00E-01	601	3	6	622.31
9939	NM_005105	RBM8A	2.40E-02	297	7.83E-01	1013	5.74E-01	705	1.23E-02	347	2.35E-01	321	2.91E-02	660	2.31E-02	178	6.42E-01	887	1.89E-01	819	4	3.66E-01	698	5.26E-03	319	9.17E-01	1049	4.89E-02	910	8.44E-02	819	4.33E-01	793	1.55E-02	145	3	7	622.50
55758	NM_018297	ngly1	4.41E-02	438	2.70E-01	684	3.90E-01	489	2.32E-01	718	7.23E-01	882	4.89E-01	1018	2.73E-02	189	3.08E-01	629	1.30E-04	263	3	2.39E-01	554	3.70E-01	944	7.67E-01	900	3.38E-02	888	6.25E-06	180	3.84E-01	759	9.05E-02	425	2	5	622.50
55111	NM_018072	HEATR1	2.93E-02	334	7.45E-01	989	4.33E-01	534	1.35E-03	187	9.72E-01	1120	1.29E-04	329	7.00E-01	999	9.48E-01	1113	5.69E-02	688	3	5.36E-04	32	2.68E-02	482	7.20E-01	850	3.82E-03	737	2.44E-07	79	7.91E-01	1024	1.11E-01	466	4	7	622.69
23062	NM_015044	Gga2	1.40E-01	570	6.04E-01	884	3.43E-01	448	3.08E-02	433	6.98E-01	863	7.59E-05	309	2.46E-01	650	7.44E-03	94	4.52E-01	962	3	7.95E-01	1012	7.94E-02	611	5.80E-01	705	8.41E-02	947	4.31E-03	521	2.31E-02	282	2.80E-01	682	2	5	623.31
10606	NM_001079524	paics	3.58E-02	378	2.48E-01	667	8.88E-02	198	2.97E-01	763	8.50E-01	1004	2.60E-02	646	8.77E-02	394	8.52E-01	1035	6.26E-04	327	3	2.59E-01	576	1.02E-03	187	5.26E-01	645	3.88E-01	1047	2.06E-01	912	6.73E-03	173	7.96E-01	1023	2	5	623.44
27069	NM_014394	GHITM	2.26E-01	629	2.72E-01	685	8.75E-01	1042	2.46E-01	730	7.94E-02	140	5.92E-06	209	5.81E-01	938	3.10E-02	205	8.07E-03	508	3	4.80E-01	800	7.20E-01	1083	1.99E-01	155	3.31E-03	729	3.19E-03	499	7.76E-01	1013	2.07E-01	611	2	5	623.50
7518	NM_022550	Xrcc4	3.36E-01	724	6.88E-01	945	2.88E-01	388	3.26E-02	442	3.18E-01	429	3.32E-03	498	9.08E-02	402	6.01E-01	860	6.97E-02	707	2	3.40E-02	237	1.36E-01	696	9.31E-01	1071	2.55E-04	585	2.31E-01	928	4.97E-02	380	2.86E-01	685	3	5	623.56
54918	NM_017812	CHCHD3	6.40E-04	38	3.05E-01	707	2.48E-01	351	7.77E-01	1033	6.14E-01	764	4.58E-06	201	1.10E-01	440	8.02E-01	1005	4.87E-05	225	3	6.45E-01	903	6.43E-01	1062	8.84E-01	1014	2.64E-05	421	7.81E-01	1103	3.03E-03	132	1.80E-01	579	2	5	623.63
22974	NM_012112	Tpx2	9.61E-01	1115	2.84E-02	328	7.88E-01	941	1.34E-01	632	9.25E-01	1072	5.74E-06	208	5.66E-02	301	9.00E-01	1073	1.91E-06	124	3	8.51E-01	1055	2.04E-03	232	4.16E-01	487	6.06E-04	645	2.30E-07	77	2.12E-01	607	8.83E-01	1088	3	6	624.06
1389	NM_139013	Mapk14	1.88E-04	27	2.77E-01	687	6.67E-01	815	5.20E-01	897	2.66E-01	366	3.22E-07	144	8.39E-01	1078	7.89E-01	995	2.60E-02	601	3	2.38E-01	553	5.10E-03	314	3.61E-01	396	7.44E-01	1127	3.51E-06	161	4.11E-01	778	8.37E-01	1048	2	5	624.19
23218	NM_015175	NBEAL2	4.33E-02	433	2.62E-01	682	7.92E-01	946	4.05E-01	835	2.22E-01	299	9.62E-02	785	8.41E-01	1079	3.96E-01	709	1.30E-06	116	2	2.41E-01	557	4.80E-05	48	8.08E-01	946	2.86E-04	589	4.79E-01	1016	4.86E-05	34	6.16E-01	921	3	5	624.69
22898	NM_014957	DENND3	2.95E-03	87	7.61E-01	997	6.79E-01	831	4.32E-01	844	3.15E-01	425	4.17E-03	511	2.76E-02	190	9.45E-01	1108	7.85E-01	1075	3	2.63E-01	582	3.24E-01	907	5.37E-01	652	1.02E-05	347	6.64E-01	1064	1.62E-02	242	1.38E-02	135	3	6	624.81
55768	NM_001146688	KDM3A	9.40E-03	166	5.38E-01	847	9.23E-01	1089	8.52E-01	1077	3.48E-01	464	4.28E-01	997	1.38E-02	125	5.61E-04	30	1.07E-02	536	4	1.04E-03	49	5.46E-02	566	6.88E-01	817	4.49E-01	1058	8.33E-01	1111	3.54E-02	330	3.55E-01	736	2	6	624.88
985	NM_001254	cdc6	6.44E-01	876	2.44E-02	286	5.80E-01	712	1.34E-02	353	5.35E-01	666	2.28E-02	637	1.40E-02	126	5.90E-01	851	1.96E-01	824	4	6.40E-01	900	2.25E-03	241	3.92E-01	454	4.02E-01	1050	1.03E-02	602	6.07E-01	907	1.39E-01	514	2	6	624.94
23189	NM_153186	KANK1	9.12E-01	1062	3.22E-03	78	5.70E-01	697	2.57E-03	216	2.33E-01	318	6.60E-09	70	5.03E-01	883	2.28E-02	172	2.74E-01	864	4	2.65E-01	583	4.02E-02	532	6.07E-01	733	4.67E-01	1064	6.14E-02	797	5.43E-01	866	8.59E-01	1067	1	5	625.13
1E+05	NM_022073	egln3	6.37E-03	136	8.04E-01	1026	7.42E-01	891	6.76E-02	540	4.55E-01	585	1.43E-02	598	7.55E-02	363	1.12E-01	389	1.47E-03	389	3	4.54E-01	775	8.06E-01	1109	9.33E-01	1075	1.24E-03	683	5.46E-01	1037	3.87E-02	343	5.69E-03	63	3	6	625.13
8473	NM_181673	Ogt	1.03E-01	532	9.59E-01	1129	5.27E-01	650	2.97E-03	228	1.75E-01	238	2.07E-01	870	2.36E-02	180	7.63E-01	980	2.18E-03	413	3	1.82E-02	174	1.06E-01	651	5.91E-01	713	6.98E-01	1118	9.95E-01	1158	1.21E-01	504	1.09E-01	465	1	4	625.19
23660	NM_014569	Zkscan5	3.29E-01	721	9.66E-03	161	4.42E-01	545	3.66E-01	812	2.42E-01	333	7.75E-01	1103	5.26E-01	902	1.28E-01	412	5.64E-01	1001	1	8.16E-01	1029	8.89E-02	626	2.92E-01	279	4.63E-03	745	2.01E-02	667	2.56E-01	648	1.64E-03	24	3	4	625.50
23328	NM_015278	SASH1	1.54E-02	217	2.92E-01	696	5.62E-01	689	6.68E-01	976	3.76E-01	494	1.48E-09	54	5.51E-01	919	2.59E-02	186	4.13E-02	657	4	4.81E-01	801	4.97E-01	1009	7.46E-01	872	1.82E-02	838	6.29E-02	800	1.68E-05	27	4.04E-01	774	2	6	625.56
8480	NM_003610	RAE1	9.80E-02	528	4.06E-02	423	2.70E-01	366	5.76E-01	936	2.57E-01	353	1.17E-02	576	4.46E-01	844	2.57E-01	580	3.81E-01	924	2	9.76E-01	1140	2.96E-01	879	4.61E-01	564	6.56E-05	489	1.52E-02	638	1.10E-03	97	2.77E-01	678	3	5	625.94
7037	NM_000660	TGFB1	7.09E-01	915	4.33E-03	92	4.81E-01	588	1.93E-01	684	9.46E-01	1096	1.96E-01	867	2.03E-01	587	6.35E-01	881	4.24E-04	311	2	4.00E-03	89	2.79E-02	485	3.80E-01	429	3.44E-03	732	9.16E-07	112	9.05E-01	1090	8.48E-01	1061	4	6	626.19
9805	NM_001145515	SCRN1	5.92E-02	483	1.03E-02	168	7.23E-01	874	8.55E-04	165	7.44E-01	898	2.16E-03	466	2.05E-01	593	7.75E-01	990	8.82E-01	1109	3	1.53E-01	451	7.51E-01	1091	7.55E-01	884	1.27E-05	367	1.58E-03	452	3.15E-01	697	5.56E-02	345	2	5	627.06
55914	NM_018839	nsfl1c	7.14E-01	916	8.34E-01	1050	4.99E-04	10	6.17E-01	951	3.43E-01	460	1.20E-02	580	1.93E-01	570	2.25E-01	553	2.47E-02	597	3	2.45E-01	564	6.85E-01	1070	2.04E-01	165	3.90E-01	1048	4.32E-03	522	5.75E-02	400	1.96E-01	595	1	4	628.19
54583	NM_022051	egln1	3.49E-04	33	1.41E-05	10	5.28E-02	163	2.56E-01	736	9.99E-01	1155	8.20E-01	1110	2.09E-02	171	1.58E-01	463	4.95E-06	152	4	5.33E-02	293	2.55E-01	823	9.23E-01	1059	2.04E-01	992	6.86E-01	1073	3.41E-01	723	9.03E-01	1101	0	4	628.56
3915	NM_006148	LASP1	2.92E-01	688	3.44E-02	378	7.51E-01	901	8.94E-01	1097	2.71E-01	373	7.09E-02	746	1.87E-01	564	9.90E-06	7	9.19E-01	1119	2	1.50E-01	446	7.75E-03	348	6.10E-01	736	4.95E-05	469	2.84E-02	709	1.80E-01	572	5.95E-01	905	3	5	628.63
7174	NM_003291	tpp2	8.81E-01	1044	1.99E-02	258	8.43E-01	1007	1.78E-02	375	3.95E-01	513	7.55E-01	1097	4.46E-02	255	8.17E-01	1017	5.73E-06	158	4	6.29E-01	896	2.59E-04	109	4.87E-01	590	3.49E-03	733	1.39E-01	871	6.63E-02	416	3.39E-01	723	2	6	628.88
6599	NM_139071	SMARCD1	1.03E-01	535	3.78E-01	753	1.49E-01	246	2.63E-01	744	4.87E-01	628	1.20E-02	581	2.49E-03	52	9.17E-01	1087	7.82E-01	1074	2	1.55E-01	458	2.59E-01	829	7.69E-01	902	4.52E-05	459	3.71E-03	509	2.51E-01	641	1.75E-01	573	2	4	629.44
64745	NM_022746	MOSC1	7.18E-01	918	1.02E-02	167	1.89E-01	292	3.38E-04	110	6.10E-01	758	9.48E-04	427	6.72E-01	987	8.16E-02	335	2.75E-01	865	3	8.56E-01	1059	7.43E-01	1090	2.10E-01	173	7.42E-03	775	2.99E-02	716	3.10E-01	690	3.20E-01	709	2	5	629.44
9209	NM_001134369	lrrfip2	9.64E-03	169	8.29E-01	1040	2.93E-01	393	5.88E-01	938	4.58E-02	94	5.19E-02	718	3.88E-01	798	3.03E-01	622	4.92E-04	319	3	3.84E-01	715	3.77E-01	949	8.86E-01	1017	9.14E-01	1150	2.21E-01	923	2.03E-03	118	1.08E-02	114	2	5	629.81
6895	NM_152295	Tars	9.97E-01	1153	4.64E-02	469	3.62E-01	465	2.16E-02	392	6.03E-01	753	2.12E-02	632	2.81E-01	693	3.86E-01	701	4.22E-02	660	4	1.03E-01	385	8.98E-02	630	6.62E-01	789	2.18E-04	572	1.49E-01	880	5.81E-01	893	4.95E-04	11	2	6	629.88
9650	NM_014637	MTFR1	8.62E-01	1029	9.14E-03	157	9.36E-01	1105	1.50E-01	651	6.41E-01	798	3.62E-08	96	2.34E-01	630	5.27E-01	798	3.05E-02	617	3	5.28E-02	290	6.21E-01	1056	1.42E-01	92	5.50E-03	754	2.66E-04	351	8.74E-01	1067	1.88E-01	588	2	5	629.94
7461	NM_032421	CLIP2	3.52E-02	374	7.96E-01	1022	7.37E-01	887	3.17E-04	105	7.66E-01	918	2.03E-03	463	5.43E-01	917	3.82E-01	697	5.83E-01	1009	3	3.27E-01	655	6.15E-05	57	7.87E-01	922	2.66E-07	129	1.13E-01	851	5.81E-01	892	2.19E-02	184	3	6	630.13
5479	NM_002706	PPM1B	3.35E-02	360	9.70E-01	1137	8.08E-01	965	3.92E-04	120	8.08E-01	968	4.83E-07	152	7.64E-03	96	6.03E-02	292	2.59E-05	200	5	9.90E-01	1149	1.86E-01	743	7.77E-01	910	1.52E-03	693	6.15E-01	1057	1.49E-02	231	7.97E-01	1024	2	7	631.06
9341	NM_004781	vamp3	3.30E-02	358	5.98E-01	879	8.77E-01	1046	6.00E-05	69	5.78E-01	722	4.06E-03	508	9.41E-02	409	9.90E-01	1151	6.55E-01	1034	3	3.07E-01	638	3.59E-01	932	7.14E-01	843	8.12E-05	506	2.38E-07	78	1.14E-01	495	9.23E-02	430	2	5	631.13
55623	NM_017768	LRRC40	7.83E-01	968	3.60E-02	390	1.80E-01	281	1.27E-01	622	4.26E-01	555	1.49E-01	833	7.11E-01	1003	6.60E-01	897	3.88E-03	452	2	1.28E-01	417	1.27E-01	683	9.46E-02	46	3.54E-02	893	1.69E-05	214	9.69E-01	1141	3.17E-01	706	2	4	631.31
57060	NM_020374	c12orf4	3.11E-01	707	3.77E-03	85	8.92E-01	1061	1.50E-01	652	2.32E-01	317	1.74E-03	455	7.96E-01	1054	1.38E-01	428	9.61E-01	1143	2	2.94E-01	620	3.21E-01	905	3.54E-02	24	5.70E-02	920	5.11E-03	542	3.58E-02	333	5.21E-01	855	3	5	631.31
2782	NM_004486	GOLGA2	3.56E-01	732	2.61E-02	309	8.22E-01	985	2.54E-03	215	4.57E-01	589	1.36E-05	233	2.21E-01	617	3.38E-01	652	7.25E-01	1052	3	9.20E-01	1099	2.47E-01	814	8.76E-01	1004	1.13E-05	359	1.70E-07	72	8.70E-01	1063	4.82E-02	307	3	6	631.38
3621	NM_002192	INHBA	2.89E-01	685	8.87E-01	1087	3.13E-01	422	7.28E-03	303	3.20E-01	434	3.88E-05	277	5.52E-01	921	8.62E-01	1044	1.83E-01	816	2	2.49E-01	571	7.93E-02	610	3.53E-01	380	2.67E-05	422	7.20E-01	1085	3.43E-03	137	6.06E-01	915	2	4	631.81
10175	NM_005776	CNIH	1.22E-02	192	5.32E-01	841	8.37E-01	1001	9.95E-01	1156	8.94E-01	1048	5.42E-07	159	7.69E-01	1034	5.42E-01	811	4.38E-11	13	3	1.64E-01	467	2.50E-03	248	8.84E-01	1015	2.94E-06	271	3.93E-02	745	1.50E-01	528	1.81E-01	580	3	6	631.81
6731	NM_182691	SRPK2	3.02E-02	339	4.49E-01	796	8.98E-01	1069	4.98E-02	505	9.38E-01	1086	3.47E-02	673	5.97E-01	946	3.46E-01	661	4.21E-04	310	4	8.01E-01	1013	3.63E-02	521	2.69E-01	245	1.23E-06	222	5.89E-04	385	4.36E-01	796	1.57E-01	543	3	7	631.88
81556	NM_001142292	LMAN2L	6.59E-01	890	4.52E-02	459	7.72E-01	922	2.23E-01	712	8.38E-01	991	7.19E-01	1090	8.95E-01	1097	5.45E-02	276	5.36E-03	478	2	2.44E-01	563	5.79E-01	1041	1.38E-01	88	6.31E-08	65	3.54E-02	732	1.85E-01	578	1.34E-02	131	3	5	632.06
768	NM_001216	CA9	9.77E-01	1129	9.09E-01	1100	3.87E-01	486	4.53E-03	255	5.53E-01	690	1.53E-13	15	4.01E-02	239	1.30E-03	41	4.18E-01	941	4	8.26E-01	1038	6.09E-01	1050	9.69E-01	1128	6.56E-08	68	2.43E-03	475	7.71E-01	1009	1.03E-01	452	2	6	632.25
1728	NM_001130823	dnmt1	2.93E-01	689	7.51E-02	525	3.77E-01	477	3.98E-08	2	1.79E-01	244	5.52E-02	723	7.86E-01	1046	3.82E-01	699	2.63E-01	858	1	7.72E-02	343	4.90E-01	1005	1.83E-01	143	3.07E-01	1023	1.09E-01	841	8.40E-01	1049	1.04E-01	454	0	1	632.56
11234	NM_181508	HPS5	2.48E-02	304	3.18E-01	715	6.51E-01	793	8.13E-02	560	9.47E-01	1097	5.16E-01	1026	1.81E-01	550	9.21E-01	1091	2.26E-02	589	2	6.31E-02	314	4.74E-04	144	1.74E-01	126	1.97E-05	395	4.37E-02	756	9.23E-01	1103	1.67E-01	559	3	5	632.63
4E+05	NM_001031827	BOLA2B	7.87E-01	972	7.69E-03	140	8.82E-01	1053	4.42E-03	252	9.55E-01	1106	1.50E-04	336	7.77E-02	373	1.72E-01	485	2.01E-02	577	4	8.06E-01	1017	1.82E-02	429	5.56E-01	676	1.53E-04	545	6.87E-01	1074	3.78E-03	144	6.75E-01	950	3	7	633.06
79029	NM_024065	Pdcl3	6.78E-01	897	3.47E-02	381	6.60E-01	804	4.76E-01	870	2.61E-01	360	3.36E-01	945	7.32E-01	1012	5.36E-02	275	1.74E-05	188	2	4.81E-01	802	7.39E-01	1089	1.27E-01	73	7.45E-06	327	6.64E-03	561	8.38E-01	1048	1.31E-01	500	2	4	633.25
4924	NM_005013	nucb2	8.19E-02	513	4.47E-02	456	5.93E-01	725	2.32E-01	719	7.03E-01	872	5.47E-01	1034	9.46E-01	1128	1.30E-01	414	1.73E-02	571	2	8.29E-01	1043	6.59E-03	334	1.37E-01	87	6.23E-06	315	3.57E-02	736	6.80E-01	959	2.96E-02	228	4	6	633.38
1665	NM_004403	Dfna5	7.55E-02	503	4.12E-02	431	6.92E-01	848	3.06E-02	430	7.06E-01	875	8.36E-02	770	9.80E-02	415	5.50E-01	822	2.84E-07	91	3	8.61E-02	361	2.18E-02	451	9.88E-01	1147	8.57E-04	659	9.86E-01	1154	3.65E-02	335	5.09E-01	843	3	6	633.44
57706	NM_001136557	GPR107	3.40E-01	725	1.79E-02	240	3.16E-01	425	9.03E-01	1105	4.51E-01	583	2.36E-01	891	2.25E-01	620	1.32E-01	422	6.65E-03	493	2	3.55E-01	688	1.34E-04	85	3.25E-01	329	3.87E-03	738	4.39E-03	525	9.45E-01	1121	9.89E-01	1152	3	5	633.88
4678	NM_002486	NCBP1	5.08E-01	821	8.32E-01	1045	4.05E-01	503	9.02E-01	1102	3.87E-02	75	4.27E-02	699	3.77E-01	789	4.40E-01	739	9.18E-01	1117	2	6.80E-04	41	2.11E-01	775	2.35E-01	196	4.33E-02	906	4.73E-02	771	5.94E-05	40	1.52E-01	529	4	6	634.25
1155	NM_000082	ERCC8	4.00E-01	762	3.37E-01	733	5.90E-01	721	1.20E-01	611	1.70E-02	30	5.16E-02	716	1.63E-01	523	9.68E-01	1129	7.74E-01	1069	1	5.88E-02	303	1.83E-01	738	7.35E-01	862	1.66E-04	550	8.10E-07	106	4.80E-02	373	6.17E-01	923	3	4	634.31
55009	NM_017943	FBXO34	8.13E-02	512	2.87E-02	332	7.32E-01	884	6.49E-02	537	4.37E-01	569	6.09E-05	296	4.04E-01	813	3.70E-01	684	2.47E-01	850	2	3.94E-01	729	3.20E-01	902	1.08E-01	57	3.53E-02	891	1.45E-02	632	7.16E-01	978	1.21E-01	486	2	4	634.50
3E+05	NM_001135919	SLC46A3	9.27E-01	1076	7.78E-03	141	1.99E-01	302	1.72E-01	675	2.62E-01	362	1.51E-02	606	4.61E-01	857	2.82E-01	602	2.61E-01	856	2	8.73E-01	1071	3.88E-01	956	3.41E-01	358	6.18E-03	761	1.18E-04	316	1.02E-01	479	3.52E-01	734	2	4	634.50
11067	NM_007021	C10orf10	6.35E-01	870	2.22E-07	3	9.77E-01	1144	9.95E-01	1157	1.61E-01	226	2.16E-03	467	1.15E-01	446	4.77E-03	78	2.47E-01	849	3	3.28E-01	657	3.35E-01	915	1.17E-01	63	5.48E-01	1080	6.23E-04	391	9.75E-01	1146	2.64E-01	662	1	4	634.63
836	NM_004346	Casp3	1.27E-01	560	2.46E-02	290	9.00E-01	1071	3.67E-01	813	2.12E-01	282	3.06E-04	369	6.51E-02	333	1.40E-01	434	1.34E-03	384	3	5.21E-01	827	5.71E-01	1036	9.31E-01	1072	1.91E-03	707	1.29E-01	868	9.06E-01	1091	2.08E-03	28	2	5	635.31
55149	NM_018116	msto1	5.85E-01	852	1.32E-02	197	1.69E-01	272	5.38E-02	520	3.83E-01	503	3.84E-01	974	1.36E-01	479	6.03E-01	861	1.84E-02	574	2	1.93E-01	510	6.97E-01	1072	5.14E-01	631	1.30E-04	533	7.02E-01	1079	3.46E-02	325	4.30E-01	792	2	4	635.88
675	NM_000059	Brca2	7.43E-01	940	2.06E-01	637	1.73E-01	279	9.69E-01	1145	8.57E-01	1013	2.19E-03	469	9.40E-02	408	4.08E-01	722	9.62E-01	1144	1	1.68E-02	164	1.47E-01	707	3.89E-01	449	2.97E-04	593	1.22E-03	436	1.54E-01	533	1.55E-01	536	3	4	635.94
10296	NM_001017405	maeA	2.19E-02	282	5.08E-01	833	5.04E-01	617	2.54E-01	735	9.21E-01	1067	2.41E-04	357	2.21E-01	618	9.94E-01	1152	1.09E-01	753	2	2.27E-01	536	7.83E-02	604	3.87E-01	443	1.41E-07	107	2.19E-01	921	5.36E-01	861	4.36E-01	290	2	4	636.00
29093	NM_014180	MRPL22	7.24E-01	920	9.78E-03	164	6.83E-01	838	8.05E-03	312	7.71E-02	138	1.69E-01	849	6.53E-02	335	9.86E-01	1146	3.18E-02	623	3	9.91E-01	1150	4.48E-01	989	1.92E-01	149	1.53E-03	694	3.86E-01	976	5.88E-01	899	7.71E-04	15	2	5	637.31
4485	NM_002451	mtaP	4.01E-01	763	1.60E-02	222	9.44E-01	1114	3.56E-01	807	4.33E-02	90	1.27E-02	586	6.38E-01	968	2.22E-01	550	2.87E-02	611	4	1.06E-01	390	8.14E-01	1112	4.14E-01	483	2.39E-02	858	2.17E-01	920	6.37E-02	412	4.99E-02	318	2	6	637.75
3309	NM_153201	HSPA8	1.36E-03	58	8.33E-01	1046	6.60E-01	805	9.19E-04	170	1.65E-01	230	5.99E-01	1050	3.84E-01	794	3.47E-01	665	6.00E-08	72	3	3.76E-01	708	6.38E-02	577	7.44E-01	870	2.61E-02	862	4.91E-01	1022	8.76E-01	1069	2.63E-02	207	2	5	637.81
81562	NM_001128309	TSPAN14	9.30E-01	1079	4.26E-02	440	1.24E-01	226	4.68E-01	865	7.28E-01	887	3.65E-01	964	4.58E-01	854	7.32E-02	319	4.19E-04	309	2	1.13E-01	403	3.59E-02	518	5.86E-01	711	2.51E-01	1004	2.03E-02	668	1.64E-01	545	8.75E-02	417	2	4	638.06
56898	NM_020143	pno1	9.87E-01	1141	2.23E-02	274	2.96E-01	399	3.92E-01	826	8.92E-01	1046	3.22E-02	667	1.61E-01	520	1.60E-03	44	1.50E-01	786	3	9.99E-01	1156	5.19E-01	1018	3.10E-01	308	2.22E-04	573	1.69E-02	649	3.56E-01	732	6.70E-03	73	3	6	638.25
22862	NM_014923	FNDC3A	7.57E-01	948	2.52E-01	672	5.08E-04	11	2.25E-02	398	6.15E-01	767	2.44E-01	896	6.13E-01	955	7.26E-01	957	2.90E-06	137	3	6.93E-01	933	4.58E-02	550	9.57E-01	1112	4.83E-03	747	5.06E-06	172	2.50E-01	639	5.16E-02	327	3	6	638.81
9521	NM_004280	EEF1E1	3.44E-01	727	6.26E-03	118	2.25E-01	333	9.65E-01	1144	9.33E-01	1083	2.77E-01	918	6.65E-01	980	9.45E-02	358	8.80E-04	353	2	3.56E-01	691	5.85E-01	1046	4.56E-01	553	7.70E-09	23	1.51E-02	637	4.93E-01	834	9.35E-02	432	2	4	639.38
5531	NM_002726	prep	2.86E-02	330	9.86E-01	1147	6.13E-01	755	2.19E-02	395	5.96E-01	744	6.94E-04	409	8.53E-01	1081	1.18E-01	398	8.20E-01	1087	3	5.08E-01	816	1.43E-02	405	8.00E-01	939	6.17E-04	646	1.10E-01	842	2.35E-03	123	1.21E-02	120	4	7	639.81
48	NM_002197	ACO1	4.03E-02	407	1.65E-01	602	9.16E-01	1083	8.06E-01	1055	6.21E-01	777	2.96E-09	61	3.64E-01	773	1.83E-01	502	7.14E-02	708	2	5.41E-01	843	4.40E-02	544	4.06E-01	469	2.69E-06	263	1.83E-01	900	8.38E-01	1047	2.62E-02	205	3	5	639.94
1E+05	NM_017647	Ftsj3	6.18E-02	486	9.30E-01	1110	2.44E-01	347	2.78E-01	755	7.65E-01	917	1.15E-11	27	1.01E-01	424	3.51E-01	670	5.79E-06	160	2	9.07E-01	1088	2.71E-04	110	9.15E-01	1044	3.15E-06	277	5.22E-02	778	9.10E-01	1094	6.83E-01	957	2	4	640.25
26747	NM_012345	NUFIP1	2.86E-01	684	3.11E-04	23	6.64E-01	807	6.05E-02	534	9.51E-01	1102	3.86E-03	507	3.84E-01	795	1.52E-01	455	4.15E-03	460	3	5.53E-01	852	2.96E-01	880	1.34E-01	80	2.12E-04	570	8.62E-01	1119	1.07E-01	485	5.80E-01	894	1	4	640.44
1E+05	NM_138459	NUS1	2.43E-01	641	3.41E-03	82	8.76E-01	1043	3.94E-03	245	8.88E-01	1039	1.03E-06	170	9.57E-01	1135	1.23E-02	124	9.02E-01	1116	4	9.67E-01	1133	1.72E-01	726	1.36E-01	84	2.34E-04	579	1.44E-02	630	6.21E-01	917	1.84E-01	584	2	6	640.50
11188	NM_007184	nisch	2.52E-02	308	6.19E-01	896	6.04E-01	745	3.23E-01	781	7.47E-01	902	2.29E-05	259	2.40E-01	640	8.57E-01	1039	1.64E-01	798	2	2.39E-02	201	4.49E-03	306	9.07E-01	1037	1.22E-06	221	1.88E-02	659	5.02E-01	838	2.14E-01	619	4	6	640.56
51691	NM_016200	naa38	2.09E-02	265	3.13E-01	711	4.17E-01	515	4.19E-01	840	3.28E-02	67	6.66E-02	737	1.27E-01	466	5.34E-01	807	3.38E-10	22	3	8.58E-01	1062	7.11E-01	1081	7.72E-01	904	1.18E-04	528	4.34E-03	523	3.60E-01	736	7.51E-01	999	2	5	641.44
26608	NM_012453	tbl2	3.04E-02	341	3.65E-01	744	5.51E-01	677	4.48E-03	253	9.97E-01	1153	8.02E-04	417	3.72E-01	784	5.91E-01	853	1.95E-06	125	4	9.01E-02	365	8.21E-01	1114	9.60E-01	1115	1.18E-04	529	3.91E-03	514	7.34E-01	987	4.68E-02	297	3	7	641.75
1616	NM_004083	DDIT3	6.67E-01	893	4.77E-02	480	6.89E-01	842	1.77E-01	680	7.36E-01	891	3.03E-05	270	9.68E-01	1144	2.10E-02	164	5.01E-01	981	3	4.75E-02	278	2.55E-02	474	5.35E-01	650	2.12E-02	851	7.00E-03	566	4.93E-02	378	3.50E-01	732	5	8	642.13
10487	NM_001105530	CAP1	1.73E-01	590	6.55E-01	919	7.87E-01	938	2.08E-02	387	8.82E-01	1031	6.75E-03	535	5.75E-01	934	2.48E-01	576	5.71E-01	1006	2	4.40E-01	766	2.27E-04	106	7.97E-01	936	7.04E-02	936	5.32E-03	545	1.34E-05	23	4.04E-03	51	4	6	642.44
8301	NM_001008660	PICALM	3.22E-01	716	3.46E-02	380	7.46E-01	895	8.59E-01	1080	7.46E-01	900	8.04E-03	544	7.59E-01	1030	6.33E-02	300	2.61E-03	423	3	3.94E-01	728	1.75E-01	728	2.31E-02	12	1.06E-03	673	3.47E-03	502	6.19E-01	912	1.09E-01	462	3	6	642.81

**79001**	**NM_024040**	**CUEDC2**	9.78E-03	171	2.37E-02	282	9.04E-01	1076	3.46E-01	800	8.50E-01	1005	9.79E-01	1150	3.37E-01	740	4.00E-02	239	3.12E-01	891	**3**	4.00E-01	732	2.82E-04	112	6.20E-01	747	1.30E-08	29	2.67E-02	699	6.37E-01	931	2.88E-01	687	**3**	**6**	**643.19**
**10713**	**NM_006590**	**Usp39**	1.25E-01	557	8.78E-01	1080	6.01E-01	742	3.48E-01	801	6.97E-01	862	1.07E-02	570	2.63E-01	671	3.00E-01	618	2.71E-01	862	**1**	5.23E-01	828	1.32E-01	689	4.12E-01	480	6.60E-05	490	2.56E-05	225	1.65E-01	548	4.24E-02	283	**3**	**4**	**644.13**
**4E+05**	**NM_001165030**	**TMEM41B**	2.70E-01	662	5.21E-02	502	3.35E-01	441	2.39E-02	403	8.67E-01	1020	3.53E-01	954	2.36E-01	635	8.26E-01	1023	9.62E-02	740	**1**	1.95E-02	183	1.60E-01	714	7.60E-01	891	6.29E-01	1100	2.24E-06	144	2.73E-01	664	3.06E-02	235	**3**	**4**	**644.44**
**64795**	**NM_023937**	**Mrpl34**	4.79E-01	803	6.69E-01	926	5.94E-01	727	5.33E-01	906	7.76E-01	929	4.28E-01	996	2.02E-01	585	3.68E-02	226	2.86E-01	874	**1**	1.38E-01	437	9.65E-03	372	8.92E-01	1022	3.31E-05	441	5.59E-08	56	7.45E-01	997	1.25E-03	20	**4**	**5**	**644.81**
**6129**	**NM_003161**	**RPS6KB1**	1.83E-02	240	2.64E-01	683	6.89E-01	843	2.92E-01	761	2.07E-01	278	1.19E-03	435	8.25E-01	1074	3.13E-02	206	4.10E-02	655	**4**	9.60E-01	1129	3.67E-01	941	4.63E-01	568	1.39E-03	689	4.63E-01	1013	9.09E-04	93	3.31E-01	715	**2**	**6**	**645.19**
**11236**	**NM_007218**	**RNF139**	1.27E-02	199	5.20E-01	839	6.03E-01	743	2.91E-02	424	6.81E-01	843	1.01E-08	76	6.33E-01	965	6.01E-02	291	1.33E-03	383	**4**	7.19E-01	948	3.59E-03	281	8.08E-01	945	1.10E-02	805	1.25E-03	437	9.74E-01	1145	7.59E-01	1003	**3**	**7**	**645.44**
**54700**	**NM_017615**	**NSMCE4A**	1.76E-02	232	9.69E-01	1136	8.12E-01	969	1.80E-02	376	5.60E-01	698	1.74E-02	618	7.91E-02	378	5.13E-01	789	3.31E-02	628	**4**	8.67E-01	1069	7.94E-01	1102	3.77E-01	421	1.13E-05	357	4.05E-05	255	8.59E-03	188	9.33E-01	1118	**3**	**7**	**645.88**
**2908**	**NM_004491**	**GRLF1**	2.64E-01	660	4.52E-01	799	7.22E-01	873	5.20E-01	898	6.57E-01	815	2.45E-01	897	5.66E-01	929	1.54E-02	138	1.26E-02	551	**2**	2.93E-01	619	3.05E-04	115	2.84E-01	265	4.32E-01	1054	8.39E-02	817	5.42E-01	864	2.84E-03	41	**2**	**4**	**645.94**
**9533**	**NM_203290**	**Polr1c**	6.08E-01	863	3.93E-03	88	8.18E-02	194	4.55E-02	496	7.01E-01	869	7.07E-01	1086	6.25E-02	329	7.18E-01	946	8.08E-02	723	**2**	1.80E-01	489	9.99E-01	1158	6.35E-01	764	1.77E-06	238	8.71E-01	1121	3.43E-02	323	2.55E-01	652	**2**	**4**	**646.19**
**1942**	**NM_001080497**	**MEGF9**	9.47E-01	1097	4.53E-03	93	7.05E-01	855	7.62E-03	310	6.72E-01	834	8.70E-01	1125	2.93E-01	703	6.09E-04	32	2.99E-05	203	**4**	9.21E-01	1101	9.43E-01	1147	3.53E-01	381	7.56E-07	187	1.69E-01	895	9.50E-01	1127	3.63E-02	257	**2**	**6**	**646.69**
**57405**	**NM_020946**	**dennd1a**	9.35E-01	1087	4.69E-02	473	5.71E-01	701	5.80E-02	528	3.84E-01	504	1.59E-01	838	4.37E-02	251	6.06E-01	862	4.07E-05	218	**3**	5.34E-01	837	4.62E-01	994	3.57E-01	389	7.77E-03	779	1.47E-04	323	1.72E-01	560	7.66E-01	1007	**2**	**5**	**646.94**
**23588**	**NM_014315**	**Klhdc2**	4.20E-02	423	2.54E-01	674	6.55E-01	799	6.15E-03	282	8.54E-01	1011	1.97E-04	347	4.52E-02	257	4.88E-01	775	5.23E-01	987	**4**	1.68E-02	163	2.63E-01	834	7.55E-01	885	2.71E-01	1012	2.13E-03	462	4.40E-01	801	2.39E-01	640	**2**	**6**	**647.00**
**8242**	**NM_004187**	**KDM5C**	3.15E-01	708	1.21E-01	564	3.11E-02	114	3.84E-01	820	4.17E-01	542	5.88E-02	729	9.11E-01	1110	1.79E-01	495	3.57E-05	211	**2**	4.12E-01	740	9.46E-01	1149	2.52E-01	214	2.27E-02	856	1.11E-02	608	5.25E-01	851	2.47E-01	646	**2**	**4**	**647.31**
**6873**	**NM_003186**	**TAGLN**	9.97E-01	1154	4.79E-02	482	5.87E-01	718	1.01E-01	590	9.77E-01	1131	9.51E-03	558	3.16E-01	722	4.16E-01	727	1.15E-03	376	**3**	2.55E-01	573	2.73E-01	847	1.01E-01	51	3.22E-05	440	1.02E-03	426	5.78E-01	889	2.74E-01	676	**2**	**5**	**647.50**
**9205**	**NM_001142684**	**ZMYM5**	4.48E-01	784	5.64E-01	859	2.36E-01	339	8.86E-01	1093	2.20E-01	297	8.20E-02	764	4.02E-02	240	9.62E-01	1123	5.30E-01	991	**1**	6.41E-01	901	1.85E-01	739	1.78E-01	132	4.74E-04	627	2.00E-02	666	1.70E-01	556	3.38E-02	249	**3**	**4**	**647.50**
**7529**	**NM_139323**	**YWHAB**	1.15E-02	184	8.97E-01	1091	7.55E-01	904	7.81E-01	1035	2.26E-01	306	6.88E-03	536	9.06E-01	1106	6.49E-02	304	4.54E-01	963	**2**	6.67E-01	914	2.11E-03	235	4.52E-01	550	1.42E-06	230	9.48E-03	593	3.40E-02	322	8.92E-01	1094	**4**	**6**	**647.94**
**26284**	**NM_005702**	**ERAL1**	2.80E-01	671	5.34E-03	107	6.21E-02	176	4.91E-03	264	6.19E-01	774	7.94E-02	755	5.11E-01	889	4.53E-01	751	8.15E-01	1085	**2**	2.89E-02	220	9.34E-01	1145	3.02E-01	297	1.10E-03	676	9.44E-03	588	8.02E-01	1033	6.55E-01	938	**3**	**5**	**648.06**
**55388**	**NM_018555**	**ZNF331**	9.19E-01	1067	1.82E-02	244	3.02E-01	408	3.54E-01	805	7.70E-01	924	2.84E-09	60	4.56E-01	852	6.85E-01	921	7.32E-03	499	**3**	1.61E-02	159	3.02E-02	500	9.86E-01	1141	7.48E-02	941	5.69E-03	552	5.95E-01	902	7.90E-02	395	**3**	**6**	**648.13**
**26112**	**NM_015621**	**ccdc69**	2.49E-02	306	6.01E-01	883	4.19E-01	518	7.01E-01	996	4.49E-01	581	2.01E-01	868	3.37E-01	738	9.52E-01	1116	4.21E-02	658	**2**	8.65E-01	1068	3.84E-03	292	1.28E-01	74	2.80E-01	1014	6.79E-06	183	2.04E-01	597	1.18E-01	479	**2**	**4**	**648.19**
**4E+05**	**NM_203318**	**Myo18a**	1.73E-01	589	4.09E-02	425	7.20E-02	183	5.44E-01	911	2.06E-01	276	3.08E-07	141	9.22E-04	23	7.90E-02	328	1.73E-01	807	**3**	6.23E-01	893	1.77E-01	730	9.03E-01	1030	3.30E-02	886	8.94E-01	1127	6.13E-01	910	9.28E-01	1112	**1**	**4**	**648.19**
**705**	**NM_004053**	**bysl**	9.42E-01	1091	1.52E-02	214	1.66E-01	267	5.10E-02	512	7.69E-01	923	2.76E-01	914	9.67E-01	1142	8.06E-01	1010	4.37E-03	466	**2**	1.71E-01	475	5.79E-01	1043	4.46E-01	535	2.00E-09	10	9.92E-01	1156	9.03E-03	191	9.33E-02	431	**2**	**4**	**648.75**
**10920**	**NM_198189**	**Cops8**	8.07E-01	991	2.76E-02	323	7.40E-01	889	9.80E-04	171	4.13E-01	535	8.08E-07	165	3.97E-02	235	4.09E-01	724	3.74E-05	215	**5**	5.34E-01	838	4.45E-01	988	3.53E-01	382	2.57E-02	860	5.77E-01	1048	8.99E-01	1084	6.53E-01	935	**1**	**6**	**648.94**
**223**	**NM_000696**	**ALDH9A1**	2.04E-02	256	5.11E-01	834	3.35E-01	439	6.31E-01	959	9.69E-01	1116	1.94E-07	131	2.82E-02	194	2.91E-01	610	8.22E-01	1089	**3**	5.63E-01	859	6.83E-05	61	7.98E-01	938	7.71E-01	1132	1.06E-02	606	2.41E-02	288	5.58E-01	878	**3**	**6**	**649.38**
**699**	**NM_004336**	**BUB1**	9.85E-01	1138	3.79E-02	405	5.69E-01	695	3.56E-02	455	6.13E-01	763	1.64E-01	846	2.94E-01	704	4.21E-01	732	1.71E-02	570	**3**	5.24E-02	289	4.74E-01	998	2.62E-01	234	9.44E-03	794	4.23E-04	366	7.34E-01	988	8.57E-02	414	**2**	**5**	**649.44**
**1385**	**NM_001310**	**CREBL2**	1.25E-02	195	8.15E-01	1032	9.57E-01	1125	9.20E-01	1116	5.33E-01	664	9.91E-01	1155	3.15E-02	210	4.88E-01	776	1.27E-05	177	**3**	2.49E-01	570	2.11E-02	448	6.90E-01	820	3.86E-02	899	3.10E-06	155	5.51E-02	392	2.72E-01	673	**3**	**6**	**650.44**
**6809**	**NM_005638**	**VAMP7**	4.36E-02	435	6.06E-01	885	1.65E-01	266	5.97E-01	943	4.77E-01	612	7.87E-03	542	2.35E-01	633	6.15E-02	296	6.34E-02	699	**2**	1.92E-01	508	5.58E-01	1031	7.90E-01	925	3.50E-04	604	1.30E-02	622	9.44E-01	1120	4.27E-02	287	**3**	**5**	**650.50**
**1351**	**NM_000097**	**CPOX**	4.96E-02	468	8.57E-02	532	7.24E-01	875	7.31E-04	151	4.35E-01	567	1.61E-01	841	4.02E-01	811	3.90E-01	703	5.06E-01	982	**2**	3.00E-02	225	2.70E-01	842	3.95E-01	458	9.09E-03	791	2.94E-03	493	8.35E-01	1045	2.29E-01	635	**3**	**5**	**651.19**
**10399**	**NM_006098**	**GNB2L1**	2.43E-02	301	8.73E-01	1076	8.50E-01	1012	4.59E-04	130	5.49E-01	685	3.45E-02	672	8.24E-02	385	9.02E-01	1074	5.80E-01	1008	**3**	7.40E-01	968	8.65E-01	1127	4.44E-01	532	1.96E-04	561	2.57E-03	480	7.49E-05	44	7.06E-02	382	**3**	**6**	**652.31**
**23300**	**NM_015251**	**ATMIN**	7.01E-01	909	7.41E-01	984	8.04E-01	962	2.92E-06	16	3.69E-01	489	9.04E-01	1135	1.77E-01	545	3.98E-01	714	4.57E-03	470	**2**	1.82E-01	493	1.30E-02	395	6.74E-01	803	2.98E-06	275	1.18E-02	615	7.16E-01	979	2.65E-01	664	**3**	**5**	**653.00**
**5634**	**NM_001042465**	**PSAP**	3.60E-01	735	9.09E-01	1101	3.90E-02	133	4.94E-01	878	1.27E-01	194	9.07E-01	1136	8.97E-01	1100	9.12E-03	108	2.38E-03	417	**3**	5.45E-01	847	1.09E-03	191	5.55E-01	674	9.03E-02	950	3.50E-05	244	7.32E-01	985	3.80E-01	756	**2**	**5**	**653.06**
**57720**	**XM_001719592**	**ANKRD36B**	2.81E-02	326	2.92E-01	697	6.31E-01	769	2.78E-01	756	4.02E-01	521	1.45E-03	446	5.69E-02	302	5.49E-01	821	1.05E-02	533	**3**	3.41E-01	672	6.09E-01	1051	7.96E-01	933	1.22E-02	812	1.03E-04	307	9.29E-01	1107	8.27E-02	410	**2**	**5**	**653.94**
**4277**	**NM_002415**	**MIF**	5.55E-03	125	9.94E-01	1153	4.59E-01	561	1.73E-01	677	2.75E-01	380	3.56E-01	956	5.18E-01	896	2.64E-01	587	7.89E-01	1076	**1**	3.72E-01	706	2.23E-01	785	2.94E-01	282	5.33E-02	917	3.04E-05	237	6.68E-03	172	6.90E-01	961	**2**	**3**	**654.44**
**55622**	**NM_017736**	**THUMPD1**	2.98E-01	698	7.37E-02	524	2.06E-02	78	7.29E-01	1006	8.07E-02	143	3.19E-02	665	4.04E-01	814	9.19E-01	1088	2.03E-01	831	**2**	5.13E-01	821	8.28E-01	1115	7.93E-01	929	4.19E-07	153	9.11E-01	1132	8.98E-02	461	7.33E-04	14	**2**	**4**	**654.50**
**9197**	**NM_004733**	**slc33a1**	2.59E-01	654	9.72E-01	1139	6.66E-01	812	5.86E-01	937	5.02E-01	642	4.85E-08	106	7.65E-02	367	2.93E-01	613	1.46E-04	270	**2**	9.78E-02	376	2.10E-01	771	9.44E-01	1093	1.46E-01	972	7.06E-03	567	2.66E-02	299	5.23E-01	856	**2**	**4**	**654.63**
**55831**	**NM_015691**	**WWC3**	4.32E-01	777	7.08E-03	129	1.00E-01	204	8.00E-01	1052	1.10E-01	178	5.43E-01	1031	7.67E-02	369	1.13E-02	117	9.40E-01	1133	**2**	6.62E-01	911	4.59E-01	992	4.32E-01	515	3.15E-02	879	2.08E-02	672	5.62E-01	878	2.36E-01	637	**2**	**4**	**654.63**
**10228**	**NM_005819**	**STX6**	6.13E-03	134	3.54E-02	386	6.51E-01	791	6.62E-01	972	1.30E-02	25	1.93E-07	130	5.55E-01	923	3.07E-01	627	2.42E-02	595	**5**	3.60E-01	693	8.90E-01	1139	1.21E-01	68	5.06E-01	1075	1.56E-01	885	6.24E-01	921	9.28E-01	1113	**0**	**5**	**654.81**
**79650**	**NM_024612**	**DHX40**	2.82E-02	327	7.31E-01	974	3.49E-02	125	3.75E-02	466	3.42E-01	459	7.83E-05	312	6.13E-01	954	9.47E-01	1112	1.51E-03	392	**5**	2.40E-01	556	3.38E-01	918	4.49E-01	542	2.16E-01	994	1.59E-01	886	1.14E-01	496	7.04E-01	975	**0**	**5**	**655.50**
**79903**	**NM_024945**	**Rmi1**	3.41E-02	364	8.39E-01	1053	2.04E-01	308	5.91E-01	942	9.60E-01	1109	2.60E-02	647	5.93E-01	943	6.65E-01	902	1.38E-04	267	**3**	5.14E-01	823	2.72E-04	111	2.08E-01	171	6.73E-02	930	1.07E-03	430	9.44E-01	1119	6.53E-02	370	**2**	**5**	**655.56**
**5877**	**NM_201434**	**rab5c**	3.88E-01	752	2.45E-02	289	9.59E-01	1126	1.22E-01	615	3.97E-02	79	4.28E-02	700	2.56E-03	54	3.38E-01	650	5.69E-01	1003	**4**	3.59E-01	692	5.07E-01	1014	9.67E-01	1125	3.53E-04	605	9.72E-04	423	1.32E-01	516	5.25E-01	857	**2**	**6**	**656.25**
**10404**	**NM_016134**	**Pgcp**	3.64E-02	383	2.03E-01	633	7.00E-01	853	9.34E-01	1125	6.11E-01	762	5.07E-01	1021	7.33E-01	1014	6.40E-06	4	1.46E-02	564	**3**	7.92E-01	1011	2.87E-01	864	4.96E-01	604	1.27E-02	813	1.08E-04	311	1.91E-02	263	4.08E-02	275	**4**	**7**	**656.25**
**9839**	**NM_014795**	**Zeb2**	7.32E-01	927	9.36E-01	1115	6.74E-05	4	6.92E-01	991	7.27E-01	886	2.30E-02	639	1.10E-01	439	2.00E-02	159	1.21E-02	545	**4**	9.47E-01	1122	1.30E-02	394	5.08E-01	626	9.72E-02	954	6.99E-04	404	9.79E-01	1152	1.55E-02	146	**3**	**7**	**656.44**
**79847**	**NM_024808**	**c13orf34**	3.04E-02	342	5.64E-01	858	7.09E-01	858	1.04E-03	175	2.14E-01	287	4.27E-01	995	6.26E-01	960	6.25E-01	877	2.36E-01	845	**2**	4.56E-02	276	1.99E-01	757	3.67E-01	407	9.63E-01	1158	1.70E-02	650	1.29E-01	513	1.61E-01	549	**2**	**4**	**656.69**
**80176**	**NM_025149**	**ACSF2**	8.06E-01	990	4.85E-01	816	8.60E-03	54	3.99E-01	830	1.88E-01	255	3.14E-03	495	3.38E-01	741	7.21E-01	951	8.77E-01	1107	**2**	3.46E-01	675	2.09E-01	769	7.19E-01	848	2.90E-02	870	1.81E-02	657	5.68E-02	398	4.93E-03	58	**3**	**5**	**657.13**
**51313**	**NM_001031700**	**fam198b**	3.95E-01	759	4.91E-02	490	8.37E-01	1000	4.26E-01	842	6.50E-01	808	2.02E-04	348	9.02E-02	400	1.99E-02	157	3.09E-03	437	**4**	6.94E-01	934	2.40E-01	809	9.44E-01	1094	7.18E-05	497	3.51E-05	245	4.62E-01	818	5.63E-01	882	**2**	**6**	**657.50**
**79095**	**NM_024121.2**	**Tmem185b**	3.55E-02	375	6.79E-01	934	8.31E-01	992	1.67E-01	672	2.38E-02	43	8.77E-01	1128	7.52E-01	1025	4.82E-01	770	2.19E-07	88	**3**	3.18E-01	648	2.73E-01	845	8.76E-01	1006	3.80E-04	610	1.27E-03	439	3.40E-01	721	2.89E-02	225	**3**	**6**	**657.56**
**3992**	**NM_001142316**	**LMO2**	2.93E-04	32	6.13E-01	890	7.20E-01	871	4.02E-01	831	7.84E-01	942	4.99E-02	712	3.44E-01	747	3.01E-02	203	4.21E-02	659	**4**	5.32E-02	292	7.78E-01	1095	3.28E-01	336	7.50E-03	776	2.35E-01	929	5.27E-01	853	5.88E-02	357	**1**	**5**	**657.81**
**63916**	**NM_022151**	**Moap1**	8.74E-03	162	6.95E-01	952	8.87E-01	1056	9.09E-01	1108	4.31E-01	561	8.97E-01	1132	8.31E-01	1076	3.09E-02	204	6.86E-03	494	**3**	8.09E-01	1020	9.02E-04	178	8.38E-01	968	4.43E-04	620	6.68E-03	562	1.51E-02	234	2.61E-02	204	**5**	**8**	**658.19**
**51540**	**NM_016510**	**SCLY**	2.92E-01	687	1.22E-02	189	6.91E-01	847	6.31E-01	960	8.75E-01	1025	9.88E-03	560	2.66E-01	676	7.84E-01	993	1.24E-05	175	**3**	7.55E-01	987	1.70E-03	225	7.84E-01	918	5.14E-07	158	1.29E-01	867	8.81E-01	1075	2.35E-02	194	**3**	**6**	**658.50**
**4152**	**NM_207294**	**Mbnl1**	5.97E-01	858	3.25E-02	366	1.70E-01	275	5.70E-02	526	7.66E-01	919	4.55E-01	1008	6.02E-02	316	2.19E-01	546	4.79E-01	970	**1**	6.88E-01	929	1.38E-01	701	8.26E-01	960	4.47E-05	458	7.65E-02	812	2.47E-01	636	3.70E-02	263	**2**	**3**	**658.94**
**6400**	**NM_006924**	**SFRS1**	2.00E-01	608	1.36E-01	575	9.40E-01	1111	9.54E-01	1133	7.99E-01	958	3.65E-03	505	3.66E-02	225	6.54E-01	891	6.22E-02	696	**2**	2.07E-01	518	1.37E-01	697	2.82E-01	262	3.64E-01	1040	7.35E-03	572	5.09E-02	384	6.60E-02	372	**1**	**3**	**659.19**
**581**	**NM_004324**	**BAX**	9.53E-02	527	9.60E-01	1131	3.94E-01	493	3.12E-02	434	6.59E-01	819	2.26E-01	884	1.24E-01	459	6.37E-01	882	3.98E-01	932	**1**	2.59E-01	577	3.36E-02	511	5.82E-01	707	1.31E-02	816	1.10E-04	313	1.99E-02	266	4.43E-01	799	**4**	**5**	**659.38**
**9180**	**NM_003999**	**OSMR**	6.77E-01	896	4.25E-02	439	6.33E-01	771	6.87E-01	989	4.29E-02	86	6.30E-01	1057	2.34E-01	629	2.04E-01	528	3.61E-03	447	**3**	9.13E-01	1094	2.82E-01	856	2.35E-03	2	6.26E-01	1098	6.61E-04	398	1.16E-01	499	3.89E-01	764	**2**	**5**	**659.56**
**30846**	**NM_014601**	**Ehd2**	8.00E-01	982	3.05E-02	351	9.61E-01	1127	9.15E-01	1111	1.51E-02	28	4.51E-02	704	9.72E-01	1146	3.03E-01	623	1.05E-03	367	**4**	2.78E-01	601	4.04E-01	970	6.64E-02	33	1.62E-02	830	3.75E-05	252	5.60E-01	877	1.66E-01	556	**2**	**6**	**659.88**
**2739**	**NM_001118890**	**GLRX**	1.50E-02	214	4.11E-01	770	6.09E-01	751	1.29E-02	350	9.25E-01	1073	5.94E-01	1048	1.51E-01	502	8.31E-01	1025	3.24E-03	438	**3**	6.62E-01	910	2.51E-02	471	6.29E-01	754	1.73E-01	979	1.15E-05	203	5.10E-01	844	2.97E-02	229	**3**	**6**	**660.06**
**29889**	**NM_013285**	**GNL2**	3.71E-02	388	8.50E-01	1062	8.51E-01	1014	4.47E-02	491	8.03E-02	142	3.17E-04	371	9.11E-01	1111	1.90E-01	515	2.11E-01	834	**3**	2.76E-01	597	2.61E-01	830	8.73E-01	1002	4.57E-04	625	2.09E-03	460	5.70E-03	166	6.91E-01	964	**3**	**6**	**660.75**
**3476**	**NM_203283**	**Rbpj**	6.11E-04	37	2.54E-01	676	7.61E-01	911	7.78E-01	1034	8.17E-01	975	3.73E-02	682	5.90E-02	311	9.24E-01	1095	2.90E-01	878	**2**	4.21E-01	749	3.09E-02	504	7.33E-01	860	5.50E-04	638	4.66E-03	529	4.76E-02	371	5.18E-02	328	**4**	**6**	**661.13**
**4332**	**NM_001166460**	**MPP1**	4.99E-02	473	8.08E-01	1029	2.49E-01	353	9.32E-01	1124	5.84E-01	728	8.15E-02	762	3.02E-02	204	9.68E-01	1128	1.12E-01	757	**2**	4.29E-01	754	2.97E-03	265	8.40E-01	971	1.08E-01	958	2.91E-05	234	3.46E-08	2	4.95E-01	836	**3**	**5**	**661.13**
**811**	**NM_004343**	**CALR**	4.05E-01	765	8.51E-01	1063	4.14E-01	511	3.55E-01	806	1.23E-01	188	1.53E-03	448	8.59E-01	1084	8.63E-01	1046	3.07E-02	618	**2**	2.36E-01	549	1.57E-02	412	8.51E-01	980	1.26E-05	366	1.68E-02	647	3.82E-02	340	3.96E-01	769	**4**	**6**	**662.00**
**55279**	**NM_018294**	**CWF19L1**	3.59E-02	380	5.06E-01	831	7.15E-01	866	7.67E-01	1029	2.86E-01	390	2.84E-02	657	6.63E-02	339	7.96E-01	1000	3.73E-05	214	**3**	8.15E-02	351	3.96E-03	294	7.32E-01	859	4.97E-02	911	9.24E-01	1139	4.24E-02	359	7.03E-01	973	**3**	**6**	**662.00**
**8725**	**NM_003796**	**C19orf2**	1.97E-02	250	7.18E-01	967	5.69E-01	696	1.33E-05	38	9.85E-01	1134	9.99E-01	1158	1.92E-01	569	7.46E-01	967	8.38E-04	349	**3**	2.28E-01	538	3.84E-03	291	6.89E-01	818	1.77E-04	558	2.42E-01	931	3.29E-02	317	7.91E-01	1022	**3**	**6**	**662.69**
**25836**	**NM_133433**	**nipbl**	4.83E-01	807	7.98E-01	1023	2.68E-01	365	5.31E-04	133	4.22E-01	550	2.57E-01	904	3.41E-01	744	9.82E-02	365	4.51E-01	960	**1**	3.69E-02	251	2.72E-01	844	6.18E-01	744	8.48E-01	1140	1.38E-03	444	4.77E-01	826	1.37E-01	509	**2**	**3**	**663.06**
**11120**	**NM_078476**	**btn2a1**	2.81E-01	674	1.78E-01	618	5.73E-01	704	1.61E-01	663	6.78E-01	838	6.80E-02	742	2.42E-01	644	7.05E-01	939	9.02E-04	356	**1**	1.80E-02	172	2.28E-02	458	4.86E-01	589	4.86E-01	1067	3.19E-01	958	8.47E-02	450	3.61E-01	740	**2**	**3**	**663.25**
**78996**	**NM_024006**	**VKORC1**	8.57E-01	1021	3.85E-02	410	7.80E-01	932	1.99E-03	199	8.52E-01	1008	1.33E-02	591	7.13E-01	1005	6.17E-01	871	1.29E-04	261	**4**	1.20E-01	410	3.00E-02	498	2.68E-01	242	2.60E-05	419	2.93E-02	714	6.03E-01	905	9.54E-01	1130	**3**	**7**	**663.50**
**10457**	**NM_001005340**	**GPNMB**	1.75E-02	229	4.51E-01	798	9.21E-01	1087	9.23E-01	1119	4.29E-01	558	8.88E-16	5	2.52E-01	657	4.30E-01	735	8.36E-02	728	**2**	9.30E-01	1108	1.88E-02	435	7.95E-01	932	1.55E-02	828	4.05E-03	517	3.84E-02	341	1.56E-01	541	**4**	**6**	**663.63**
**7035**	**NM_001128148**	**TFRC**	8.70E-01	1034	6.32E-01	902	1.07E-02	64	7.56E-01	1022	4.70E-01	605	3.03E-01	930	1.71E-03	40	1.93E-02	156	9.65E-02	741	**3**	8.03E-01	1015	2.03E-01	762	9.51E-01	1103	5.27E-05	475	4.52E-01	1005	3.53E-03	139	2.26E-01	630	**2**	**5**	**663.94**
**23513**	**NM_182706**	**scrib**	6.21E-01	867	7.38E-03	136	8.19E-01	981	8.26E-01	1065	8.39E-01	992	1.35E-05	232	7.85E-01	1045	7.51E-01	972	3.47E-01	910	**2**	1.33E-02	143	8.18E-03	354	1.81E-01	141	7.73E-01	1133	1.05E-06	118	9.00E-01	1085	1.03E-01	451	**3**	**5**	**664.06**
**57178**	**NM_020445**	**ACTR3B**	3.28E-03	91	2.01E-01	632	1.83E-01	285	3.43E-01	798	7.02E-01	870	1.09E-08	79	2.89E-01	700	7.07E-01	941	4.39E-01	952	**2**	6.09E-01	887	6.81E-05	60	9.99E-01	1156	8.40E-01	1139	5.27E-02	782	1.87E-03	114	9.78E-01	1146	**2**	**4**	**664.50**
**25901**	**NM_015439**	**CCDC28A**	4.55E-01	790	3.25E-01	720	4.77E-02	156	5.65E-01	925	7.38E-01	893	2.69E-03	479	3.75E-01	787	3.87E-02	229	6.76E-01	1040	**3**	1.92E-01	509	3.61E-02	520	7.27E-01	853	3.42E-04	602	9.67E-01	1148	3.97E-01	769	2.69E-02	213	**3**	**6**	**664.56**
**2533**	**NM_005255**	**GAK**	3.76E-02	394	8.88E-01	1088	7.57E-02	190	7.17E-01	1000	1.07E-01	175	2.05E-04	349	1.68E-01	534	7.23E-01	955	6.86E-02	705	**2**	4.33E-01	758	2.14E-02	450	9.50E-01	1101	4.34E-01	1055	2.84E-01	943	4.60E-07	6	6.34E-01	931	**2**	**4**	**664.63**
**5042**	**NM_000430**	**Pafah1b1**	2.57E-01	652	3.46E-02	379	6.34E-01	772	3.93E-03	244	1.08E-01	176	7.42E-02	751	3.71E-01	783	2.41E-01	569	1.08E-02	537	**3**	8.06E-01	1018	2.34E-01	801	8.76E-01	1007	2.69E-02	864	1.15E-01	853	7.96E-02	442	4.30E-01	791	**1**	**4**	**664.94**
**3178**	**NM_031243**	**HNRNPA2B**	1.87E-01	596	7.62E-01	998	9.36E-01	1104	6.09E-03	281	6.82E-01	844	6.73E-01	1074	1.49E-01	498	5.79E-01	840	3.28E-01	901	**1**	8.77E-01	1074	5.39E-02	563	2.10E-01	174	2.61E-05	420	1.10E-01	843	3.80E-03	146	4.34E-02	288	**3**	**4**	**665.25**
**11100**	**NM_144732**	**HNRNPUL1**	2.19E-01	623	2.80E-01	690	1.00E+00	1158	5.98E-02	531	1.38E-01	200	1.31E-01	823	7.63E-02	365	4.76E-01	763	4.17E-05	219	**1**	9.38E-02	373	9.44E-01	1148	3.50E-01	376	2.25E-02	855	5.85E-03	554	5.16E-01	846	9.41E-01	1122	**2**	**3**	**665.38**
**55197**	**NM_024662**	**Nat10**	2.24E-02	285	4.14E-01	774	4.95E-01	605	1.29E-02	351	8.92E-01	1045	1.40E-03	444	6.21E-02	327	3.52E-02	222	1.02E-04	251	**5**	7.20E-01	953	2.82E-01	855	6.31E-01	757	6.45E-01	1105	6.90E-01	1077	6.99E-01	968	2.39E-01	639	**0**	**5**	**666.13**
**1432**	**NM_001316**	**CSE1L**	7.36E-03	146	5.88E-01	874	7.87E-01	939	3.23E-01	782	1.32E-01	196	8.78E-03	551	2.11E-01	601	3.98E-01	713	3.46E-01	909	**2**	4.85E-03	101	3.81E-01	953	5.58E-01	679	6.48E-05	487	9.23E-01	1138	3.94E-01	768	4.77E-01	823	**2**	**4**	**666.25**
**27230**	**NM_014445**	**serp1**	3.73E-01	745	9.38E-01	1116	3.02E-01	409	2.25E-04	95	7.34E-01	889	9.14E-01	1139	5.79E-02	307	2.08E-01	531	2.75E-07	90	**2**	5.68E-01	861	4.43E-04	142	9.16E-01	1047	5.43E-03	753	1.11E-01	847	3.58E-01	735	6.81E-01	956	**2**	**4**	**666.38**
**10553**	**NM_001098522**	**htatip2**	4.97E-01	814	2.37E-01	658	8.33E-01	995	9.26E-03	324	5.64E-01	705	2.04E-06	185	8.84E-01	1091	3.88E-01	702	5.45E-01	995	**2**	7.92E-01	1009	7.83E-02	605	9.04E-01	1033	6.05E-05	482	2.89E-02	712	5.67E-04	82	4.10E-02	277	**4**	**6**	**666.81**
**11196**	**NM_007190**	**SEC23IP**	4.20E-01	769	2.80E-01	691	8.94E-01	1064	6.19E-01	954	7.18E-01	880	3.66E-01	965	4.19E-02	244	6.75E-01	910	1.06E-06	111	**2**	7.37E-03	116	6.38E-03	333	9.32E-01	1074	1.04E-04	519	1.99E-05	217	6.78E-01	957	5.43E-01	868	**4**	**6**	**667.00**
**2870**	**NM_199054**	**MKNK2**	6.70E-02	491	3.02E-01	705	4.69E-01	576	5.60E-07	7	9.99E-01	1158	4.30E-01	1000	9.93E-01	1155	5.70E-02	282	7.90E-01	1077	**1**	7.68E-01	998	3.35E-03	274	1.26E-01	71	1.89E-03	706	4.83E-01	1019	7.41E-02	434	3.37E-01	721	**2**	**3**	**667.13**
**6793**	**NM_004177**	**STX3**	2.92E-02	332	5.62E-01	857	4.16E-01	512	6.04E-01	946	2.45E-01	339	2.74E-08	94	5.78E-02	305	1.81E-01	499	7.77E-01	1071	**2**	5.45E-01	849	2.86E-01	862	9.54E-01	1108	4.57E-01	1062	5.97E-01	1053	1.90E-01	582	2.62E-02	206	**1**	**3**	**667.31**

10363	NM_018200	Hmg20a	6.45E-01	878	3.44E-02	377	9.61E-01	1128	6.54E-02	538	5.85E-01	730	2.72E-01	911	1.14E-02	114	8.82E-01	1062	6.29E-04	328	3	6.02E-01	880	2.87E-01	865	7.83E-02	38	4.45E-04	621	2.76E-03	484	6.21E-01	918	4.53E-01	806	2	5	667.38
55280	NM_018321	BRIX1	3.18E-01	710	1.62E-02	224	4.80E-01	586	3.20E-02	439	5.39E-01	672	4.08E-01	988	3.67E-01	778	2.73E-01	595	7.13E-01	1051	2	2.36E-01	551	3.96E-02	529	6.74E-01	804	5.43E-04	637	5.19E-01	1030	8.93E-01	1081	3.27E-04	7	3	5	667.63
23649	NM_002689	pola2	8.19E-01	1002	1.21E-03	46	9.94E-01	1154	2.59E-01	740	4.90E-01	631	5.08E-02	714	4.77E-01	865	2.49E-02	184	2.83E-04	294	3	9.10E-01	1092	7.04E-01	1078	6.00E-01	722	4.62E-06	293	1.07E-05	199	1.40E-01	523	9.87E-01	1150	2	5	667.94
9877	NM_014827	Zc3h11a	1.26E-02	196	6.91E-01	947	5.09E-01	626	2.78E-01	754	5.13E-01	647	4.55E-01	1007	5.26E-01	903	1.90E-01	514	5.51E-01	997	1	9.81E-02	378	8.25E-03	355	6.33E-01	759	3.65E-04	607	4.23E-02	754	6.08E-01	908	5.36E-02	338	3	4	668.13
53838	NM_022338	C11orf24	3.97E-01	761	6.55E-01	920	9.13E-03	56	7.96E-01	1049	8.46E-01	1000	8.61E-01	1123	2.42E-02	182	2.88E-02	199	7.57E-03	503	4	8.29E-01	1041	5.21E-03	318	6.86E-01	814	1.47E-01	973	3.25E-01	960	2.48E-01	637	1.74E-02	155	2	6	668.19
51562	NM_001144891	Mbip	9.43E-01	1093	1.46E-02	207	2.79E-01	376	3.04E-01	768	4.80E-01	620	9.36E-01	1143	3.29E-02	216	2.95E-02	202	7.33E-01	1055	3	3.29E-01	658	2.10E-01	770	9.99E-01	1157	3.08E-02	876	7.66E-04	407	3.17E-01	700	9.86E-02	444	2	5	668.25
55245	NM_018269	ADI1	4.81E-01	806	4.69E-01	810	4.68E-02	154	4.63E-01	862	4.76E-01	611	2.06E-08	90	4.32E-02	250	4.80E-01	765	6.08E-02	694	3	7.71E-01	1001	6.98E-01	1074	2.89E-01	271	3.18E-03	725	9.51E-03	594	9.63E-01	1136	5.13E-01	850	2	5	668.31
10106	XM_001720210	CTDSP2	7.36E-01	933	8.05E-01	1027	2.73E-03	24	4.11E-01	837	5.57E-01	695	5.62E-05	291	6.85E-01	993	2.27E-02	171	3.25E-01	898	3	2.36E-01	548	9.55E-01	1151	9.01E-01	1029	1.12E-03	678	4.61E-01	1011	1.34E-03	103	4.81E-02	306	3	6	668.44
83461	NM_031449	Zmiz2	8.02E-01	985	4.55E-02	461	8.54E-01	1020	8.37E-01	1068	9.22E-01	1068	6.59E-03	532	1.14E-01	445	7.37E-01	961	1.66E-05	187	3	1.14E-01	406	1.51E-02	411	3.55E-01	387	9.54E-01	1157	1.44E-07	67	7.65E-01	1005	1.59E-01	545	2	5	669.06
54465	NM_019002	etaa1	7.25E-01	922	4.80E-02	483	8.15E-01	975	7.65E-02	551	8.48E-02	147	3.62E-01	962	5.00E-03	75	6.58E-01	895	4.86E-01	975	2	2.13E-01	524	1.72E-01	725	8.08E-01	947	3.55E-02	894	1.89E-01	903	3.16E-01	699	2.44E-03	33	2	4	669.38
55322	NM_018359	UFSP2	3.71E-02	387	1.48E-02	209	9.76E-01	1143	3.09E-01	776	1.82E-01	250	1.34E-01	826	5.11E-02	276	5.05E-02	272	1.06E-04	252	3	4.37E-01	761	3.43E-01	922	8.60E-01	985	3.44E-04	603	4.01E-01	982	8.80E-01	1073	7.51E-01	998	1	4	669.69
79866	NM_024845	NAT15	5.71E-01	845	5.05E-01	830	2.82E-01	382	8.98E-01	1100	5.29E-01	660	6.37E-01	1062	3.48E-01	756	2.32E-02	175	3.36E-01	904	1	1.78E-02	170	2.70E-01	841	2.71E-01	247	1.72E-04	554	1.61E-01	891	5.29E-01	855	1.02E-01	449	2	3	670.06
55968	NM_001031710	KLHL7	1.04E-02	174	6.74E-01	929	8.48E-01	1011	3.69E-02	461	4.81E-01	622	1.81E-02	621	2.44E-02	183	2.32E-01	559	9.21E-11	16	5	5.23E-01	829	7.86E-02	607	9.89E-01	1148	5.54E-01	1085	3.00E-01	952	5.32E-01	857	2.68E-01	669	0	5	670.19
6502	NM_006516	slc2a1	6.45E-01	879	3.02E-02	347	6.66E-01	811	5.49E-02	522	9.24E-01	1071	1.89E-01	861	3.20E-01	725	1.37E-02	131	5.28E-01	990	2	1.90E-03	61	3.82E-02	526	8.66E-01	992	5.25E-02	914	4.46E-04	370	3.47E-01	727	4.45E-01	802	3	5	670.56
4719	NM_182966	Nedd9	9.01E-03	164	2.54E-01	675	8.72E-01	1039	9.49E-03	326	8.10E-02	144	6.08E-03	528	2.90E-01	701	1.72E-02	148	4.06E-03	456	5	7.37E-01	965	8.33E-01	1117	4.84E-01	586	1.27E-01	964	4.40E-01	1003	6.02E-01	904	8.14E-01	1029	0	5	671.81
9734	NM_014707	hdac9	3.20E-02	352	6.23E-01	898	9.86E-01	1148	8.76E-01	1087	2.17E-01	295	3.97E-02	691	5.30E-04	17	9.56E-01	1120	8.31E-02	727	3	4.65E-01	784	4.80E-02	553	3.00E-01	292	3.85E-01	1045	8.10E-03	580	1.75E-01	562	2.01E-01	603	2	5	672.13
4665	NM_005968	hnrnpm	3.61E-01	736	4.24E-03	91	8.85E-01	1054	5.15E-02	517	7.33E-01	888	2.36E-01	888	1.64E-01	526	9.32E-01	1098	4.87E-03	475	2	1.65E-01	471	6.57E-02	581	1.00E+00	1158	1.73E-02	834	4.43E-02	762	1.24E-01	509	2.42E-02	196	3	5	674.00
3E+05	NM_199294	Apitd1	2.76E-02	324	5.75E-01	864	8.34E-01	997	8.59E-01	1079	4.18E-01	544	2.80E-01	921	8.15E-01	1066	8.02E-01	1007	2.76E-02	608	2	7.53E-01	985	3.95E-01	963	1.56E-01	104	1.59E-05	381	1.00E-01	832	7.78E-06	16	8.22E-03	95	3	5	674.13
1955	NM_004094	EIF2S1	2.16E-02	276	5.92E-01	876	8.55E-01	1021	4.80E-01	872	4.69E-01	603	1.89E-05	250	7.04E-01	1000	1.21E-01	402	1.69E-03	400	3	3.78E-01	710	3.58E-02	517	7.98E-01	937	1.95E-01	990	4.79E-06	170	2.52E-01	643	9.54E-01	1131	2	5	674.88
51606	NM_213620	ATP6V1H	1.88E-03	71	5.15E-01	836	2.10E-02	84	3.63E-01	811	7.88E-01	947	8.14E-01	1109	1.96E-01	578	3.73E-01	687	4.27E-01	947	2	2.35E-01	547	1.31E-01	686	2.35E-01	198	8.42E-03	784	8.81E-02	823	5.04E-01	839	5.14E-01	851	1	3	674.88
64236	NM_022473	ZFP106	3.33E-01	722	5.83E-03	115	6.65E-01	808	3.79E-02	467	1.49E-01	216	1.23E-02	583	4.18E-03	70	3.40E-01	655	2.14E-06	127	5	7.63E-01	991	2.79E-01	851	9.67E-01	1126	3.01E-01	1021	7.80E-01	1101	7.90E-01	1023	6.74E-01	949	0	5	676.56
6386	NM_001143998	sec14l1	8.20E-04	43	2.03E-01	634	8.87E-01	1055	7.94E-01	1048	1.84E-01	251	3.84E-05	276	8.98E-01	1103	8.90E-01	1067	6.96E-01	1045	2	6.36E-01	899	1.76E-04	98	6.71E-01	799	2.83E-02	868	2.19E-07	75	5.86E-01	898	2.72E-01	674	3	5	677.06
51322	NM_100486	WAC	2.06E-02	260	2.22E-01	643	9.11E-01	1079	3.08E-01	773	6.54E-01	811	5.17E-07	156	5.35E-01	911	7.16E-01	944	1.65E-01	799	2	4.02E-01	734	1.35E-01	691	7.66E-01	898	1.15E-03	679	5.66E-01	1043	9.33E-03	192	2.83E-02	220	3	5	677.06
3303	NM_005345	HSPA1B	1.92E-02	248	4.38E-01	790	3.65E-01	470	1.20E-02	346	5.61E-01	701	6.93E-04	408	3.68E-01	779	7.69E-01	986	1.57E-01	789	3	9.44E-01	1118	3.12E-01	897	8.72E-01	1001	8.89E-06	338	4.47E-02	763	9.09E-01	1093	1.00E-02	107	3	6	677.13
9337	NM_004779	cnot8	3.81E-02	399	7.16E-01	966	2.92E-01	392	1.67E-02	368	6.17E-01	770	2.37E-03	472	4.53E-01	849	7.18E-01	948	4.30E-02	662	4	6.56E-01	908	2.30E-01	796	9.03E-01	1032	1.01E-06	204	4.10E-01	986	8.45E-01	1050	2.57E-03	34	2	6	677.25
7107	NM_003274	TRAPPC10	4.94E-01	811	1.98E-02	257	6.72E-01	821	4.61E-01	860	7.55E-01	907	4.04E-01	986	3.69E-01	782	2.15E-03	54	1.33E-02	556	3	8.00E-02	349	7.27E-03	343	6.42E-01	771	8.83E-01	1148	9.15E-02	826	1.87E-02	261	9.10E-01	1107	2	5	677.44
4013	NM_000428	LTBP2	9.31E-01	1082	8.22E-01	1037	1.54E-01	252	8.43E-01	1070	9.45E-01	1094	3.71E-01	969	3.02E-01	712	9.37E-01	1100	4.68E-07	106	1	4.05E-01	738	2.63E-01	835	2.90E-01	275	1.13E-05	358	5.80E-03	553	4.03E-02	348	5.03E-02	321	3	4	678.13
1845	NM_001949	E2F3	2.85E-02	329	3.17E-02	361	8.40E-01	1004	8.92E-02	575	6.59E-01	820	4.33E-01	1002	7.40E-01	1017	7.50E-01	970	8.67E-02	730	2	3.90E-01	720	1.91E-01	749	4.36E-01	523	1.76E-01	980	9.48E-05	303	4.03E-02	349	9.05E-02	426	2	4	678.63
4690	NM_005381	ncl	1.50E-02	213	6.81E-01	937	8.46E-01	1008	6.53E-01	970	9.86E-02	162	4.56E-01	1009	3.07E-02	206	3.18E-01	636	8.24E-02	725	2	8.25E-01	1035	9.21E-03	368	4.55E-01	552	6.11E-05	483	2.78E-03	487	6.80E-01	958	9.29E-01	1116	3	5	679.06
25972	NM_014044	UNC50	3.72E-02	391	5.60E-01	856	7.71E-01	920	2.97E-02	427	6.93E-01	857	2.02E-02	628	6.70E-01	985	4.50E-01	749	5.82E-10	28	4	9.78E-01	1141	6.06E-02	573	5.21E-01	638	2.99E-05	433	7.66E-04	408	3.39E-01	719	9.55E-01	1132	2	6	680.31
4303	NM_002432	MNDA	4.25E-02	428	8.17E-01	1034	3.90E-01	488	5.67E-01	926	2.90E-01	398	5.20E-03	520	7.40E-02	358	5.75E-01	836	3.02E-04	298	3	7.24E-01	958	6.55E-01	1066	5.48E-01	664	5.11E-04	635	2.80E-02	707	1.57E-01	540	8.38E-01	1049	2	5	681.56
10165	NM_001160210	SLC25A13	3.83E-02	400	6.50E-01	915	5.94E-01	728	8.63E-02	571	7.52E-01	905	8.38E-01	1115	5.49E-01	918	9.70E-01	1130	8.16E-04	346	2	1.44E-01	440	9.17E-02	633	3.29E-01	341	8.91E-07	197	7.71E-05	294	8.07E-01	1035	6.72E-01	946	2	4	682.13
51008	NM_015947	ASCC1	1.16E-03	51	6.62E-01	924	8.16E-01	978	3.29E-01	788	5.75E-01	719	5.20E-01	1027	2.05E-03	44	2.73E-01	597	1.54E-02	569	3	4.51E-01	772	2.22E-03	237	6.48E-01	776	8.33E-02	946	1.12E-03	432	9.14E-01	1097	6.86E-01	959	2	5	682.25
10130	NM_005742	pdia6	5.31E-01	830	8.65E-01	1068	8.56E-01	1024	4.06E-05	60	6.44E-01	802	2.41E-03	475	1.54E-02	135	2.16E-01	541	1.68E-08	56	4	8.47E-01	1052	1.62E-02	415	7.81E-01	915	1.39E-04	540	2.72E-01	940	6.19E-01	913	9.88E-01	1151	2	6	682.31
2639	NM_000170	Gldc	9.60E-01	1114	3.26E-03	79	6.38E-01	777	2.99E-01	766	4.21E-01	546	1.23E-04	327	4.35E-01	836	1.88E-02	155	7.59E-01	1066	3	4.40E-01	764	3.80E-01	952	7.20E-01	851	8.98E-03	789	5.56E-04	381	5.58E-01	876	2.41E-01	642	2	5	682.56
7157	NM_001126113	tp53	9.96E-01	1149	5.77E-01	866	4.56E-01	557	8.86E-02	574	3.99E-02	80	3.60E-01	959	1.59E-01	517	8.07E-01	1013	2.13E-01	835	1	3.40E-01	670	5.22E-02	558	7.05E-01	835	8.48E-04	658	2.64E-04	349	4.73E-01	822	1.19E-01	482	2	3	682.75
56882	NM_020123	TM9SF3	2.66E-02	321	7.33E-01	977	8.21E-01	983	8.02E-06	31	9.03E-01	1053	6.08E-04	397	7.16E-02	350	3.90E-01	704	8.70E-01	1106	3	4.73E-03	98	5.61E-03	322	9.68E-01	1127	3.14E-01	1027	1.07E-01	838	7.23E-01	981	2.16E-01	622	2	5	683.56
54976	NM_017906	PAK1IP1	5.77E-01	850	1.53E-02	215	8.14E-01	972	6.53E-03	289	3.05E-01	413	4.57E-01	1010	9.91E-01	1153	4.56E-01	752	9.98E-01	1158	2	6.79E-02	320	3.29E-01	910	3.81E-01	431	9.60E-04	666	2.28E-02	682	6.27E-01	922	2.44E-02	197	3	5	683.75
808	NM_005184	CALM1	7.86E-02	511	8.71E-01	1072	7.05E-01	854	9.11E-01	1110	2.98E-01	407	2.30E-04	352	8.34E-02	388	7.83E-01	992	4.11E-01	939	1	4.40E-01	765	2.25E-02	456	3.16E-01	318	2.48E-05	417	3.09E-01	957	5.81E-01	894	1.39E-01	515	2	3	684.19
96764	NM_001144945	MYL12B	4.41E-01	779	4.14E-02	432	5.17E-01	636	1.42E-01	640	9.40E-01	1088	6.69E-04	404	4.72E-01	862	7.86E-02	326	2.60E-03	422	3	6.55E-01	907	1.67E-01	720	2.02E-01	160	8.27E-02	945	8.50E-01	1116	2.92E-01	678	4.96E-01	837	0	3	684.50
7748	NM_001130520	ZNF195	3.65E-01	739	2.23E-02	275	9.29E-01	1097	4.53E-04	129	3.34E-01	448	5.38E-01	1030	3.90E-01	801	8.90E-01	1068	6.96E-01	1046	2	4.14E-01	743	1.16E-01	665	4.36E-01	525	2.30E-07	123	3.71E-01	971	7.43E-03	182	9.28E-01	1114	2	4	684.75
920	NM_000616	CD4	1.90E-01	600	3.64E-02	392	7.76E-01	928	7.99E-02	558	1.40E-01	203	8.99E-02	778	4.47E-01	845	8.80E-01	1060	3.10E-02	620	2	6.54E-01	906	5.31E-01	1023	6.68E-01	796	3.23E-02	884	2.10E-01	913	4.85E-06	13	9.61E-02	438	2	4	684.81
10211	NM_005803	flot1	4.26E-02	429	4.33E-01	783	5.65E-01	692	3.23E-01	783	4.26E-01	554	9.19E-02	780	5.53E-02	294	3.73E-01	688	1.15E-02	541	2	8.25E-01	1037	8.87E-01	1134	2.10E-01	176	3.85E-04	611	5.46E-01	1038	3.09E-02	310	9.11E-01	1108	2	4	684.88
8648	NM_147233	NCOA1	1.17E-03	53	8.66E-01	1069	4.58E-01	559	3.09E-01	775	4.61E-01	594	3.77E-01	972	9.97E-01	1157	9.99E-01	1156	7.71E-01	1068	1	1.25E-02	141	1.01E-02	375	4.89E-01	595	8.12E-03	782	1.09E-01	840	5.68E-06	15	4.64E-01	814	4	5	685.31
22985	NM_001164817	ACIN1	4.05E-02	409	9.92E-01	1151	6.55E-01	798	1.98E-04	87	7.89E-01	949	6.60E-05	301	3.58E-01	768	5.65E-01	832	5.38E-01	994	3	2.45E-02	206	9.74E-03	373	7.11E-01	841	3.20E-02	881	3.28E-02	725	8.78E-01	1071	1.82E-01	582	4	7	685.50
11221	NM_144729	Dusp10	1.91E-01	603	4.46E-01	794	8.59E-01	1027	9.94E-01	1155	4.16E-01	541	8.51E-03	549	2.76E-01	689	5.56E-07	3	7.42E-04	338	3	3.92E-01	722	6.16E-01	1055	6.73E-01	801	3.72E-01	1041	5.37E-01	1034	9.39E-05	48	1.73E-01	570	1	4	685.63
80323	NM_025215	PUS1	9.12E-01	1063	9.10E-01	1103	3.36E-02	121	6.78E-01	981	3.39E-01	455	2.04E-01	869	2.15E-04	11	9.77E-01	1139	2.16E-01	837	2	9.06E-01	1087	2.92E-01	875	3.95E-01	457	3.98E-02	900	2.11E-10	11	4.31E-01	790	4.46E-02	291	3	5	686.88
8525	NM_001105540	DGKZ	3.72E-02	389	9.75E-01	1140	8.00E-01	954	2.35E-01	721	7.93E-01	952	7.62E-05	310	5.66E-01	928	5.13E-01	790	4.12E-03	457	3	2.62E-01	579	2.18E-02	452	9.24E-01	1062	1.83E-01	983	3.09E-05	238	1.86E-01	579	1.16E-01	473	2	5	687.94
6500	NM_005983	Skp2	6.41E-01	874	1.81E-01	621	1.09E-01	213	8.68E-03	317	8.96E-01	1049	9.74E-01	1149	1.58E-01	513	7.05E-01	938	4.88E-01	977	1	6.09E-01	886	7.90E-03	350	5.26E-01	646	8.44E-03	785	7.72E-02	813	1.22E-01	506	6.79E-02	378	2	3	688.44
51035	NM_015853	Ubxn1	5.19E-01	826	4.87E-01	817	4.25E-01	524	2.73E-01	750	4.06E-01	528	3.18E-02	664	2.79E-01	692	1.75E-01	490	5.67E-04	322	2	8.33E-01	1044	2.25E-03	240	9.89E-01	1149	7.32E-02	938	1.53E-01	883	2.93E-02	305	5.10E-01	847	2	4	688.69
6615	NM_003082	SNAPC1	8.83E-02	521	2.63E-02	311	9.83E-01	1146	7.74E-01	1032	3.76E-01	495	8.76E-13	20	1.38E-01	482	1.14E-02	118	2.61E-01	857	3	9.92E-01	1151	2.85E-01	861	8.61E-01	988	2.23E-02	854	3.41E-02	728	2.55E-01	647	4.89E-01	831	2	5	690.13
29775	NM_014550	CARD10	8.58E-01	1025	3.71E-02	398	4.66E-01	573	6.15E-03	283	1.53E-01	218	8.58E-01	1122	1.63E-01	524	9.88E-01	1149	2.07E-04	280	3	3.15E-01	646	4.07E-01	973	3.78E-01	424	3.26E-02	885	7.07E-02	804	6.35E-01	930	4.61E-01	811	1	4	690.31
2778	NM_002074	GNB1	3.65E-01	741	2.25E-01	646	5.42E-01	668	4.38E-01	849	5.97E-01	745	1.55E-02	609	6.20E-01	958	4.09E-01	723	8.60E-01	1099	1	7.19E-01	950	1.78E-02	427	8.60E-01	984	3.40E-04	601	2.18E-12	2	9.85E-02	474	1.75E-01	574	3	4	690.63
5281	NM_002647	PIK3C3	7.83E-01	969	4.13E-01	773	5.27E-01	647	4.69E-03	259	9.94E-01	1150	4.89E-02	709	3.50E-01	759	3.93E-01	705	7.70E-05	242	3	2.86E-01	611	9.14E-03	367	8.11E-01	951	6.61E-03	768	1.03E-02	601	5.48E-01	871	2.79E-01	679	3	6	691.31
23186	NM_015156	RCOR1	7.45E-01	944	9.95E-01	1157	4.89E-02	158	9.64E-01	1143	6.39E-01	796	6.23E-09	68	3.87E-03	67	8.76E-01	1054	2.27E-02	590	4	8.54E-01	1057	3.64E-03	287	2.61E-01	233	2.68E-02	863	2.04E-02	669	8.45E-01	1051	6.32E-01	927	3	7	691.50
7109	NM_007114	TMF1	8.87E-02	524	2.35E-01	656	5.25E-01	645	3.59E-01	808	6.95E-01	858	2.79E-02	653	1.68E-01	532	7.90E-02	327	4.51E-02	664	2	2.08E-01	520	5.17E-01	1016	8.98E-01	1028	3.40E-01	1031	3.81E-02	742	4.39E-02	365	3.13E-01	704	2	4	692.06
10461	NM_006343	MERTK	2.51E-05	12	7.02E-01	955	6.32E-01	770	9.20E-01	1115	7.63E-01	915	1.20E-01	809	3.89E-01	800	1.39E-01	432	1.45E-03	388	2	4.77E-01	797	2.90E-01	872	9.66E-01	1124	3.37E-03	730	1.03E-03	427	1.89E-02	262	2.68E-01	671	3	5	692.44
6128	NM_000971	RPL7	1.11E-02	180	7.56E-02	526	6.73E-01	822	6.00E-01	945	9.49E-01	1099	3.57E-04	377	1.82E-01	551	4.17E-01	731	1.19E-01	764	2	5.61E-01	856	6.71E-02	588	9.94E-01	1152	1.76E-05	389	1.41E-02	628	6.80E-02	422	8.38E-01	1051	2	4	692.56
2618	NM_032638	GATA2	8.88E-01	1050	3.66E-02	394	9.39E-01	1107	7.02E-03	300	2.36E-01	322	9.47E-01	1144	1.71E-01	536	7.94E-01	998	6.19E-04	326	3	6.14E-01	888	2.58E-01	826	6.09E-01	734	3.80E-07	146	9.26E-01	1140	6.40E-01	932	3.26E-02	243	2	5	692.88
27091	NM_145811	CACNG5	8.86E-02	523	3.84E-02	408	3.27E-01	431	5.76E-01	935	6.05E-02	116	9.90E-04	430	3.69E-01	781	2.81E-01	600	1.17E-02	543	3	5.07E-01	815	9.43E-02	636	9.09E-01	1039	5.53E-01	1084	4.17E-01	990	3.62E-01	737	8.11E-01	1028	0	3	693.50
5878	NM_002872	rac2	2.57E-02	313	6.86E-01	942	3.93E-01	491	4.55E-02	495	6.17E-01	769	9.26E-01	1142	1.78E-01	547	7.30E-01	958	4.55E-01	964	2	2.35E-01	545	6.62E-01	1068	4.35E-01	522	6.51E-03	763	4.78E-01	1015	8.79E-02	457	1.03E-02	111	2	4	693.88
51133	NM_016121	KCTD3	7.70E-01	959	6.65E-01	925	2.47E-03	23	3.94E-02	476	1.03E-01	170	6.41E-01	1063	9.35E-01	1124	7.18E-01	947	9.52E-03	525	3	3.26E-01	652	3.41E-01	920	8.69E-01	997	2.50E-04	584	2.00E-08	43	9.94E-01	1157	1.56E-01	538	2	5	693.94
10943	NM_078630	MSL3	5.85E-01	853	6.52E-01	918	5.09E-01	627	9.54E-01	1134	4.85E-01	627	4.23E-01	994	3.75E-02	228	4.45E-01	744	1.11E-01	756	1	3.40E-01	671	1.42E-01	705	2.53E-01	216	3.01E-01	1020	4.02E-06	167	5.27E-02	388	8.59E-01	1068	1	2	694.75
3176	NM_031157	HNRNPA1	2.02E-01	610	4.42E-01	793	6.08E-01	749	9.41E-01	1127	9.27E-01	1076	1.73E-01	852	2.33E-01	628	4.23E-01	733	9.38E-02	737	0	5.30E-01	835	1.37E-01	698	8.63E-01	989	8.68E-07	196	4.20E-07	91	3.42E-01	724	4.48E-02	293	3	3	695.69
6652	NM_003116	SPAG4	3.24E-02	355	9.89E-01	1148	1.24E-01	225	2.15E-01	702	4.22E-01	549	8.41E-03	548	2.08E-02	170	5.33E-01	804	7.30E-01	1053	3	1.05E-01	389	1.51E-01	710	9.66E-01	1121	1.27E-01	966	3.91E-05	254	9.33E-01	1110	8.16E-01	1031	1	4	695.94
10240	NM_005830	mRpS31	8.64E-01	1030	2.69E-02	317	4.27E-01	525	4.95E-02	504	6.96E-01	861	7.26E-01	1094	5.61E-01	925	6.57E-01	894	3.91E-01	928	2	4.32E-01	756	4.35E-02	542	2.36E-01	199	3.70E-07	145	2.74E-06	151	9.50E-01	1126	9.66E-01	1139	3	5	696.00
586	NM_005504	BCAT1	1.21E-02	189	8.50E-01	1061	5.90E-01	722	4.43E-01	852	9.50E-01	1100	9.61E-01	1146	7.66E-02	368	1.09E-01	384	1.89E-01	818	1	2.27E-01	537	3.55E-04	122	4.32E-01	516	2.97E-01	1018	2.26E-01	925	6.47E-01	935	1.15E-01	468	1	2	697.56
10284	NM_005870	SAP18	7.02E-01	911	1.53E-02	216	8.54E-01	1018	1.38E-03	188	5.71E-01	714	9.01E-01	1133	4.97E-01	875	6.78E-01	915	1.68E-03	399	3	5.56E-01	854	1.80E-01	731	7.16E-01	845	3.61E-04	606	1.24E-01	862	5.95E-05	41	5.37E-01	864	2	5	698.25
1871	NM_004424	E4F1	8.34E-02	516	8.56E-01	1065	8.25E-01	990	3.34E-02	448	3.20E-02	65	1.45E-04	334	9.92E-01	1154	8.62E-01	1045	6.12E-04	325	4	3.39E-01	669	9.18E-01	1144	9.51E-01	1104	1.33E-02	820	2.61E-05	227	8.77E-02	456	4.90E-01	832	2	6	699.63
65057	NM_023075	MPPE1	5.77E-01	849	4.01E-02	422	8.67E-01	1036	5.90E-06	25	8.44E-01	996	5.17E-03	517	7.79E-01	1042	8.89E-01	1066	2.79E-01	868	3	3.05E-01	636	5.33E-02	562	3.45E-01	367	3.94E-03	740	8.23E-01	1109	3.61E-02	334	2.31E-01	636	2	5	700.31
63892	NM_133171	ELMO2	5.93E-01	855	2.01E-04	17	5.00E-01	610	3.95E-01	827	3.68E-01	486	6.03E-01	1051	8.61E-02	393	8.52E-01	1034	4.69E-03	471	2	7.78E-01	1004	3.89E-03	293	7.91E-01	926	4.68E-02	909	1.61E-01	890	8.72E-02	454	7.35E-01	988	2	4	700.50
28959	NM_014020	TMEM176B	4.22E-02	426	8.16E-01	1033	5.09E-01	625	9.95E-04	173	4.72E-01	608	1.54E-01	836	1.03E-01	427	8.79E-01	1058	3.19E-01	895	2	7.83E-01	1006	2.49E-01	819	9.66E-01	1123	1.02E-07	93	3.09E-02	719	2.45E-01	634	3.84E-01	760	2	4	702.19
10668	NM_006568	cgrrf1	4.76E-01	802	4.43E-02	453	5.43E-01	669	5.56E-01	917	4.91E-01	634	8.76E-02	773	5.61E-02	299	5.81E-01	843	7.32E-04	335	2	3.70E-01	704	6.81E-02	592	6.98E-01	826	1.36E-02	823	3.51E-03	504	8.55E-01	1058	7.70E-01	1008	2	4	702.50
57730	NM_021190	ptbp2	6.99E-01	907	7.19E-01	968	2.60E-02	100	6.20E-01	955	5.07E-01	645	5.16E-02	715	1.02E-03	25	8.68E-01	1048	7.54E-01	1063	2	9.36E-02	372	6.29E-01	1057	3.26E-01	332	9.15E-03	792	8.41E-03	584	2.69E-01	659	7.87E-01	1019	2	4	702.56
7572	NM_006965	ZNF24	4.92E-02	466	6.37E-01	905	2.00E-01	303	2.29E-01	716	8.10E-01	969	6.36E-01	1060	1.94E-01	571	1.71E-01	484	7.38E-02	711	1	1.97E-01	513	3.36E-01	917	6.62E-01	790	2.64E-03	716	2.16E-02	678	1.68E-01	554	5.83E-01	897	2	3	703.13
54948	NM_017847	c1orf27	6.81E-01	899	3.88E-02	412	8.03E-01	961	6.92E-02	543	4.38E-01	571	5.49E-01	1036	6.60E-02	337	2.35E-01	562	7.34E-01	1056	1	5.98E-01	879	5.65E-01	1034	8.41E-01	972	3.90E-08	45	9.96E-02	830	8.46E-01	1052	7.32E-03	79	2	3	704.25
23339	NM_015289	VPS39	2.83E-02	328	8.34E-01	1049	3.17E-01	426	3.05E-01	770	3.34E-01	449	5.98E-01	1049	4.39E-01	837	6.74E-01	907	2.04E-04	278	2	1.35E-01	430	5.33E-01	1024	3.46E-01	370	3.21E-02	883	1.46E-03	450	8.89E-01	1079	6.56E-01	940	2	4	704.31
10144	NM_014883	Fam13a	6.62E-02	490	6.60E-03	122	4.10E-03	29	9.93E-01	1153	4.69E-01	602	3.17E-06	195	2.13E-01	606	3.47E-01	664	2.01E-01	829	3	4.88E-02	283	6.36E-01	1061	9.08E-01	1038	6.80E-01	1114	8.52E-01	1117	5.88E-01	900	8.59E-01	1069	1	4	704.50
8458	NM_003594	TTF2	8.77E-01	1042	1.67E-02	231	6.94E-01	850	2.58E-01	739	4.93E-02	101	3.75E-02	683	9.12E-01	1112	1.64E-01	470	1.29E-05	179	4	5.79E-01	868	1.33E-01	690	7.78E-01	911	1.81E-01	982	2.01E-01	907	9.54E-02	468	8.32E-01	1043	0	4	704.75

5902	NM_006743	RBM3	6.79E-01	898	3.71E-02	400	7.12E-01	863	1.47E-01	648	6.95E-01	859	2.37E-01	892	8.69E-01	1086	3.31E-01	647	1.11E-04	255	2	1.58E-02	157	2.22E-01	782	7.03E-01	832	1.12E-06	213	4.92E-02	775	8.53E-01	1056	6.07E-01	916	3	5	704.94
9679	NM_014661	FAM53B	3.60E-02	381	9.78E-01	1142	3.10E-01	419	3.18E-04	106	5.19E-01	656	2.86E-06	194	9.73E-01	1147	6.14E-02	295	1.65E-03	397	4	9.66E-01	1130	1.85E-01	740	9.18E-01	1053	2.88E-01	1015	5.63E-01	1042	1.29E-01	514	8.51E-01	1063	0	4	705.88
3839	NM_002270	tnpo1	4.55E-02	445	4.38E-01	789	9.94E-01	1155	5.72E-01	933	1.36E-01	199	8.45E-04	418	6.04E-01	950	5.66E-02	280	1.57E-01	790	2	2.19E-02	191	3.86E-01	955	8.12E-01	952	1.31E-03	688	9.46E-03	590	4.77E-01	827	9.60E-01	1135	3	5	706.06
5001	NM_001017962	P4HA1	7.99E-01	980	9.65E-01	1135	2.86E-01	384	5.12E-01	889	9.30E-01	1077	2.79E-02	654	4.85E-02	265	9.44E-01	1107	9.69E-03	527	3	5.28E-01	834	1.20E-01	670	3.24E-01	326	4.09E-08	48	3.13E-02	721	3.18E-01	702	7.12E-01	978	2	5	706.06
11182	NM_001145099	SLC2A6	3.58E-02	379	3.22E-01	719	7.50E-01	900	6.67E-03	292	9.47E-01	1098	1.15E-02	575	9.07E-01	1107	3.51E-01	669	9.93E-01	1157	3	3.26E-01	653	3.63E-03	285	9.80E-01	1136	5.63E-02	918	9.70E-06	196	1.18E-03	100	9.36E-01	1120	3	6	706.50
2766	NM_016592	Gnas	2.74E-01	665	2.04E-02	260	1.91E-01	293	9.15E-01	1112	8.24E-01	980	2.85E-01	923	2.73E-01	686	9.72E-01	1131	1.93E-07	87	2	7.49E-01	979	3.60E-01	934	1.28E-03	1	6.73E-01	1113	6.81E-05	290	7.46E-01	998	5.47E-01	872	2	4	707.75
27032	NM_014382	ATP2C1	1.81E-04	25	7.78E-01	1009	5.86E-01	717	4.62E-01	861	9.10E-01	1059	2.86E-03	486	6.29E-01	962	1.17E-01	395	1.62E-01	796	2	1.37E-01	432	1.48E-01	708	7.52E-01	881	3.30E-01	1028	1.73E-03	455	9.22E-02	464	8.46E-01	1058	1	3	708.50
26118	NM_015626	WSB1	2.74E-01	664	3.35E-01	730	5.36E-01	663	9.22E-01	1118	9.30E-01	1078	1.64E-01	847	2.39E-01	638	2.43E-01	572	4.06E-01	935	0	8.47E-03	123	1.11E-03	193	5.98E-01	719	1.80E-01	981	1.75E-01	896	7.69E-02	439	3.67E-01	746	2	2	708.88
476	NM_001160233	ATP1A1	3.95E-02	405	9.05E-01	1096	1.61E-01	259	1.04E-02	334	6.69E-01	830	1.64E-02	614	1.19E-02	116	6.90E-01	924	3.27E-01	900	4	9.10E-01	1091	1.87E-01	744	6.19E-01	745	7.13E-02	937	8.34E-01	1112	1.13E-01	494	3.71E-01	750	0	4	709.44
57226	NM_001033873	smagp	8.42E-01	1016	3.61E-02	391	1.80E-01	283	7.81E-01	1036	1.59E-01	224	3.07E-04	370	6.51E-01	974	6.68E-01	903	4.05E-01	934	2	5.90E-02	305	3.32E-01	913	6.88E-01	816	2.99E-01	1019	1.29E-01	866	8.46E-01	1054	3.55E-02	252	1	3	709.75
51463	NM_016334	GPR89A	2.18E-02	278	4.98E-01	824	7.19E-01	870	8.47E-02	568	6.85E-01	848	2.33E-04	354	3.33E-01	736	7.02E-02	313	1.58E-01	792	2	9.82E-01	1144	7.61E-01	1093	6.62E-01	788	3.20E-04	596	2.19E-04	340	5.35E-01	860	6.90E-01	962	2	4	710.38
83637	NM_032582	USP32	5.59E-02	478	7.95E-01	1021	8.46E-01	1010	8.44E-03	314	7.53E-01	906	5.84E-01	1045	4.38E-02	253	2.68E-02	190	6.74E-01	1039	3	2.47E-01	566	8.01E-01	1105	6.52E-02	32	8.91E-01	1149	4.88E-03	534	4.37E-01	797	6.48E-01	933	1	4	710.75
8045	NM_001143994	RASSF7	1.60E-01	581	2.31E-01	650	7.68E-01	919	2.96E-02	426	6.07E-01	755	4.72E-01	1014	1.14E-01	444	8.90E-01	1069	7.28E-05	240	2	9.05E-01	1085	1.81E-01	734	4.57E-01	557	3.70E-04	608	6.04E-01	1055	6.34E-01	927	4.93E-02	313	2	4	711.06
23463	NM_012405	icmt	1.61E-01	582	9.71E-01	1138	5.56E-01	682	4.60E-02	497	4.50E-01	582	7.19E-01	1092	5.70E-01	931	4.39E-01	738	3.88E-01	927	1	6.96E-01	936	2.28E-01	795	5.05E-01	616	1.69E-04	551	2.66E-02	698	2.33E-02	284	5.31E-02	335	3	4	711.50
25788	NM_012415	rad54b	9.90E-01	1144	2.55E-02	303	4.38E-01	538	8.49E-01	1073	6.29E-01	788	4.11E-03	509	6.96E-01	997	9.22E-01	1093	8.21E-04	347	3	2.76E-01	596	5.41E-02	564	8.58E-01	983	1.73E-03	701	8.30E-06	189	9.67E-01	1139	8.83E-02	421	2	5	711.56
7763	NM_006007	ZFAND5	1.18E-01	552	3.67E-01	747	9.20E-01	1085	7.39E-02	547	8.06E-01	966	3.26E-01	941	9.85E-01	1151	7.32E-01	960	1.01E-03	365	1	4.49E-01	770	2.89E-03	262	7.00E-01	830	5.57E-07	162	1.13E-01	850	6.85E-01	962	3.71E-02	264	3	4	713.38
2801	NM_000175	GPI	8.59E-01	1026	1.78E-02	239	4.69E-01	577	3.70E-01	815	6.08E-01	756	5.56E-01	1038	2.75E-01	688	5.82E-01	844	1.35E-06	118	2	6.58E-01	909	7.35E-01	1087	6.66E-01	793	2.92E-06	270	4.26E-01	996	8.19E-01	1038	2.84E-02	222	2	4	713.50
9873	NM_014824	fchsd2	2.40E-02	298	3.33E-01	728	5.18E-01	638	7.44E-01	1012	8.42E-01	995	4.95E-05	288	1.76E-01	544	5.28E-01	799	1.42E-02	562	3	3.32E-01	662	2.58E-01	828	2.19E-01	185	3.61E-01	1039	8.91E-02	824	8.11E-01	1036	7.74E-01	1010	0	3	715.50
5983	NM_181573	RfC4	9.80E-01	1131	3.70E-02	397	6.57E-02	180	2.69E-01	747	3.50E-01	465	5.69E-02	726	8.03E-02	382	6.80E-01	917	7.25E-11	14	2	2.17E-01	528	5.77E-01	1040	9.81E-01	1137	8.75E-01	1146	5.86E-01	1052	9.04E-01	1089	1.31E-01	498	0	2	715.56
4150	NM_015846	mbd1	7.45E-01	942	2.94E-02	341	2.94E-01	396	6.78E-01	979	6.85E-01	847	3.07E-02	663	2.58E-01	667	2.99E-01	617	7.70E-02	715	2	1.97E-01	512	4.34E-01	982	2.67E-01	241	9.52E-01	1156	3.91E-02	744	6.61E-01	947	3.11E-01	702	1	3	715.69
51362	NM_015891	Cdc40	9.98E-01	1155	7.88E-01	1019	6.80E-01	833	2.98E-01	765	9.11E-01	1060	6.13E-03	529	2.26E-01	622	7.65E-01	983	1.90E-01	820	1	3.07E-01	639	6.80E-02	591	5.20E-01	636	1.07E-05	351	1.07E-07	64	9.77E-01	1150	3.30E-02	245	3	4	716.38
8942	NM_001032998	Kynu	7.03E-02	498	8.34E-01	1047	4.00E-01	501	5.15E-01	895	6.83E-01	845	2.45E-01	898	2.45E-02	184	6.76E-01	911	2.87E-02	612	2	9.09E-01	1090	1.08E-03	190	9.19E-01	1054	2.49E-05	418	3.00E-01	951	2.95E-02	306	8.92E-01	1093	3	5	718.31
4664	NM_005967	NAB2	3.85E-03	98	5.17E-01	837	4.52E-01	551	9.57E-01	1137	7.72E-01	927	9.03E-01	1134	7.83E-01	1043	2.78E-02	194	2.53E-01	853	2	4.63E-01	782	1.04E-02	378	3.66E-01	404	1.13E-02	809	2.90E-01	946	8.00E-01	1032	7.27E-02	386	2	4	719.44
158	NM_000026	ADSL	7.93E-01	975	2.32E-01	654	2.59E-01	360	6.99E-01	994	8.87E-01	1038	1.75E-01	854	1.38E-01	483	1.17E-01	396	1.49E-08	55	1	9.29E-01	1107	2.34E-01	800	8.94E-01	1023	5.29E-08	58	6.65E-02	801	6.47E-01	936	7.26E-01	982	1	2	719.75
23598	NM_032051	PATZ1	9.63E-01	1118	5.71E-01	862	4.65E-01	569	5.24E-01	903	4.46E-01	579	3.90E-02	690	7.85E-02	377	6.43E-01	889	3.43E-01	906	1	3.53E-01	685	5.20E-01	1019	2.37E-02	14	9.56E-04	664	8.64E-02	821	3.74E-01	751	2.74E-01	675	2	3	720.13
8874	NM_145735	Arhgef7	4.39E-03	105	7.52E-01	993	5.22E-01	642	6.02E-02	533	2.98E-02	60	3.34E-01	944	2.98E-01	708	4.68E-01	758	3.40E-02	633	3	6.86E-01	927	1.12E-02	381	7.96E-01	935	2.70E-01	1011	4.49E-01	1004	4.32E-01	792	9.03E-01	1102	1	4	720.50
9126	NM_005445	smc3	4.21E-01	770	6.10E-01	888	2.26E-01	334	4.55E-02	494	6.63E-01	824	5.58E-01	1039	1.50E-01	499	8.58E-01	1040	1.72E-01	805	1	1.87E-03	59	2.92E-01	877	9.56E-01	1110	1.61E-04	547	5.69E-02	789	2.04E-01	599	5.35E-01	863	2	3	721.06
23279	NM_015231	NUP160	6.72E-01	895	1.42E-01	578	1.38E-01	238	7.71E-01	1031	4.78E-01	614	4.92E-01	1020	1.62E-01	521	4.46E-01	745	2.77E-04	293	1	3.04E-01	635	2.99E-01	882	2.97E-01	286	7.47E-02	940	6.19E-02	798	9.69E-01	1142	6.10E-01	919	0	1	721.06
1161	NM_001284	ap3s1	8.81E-01	1045	3.69E-02	396	6.78E-01	828	9.60E-01	1140	4.59E-01	590	1.94E-01	865	4.98E-01	879	9.93E-02	367	3.14E-02	621	2	8.58E-02	359	3.31E-02	509	5.04E-01	615	1.24E-01	963	1.10E-01	845	7.60E-02	437	8.72E-01	1083	1	3	721.38
55841	NM_018464	CISD1	8.42E-04	44	5.99E-01	880	7.28E-01	880	3.49E-01	802	8.62E-01	1016	6.99E-01	1082	1.72E-01	537	2.10E-01	534	2.08E-02	580	2	6.30E-01	897	9.18E-01	1143	6.77E-01	807	1.03E-03	670	3.51E-03	503	1.83E-02	257	6.24E-01	925	3	5	722.31
23589	NM_001042476	carhsp1	3.56E-02	377	6.73E-01	928	7.91E-01	945	1.22E-03	179	1.55E-01	220	4.84E-01	1017	3.48E-01	755	8.99E-01	1072	2.01E-01	828	2	3.51E-01	681	1.12E-02	383	4.06E-01	468	5.00E-01	1073	1.78E-01	897	6.23E-01	920	5.07E-01	841	1	3	724.00
10652	NM_006555	YKT6	4.75E-02	458	3.99E-01	765	3.77E-01	476	3.28E-02	444	8.46E-01	999	8.23E-03	547	5.15E-01	892	4.32E-02	251	1.97E-01	825	4	3.21E-01	649	3.09E-01	892	9.38E-01	1079	5.67E-01	1086	4.52E-02	766	5.08E-01	843	2.19E-01	623	1	5	724.69
3937	NM_005566	LdhA	3.22E-02	354	7.72E-01	1006	6.80E-01	834	9.37E-01	1126	5.95E-01	743	5.20E-02	719	2.20E-01	615	4.89E-05	9	3.02E-02	615	3	7.32E-01	963	9.49E-01	1150	9.30E-01	1068	6.63E-01	1112	8.33E-03	583	4.09E-06	11	2.93E-01	692	2	5	725.00
56926	NM_020177	FEM1C	7.03E-01	912	2.92E-02	335	9.64E-01	1131	7.64E-04	153	2.96E-01	403	6.90E-01	1078	6.92E-01	995	6.68E-01	905	2.64E-02	603	3	1.12E-01	399	2.68E-01	839	3.34E-01	348	3.47E-01	1033	7.52E-02	811	5.06E-01	840	4.70E-01	818	0	3	725.19
26003	NM_015530	gorasp2	4.33E-02	434	1.94E-01	627	7.25E-01	878	8.02E-03	311	8.54E-01	1010	1.62E-01	844	2.29E-01	624	3.24E-01	641	7.99E-03	507	3	9.36E-01	1113	3.15E-03	268	7.51E-01	877	8.65E-01	1144	2.92E-03	492	4.04E-01	772	8.52E-01	1065	2	5	725.44
9135	NM_001083585	RABEP1	9.42E-01	1092	2.24E-01	644	3.27E-01	430	6.83E-01	985	8.26E-01	985	1.87E-01	860	1.42E-02	127	4.67E-02	262	7.50E-01	1062	2	8.09E-01	1021	3.99E-01	966	9.03E-01	1031	6.63E-06	321	4.85E-04	373	4.19E-01	784	2.91E-01	690	2	4	727.06
2624	NM_001005742	GBA	7.64E-01	952	1.23E-02	193	5.58E-01	684	9.56E-01	1136	8.51E-01	1007	3.25E-01	940	4.24E-01	827	9.94E-01	1153	6.39E-01	1031	1	5.01E-01	810	1.93E-01	754	3.45E-01	365	1.04E-01	955	8.53E-10	19	2.03E-03	117	3.12E-01	703	2	3	727.88
8878	NM_003900	sqstm1	3.20E-01	712	8.03E-01	1025	7.37E-02	186	6.39E-01	964	7.46E-01	899	3.49E-01	952	2.35E-01	632	4.81E-01	767	3.18E-02	622	1	4.60E-01	778	4.42E-01	986	3.32E-01	347	7.20E-01	1122	5.91E-05	281	9.70E-01	1143	3.01E-02	230	2	3	727.88
9874	NM_001136554	Tlk1	2.85E-01	683	1.69E-01	605	6.42E-01	780	7.22E-01	1002	4.35E-01	565	1.53E-01	835	4.98E-01	878	1.61E-01	466	3.53E-01	913	0	9.92E-02	379	4.07E-01	972	1.17E-01	64	4.59E-01	1063	5.83E-01	1051	4.54E-01	810	1.87E-01	587	0	0	728.31
9968	NM_005120	MED12	3.83E-01	749	1.65E-01	601	7.85E-01	937	5.46E-02	521	8.58E-01	1014	8.01E-02	759	3.22E-01	731	7.49E-01	968	3.17E-01	893	0	5.31E-01	836	2.01E-03	231	1.62E-01	115	2.48E-01	1003	4.43E-02	761	1.66E-01	550	7.49E-01	996	2	2	729.06
4E+05	NM_001004060	NOMO2	4.07E-02	410	9.52E-01	1124	6.70E-01	819	2.03E-01	690	2.34E-01	319	3.56E-01	957	4.08E-01	818	1.88E-01	511	2.57E-01	854	1	8.79E-01	1077	3.21E-01	904	9.17E-01	1048	5.03E-05	472	6.87E-01	1075	4.01E-03	150	9.80E-02	442	2	3	729.38
11006	NM_006847	LILRB4	3.90E-02	402	8.74E-01	1077	7.56E-01	906	6.78E-01	980	1.48E-01	212	3.62E-02	679	6.09E-02	318	6.95E-01	929	7.31E-01	1054	2	2.99E-01	626	8.36E-03	357	9.78E-01	1135	1.62E-01	977	7.35E-01	1090	1.92E-08	1	6.36E-01	932	2	4	729.69
3146	NM_006895	HNMT	5.02E-01	820	4.59E-02	463	8.52E-01	1015	1.10E-01	600	5.91E-01	739	7.15E-01	1089	6.48E-02	332	8.37E-01	1028	3.25E-01	899	1	3.63E-01	695	9.33E-02	635	6.82E-01	810	3.11E-05	436	3.16E-05	240	9.40E-01	1114	4.06E-01	775	2	3	730.63
9260	NM_203352	Pdlim7	5.11E-01	822	1.17E-01	557	6.00E-01	740	2.33E-02	402	1.45E-01	208	2.04E-02	629	6.82E-01	989	8.15E-01	1015	4.97E-01	980	2	5.73E-01	864	1.89E-01	746	5.52E-01	672	6.54E-01	1108	4.22E-02	753	9.89E-02	475	3.49E-01	730	1	3	730.63
6602	NM_004595	Sms	2.92E-02	333	9.89E-01	1149	9.39E-01	1108	1.74E-05	43	9.85E-01	1135	2.74E-01	912	3.72E-01	785	9.20E-01	1090	6.40E-01	1032	2	2.40E-02	202	2.67E-02	480	8.28E-01	963	5.37E-01	1077	4.31E-04	368	1.11E-01	489	1.51E-01	526	3	5	730.75
7029	NM_001032281	TFPI	4.68E-01	797	7.43E-01	987	9.20E-01	1086	8.31E-01	1067	9.37E-01	1085	2.68E-04	361	2.08E-01	597	9.15E-01	1086	4.61E-01	966	1	2.17E-01	529	2.73E-01	846	3.29E-01	339	2.98E-02	873	3.28E-02	724	2.57E-02	294	5.94E-03	66	4	5	731.44
5876	NM_002871	Rabif	9.28E-01	1077	4.24E-01	779	9.04E-01	1074	7.51E-01	1017	2.25E-01	305	1.66E-09	56	1.78E-01	548	9.80E-01	1141	5.51E-03	481	2	9.34E-01	1112	1.47E-02	407	4.23E-01	494	9.97E-03	797	9.66E-01	1147	8.34E-01	1043	2.91E-02	226	3	5	731.50
79039	NM_024096	DCTPP1	7.54E-01	946	2.38E-02	283	1.86E-01	287	5.59E-03	277	6.31E-01	791	5.21E-01	1028	9.20E-02	403	3.08E-01	628	9.32E-02	735	2	6.18E-01	890	4.38E-01	983	5.86E-01	710	4.10E-01	1051	1.23E-01	861	7.24E-01	982	5.54E-01	874	0	2	733.06
54877	NM_001161584	Tex10	7.75E-01	962	7.59E-03	139	5.32E-01	655	3.50E-02	452	3.61E-02	72	4.20E-02	698	2.26E-01	621	3.48E-01	666	4.19E-01	943	4	8.86E-01	1080	8.05E-02	614	9.70E-01	1130	3.45E-02	890	9.98E-02	831	8.62E-01	1061	6.33E-01	930	1	5	734.00
23351	NM_015299	khnyn	1.21E-01	554	4.41E-01	792	6.78E-01	829	4.52E-01	856	3.91E-02	76	6.10E-02	731	6.56E-01	975	8.92E-01	1071	9.37E-01	1132	1	1.48E-01	445	9.24E-02	634	3.57E-01	390	5.13E-03	750	7.57E-01	1096	2.30E-01	620	4.53E-01	807	1	2	734.88
6397	NM_005065	SEL1L	4.57E-01	792	3.59E-01	741	3.06E-01	414	9.00E-01	1101	5.00E-01	641	7.95E-01	1107	1.00E-01	423	6.30E-02	299	5.63E-01	1000	0	2.15E-01	525	3.16E-01	901	4.94E-01	599	1.70E-01	978	3.03E-05	236	7.53E-01	1001	7.64E-01	1006	1	1	735.25
58155	NM_021198	Ctdsp1	2.53E-01	650	6.79E-01	935	4.09E-02	136	4.68E-02	499	4.60E-01	592	1.03E-01	794	1.32E-03	32	7.22E-01	953	1.77E-01	812	3	8.86E-01	1079	8.88E-01	1135	3.18E-01	320	5.71E-01	1088	4.35E-03	524	8.75E-01	1068	9.84E-01	1148	1	4	735.31
382	NM_001663	Arf6	9.39E-02	526	6.92E-01	948	8.79E-01	1050	4.89E-02	503	4.65E-01	596	1.03E-01	795	5.18E-01	898	4.11E-02	245	1.39E-02	559	3	8.25E-02	355	7.95E-01	1103	6.39E-01	770	3.10E-02	878	9.93E-01	1157	3.63E-01	739	2.81E-01	683	1	4	737.81
9019	NM_024569	MPZL1	5.65E-01	840	7.73E-01	1007	4.10E-01	507	1.26E-01	620	5.85E-01	729	2.62E-06	192	1.35E-01	477	2.11E-02	165	2.10E-03	408	3	4.54E-01	776	9.38E-01	1146	8.28E-01	962	5.49E-01	1082	9.47E-01	1144	4.90E-01	832	6.16E-01	920	0	3	737.94
1452	NM_001894	CSNK1E	2.19E-02	280	6.40E-01	909	5.35E-01	659	2.74E-03	222	8.84E-01	1034	7.81E-01	1104	1.72E-02	144	5.03E-01	781	2.24E-01	842	3	3.03E-01	633	6.48E-01	1065	2.72E-01	249	8.94E-02	949	7.81E-01	1102	8.84E-01	1077	4.29E-01	790	0	3	740.00
27252	NM_014458	KLHL20	2.81E-01	675	1.87E-02	248	5.82E-02	172	6.67E-01	975	5.76E-01	721	2.65E-02	650	6.58E-01	978	3.26E-01	642	2.55E-03	420	3	2.48E-01	568	1.93E-01	752	9.23E-01	1060	2.76E-02	866	9.91E-01	1155	5.78E-01	891	8.72E-01	1082	1	4	740.94
22820	NM_016128	CopG	3.51E-02	373	3.60E-02	389	8.17E-01	979	2.40E-01	724	6.50E-01	809	2.77E-02	652	2.04E-01	592	4.81E-01	768	1.20E-01	765	3	9.28E-01	1106	5.82E-01	1044	5.22E-01	640	1.68E-02	832	4.31E-01	999	1.43E-02	222	6.94E-01	966	2	5	741.25
6727	NM_003135	SRP19	6.06E-01	861	4.12E-02	430	7.94E-01	948	4.70E-01	867	4.21E-01	547	4.28E-06	199	4.00E-01	808	1.24E-01	406	7.78E-01	1072	2	9.98E-01	1155	2.74E-02	484	8.71E-01	999	7.13E-01	1120	1.98E-04	335	1.98E-01	591	8.30E-01	1041	2	4	741.44
9728	NM_014701	secisbp2	9.22E-01	1070	6.50E-01	916	3.12E-01	421	6.52E-01	969	4.46E-01	578	1.62E-02	612	6.83E-01	991	9.72E-01	1132	1.42E-01	780	1	2.17E-02	188	4.12E-02	537	9.65E-01	1119	2.76E-04	587	2.91E-03	491	5.71E-01	883	1.99E-01	599	4	5	742.06
8487	NM_001009183	sip1	5.66E-01	842	4.63E-02	468	8.15E-01	976	9.80E-01	1152	1.80E-01	246	5.52E-04	392	9.20E-01	1117	5.52E-01	824	4.45E-01	957	2	5.81E-01	869	1.32E-05	25	4.46E-01	537	3.91E-03	739	1.72E-02	651	9.27E-01	1104	7.38E-01	994	3	5	743.31
65258	NM_023077	C1orf163	6.89E-02	497	3.93E-01	761	9.34E-01	1102	7.59E-01	1025	7.69E-01	922	5.47E-01	1033	7.64E-02	366	1.31E-01	419	5.95E-01	1017	0	2.61E-01	578	7.96E-03	351	2.01E-01	158	6.50E-01	1106	4.48E-02	764	5.48E-01	872	6.16E-01	922	2	2	743.31
54953	NM_001142317	Txnl4b	7.07E-01	914	1.04E-02	169	9.78E-01	1145	4.88E-01	876	8.01E-01	962	6.98E-01	1081	7.76E-01	1040	8.21E-02	337	1.01E-03	366	2	7.15E-02	329	6.71E-02	589	7.08E-01	837	2.60E-02	861	8.30E-01	1110	7.73E-01	1011	4.12E-02	278	2	4	744.06
10640	NM_006544	exoc5	5.80E-03	130	5.50E-01	851	8.40E-01	1005	2.61E-01	741	9.69E-01	1118	5.20E-03	519	2.82E-02	193	3.82E-01	698	1.52E-05	181	4	7.22E-01	955	1.35E-01	693	8.68E-01	995	2.34E-01	999	4.92E-01	1023	4.10E-01	775	8.27E-01	1038	0	4	744.63
94081	NM_058191	GCFC1	7.42E-03	147	7.01E-01	954	9.30E-01	1098	2.03E-02	385	8.73E-01	1024	1.06E-04	323	8.79E-01	1089	2.52E-01	578	9.72E-01	1148	3	2.62E-01	580	1.33E-03	206	9.65E-01	1118	7.57E-03	777	2.02E-01	908	6.70E-01	952	2.62E-01	659	2	5	746.63
963	NM_001040033	Cd53	4.10E-02	414	6.31E-01	900	5.73E-01	703	1.65E-01	667	9.17E-01	1064	2.62E-02	648	1.60E-01	518	9.13E-01	1085	9.34E-01	1130	2	3.92E-01	723	2.94E-02	494	9.42E-01	1086	4.90E-01	1069	7.23E-02	807	2.23E-04	62	1.80E-01	577	2	4	746.69
56889	NM_020139	bdh2	1.80E-01	594	4.47E-01	795	7.80E-01	931	1.27E-01	621	8.28E-01	986	4.83E-08	105	7.39E-01	1016	5.80E-01	842	9.56E-02	739	1	2.40E-01	555	3.95E-01	962	8.67E-01	994	6.64E-03	769	7.86E-04	412	1.23E-01	508	9.70E-01	1141	2	3	748.13
22926	NM_007348	Atf6	3.06E-02	343	4.15E-01	775	4.54E-01	554	6.79E-01	982	9.89E-01	1144	1.45E-02	601	4.40E-01	839	9.04E-01	1076	2.81E-01	870	2	5.44E-01	845	2.92E-01	876	9.24E-01	1061	5.10E-04	633	8.31E-03	582	1.58E-01	541	3.55E-02	254	3	5	748.50
10014	NM_001015053	HDAC5	4.14E-02	419	7.24E-01	971	7.59E-01	908	6.35E-01	962	4.70E-01	604	9.39E-03	556	9.77E-01	1150	3.31E-01	645	1.21E-01	766	2	3.47E-01	676	5.74E-01	1039	7.61E-01	892	1.00E-03	669	1.29E-02	621	4.11E-02	355	4.06E-01	776	3	5	750.56
1523	NM_001914	cyb5a	6.46E-01	880	3.11E-02	355	3.90E-01	490	6.77E-03	293	3.66E-01	480	3.79E-02	686	1.98E-01	581	1.06E-01	378	4.31E-02	663	4	9.47E-01	1120	3.33E-01	914	7.59E-01	890	2.28E-01	997	9.11E-01	1133	8.78E-01	1070	9.38E-01	1121	0	4	753.19
1230	NM_007310	COMT	2.30E-02	287	7.58E-01	996	7.63E-01	913	5.33E-01	907	6.24E-01	779	8.26E-02	767	1.44E-01	490	8.79E-01	1059	3.29E-02	627	2	7.50E-01	981	3.00E-02	497	9.71E-01	1132	6.56E-01	1111	4.89E-01	1021	5.90E-04	85	8.96E-02	423	2	4	754.69
80024	NM_025057	C14orf45	1.35E-01	566	9.82E-01	1146	1.57E-01	257	2.45E-01	728	4.13E-01	534	6.39E-02	736	6.05E-01	951	6.14E-01	869	6.73E-01	1038	0	4.61E-01	780	3.18E-06	10	7.56E-01	887	1.59E-01	976	2.46E-01	932	7.37E-01	991	2.79E-01	680	1	1	755.06
5099	NM_002592	pcnA	9.95E-01	1147	9.98E-01	1158	5.68E-01	693	6.70E-01	977	9.99E-01	1154	3.40E-03	500	7.14E-02	348	2.70E-01	592	2.00E-01	826	1	1.52E-02	155	2.13E-01	776	3.14E-01	314	4.13E-01	1053	6.75E-01	1068	3.81E-02	339	7.42E-01	995	2	3	755.94
23741	NM_014335	EID1	8.09E-01	993	7.70E-01	1002	7.46E-04	15	5.09E-02	510	8.89E-01	1040	5.18E-03	518	2.03E-01	588	8.83E-01	1063	3.05E-01	886	2	9.06E-01	1086	8.77E-01	1130	5.42E-01	659	1.07E-03	675	3.17E-10	15	7.55E-01	1002	6.10E-01	918	2	4	756.25
57192	NM_020466	LYRM2	4.37E-02	436	4.17E-01	776	5.71E-01	699	4.03E-04	121	6.02E-01	751	3.54E-01	955	6.42E-01	970	6.34E-01	880	9.27E-01	1126	2	1.89E-02	180	3.92E-01	960	9.14E-01	1041	5.68E-02	919	2.72E-02	704	4.11E-01	777	5.10E-01	844	2	4	758.69
5311	NM_006718	PLAGL1	1.35E-02	203	8.89E-01	1089	8.02E-01	959	2.07E-01	694	6.20E-01	776	1.85E-02	623	8.01E-01	1058	4.06E-02	242	2.43E-01	846	3	7.52E-01	982	7.01E-02	594	7.24E-01	852	6.00E-04	644	3.87E-03	513	9.57E-01	1131	6.57E-01	941	2	5	759.19
64764	NM_001135095	FNDC3B	2.67E-02	322	5.55E-02	508	5.06E-01	618	2.98E-01	764	7.97E-01	956	7.67E-01	1101	3.03E-01	713	9.66E-01	1126	4.76E-06	151	2	1.00E+00	1158	2.80E-01	852	3.53E-01	384	2.57E-01	1007	3.99E-01	979	3.47E-01	726	4.33E-01	794	0	2	759.94
3140	NM_002128	HMGB1	1.22E-01	556	7.71E-01	1004	2.99E-01	402	5.11E-01	888	8.46E-01	998	4.02E-02	692	3.54E-02	221	5.45E-01	816	4.83E-01	973	2	3.68E-01	701	7.82E-01	1098	6.30E-01	755	1.97E-02	845	3.68E-03	506	8.15E-01	1037	2.68E-01	670	2	4	760.13
81628	NM_030940	iscA1	9.67E-03	170	9.51E-01	1123	9.46E-01	1116	2.17E-01	707	4.66E-01	597	1.05E-02	568	9.52E-01	1132	6.71E-01	906	3.44E-01	907	2	2.42E-01	558	1.00E-01	643	8.45E-01	976	3.09E-02	877	1.60E-02	645	3.21E-01	703	1.77E-01	576	2	4	762.75
23450	NM_012426	sf3b3	5.74E-01	848	2.51E-01	669	9.73E-01	1138	1.22E-01	616	4.35E-01	566	8.87E-01	1129	4.24E-01	826	6.75E-01	909	5.96E-01	1018	0	5.13E-01	822	1.71E-01	723	7.57E-01	889	6.26E-04	648	1.67E-02	646	6.29E-02	410	6.00E-02	360	2	2	763.56

**800**	**NM_033139**	**CALD1**	7.73E-01	961	9.05E-01	1095	7.89E-01	942	6.10E-01	949	9.94E-02	164	3.01E-01	929	2.03E-01	589	6.96E-01	932	6.75E-02	704	**0**	9.57E-01	1127	1.04E-01	648	7.74E-01	909	3.57E-01	1035	1.03E-05	198	1.54E-02	237	4.79E-01	825	**2**	**2**	**765.25**
**10385**	**NM_181531**	**BTN2A2**	3.63E-01	738	9.90E-01	1150	4.66E-01	570	5.47E-01	915	7.65E-01	916	4.75E-02	707	6.42E-02	331	4.79E-01	764	8.14E-01	1084	**1**	5.29E-02	291	1.18E-01	667	8.90E-01	1019	7.88E-02	943	6.36E-01	1060	1.87E-02	260	5.00E-01	838	**1**	**2**	**765.81**
**27101**	**NM_014412**	**CACYBP**	9.63E-01	1117	9.45E-01	1119	9.65E-01	1132	4.89E-02	502	9.44E-01	1092	2.00E-02	627	1.09E-02	109	4.68E-01	757	3.81E-02	646	**4**	6.70E-01	916	5.22E-01	1020	5.59E-01	681	2.54E-01	1006	2.44E-03	479	3.15E-01	696	6.07E-02	361	**1**	**5**	**766.25**
**4771**	**NM_005384**	**NFIL3**	3.17E-02	350	8.58E-01	1066	2.02E-01	305	3.49E-01	803	2.12E-01	283	1.19E-01	807	4.59E-01	856	9.13E-01	1083	1.75E-01	808	**1**	1.35E-01	428	4.95E-01	1007	8.43E-01	974	3.78E-02	897	1.42E-01	876	7.35E-01	989	3.48E-01	729	**1**	**2**	**766.31**
**7073**	**NM_004614**	**tk2**	9.96E-01	1151	9.33E-01	1112	6.37E-01	774	8.82E-01	1089	9.99E-01	1156	4.13E-01	989	3.92E-01	802	8.51E-01	1033	7.46E-01	1060	**0**	2.65E-01	584	1.09E-01	657	3.28E-01	338	9.01E-04	661	3.44E-05	243	2.19E-02	272	5.39E-02	340	**3**	**3**	**766.31**
**11060**	**NM_007014**	**WWP2**	6.94E-01	905	6.15E-01	892	6.84E-01	840	3.25E-01	785	3.57E-01	473	8.21E-02	765	1.75E-01	543	3.20E-01	637	7.96E-02	719	**0**	1.75E-01	482	4.48E-01	990	4.11E-01	479	7.98E-01	1135	4.36E-01	1002	3.65E-01	742	5.79E-01	893	**0**	**0**	**767.63**
**1983**	**NM_030666**	**SERPINB1**	7.85E-01	971	9.81E-01	1144	7.11E-01	860	3.27E-01	787	8.54E-01	1009	1.38E-01	829	3.74E-01	786	6.80E-01	916	7.66E-02	714	**0**	4.76E-01	794	1.13E-02	384	8.98E-01	1027	9.27E-10	7	5.32E-02	783	3.42E-03	136	9.86E-01	1149	**3**	**3**	**768.50**
**317**	**NM_181869**	**Apaf1**	9.13E-01	1064	4.93E-02	491	1.47E-01	244	5.63E-02	524	6.10E-01	759	1.84E-01	859	4.43E-01	842	2.03E-02	160	4.12E-03	458	**3**	9.39E-01	1116	1.26E-01	681	9.39E-01	1082	4.49E-01	1059	7.41E-01	1092	5.33E-01	859	8.25E-01	1036	**0**	**3**	**770.38**
**11161**	**NM_007176**	**c14orf1**	9.88E-01	1143	9.94E-01	1154	5.14E-01	630	5.65E-01	924	4.04E-01	527	1.25E-01	815	2.17E-02	173	9.55E-01	1119	5.57E-03	482	**2**	9.72E-01	1138	9.01E-02	631	5.01E-01	613	2.66E-03	717	3.03E-01	955	6.22E-02	409	5.93E-01	903	**1**	**3**	**770.81**
**23600**	**NM_014324**	**AMACR**	5.71E-01	844	6.86E-01	943	3.27E-01	432	5.10E-01	887	7.80E-01	936	2.76E-01	916	5.94E-02	314	5.47E-01	818	6.38E-02	700	**0**	6.71E-01	917	4.18E-03	298	8.09E-01	949	6.41E-02	928	5.34E-01	1033	1.15E-01	497	6.73E-01	947	**1**	**1**	**772.44**
**1E+05**	**NM_138444**	**kctd12**	9.87E-01	1142	4.11E-02	428	6.08E-01	748	1.45E-03	193	5.34E-01	665	7.40E-01	1095	6.47E-01	972	8.81E-01	1061	8.90E-01	1111	**2**	1.90E-01	507	7.91E-01	1101	1.80E-01	137	1.07E-05	352	4.52E-01	1006	6.27E-01	923	7.80E-01	1016	**1**	**3**	**778.56**
**3092**	**NM_001531**	**MR1**	4.79E-02	459	3.75E-01	751	3.51E-01	457	3.54E-03	238	6.68E-01	828	3.73E-01	970	4.39E-01	838	7.73E-01	988	3.73E-02	643	**3**	3.64E-01	697	2.15E-01	778	5.34E-01	648	7.37E-01	1126	4.04E-01	983	9.43E-01	1118	7.02E-01	972	**0**	**3**	**780.88**
**1E+05**	**NM_152581**	**Mospd2**	1.77E-01	591	8.21E-01	1036	9.06E-02	200	4.41E-02	490	3.77E-01	496	4.29E-01	999	8.45E-01	1080	8.72E-01	1050	2.06E-04	279	**2**	9.13E-01	1095	7.89E-01	1099	7.89E-01	924	4.07E-02	901	5.24E-03	543	5.73E-01	885	4.80E-01	826	**2**	**4**	**780.88**
**94104**	**NM_024831**	**tgs1**	2.08E-01	613	3.38E-02	373	4.28E-01	529	2.13E-01	700	2.94E-01	400	9.84E-01	1152	9.74E-01	1149	8.55E-01	1038	1.68E-01	803	**1**	3.96E-01	730	8.72E-01	1128	4.24E-01	498	1.06E-01	956	7.21E-03	568	3.87E-01	762	8.97E-01	1097	**1**	**2**	**781.00**
**51643**	**NM_016056**	**Tmbim4**	6.58E-01	888	8.77E-01	1079	1.47E-02	70	6.87E-01	988	6.46E-01	805	5.42E-03	522	2.16E-01	611	7.32E-01	959	4.83E-01	972	**2**	4.74E-01	790	6.01E-01	1048	4.78E-01	579	1.30E-04	534	6.46E-04	395	9.48E-01	1125	9.70E-01	1142	**2**	**4**	**781.69**
**9641**	**NM_014002**	**IKBKE**	1.57E-01	579	8.29E-01	1039	8.01E-01	956	6.08E-01	947	9.03E-01	1054	5.33E-02	720	3.88E-01	799	1.57E-02	142	1.56E-01	788	**1**	6.28E-01	895	4.08E-01	974	3.95E-01	459	6.54E-01	1109	8.62E-02	820	5.84E-01	897	5.44E-02	342	**0**	**1**	**782.50**
**55069**	**NM_018049**	**PLEKHJ1**	1.83E-02	239	7.14E-01	963	6.90E-01	846	6.29E-01	958	6.46E-01	804	4.04E-01	985	2.22E-02	175	9.05E-01	1079	3.29E-03	440	**3**	1.33E-01	425	2.90E-01	870	8.90E-01	1018	3.35E-04	599	4.57E-01	1010	6.84E-01	961	9.94E-01	1154	**1**	**4**	**782.88**
**25840**	**NM_014033**	**mettl7a**	7.45E-01	943	8.43E-01	1056	3.78E-01	479	6.76E-01	978	3.93E-01	509	1.83E-01	857	5.61E-02	298	9.47E-01	1111	1.23E-01	769	**0**	2.63E-01	581	3.80E-01	951	9.77E-01	1134	9.56E-02	952	2.20E-01	922	4.76E-02	370	2.20E-01	625	**1**	**1**	**783.44**
**8943**	**NM_003938**	**Ap3d1**	3.28E-01	719	8.73E-01	1075	6.31E-01	768	1.39E-01	636	9.52E-01	1105	8.46E-01	1118	2.03E-01	586	2.68E-01	590	1.02E-02	530	**1**	1.68E-01	472	6.65E-02	585	9.59E-01	1114	2.97E-01	1017	3.83E-02	743	1.34E-01	519	7.35E-01	989	**1**	**2**	**785.38**
**23476**	**NM_014299**	**brd4**	3.45E-01	729	4.70E-01	811	7.44E-01	893	2.32E-02	400	1.09E-01	177	2.75E-01	913	2.61E-01	668	3.38E-01	653	9.10E-03	521	**2**	5.40E-01	841	1.80E-01	733	9.63E-01	1117	3.59E-01	1038	3.82E-01	974	9.98E-01	1158	7.08E-01	977	**0**	**2**	**787.69**
**1977**	**NM_183004**	**EIF5**	9.48E-01	1098	3.11E-01	708	4.99E-01	609	9.72E-02	585	1.77E-02	31	2.55E-04	359	9.90E-01	1152	3.95E-01	707	2.24E-04	284	**3**	7.23E-01	957	3.01E-01	885	9.18E-01	1051	6.43E-01	1104	5.74E-01	1046	6.47E-01	934	9.04E-01	1103	**0**	**3**	**788.31**
**54885**	**NM_017760**	**NCAPG2**	8.83E-01	1046	4.00E-02	421	3.44E-01	449	6.45E-01	966	8.34E-01	988	6.39E-02	735	4.50E-02	256	1.78E-01	492	1.74E-02	572	**3**	7.68E-01	997	7.23E-01	1084	9.28E-01	1067	1.93E-02	842	5.93E-02	793	4.61E-01	817	8.92E-01	1092	**1**	**4**	**788.56**
**1612**	**NM_001349**	**Dars**	6.58E-01	887	4.37E-01	788	6.79E-01	832	7.15E-01	998	4.33E-01	563	4.20E-02	697	3.72E-02	226	9.33E-01	1099	5.09E-01	983	**2**	3.03E-01	632	6.65E-02	584	3.39E-01	355	1.31E-02	817	5.76E-01	1047	7.83E-01	1019	9.96E-01	1157	**1**	**3**	**792.75**
**7372**	**NM_000373**	**UMPS**	9.34E-01	1085	8.55E-01	1064	6.08E-01	747	8.80E-01	1088	7.98E-01	957	2.76E-01	915	3.21E-01	727	8.16E-01	1016	9.26E-01	1124	**0**	1.17E-02	139	5.42E-02	565	4.25E-01	500	3.03E-08	39	1.23E-02	618	9.29E-01	1108	8.18E-01	1032	**3**	**3**	**795.25**
**79366**	**NM_024520**	**c2orf47**	7.89E-01	974	8.79E-01	1081	5.09E-01	624	8.57E-01	1078	6.96E-01	860	6.19E-01	1056	5.02E-02	272	3.74E-01	689	9.84E-01	1153	**0**	4.18E-01	746	3.65E-01	938	4.74E-01	576	6.76E-03	771	7.07E-01	1080	3.66E-01	743	7.72E-03	85	**2**	**2**	**795.38**
**10955**	**NM_198941**	**SERINC3**	6.56E-01	885	6.40E-01	906	8.99E-01	1070	3.98E-01	829	7.34E-02	134	2.67E-01	910	8.95E-01	1098	2.85E-01	604	9.19E-01	1120	**0**	2.44E-01	562	9.77E-02	641	5.50E-01	668	3.75E-01	1042	2.23E-01	924	9.52E-02	467	5.42E-01	867	**0**	**0**	**795.44**
**9031**	**NM_032408**	**baz1b**	4.07E-04	35	6.19E-01	897	9.86E-01	1149	1.30E-01	627	2.88E-01	394	8.44E-01	1117	2.61E-02	186	7.41E-01	962	9.81E-05	250	**3**	6.91E-01	930	2.56E-01	824	9.15E-01	1045	6.99E-01	1119	8.90E-01	1124	7.92E-01	1025	9.23E-01	1110	**0**	**3**	**799.63**
**6533**	**NM_003059**	**slc22a4**	1.89E-01	599	1.70E-01	606	3.77E-01	478	9.46E-01	1129	9.88E-01	1138	6.92E-02	744	3.50E-01	760	5.46E-01	817	6.76E-01	1042	**0**	2.31E-01	541	8.01E-02	613	4.33E-01	517	3.37E-01	1030	5.74E-01	1045	2.02E-01	593	9.76E-01	1145	**0**	**0**	**799.81**
**81545**	**NM_030799**	**yipf5**	9.52E-01	1103	1.72E-02	234	6.42E-01	781	3.16E-01	779	8.61E-01	1015	1.08E-01	800	8.96E-01	1099	1.85E-01	507	2.23E-02	587	**2**	7.69E-01	1000	7.48E-02	600	8.38E-01	967	6.15E-02	924	1.10E-01	844	6.20E-01	915	2.93E-01	693	**0**	**2**	**803.00**
**23276**	**NM_025010**	**KLHL18**	2.57E-03	80	1.17E-01	559	5.03E-01	614	3.92E-01	824	9.21E-01	1066	3.02E-04	367	7.74E-01	1038	7.73E-01	987	1.89E-06	122	**3**	7.92E-01	1010	7.23E-01	1085	4.53E-01	551	9.33E-01	1155	9.49E-01	1145	9.36E-01	1112	9.75E-01	1144	**0**	**3**	**803.69**
**4437**	**NM_020998**	**MST1**	8.67E-01	1031	8.30E-01	1042	7.67E-03	52	5.12E-03	267	9.31E-01	1081	3.42E-03	501	3.31E-02	217	6.98E-01	935	4.69E-01	968	**4**	8.24E-01	1034	2.85E-01	860	9.06E-01	1034	4.84E-01	1066	7.33E-01	1089	7.33E-01	986	4.66E-01	816	**0**	**4**	**811.19**
**427**	**NM_177924**	**ASAH1**	7.37E-01	934	7.13E-01	961	3.03E-01	410	7.64E-01	1026	7.12E-01	877	7.08E-03	537	7.34E-02	353	5.34E-01	806	1.35E-01	775	**1**	4.84E-01	803	1.01E-01	645	9.27E-01	1066	2.90E-01	1016	5.08E-01	1028	4.25E-01	786	7.50E-01	997	**0**	**1**	**813.75**
**405**	**NM_001668**	**ARNT**	2.74E-01	666	3.71E-01	749	5.32E-01	654	6.79E-01	983	9.66E-01	1113	5.07E-01	1022	1.97E-01	579	1.45E-01	443	6.30E-01	1029	**0**	7.12E-01	945	4.01E-01	969	4.80E-01	582	5.30E-03	751	5.00E-01	1025	7.38E-02	433	8.77E-01	1084	**1**	**1**	**814.19**
**2909**	**NM_001146156**	**GSK3B**	7.78E-02	508	7.85E-01	1015	3.84E-01	482	3.38E-01	793	4.68E-01	600	8.22E-02	766	2.56E-01	663	8.12E-01	1014	8.99E-01	1114	**0**	6.85E-01	924	1.72E-01	724	9.07E-01	1035	2.96E-03	720	6.76E-02	802	8.90E-01	1080	5.20E-01	854	**1**	**1**	**818.38**
**8359**	**NM_021968**	**HIST2H4A**	9.85E-01	1139	9.55E-01	1126	6.08E-01	750	7.86E-01	1041	9.89E-01	1145	3.88E-01	976	1.95E-01	576	9.95E-01	1155	6.12E-01	1021	**0**	9.70E-01	1137	1.78E-02	426	3.08E-01	307	1.32E-05	369	1.80E-04	333	9.65E-01	1138	1.22E-01	489	**3**	**3**	**820.50**
**23345**	**NM_133650**	**SYNE1**	4.53E-03	109	3.48E-01	736	4.80E-01	587	3.59E-01	809	5.87E-01	733	3.77E-04	381	5.32E-01	908	6.37E-01	884	3.46E-01	908	**2**	8.64E-01	1066	3.64E-01	937	9.86E-01	1143	7.17E-01	1121	7.45E-01	1095	4.53E-01	808	6.64E-01	942	**0**	**2**	**822.94**
**6430**	**NM_001031684**	**sfrs7**	3.09E-02	345	7.38E-01	979	6.83E-01	839	8.86E-01	1091	8.82E-01	1030	6.88E-01	1077	6.32E-01	963	6.10E-01	864	2.92E-01	880	**1**	8.52E-01	1056	2.31E-03	244	5.91E-01	712	4.21E-02	903	8.91E-02	825	8.23E-01	1039	5.35E-02	337	**2**	**3**	**824.00**
**22906**	**NM_014965**	**TRAK1**	7.65E-01	956	6.47E-01	913	8.60E-01	1028	4.02E-01	832	3.27E-01	442	1.08E-01	799	2.63E-01	670	3.99E-01	716	7.96E-01	1079	**0**	2.34E-01	543	7.62E-01	1094	2.55E-01	218	3.20E-02	882	3.35E-01	963	9.56E-01	1130	6.56E-01	939	**1**	**1**	**825.25**
**9516**	**NM_004862**	**LITAF**	3.43E-02	368	9.14E-01	1105	9.67E-01	1135	7.36E-01	1009	6.90E-01	854	5.66E-06	207	9.87E-02	417	3.80E-01	695	1.66E-01	800	**2**	7.66E-01	995	7.98E-02	612	8.71E-01	998	2.28E-01	996	6.33E-01	1059	6.12E-01	909	8.47E-01	1059	**0**	**2**	**826.13**
**4686**	**NM_006153**	**NCK1**	9.00E-01	1058	9.44E-01	1118	4.39E-01	539	6.40E-01	965	7.77E-01	930	9.59E-01	1145	2.78E-02	192	9.88E-01	1148	5.60E-01	999	**1**	1.82E-01	492	1.14E-01	660	3.87E-01	440	3.75E-02	896	8.54E-01	1118	9.68E-02	472	8.79E-01	1085	**1**	**2**	**828.56**
**942**	**NM_175862**	**CD86**	4.45E-04	36	9.63E-01	1133	5.97E-01	733	8.88E-01	1095	9.88E-01	1139	4.03E-01	983	3.48E-01	753	9.73E-01	1133	2.56E-02	600	**2**	7.42E-01	973	2.68E-02	481	9.30E-01	1069	5.42E-01	1078	7.16E-01	1082	1.56E-05	26	6.71E-01	945	**2**	**4**	**828.69**
**5519**	**NM_006244**	**PPP2R5B**	5.94E-01	856	6.35E-01	904	7.10E-01	859	9.02E-01	1103	8.50E-01	1006	2.08E-01	872	1.32E-02	120	2.68E-01	591	6.05E-01	1019	**1**	8.60E-01	1065	3.71E-01	945	8.37E-01	966	1.10E-01	959	8.01E-02	815	9.10E-01	1095	1.03E-02	109	**1**	**2**	**830.25**
**22861**	**NM_014922**	**Nlrp1**	9.66E-01	1122	6.13E-01	889	1.66E-02	72	7.84E-01	1038	6.63E-01	823	9.11E-05	317	3.79E-01	792	1.92E-01	518	8.03E-01	1080	**2**	8.18E-01	1031	8.62E-01	1124	6.58E-01	786	8.63E-01	1143	1.12E-01	848	2.02E-01	594	9.46E-01	1126	**0**	**2**	**831.44**
**7181**	**NM_001032287**	**nr2c1**	9.92E-01	1145	9.81E-01	1145	2.96E-01	397	7.56E-01	1023	3.98E-01	516	1.73E-02	617	1.99E-02	162	9.05E-01	1078	2.03E-01	832	**2**	4.72E-01	788	8.02E-01	1106	8.77E-01	1008	8.69E-01	1145	1.59E-02	644	2.51E-01	642	9.08E-01	1106	**1**	**3**	**834.63**
**403**	**NM_004311**	**Arl3**	8.88E-01	1049	5.47E-01	850	5.18E-01	637	4.90E-01	877	9.12E-01	1061	9.84E-01	1151	1.66E-01	529	7.54E-01	973	2.16E-01	838	**0**	3.27E-01	656	1.81E-01	735	6.38E-01	769	3.07E-02	875	9.30E-01	1142	6.89E-01	965	4.10E-02	276	**2**	**2**	**836.44**
**4670**	**NM_004539**	**Nars**	3.00E-01	703	4.51E-02	458	5.81E-01	713	1.96E-02	381	4.27E-01	556	3.52E-03	503	9.31E-01	1122	1.02E-01	371	3.96E-01	931	**3**	8.18E-01	1030	5.45E-01	1027	9.85E-01	1140	8.82E-01	1147	9.12E-01	1134	8.78E-01	1072	9.42E-01	1125	**0**	**3**	**838.31**
**9659**	**NM_022359**	**PDE4DIP**	7.64E-01	953	7.20E-01	969	4.76E-01	584	8.68E-01	1083	4.79E-01	616	2.66E-01	909	1.45E-01	492	9.53E-01	1117	9.58E-01	1142	**0**	9.56E-01	1126	3.50E-01	926	9.98E-01	1155	2.41E-01	1000	2.13E-03	463	1.35E-02	215	3.84E-01	762	**2**	**2**	**844.50**
**53918**	**NM_015946**	**PELO**	7.82E-01	966	8.49E-01	1060	4.40E-01	542	5.26E-01	905	7.03E-01	871	9.45E-02	781	1.38E-02	124	8.52E-01	1036	1.61E-01	794	**1**	8.74E-01	1073	1.21E-01	672	9.45E-01	1095	1.90E-01	986	3.63E-01	969	1.89E-01	580	9.00E-01	1099	**0**	**1**	**847.06**
**9477**	**NM_004275**	**MED20**	9.35E-01	1086	1.31E-01	569	2.31E-01	337	4.10E-01	836	6.70E-01	832	7.87E-01	1105	3.01E-01	710	2.52E-01	577	7.47E-01	1061	**0**	4.19E-01	747	3.09E-01	893	9.49E-01	1098	4.24E-02	904	3.44E-01	964	9.92E-01	1156	3.51E-01	733	**1**	**1**	**850.50**
**10935**	**NM_006793**	**prdx3**	8.04E-01	988	8.80E-01	1084	5.74E-01	706	7.98E-01	1050	3.77E-01	498	2.09E-01	874	1.68E-01	533	9.67E-01	1127	9.29E-01	1127	**0**	8.26E-01	1039	6.47E-01	1064	3.04E-01	298	4.38E-01	1056	1.45E-01	878	6.80E-01	960	5.15E-02	326	**0**	**0**	**850.50**
**4507**	**NM_000254**	**mtr**	9.69E-01	1127	9.09E-01	1102	7.85E-01	936	9.18E-01	1114	5.38E-01	670	1.07E-01	798	4.51E-01	848	8.53E-01	1037	2.84E-01	873	**0**	7.39E-01	967	3.04E-01	886	1.63E-01	117	4.89E-04	631	9.35E-01	1143	4.27E-01	788	2.65E-01	667	**1**	**1**	**856.50**
**57035**	**NM_033009**	**PCBP4**	7.02E-01	910	2.17E-01	641	2.49E-01	352	9.78E-01	1151	7.80E-01	934	6.79E-02	741	6.15E-02	319	3.55E-01	673	6.25E-01	1027	**0**	8.56E-01	1060	3.93E-01	961	9.25E-01	1063	6.81E-01	1115	6.21E-01	1058	6.06E-01	906	9.61E-01	1136	**0**	**0**	**877.94**
**3423**	**NM_005531**	**IFI16**	9.83E-01	1134	8.45E-01	1059	4.90E-01	602	7.65E-01	1027	7.99E-01	959	8.92E-02	777	3.36E-02	219	9.74E-01	1136	3.23E-01	897	**1**	8.11E-01	1024	1.39E-01	703	7.42E-01	867	3.57E-01	1034	5.41E-01	1035	7.44E-01	994	3.11E-01	700	**0**	**1**	**885.44**
**5616**	**NM_000311**	**PRNP**	8.68E-01	1032	3.01E-01	704	5.63E-01	690	8.96E-01	1098	6.19E-01	773	2.26E-01	883	1.94E-01	573	7.64E-01	981	5.46E-01	996	**0**	8.41E-01	1047	2.58E-01	827	6.20E-01	746	4.95E-01	1071	1.60E-01	889	7.67E-01	1007	8.34E-01	1046	**0**	**0**	**897.69**
**3077**	**NM_181054**	**HIF1A**	8.74E-01	1039	5.96E-01	877	9.76E-01	1142	7.88E-01	1044	9.72E-01	1119	9.73E-01	1148	4.35E-01	835	8.17E-01	1018	5.15E-01	984	**0**	4.21E-01	748	5.32E-02	561	3.55E-01	386	1.73E-04	556	5.45E-01	1036	4.20E-01	785	8.96E-01	1096	**1**	**1**	**898.38**
**861**	**NM_001122607**	**Runx1**	8.23E-02	515	7.46E-01	990	5.50E-01	676	2.12E-01	699	9.25E-01	1075	5.43E-01	1032	3.44E-01	748	8.87E-01	1065	5.86E-01	1012	**0**	7.36E-01	964	1.25E-01	678	9.54E-01	1107	4.10E-01	1052	1.45E-01	879	4.05E-01	773	9.52E-01	1129	**0**	**0**	**899.63**
**5586**	**NM_002755**	**MAP2K1**	9.69E-01	1128	8.72E-01	1074	3.71E-01	474	9.03E-01	1104	9.52E-01	1104	4.45E-02	703	4.31E-02	249	9.43E-01	1106	6.62E-01	1036	**2**	2.91E-01	617	8.62E-01	1125	9.21E-01	1056	2.63E-01	1009	8.46E-01	1114	3.11E-01	691	8.66E-01	1078	**0**	**2**	**916.75**
**2002**	**NM_001423**	**emp1**	9.46E-01	1096	8.24E-01	1038	5.80E-01	711	9.77E-01	1148	8.78E-01	1028	1.91E-01	863	3.37E-01	739	9.77E-01	1138	9.27E-01	1125	**0**	9.08E-01	1089	4.18E-01	977	9.39E-01	1084	7.23E-01	1123	8.38E-01	1113	7.94E-01	1027	8.48E-01	1060	**0**	**0**	**1022.44**
