# Supplementary material for: Meta-Analysis and Gene Set Analysis of Archived Microarrays Suggest Implication of the Spliceosome in Metastatic and Hypoxic Phenotypes
Source: PLoS One. 2014 Jan 31;9(1):e86699. doi: 10.1371/journal.pone.0086699 (PMC3908947; doi:10.1371/journal.pone.0086699)
Supplement: Table S2 — List of pathways highlighted in the over-representation analysis using DAVID. (PDF) [file pone.0086699.s002.pdf]

Pathways	Count	P-value
**Pathways in cancer**	34	0.128993029
**Spliceosome**	30	2.15E-07
**Cell Cycle**	25	6.33E-05
**MAPK signaling pathway**	23	0.534279185
**Purine metabolism**	20	0.045141202
**Lysosome**	17	0.029382066
**Ubiquitin mediated proteolysis**	16	0.155492378
**Wnt signaling pathway**	16	0.260251872
**Oocyte meiosis**	15	0.066272587
**Focal adhesion**	15	0.785133927
**Regulation of actin cytoskeleton**	15	0.858864907
**Glycolysis / Gluconeogenesis**	14	9.55E-04
**Antigen processing and presentation**	13	0.037957548
**Neurotrophin signaling pathway**	13	0.322854344
**Insulin signaling pathway**	13	0.436160431
**Cytokine-cytokine receptor interaction**	13	0.993685858
**Colorectal cancer**	12	0.08222436
**Apoptosis**	12	0.099742861
**Pyrimidine metabolism**	12	0.156356087
**Endocytosis**	12	0.90335901
**Renal cell carcinoma**	11	0.059176675
**Natural killer cell mediated cytotoxicity**	11	0.66712723
**Aminoacyl-tRNA biosynthesis**	10	0.005207646
**p53 signaling pathway**	10	0.103905313
**Small cell lung cancer**	10	0.253266783
**Toll-like receptor signaling pathway**	10	0.454576747
**Amyotrophic lateral sclerosis (ALS)**	9	0.066401651
**Axon guidance**	9	0.845945896
**Chemokine signaling pathway**	9	0.989661573
**Cysteine and methionine metabolism**	8	0.018729259
**Prion diseases**	8	0.02180719
**Pyruvate metabolism**	8	0.042473007
**RNA degradation**	8	0.186022611
**Adherens junction**	8	0.451539961
**Cardiac muscle contraction**	8	0.465288019
**TGF-beta signaling pathway**	8	0.583878161
**Fc gamma R-mediated phagocytosis**	8	0.677384723
**Pentose phosphate pathway**	7	0.013859841
**Proteasome**	7	0.186889242
**Glutathione metabolism**	7	0.226784813
**Pathogenic Escherichia coli infection**	7	0.328172135
**NOD-like receptor signaling pathway**	7	0.403873607
**Epithelial cell signaling in Helicobacter pylori infection**	7	0.493687656
**Phosphatidylinositol signaling system**	7	0.578577935
**VEGF signaling pathway**	7	0.59203133
**Systemic lupus erythematosus**	7	0.836973122
**Melanogenesis**	7	0.836973122
**Leukocyte transendothelial migration**	7	0.932455598
**Tight junction**	7	0.970367173
**Base excision repair**	6	0.156973199
**Sphingolipid metabolism**	6	0.214898306
**mTOR signaling pathway**	6	0.429131612
**Arginine and proline metabolism**	6	0.445799185
**Vibrio cholerae infection**	6	0.49497103
**Fc epsilon RI signaling pathway**	6	0.782126807
**Progesterone-mediated oocyte maturation**	6	0.848494797
**GnRH signaling pathway**	6	0.91604753
**Cell adhesion molecules (CAMs)**	6	0.987452597
**Jak-STAT signaling pathway**	6	0.996989747

**One carbon pool by folate**	5	0.037298433
**Thyroid cancer**	5	0.212933078
**Citrate cycle (TCA cycle)**	5	0.249363971
**Fructose and mannose metabolism**	5	0.306106137
**SNARE interactions in vesicular transport**	5	0.383325467
**Aldosterone-regulated sodium reabsorption**	5	0.440699836
**Drug metabolism**	5	0.478082153
**Glycerolipid metabolism**	5	0.514465272
**Cytosolic DNA-sensing pathway**	5	0.675442553
**PPAR signaling pathway**	5	0.832171722
**Gap junction**	5	0.943265429
**Dilated cardiomyopathy**	5	0.952327629
**Calcium signaling pathway**	5	0.999823815
**Homologous recombination**	4	0.409463912
**RNA polymerase**	4	0.409463912
**Amino sugar and nucleotide sugar metabolism**	4	0.715168571
**Intestinal immune network for IgA production**	4	0.781231369
**Inositol phosphate metabolism**	4	0.834143911
**Hedgehog signaling pathway**	4	0.852015312
**Acute myeloid leukemia**	4	0.868187298
**Adipocytokine signaling pathway**	4	0.923173192
**Glycerophospholipid metabolism**	4	0.927772202
**Viral myocarditis**	4	0.940096025
**ECM-receptor interaction**	4	0.974136255
**Hypertrophic cardiomyopathy (HCM)**	4	0.975796891
**Ribosome**	4	0.978819489
**Vascular smooth muscle contraction**	4	0.99625566
**Protein export**	3	0.136207808
**Folate biosynthesis**	3	0.228027952
**Glycosphingolipid biosynthesis**	3	0.353961691
**Other glycan degradation**	3	0.3845696
**Histidine metabolism**	3	0.703156443
**Alanine, aspartate and glutamate metabolism**	3	0.737776543
**Ether lipid metabolism**	3	0.79675566
**Primary immunodeficiency**	3	0.79675566
**Basal transcription factors**	3	0.79675566
**Starch and sucrose metabolism**	3	0.872206597
**Notch signaling pathway**	3	0.909305643
**Metabolism of xenobiotics by cytochrome P450**	3	0.964061078
**Long-term potentiation**	3	0.980053388
**Long-term depression**	3	0.981484305
**RIG-I-like receptor signaling pathway**	3	0.984053805
**Arrhythmogenic right ventricular cardiomyopathy (ARVC)**	3	0.989055096
