# Supplementary material for: Meta-Analysis and Gene Set Analysis of Archived Microarrays Suggest Implication of the Spliceosome in Metastatic and Hypoxic Phenotypes
Source: PLoS One. 2014 Jan 31;9(1):e86699. doi: 10.1371/journal.pone.0086699 (PMC3908947; doi:10.1371/journal.pone.0086699)
Supplement: Table S3 — Full list of p-values obtained in the geneset analysis. (PDF) [file pone.0086699.s003.pdf]

	Hypoxia							Metastasis											
**Geneset Name**	**E-MEXP-445**	**GSE4725**	**GSE11341**	**E-MEXP-1896**	**GSE4086**	**GSE5579**	**GSE9234**	**E-GEOD-1323**	**E-GEOD-2280**	**GSE7929**	**GSE7930**	**GSE7956**	**GSE8401**	**GSE3325**	**GSE8977**	**GSE9576**	**5 % Hypoxia**	**5% Metastasis**	**% Total**
KEGG_PATHWAYS_IN_CANCER	0.002	0	0	0	0	0	0	0	0	0	0	0.029	0	0	0	0	7	9	100
KEGG_GLYCOLYSIS_GLUCONEOGENESIS	0.001	0	0	0.001	0	0	0	0	0	0	0	0.336	0	0	0	0.008	7	8	93.75
KEGG_PURINE_METABOLISM	0.046	0	0	0	0	0	0	0	0	0	0	0.127	0	0	0	0.002	7	8	93.75
KEGG_RIBOSOME	0	0	0	0	0.0005	0.005	0	0	0	0	0	0.256	0	0	0	0.013	7	8	93.75
KEGG_PPAR_SIGNALING_PATHWAY	0.004	0	0	0.0055	0	0	0	0	0	0	0	0.231	0	0	0.002	0.008	7	8	93.75
KEGG_MAPK_SIGNALING_PATHWAY	0.028	0	0	0	0	0.0055	0	0	0	0	0	0.097	0	0	0	0	7	8	93.75
KEGG_ERBB_SIGNALING_PATHWAY	0.009	0	0	0.001	0	0.042	0	0	0	0	0	0.276	0	0	0.001	0.009	7	8	93.75
KEGG_CYTOKINE_CYTOKINE_RECEPTOR_INTERACTION	0.022	0	0	0.0385	0	0.0005	0	0	0	0	0	0.217	0	0	0	0.002	7	8	93.75
KEGG_CHEMOKINE_SIGNALING_PATHWAY	0	0	0	0.001	0	0	0	0	0	0	0	0.188	0	0	0	0.001	7	8	93.75
KEGG_ENDOCYTOSIS	0.001	0	0	0.0005	0	0.013	0	0	0	0	0	0.112	0	0	0	0	7	8	93.75
KEGG_APOPTOSIS	0.011	0	0	0	0	0.014	0	0	0	0	0	0.264	0	0	0.001	0.006	7	8	93.75
KEGG_VEGF_SIGNALING_PATHWAY	0.019	0	0	0	0	0.002	0	0	0	0	0	0.266	0	0	0.01	0.005	7	8	93.75
KEGG_FOCAL_ADHESION	0.026	0	0	0	0	0	0	0	0	0	0	0.055	0	0	0	0	7	8	93.75
KEGG_REGULATION_OF_ACTIN_CYTOSKELETON	0.043	0	0	0	0	0	0	0	0	0	0	0.072	0	0	0	0	7	8	93.75
KEGG_ADIPOCYTOKINE_SIGNALING_PATHWAY	0.04	0	0	0.0005	0	0.0235	0	0.003	0	0	0	0.384	0	0	0.002	0.038	7	8	93.75
KEGG_HUNTINGTONS_DISEASE	0.001	0	0	0	0	0	0	0	0	0	0	0.063	0	0	0	0	7	8	93.75
KEGG_PANCREATIC_CANCER	0.008	0	0	0	0	0.0015	0	0	0	0	0	0.284	0	0	0.001	0.007	7	8	93.75
KEGG_CHRONIC_MYELOID_LEUKEMIA	0.036	0	0	0	0	0.0085	0	0	0	0	0	0.243	0	0	0.001	0.016	7	8	93.75
KEGG_CITRATE_CYCLE_TCA_CYCLE	0.036	0	0	0.0005	0	0.213	0	0	0	0	0	0.447	0	0	0.048	0.033	6	8	87.5
KEGG_FRUCTOSE_AND_MANNOSE_METABOLISM	0.003	0	0	0.027	0	0.0155	0	0.003	0	0	0	0.405	0	0	0.055	0.034	7	7	87.5
KEGG_OXIDATIVE_PHOSPHORYLATION	0.019	0	0	0	0	0.1485	0	0	0	0	0	0.119	0	0	0.001	0.001	6	8	87.5
KEGG_VALINE_LEUCINE_AND_ISOLEUCINE_DEGRADATION	0.026	0	0	0	0	0.685	0	0	0	0	0	0.304	0	0	0.038	0.034	6	8	87.5
KEGG_GLUTATHIONE_METABOLISM	0.04	0	0	0.015	0	0.2755	0	0	0	0	0	0.317	0	0	0.007	0.038	6	8	87.5
KEGG_AMINO_SUGAR_AND_NUCLEOTIDE_SUGAR_METABOLISM	0.013	0	0	0.0025	0.0025	0.0235	0	0	0	0	0	0.25	0	0.001	0.039	0.063	7	7	87.5
KEGG_PYRUVATE_METABOLISM	0.013	0	0	0.0115	0	0.228	0	0	0	0	0	0.443	0	0	0.011	0.049	6	8	87.5
KEGG_RNA_DEGRADATION	0.098	0	0	0	0	0.0205	0	0	0	0	0	0.361	0	0	0.012	0.018	6	8	87.5
KEGG_SPLICEOSOME	0.015	0	0	0	0	0.9215	0	0	0	0	0	0.223	0	0	0	0.001	6	8	87.5
KEGG_CALCIUM_SIGNALING_PATHWAY	0.751	0	0	0.004	0	0	0	0	0	0	0	0.225	0	0	0	0.003	6	8	87.5
KEGG_NEUROACTIVE_LIGAND_RECEPTOR_INTERACTION	0.908	0	0	0.0125	0	0	0	0	0	0	0	0.425	0	0	0	0.01	6	8	87.5
KEGG_CELL_CYCLE	0.173	0	0	0	0	0.0065	0	0	0	0	0	0.181	0	0	0	0.022	6	8	87.5
KEGG_OOCYTE_MEIOSIS	0.428	0	0	0	0	0.0445	0	0	0	0	0	0.205	0	0	0.001	0.011	6	8	87.5
KEGG_P53_SIGNALING_PATHWAY	0.137	0	0	0	0	0.0225	0	0	0	0	0	0.215	0	0	0.002	0.022	6	8	87.5
KEGG_UBIQUITIN_MEDIATED_PROTEOLYSIS	0.027	0	0	0	0	0.195	0	0	0	0	0	0.112	0	0	0	0.002	6	8	87.5
KEGG_LYSOSOME	0	0	0	0	0	0.342	0	0	0	0	0	0.088	0	0	0	0	6	8	87.5
KEGG_MTOR_SIGNALING_PATHWAY	0.056	0	0	0.003	0.0005	0.0095	0	0	0	0	0	0.409	0	0	0.009	0.036	6	8	87.5
KEGG_VASCULAR_SMOOTH_MUSCLE_CONTRACTION	0.4	0	0	0.005	0	0	0	0	0	0	0	0.196	0	0	0	0.002	6	8	87.5
KEGG_WNT_SIGNALING_PATHWAY	0.048	0	0	0	0	0.1315	0	0	0	0	0	0.189	0	0	0	0.003	6	8	87.5
KEGG_AXON_GUIDANCE	0.489	0	0	0.0035	0	0.0085	0	0	0	0	0	0.123	0	0	0	0.009	6	8	87.5
KEGG_ECM_RECEPTOR_INTERACTION	0.213	0	0	0.0045	0	0.0025	0	0	0	0	0	0.181	0	0	0	0.033	6	8	87.5
KEGG_ADHERENS_JUNCTION	0.087	0	0	0	0	0.0035	0	0	0	0	0	0.208	0	0	0	0.004	6	8	87.5
KEGG_TIGHT_JUNCTION	0.151	0	0	0	0	0.0165	0	0	0	0	0	0.217	0	0	0	0.003	6	8	87.5
KEGG_GAP_JUNCTION	0.294	0	0	0	0	0.0165	0	0	0	0	0	0.137	0	0	0	0.016	6	8	87.5
KEGG_ANTIGEN_PROCESSING_AND_PRESENTATION	0.046	0	0	0.0015	0	0.726	0	0	0	0	0	0.301	0	0	0	0.001	6	8	87.5
KEGG_TOLL_LIKE_RECEPTOR_SIGNALING_PATHWAY	0	0	0	0.002	0	0.2435	0	0	0	0	0	0.275	0	0	0.001	0.014	6	8	87.5
KEGG_RIG_I_LIKE_RECEPTOR_SIGNALING_PATHWAY	0.011	0	0	0.0005	0	0.23	0	0	0	0	0	0.357	0	0	0.007	0.037	6	8	87.5
KEGG_JAK_STAT_SIGNALING_PATHWAY	0.295	0	0	0	0	0.0165	0	0	0	0	0	0.255	0	0	0	0.006	6	8	87.5
KEGG_NATURAL_KILLER_CELL_MEDIATED_CYTOTOXICITY	0	0	0	0.0015	0	0.255	0	0	0	0	0	0.235	0	0	0	0.003	6	8	87.5
KEGG_T_CELL_RECEPTOR_SIGNALING_PATHWAY	0.011	0	0	0	0	0.08	0	0.001	0	0	0	0.289	0	0	0.001	0.004	6	8	87.5
KEGG_B_CELL_RECEPTOR_SIGNALING_PATHWAY	0.005	0	0	0	0	0.356	0	0	0	0	0	0.357	0	0	0.001	0.013	6	8	87.5
KEGG_FC_EPSILON_RI_SIGNALING_PATHWAY	0.009	0	0	0.0405	0	0.3415	0	0	0	0	0	0.342	0	0	0.001	0.008	6	8	87.5
KEGG_FC_GAMMA_R_MEDIATED_PHAGOCYTOSIS	0	0	0	0	0	0.1215	0	0	0	0	0	0.28	0	0	0	0.002	6	8	87.5
KEGG_LEUKOCYTE_TRANSENDOTHELIAL_MIGRATION	0.12	0	0	0.0375	0	0.002	0	0	0	0	0	0.189	0	0	0	0.002	6	8	87.5
KEGG_NEUROTROPHIN_SIGNALING_PATHWAY	0	0	0	0	0	0.51	0	0	0	0	0	0.184	0	0	0	0.002	6	8	87.5
KEGG_LONG_TERM_DEPRESSION	0.393	0	0	0.006	0	0.045	0	0	0	0	0	0.293	0	0	0.002	0.021	6	8	87.5
KEGG_ALZHEIMERS_DISEASE	0.023	0	0	0	0	0.06	0	0	0	0	0	0.058	0	0	0	0	6	8	87.5
KEGG_PARKINSONS_DISEASE	0.027	0	0	0	0	0.1125	0	0	0	0	0	0.128	0	0	0.001	0	6	8	87.5

KEGG_VIBRIO_CHOLERAE_INFECTION	0.018	0	0	0	0	0.151	0	0	0	0	0	0.312	0	0	0.012	0.031	6	8	87.5
KEGG_EPITHELIAL_CELL_SIGNALING_IN_HELICOBACTER_PYLORI_INFECTION	0.003	0	0	0.0015	0	0.099	0	0	0	0	0	0.235	0	0	0.001	0.029	6	8	87.5
KEGG_LEISHMANIA_INFECTION	0.016	0	0	0.0045	0	0.331	0	0	0	0	0	0.315	0	0	0	0.025	6	8	87.5
KEGG_COLORECTAL_CANCER	0.016	0	0	0.0005	0	0.323	0	0	0	0	0	0.311	0	0	0.003	0.012	6	8	87.5
KEGG_RENAL_CELL_CARCINOMA	0	0	0	0	0	0.0015	0	0	0	0	0	0.248	0	0	0.001	0.028	7	8	93.75
KEGG_PROSTATE_CANCER	0.135	0	0	0	0	0.0105	0	0	0	0	0	0.239	0	0	0	0.002	6	8	87.5
KEGG_MELANOMA	0.464	0	0	0.003	0	0.007	0	0	0	0	0	0.374	0	0	0	0.015	6	8	87.5
KEGG_BLADDER_CANCER	0.045	0	0	0.0195	0.003	0	0	0	0	0	0	0.37	0	0	0.028	0.055	7	7	87.5
KEGG_ACUTE_MYELOID_LEUKEMIA	0.009	0	0	0	0	0.626	0	0	0	0	0	0.342	0	0	0	0.018	6	8	87.5
KEGG_SMALL_CELL_LUNG_CANCER	0.346	0	0	0	0	0	0	0	0	0	0	0.131	0	0	0	0.029	6	8	87.5
KEGG_VIRAL_MYOCARDITIS	0.025	0	0	0.0205	0	0.432	0	0	0	0	0	0.309	0	0	0	0.018	6	8	87.5
KEGG_FATTY_ACID_METABOLISM	0.246	0	0	0.0035	0	0.846	0	0	0	0	0	0.391	0	0	0.028	0.018	5	8	81.25
KEGG_PYRIMIDINE_METABOLISM	0.18	0	0	0	0	0.6305	0	0	0	0	0	0.253	0	0	0.002	0.009	5	8	81.25
KEGG_ALANINE_ASPARTATE_AND_GLUTAMATE_METABOLISM	0.614	0	0	0.074	0.001	0.009	0	0	0	0	0	0.38	0	0.003	0.041	0.04	5	8	81.25
KEGG_LYSINE_DEGRADATION	0.097	0	0	0.0045	0	0.258	0	0	0	0	0	0.463	0	0	0.009	0.045	5	8	81.25
KEGG_TRYPTOPHAN_METABOLISM	0.27	0	0	0.1825	0	0.049	0	0.001	0	0	0	0.434	0	0	0.009	0.04	5	8	81.25
KEGG_GLYCEROLIPID_METABOLISM	0.313	0	0	0.006	0	0.4985	0	0.004	0	0	0	0.42	0	0	0.009	0.009	5	8	81.25
KEGG_INOSITOL_PHOSPHATE_METABOLISM	0.035	0	0	0.0075	0	0.299	0	0	0	0	0	0.365	0	0	0.036	0.091	6	7	81.25
KEGG_GLYCEROPHOSPHOLIPID_METABOLISM	0.49	0	0	0.0005	0	0.263	0	0.002	0	0	0	0.396	0	0	0.005	0.012	5	8	81.25
KEGG_BUTANOATE_METABOLISM	0.557	0	0	0.021	0	0.463	0	0	0	0	0	0.448	0	0	0.026	0.017	5	8	81.25
KEGG_METABOLISM_OF_XENOBIOTICS_BY_CYTOCHROME_P450	0.416	0	0	0.2785	0	0	0	0.001	0	0	0	0.48	0	0	0.001	0.022	5	8	81.25
KEGG_DRUG_METABOLISM_OTHER_ENZYMES	0.329	0	0	0.028	0	0.5895	0	0.001	0	0	0	0.534	0	0	0.031	0.023	5	8	81.25
KEGG_BASAL_TRANSCRIPTION_FACTORS	0.156	0	0	0.0015	0	0.3815	0	0	0	0	0.001	0.46	0	0.002	0.043	0.03	5	8	81.25
KEGG_PHOSPHATIDYLINOSITOL_SIGNALING_SYSTEM	0.168	0	0	0.0105	0	0.217	0	0	0	0	0	0.296	0	0	0.008	0.02	5	8	81.25
KEGG_PEROXISOME	0.199	0	0	0	0	0.1435	0	0	0	0	0	0.281	0	0	0.009	0.004	5	8	81.25
KEGG_NOTCH_SIGNALING_PATHWAY	0.166	0	0	0.0085	0	0.049	0	0	0	0	0	0.411	0	0	0.013	0.095	6	7	81.25
KEGG_HEDGEHOG_SIGNALING_PATHWAY	0.524	0	0	0.0065	0	0.0295	0	0	0	0	0	0.542	0	0	0.019	0.106	6	7	81.25
KEGG_TGF_BETA_SIGNALING_PATHWAY	0.188	0	0	0.001	0	0.2005	0	0	0	0	0	0.25	0	0	0.001	0.021	5	8	81.25
KEGG_CELL_ADHESION_MOLECULES_CAMS	0.104	0	0	0.0015	0	0.111	0	0	0	0	0	0.258	0	0	0	0	5	8	81.25
KEGG_NOD_LIKE_RECEPTOR_SIGNALING_PATHWAY	0	0	0	0.0035	0	0.12	0	0	0	0	0	0.374	0	0	0.001	0.068	6	7	81.25
KEGG_CYTOSOLIC_DNA_SENSING_PATHWAY	0.028	0	0	0.0035	0	0.495	0	0.001	0	0	0	0.397	0	0	0.001	0.074	6	7	81.25
KEGG_HEMATOPOIETIC_CELL_LINEAGE	0.592	0	0	0.043	0	0.1335	0	0	0	0	0	0.441	0	0	0	0.014	5	8	81.25
KEGG_LONG_TERM_POTENTIATION	0.59	0	0	0.005	0	0.601	0	0	0	0	0	0.254	0	0	0.01	0.017	5	8	81.25
KEGG_INSULIN_SIGNALING_PATHWAY	0.055	0	0	0	0	0.0645	0	0	0	0	0	0.177	0	0	0	0	5	8	81.25
KEGG_GNRH_SIGNALING_PATHWAY	0.142	0	0	0.003	0	0.304	0	0	0	0	0	0.256	0	0	0.001	0.005	5	8	81.25
KEGG_PROGESTERONE_MEDIATED_OOCYTE_MATURATION	0.208	0	0	0	0	0.2625	0	0	0	0	0	0.299	0	0	0.002	0.018	5	8	81.25
KEGG_MELANOGENESIS	0.164	0	0	0	0	0.218	0	0	0	0	0	0.271	0	0	0	0.009	5	8	81.25
KEGG_VASOPRESSIN_REGULATED_WATER_REABSORPTION	0.138	0	0	0.0005	0	0.3035	0	0	0	0	0	0.462	0	0	0.026	0.032	5	8	81.25
KEGG_AMYOTROPHIC_LATERAL_SCLEROSIS_ALS	0.208	0	0	0.006	0	0.0545	0	0	0	0	0	0.401	0	0	0.006	0.007	5	8	81.25
KEGG_PATHOGENIC_ESCHERICHIA_COLI_INFECTION	0.012	0	0	0	0	0.473	0	0	0	0	0	0.2	0	0	0.003	0.064	6	7	81.25
KEGG_ENDOMETRIAL_CANCER	0.062	0	0	0	0	0.326	0	0	0	0	0	0.393	0	0	0.008	0.013	5	8	81.25
KEGG_GLIOMA	0.115	0	0	0.0015	0	0.1795	0	0	0	0	0	0.396	0	0	0.003	0.01	5	8	81.25
KEGG_THYROID_CANCER	0.03	0	0	0	0.0045	0.1995	0	0	0	0	0	0.47	0	0	0.01	0.076	6	7	81.25
KEGG_BASAL_CELL_CARCINOMA	0.231	0	0	0.0035	0	0.021	0	0	0	0	0	0.495	0	0.001	0.006	0.11	6	7	81.25
KEGG_NON_SMALL_CELL_LUNG_CANCER	0.071	0	0	0	0	0.275	0	0	0	0	0	0.362	0	0	0.008	0.026	5	8	81.25
KEGG_SYSTEMIC_LUPUS_ERYTHEMATOSUS	0.172	0	0	0.017	0	0.1865	0	0	0	0	0	0.441	0	0	0	0.029	5	8	81.25
KEGG_ARRHYTHMOGENIC_RIGHT_VENTRICULAR_CARDIOMYOPATHY_ARVC	0.561	0	0	0.001	0	0.1355	0	0	0	0	0	0.313	0	0	0.002	0.029	5	8	81.25
KEGG_DILATED_CARDIOMYOPATHY	0.621	0	0	0.004	0	0.135	0	0.011	0	0	0	0.322	0	0	0.001	0.046	5	8	81.25
KEGG_PENTOSE_PHOSPHATE_PATHWAY	0.007	0	0	0.0335	0.0025	0.057	0	0	0	0	0	0.465	0	0.001	0.101	0.111	6	6	75
KEGG_GALACTOSE_METABOLISM	0.035	0	0	0.0205	0.0295	0.5615	0	0.01	0	0	0	0.498	0	0.004	0.104	0.077	6	6	75
KEGG_STEROID_BIOSYNTHESIS	0.068	0	0	0.015	0	0.026	0	0	0	0	0	0.657	0	0.034	0.154	0.303	6	6	75
KEGG_STEROID_HORMONE_BIOSYNTHESIS	0.592	0	0	0.328	0.003	0	0	0.028	0	0	0	0.656	0	0.002	0.046	0.05	5	7	75
KEGG_CYSTEINE_AND_METHIONINE_METABOLISM	0.053	0	0	0.0185	0	0.3965	0	0	0	0	0	0.501	0	0	0.014	0.059	5	7	75
KEGG_ARGININE_AND_PROLINE_METABOLISM	0.085	0	0	0.0705	0	0.861	0	0	0	0	0	0.302	0	0	0	0.012	4	8	75
KEGG_HISTIDINE_METABOLISM	0.24	0	0	0.0985	0	0.6995	0	0.002	0	0	0	0.594	0	0	0.021	0.014	4	8	75
KEGG_TYROSINE_METABOLISM	0.062	0	0	0.123	0.0005	0.5955	0	0.002	0	0	0	0.502	0	0.001	0.024	0.025	4	8	75
KEGG_SELENOAMINO_ACID_METABOLISM	0.152	0	0	0.0065	0.0005	0.031	0	0	0	0	0	0.547	0	0.004	0.097	0.071	6	6	75
KEGG_ARACHIDONIC_ACID_METABOLISM	0.681	0	0	0.2475	0	0.062	0	0.003	0	0	0	0.465	0	0	0.002	0.048	4	8	75
KEGG_SPHINGOLIPID_METABOLISM	0.008	0	0	0.012	0.0015	0.268	0	0.002	0	0	0	0.485	0	0.002	0.054	0.094	6	6	75
KEGG_RIBOFLAVIN_METABOLISM	0.058	0	0	0.047	0	0.0055	0	0	0	0	0.012	0.584	0	0.008	0.076	0.245	6	6	75
KEGG_RETINOL_METABOLISM	0.963	0	0	0.0615	0	0	0	0.007	0	0	0	0.613	0	0.002	0.017	0.074	5	7	75
KEGG_PORPHYRIN_AND_CHLOROPHYLL_METABOLISM	0.526	0	0	0	0	0	0	0.001	0	0	0.03	0.63	0	0.007	0.173	0.059	6	6	75
KEGG_NITROGEN_METABOLISM	0.119	0	0	0.014	0.0035	0.021	0	0.004	0	0	0	0.551	0	0.003	0.083	0.107	6	6	75
KEGG_AMINOACYL_TRNA_BIOSYNTHESIS	0.083	0	0	0.002	0	0.35	0	0	0	0	0	0.293	0	0	0.077	0.045	5	7	75
KEGG_DRUG_METABOLISM_CYTOCHROME_P450	0.505	0	0	0.155	0	0.2605	0	0.002	0	0	0	0.45	0	0.001	0.005	0.013	4	8	75
KEGG_BIOSYNTHESIS_OF_UNSATURATED_FATTY_ACIDS	0.039	0	0	0.0035	0.006	0.145	0	0.004	0	0	0	0.46	0	0.005	0.085	0.079	6	6	75

KEGG_RNA_POLYMERASE	0.521	0	0	0.002	0.001	0.5285	0	0	0	0	0.003	0.537	0	0.004	0.026	0.12	5	7	75
KEGG_DNA_REPLICATION	0.449	0	0	0	0	0.817	0	0	0	0	0	0.584	0	0	0.039	0.273	5	7	75
KEGG_BASE_EXCISION_REPAIR	0.324	0	0	0	0	0.7545	0	0	0	0	0.003	0.543	0	0	0.036	0.08	5	7	75
KEGG_NUCLEOTIDE_EXCISION_REPAIR	0.101	0	0	0	0	0.0135	0	0	0	0	0	0.429	0	0	0.058	0.113	6	6	75
KEGG_SNARE_INTERACTIONS_IN_VESICULAR_TRANSPORT	0.089	0	0	0.0005	0	0.314	0	0	0	0	0	0.383	0	0	0.057	0.008	5	7	75
KEGG_CARDIAC_MUSCLE_CONTRACTION	0.477	0	0	0.1495	0	0.246	0	0	0	0	0	0.433	0	0	0.002	0.023	4	8	75
KEGG_COMPLEMENT_AND_COAGULATION_CASCADES	0.266	0	0	0.349	0	0.377	0	0.001	0	0	0	0.328	0	0	0	0.019	4	8	75
KEGG_OLFACTORY_TRANSDUCTION	0.999	0	0	0.14	0	0.2455	0	0.001	0	0	0	0.71	0	0	0	0.019	4	8	75
KEGG_TYPE_I_DIABETES_MELLITUS	0.33	0	0	0.142	0	0.5815	0	0.001	0	0	0	0.541	0	0	0	0.045	4	8	75
KEGG_ALDOSTERONE_REGULATED_SODIUM_REABSORPTION	0.313	0	0	0.1915	0	0.301	0	0	0	0	0	0.65	0	0	0.017	0.032	4	8	75
KEGG_PRION_DISEASES	0	0	0	0.051	0.0005	0.4255	0	0	0	0	0	0.374	0	0	0.021	0.096	5	7	75
KEGG_PRIMARY_IMMUNODEFICIENCY	0.276	0	0	0.0395	0	0.4475	0	0.007	0	0	0	0.71	0	0.012	0.006	0.152	5	7	75
KEGG_HYPERTROPHIC_CARDIOMYOPATHY_HCM	0.385	0	0	0.0185	0	0.105	0	0.011	0	0	0	0.341	0	0	0	0.057	5	7	75
KEGG_STARCH_AND_SUCROSE_METABOLISM	0.058	0	0	0.0705	0.001	0.197	0	0.007	0	0	0	0.47	0	0	0.082	0.019	4	7	68.75
KEGG_N_GLYCAN_BIOSYNTHESIS	0.365	0	0	0	0	0.7125	0	0	0	0	0	0.326	0	0	0.051	0.12	5	6	68.75
KEGG_OTHER_GLYCAN_DEGRADATION	0.066	0	0	0.023	0.021	0.894	0	0.008	0	0	0.002	0.668	0	0.013	0.207	0.231	5	6	68.75
KEGG_O_GLYCAN_BIOSYNTHESIS	0.355	0	0	0.062	0.001	0	0	0.091	0	0	0	0.642	0	0.021	0.024	0.164	5	6	68.75
KEGG_GLYCOSAMINOGLYCAN_BIOSYNTHESIS_CHONDROITIN_SULFATE	0.431	0	0	0.0155	0.0175	0.7855	0	0.002	0	0	0	0.615	0	0.008	0.121	0.261	5	6	68.75
KEGG_GLYCOSAMINOGLYCAN_BIOSYNTHESIS_HEPARAN_SULFATE	0.547	0	0	0.0025	0	0.8585	0	0.04	0	0	0	0.616	0	0.012	0.192	0.132	5	6	68.75
KEGG_GLYCOSYLPHOSPHATIDYLINOSITOL_GPI_ANCHOR_BIOSYNTHESIS	0.314	0	0	0.002	0.0055	0.431	0	0.012	0	0	0	0.468	0	0.018	0.147	0.195	5	6	68.75
KEGG_ETHER_LIPID_METABOLISM	0.1	0	0	0.022	0	0.088	0	0.05	0	0	0	0.524	0	0.006	0.066	0.048	5	6	68.75
KEGG_LINOLEIC_ACID_METABOLISM	0.811	0	0	0.246	0	0	0	0.048	0	0	0	0.737	0	0.036	0.108	0.076	5	6	68.75
KEGG_PROPANOATE_METABOLISM	0.067	0	0	0.007	0	0.714	0	0	0	0	0	0.453	0	0.001	0.063	0.05	5	6	68.75
KEGG_ABC_TRANSPORTERS	0.906	0	0	0.106	0	0.289	0	0.002	0	0	0	0.528	0	0	0.002	0.057	4	7	68.75
KEGG_PROTEASOME	0.544	0	0	0.001	0	0.5985	0	0	0	0	0.004	0.313	0	0	0.085	0.085	5	6	68.75
KEGG_PROTEIN_EXPORT	0.215	0	0	0.0315	0	0.356	0	0	0	0	0	0.539	0	0.012	0.12	0.166	5	6	68.75
KEGG_MISMATCH_REPAIR	0.87	0	0	0	0	0.466	0	0	0	0	0	0.587	0	0.003	0.149	0.21	5	6	68.75
KEGG_HOMOLOGOUS_RECOMBINATION	0.799	0	0	0.003	0	0.411	0	0	0	0	0	0.512	0	0	0.069	0.128	5	6	68.75
KEGG_REGULATION_OF_AUTOPHAGY	0.219	0	0	0.006	0	0.551	0	0.039	0	0	0	0.528	0	0.001	0.072	0.153	5	6	68.75
KEGG_DORSO_VENTRAL_AXIS_FORMATION	0.21	0	0	0.0215	0.005	0.0905	0	0.003	0	0	0	0.652	0	0.009	0.053	0.111	5	6	68.75
KEGG_INTESTINAL_IMMUNE_NETWORK_FOR_IGA_PRODUCTION	0.329	0	0	0.114	0	0.2245	0	0.029	0	0	0	0.533	0	0	0	0.102	4	7	68.75
KEGG_TYPE_II_DIABETES_MELLITUS	0.288	0	0	0.0605	0.0005	0.2615	0	0.011	0	0	0	0.598	0	0	0.02	0.079	4	7	68.75
KEGG_AUTOIMMUNE_THYROID_DISEASE	0.456	0	0	0.235	0	0.3675	0	0.015	0	0	0	0.573	0	0	0	0.083	4	7	68.75
KEGG_ALLOGRAFT_REJECTION	0.314	0	0	0.144	0	0.4655	0	0.009	0	0	0	0.588	0	0.001	0	0.099	4	7	68.75
KEGG_GRAFT_VERSUS_HOST_DISEASE	0.064	0	0	0.137	0	0.157	0	0.005	0	0	0	0.61	0	0.001	0	0.101	4	7	68.75
KEGG_GLYCINE_SERINE_AND_THREONINE_METABOLISM	0.686	0	0	0.228	0.0025	0.056	0	0.008	0	0	0	0.405	0	0.001	0.08	0.056	4	6	62.5
KEGG_VALINE_LEUCINE_AND_ISOLEUCINE_BIOSYNTHESIS	0.051	0	0	0.2055	0.0035	0.359	0	0.002	0	0	0	0.592	0	0.004	0.096	0.281	4	6	62.5
KEGG_PHENYLALANINE_METABOLISM	0.098	0	0	0.265	0.0175	0.9105	0	0.026	0	0	0	0.653	0	0.028	0.067	0.109	4	6	62.5
KEGG_BETA_ALANINE_METABOLISM	0.706	0	0	0.109	0	0.845	0	0	0	0	0	0.595	0	0.001	0.081	0.057	4	6	62.5
KEGG_GLYCOSAMINOGLYCAN_DEGRADATION	0.084	0	0	0.0185	0.1075	0.2485	0	0.041	0	0	0	0.609	0	0.002	0.078	0.184	4	6	62.5
KEGG_GLYCOSAMINOGLYCAN_BIOSYNTHESIS_KERATAN_SULFATE	0.311	0	0	0.0575	0.014	0.639	0	0.006	0	0	0	0.586	0	0.013	0.225	0.291	4	6	62.5
KEGG_GLYCOSPHINGOLIPID_BIOSYNTHESIS_LACTO_AND_NEOLACTO_SERIES	0.602	0	0	0.066	0.0045	0.227	0	0.049	0	0	0	0.617	0	0.001	0.117	0.138	4	6	62.5
KEGG_GLYCOSPHINGOLIPID_BIOSYNTHESIS_GLOBO_SERIES	0.487	0	0	0.108	0.0455	0.811	0	0.041	0	0	0	0.685	0	0.04	0.219	0.317	4	6	62.5
KEGG_GLYCOSPHINGOLIPID_BIOSYNTHESIS_GANGLIO_SERIES	0.099	0	0	0.2465	0.0035	0.867	0	0.032	0	0	0	0.625	0	0.02	0.248	0.448	4	6	62.5
KEGG_GLYOXYLATE_AND_DICARBOXYLATE_METABOLISM	0.115	0	0	0.0345	0.0295	0.1965	0.001	0.008	0	0	0.018	0.734	0	0.065	0.343	0.3	5	5	62.5
KEGG_ONE_CARBON_POOL_BY_FOLATE	0.353	0	0	0.1335	0.004	0.4335	0	0	0	0	0.041	0.522	0	0	0.221	0.227	4	6	62.5
KEGG_NICOTINATE_AND_NICOTINAMIDE_METABOLISM	0.355	0	0	0.24	0.0005	0.4805	0	0.04	0	0	0	0.64	0	0.002	0.169	0.128	4	6	62.5
KEGG_PANTOTHENATE_AND_COA_BIOSYNTHESIS	0.059	0	0	0.4485	0.0005	0.202	0	0.03	0	0	0	0.589	0	0.001	0.12	0.175	4	6	62.5
KEGG_TERPENOID_BACKBONE_BIOSYNTHESIS	0.149	0	0	0.116	0	0.166	0	0.01	0	0	0	0.604	0	0.017	0.23	0.142	4	6	62.5
KEGG_NON_HOMOLOGOUS_END_JOINING	0.517	0	0	0.0005	0	0.774	0	0.001	0	0	0	0.69	0	0.064	0.223	0.287	5	5	62.5
KEGG_TASTE_TRANSDUCTION	0.949	0	0	0.1565	0	0.248	0	0.006	0	0	0	0.736	0	0	0.059	0.216	4	6	62.5
KEGG_PROXIMAL_TUBULE_BICARBONATE_RECLAMATION	0.331	0	0	0.158	0	0.385	0	0.001	0	0	0	0.674	0	0.002	0.095	0.052	4	6	62.5
KEGG_ASTHMA	0.672	0	0	0.2195	0	0.354	0	0.827	0	0	0	0.722	0	0.001	0.001	0.147	4	6	62.5
KEGG_PENTOSE_AND_GLUCURONATE_INTERCONVERSIONS	0.342	0	0	0.2215	0.0005	0.6485	0	0.11	0	0	0	0.556	0	0.041	0.167	0.177	4	5	56.25
KEGG_ASCORBATE_AND_ALDARATE_METABOLISM	0.314	0	0	0.3335	0	0.6175	0.0175	0.052	0	0	0	0.719	0	0.021	0.124	0.192	4	5	56.25
KEGG_PRIMARY_BILE_ACID_BIOSYNTHESIS	0.572	0	0	0.0705	0	0.2935	0	0.023	0	0	0	0.623	0	0.07	0.162	0.188	4	5	56.25
KEGG_TAURINE_AND_HYPOTAURINE_METABOLISM	0.977	0	0	0.132	0	0	0	0.087	0	0	0	0.731	0	0.056	0.192	0.273	5	4	56.25
KEGG_FOLATE_BIOSYNTHESIS	0.437	0	0	0.19	0.011	0.7565	0	0.001	0	0	0	0.659	0	0.055	0.358	0.413	4	5	56.25
KEGG_LIMONENE_AND_PINENE_DEGRADATION	0.392	0	0	0.0845	0	0.6905	0	0.001	0	0	0.001	0.672	0	0.061	0.2	0.231	4	5	56.25
KEGG_SULFUR_METABOLISM	0.186	0	0	0.078	0.006	0.307	0	0.056	0	0	0	0.756	0	0.018	0.155	0.164	4	5	56.25
KEGG_RENIN_ANGIOTENSIN_SYSTEM	0.98	0	0	0.5255	0.0685	0.3385	0.0015	0.05	0	0	0	0.89	0	0.01	0.035	0.063	3	6	56.25
KEGG_ALPHA_LINOLENIC_ACID_METABOLISM	0.213	0	0	0.1905	0	0.2215	0.002	0.068	0	0	0	0.634	0	0.061	0.219	0.077	4	4	50
KEGG_CIRCADIAN_RHYTHM_MAMMAL	0.522	0	0	0.0445	0.1025	0.3815	0.0005	0.161	0	0	0.09	0.561	0	0.031	0.155	0.22	4	4	50
KEGG_MATURITY_ONSET_DIABETES_OF_THE_YOUNG	0.934	0	0	0.2415	0	0.761	0	0.087	0	0	0	0.743	0	0.051	0.127	0.381	4	4	50
