# Supplementary material for: Meta-Analysis and Gene Set Analysis of Archived Microarrays Suggest Implication of the Spliceosome in Metastatic and Hypoxic Phenotypes
Source: PLoS One. 2014 Jan 31;9(1):e86699. doi: 10.1371/journal.pone.0086699 (PMC3908947; doi:10.1371/journal.pone.0086699)
Supplement: Table S4 — Distribution of the highlighted genes in the spliceosome pathway. (PDF) [file pone.0086699.s004.pdf]

Distribution of genes in the spliceosome pathway. Each Unit of the spliceosome pathway consists of a core of proteins combined with a snRNA. There are also a large number of related proteins that play a role in the regulation of the splicing process. The genes highlighted in red are the results of our analysis.

**Unit**	**U1**				**U1 related**			
**Subunits**	**Sm**	**U1-70K**	**UI1**	**U1C**	**FNP11**	**S164**	**p68**	**CA150**
	SNRPB	SNRNP70	SNRPA	SNRPC	PRPF40B	RBM25	DDX5	TCERG1
	SNRPD1				PRPF40A			
	SNRPD2							
	SNRPD3							
	SNRPE							
	SNRPF							
	SNRPG							

**Unit**	**U2**					**U2 related**						
**Subunits**	**Sm**	**U2A'**	**U2B''**	**SF3a**	**SF3b**	**U2AF**	**PUF60**	**SPF30**	**SPF45**	**CHERP**	**SR140**	**Prp43**
	SNRPB	SNRPA1	SNRPB2	SF3A1	SF3B1	U2AF1	PUF60	SMNDC1	RBM17	CHERP	U2SURP	DHX15
	SNRPD1			SF3A2	SF3B2	U2AF2						
	SNRPD2			SF3A3	SF3B3							
	SNRPD3				SF3B4							
	SNRPE				SF3B5							
	SNRPF				SF3B14							
	SNRPG				DDX42							
					PHF5A							

**Unit**	**U4/U6**							**U4/U6.U5 ass**				
**Subunits**	**Lsm**	**Sm**	**Prp3**	**Prp4**	**CypH**	**Prp31**	**Snu13**	**SnRNP27**	**Sad1**	**Snu66**	**Snu23**	**Prp38**
	LSM2	SNRPB	PRPF3	PRPF4	PPIH	PRPF31	NHP2L1	SNRNP27	USP39	SART1	ZMAT2	PRPF38A
	LSM3	SNRPD1										PRPF38B
	LSM4	SNRPD2										
	LSM5	SNRPD3										
	LSM6	SNRPE										
	LSM7	SNRPF										
	NAA38	SNRPG										

**Unit**	**U5**							
**Subunits**	**Sm**	**Snu114**	**Brr2**	**Prp6**	**Prp8**	**Prp8BP**	**Prp28**	**DIB1**
	SNRPB	EFTUD2	SNRNP200	PRPF6	PRPF8	SNRNP40	DDX23	TXNL4A
	SNRPD1							
	SNRPD2							
	SNRPD3							
	SNRPE							
	SNRPF							
	SNRPG							

**Unit**	**Prp19 complex**								
**Subunits**	**Prp19**	**CDC5**	**SPF27**	**PRL1**	**AD002**	**CINNEL1**	**HSP73**	**NPW38**	**NPW38BP**
	PRPF19	CDC5L	BCAS2	PRLG1	CWC15	CTNNBL1	HSPA1A	PQBP1	WBP11
							HSPA1B		
							HSPA1L		
							HSPA2		
							HSPA6		
							HSPA8		

**Unit**	**Prp19 related**								
**Subunits**	**SKIP**	**Syf**	**Isy1**	**PPIL1**	**CypE**	**CCDC12**	**RBM22**	**G10**	**AQR**
	SNW1	SYF2	ISY1	PPIL1	PPIE	CCDC12	RBM22	BUD31	AQR
		CRNKL1							
		XAB2							

**Unit**	**EJC/TREX**					
**Subunits**	**ACINUS**	**eIFA3**	**Y14**	**magoh**	**UAP56**	**THOC**
	ACIN1	EIF4A3	RBM8A	MAGOH	DDX39B	THOC1
				MAGOHB		THOC2
						THOC3
						THOC4

**Unit**	**Common components**			**N/A**								
**Subunits**	**CBP80/20**	**hnRNPs**	**SR**	**Prp5**	**UAP56**	**Prp2**	**Prp16**	**Prp17**	**Prp18**	**Prp22**	**Slu7**	**Prp43**
	NCBP1	HNRNPA1L2	TRA2A	DDX46	DDX39B	DHX16	DHX38	CDC40	PRPF18	DHX8	SLU7	DHX15
	NCBP2	HNRNPA3	TRA2B									
		RBMX	SRSF1									
		HNRNPA1	SRSF2									
		HNRNPC	SRSF3									
		HNRNPK	SRSF4									
		HNRNPU	SRSF5									
		HNRNPM	SRSF6									
		RBMXL1	SRSF7									
		PCBP1	SRSF8									
			SRSF9									
			SRSF10									
