# Supplementary material for: Meta-Analysis and Gene Set Analysis of Archived Microarrays Suggest Implication of the Spliceosome in Metastatic and Hypoxic Phenotypes
Source: PLoS One. 2014 Jan 31;9(1):e86699. doi: 10.1371/journal.pone.0086699 (PMC3908947; doi:10.1371/journal.pone.0086699)
Supplement: Table S5 — R code for the full analysis. (PDF) [file pone.0086699.s005.pdf]

R code for the analysis presented in this manuscript.

```
# In this analysis, we first perform separated pretreatment and treatment,
# then combine results in the meta-analysis process.
## 1. Pretreatment in R2.7 for a HG-U133a dataset ##

library(affy)
library(gcrma)
library(hgu133acdf)
# The alternative cdf file needs dimensions from the classic cdf
library(hgu133atranscriptccds)
data(hgu133atranscriptccds)
hgu133atranscriptccdsdim<-hgu133adim
# Dimensions are taken into account here
cel<-list.celfiles()
a<-ReadAffy(filenames=cel)
b<-gcrma(a, cdfname="hgu133atranscriptccds")
c<-exprs(b)
# GCRMA classic pretreatment with alternative cdf used
save(b, file="b.Rdata", compress=T)

## 2. Treatment in R 2.11 ##
# This step has to be done separately for each dataset
library(st)
load("c.Rdata")

# Here, ncontr and ntest represent the number of control and tests
replicates
C<-ncontr
T<-ntest
labels<-c(rep(1, C), rep(2, T))
# Shrinkage t needs this ‘labels’ vector for statuses of samples
# (control or test)
d<-t(c)
# Shrinkage t also needs data to be transposed, rows are now samples
# and columns are probe sets
st.result<-shrinkt.stat(X=d, L=labels, var.equal=T)
pvalues<-(1-pt(abs(st.result), df=(C+T)))*2
pvalues[is.na(pvalues)]<-1
st.pvals<-cbind(pvalues)
# Pvalues are obtained
save(st.pvals, file="st.pvals.Rdata", compress=T)

## 3. Drawing of volcano plot ##
# Volcano plots compare pvalues (st.pvals) to fold changes
meanC<-c()
meanT<-c()
MeanC<-rowMeans(c[,1:C])
MeanT<-rowMeans(c[, T:C(+T)])
fc<-MeanC-MeanT
# Computation of fold changes. Since expression values are in log2 (gcrma),
# the fold change is meanControls - meanTests
png(filename="Volcano_plot.png")
plot(fc, -log10(st.pvals), pch=".", main="Volcanoplot", xlab="Fold Change",
ylab="-log10 pvalues")
abline(h=-log10(0.01),col="blue")
abline(v= 2, col="green")
abline(v=-2, col="green")
# Lines are added to provide visual marks on the graph
dev.off()
# The individual analyses are done
```

```
## 4. Computing of counts through all experiments ##
# First create an object containing all pvalues computed for all
experiements (within a technology) called 'pvals.gen'
count<-rep(0, times=15278)
# The ‘count’ vector will contain all occurrences of having a pvalue under
# 5% for each probe set. Its lengtht depends on the technology: HG-U133a
#(with alternative cdf)= 15278; HG-U133plus2 (with alternative cdf): 23798
test<-c()
temp<-c()
for (i in 1:nrow(pvals.gen))
# For every row of the pvals.gen object
	{
	test<-c()
	test<-pvals.gen[i,]
	# Selection of a single row
	temp<-c()
	for (j in 1:length(test))
	# For every column in the singled out row
		{
		if(test[j]<0.05)
		# If pvalue is under 5%
			{
			temp<-temp+1
			# Add 1 to the count
			}
		count[i]<-temp
		# In the ‘temp’ object are the counts for each dataset row
		}
	}

# Select the probe sets with a count superior to 1 (or other value)
DEG2<-which(count>=1)
# One first selection can be performed here to select transcript within
# same technology datasets

# Then if 'ls' contains the names of the folder of the individual analyses
for (i in 1:length(ls))
	{
	setwd(ls[i])
	load("c.Rdata")
	temp<-c()
	temp<-c[DEG2,]
	sub2<-cbind(sub2,temp)
	setwd("../")
	}
# This step selects data rows corresponding to probe sets selected at the
# previous step

# To compare results from different technologies, translation to ENTREZID
of probesetID
EntrezID<-c()
for (i in 1:nrow(sub2))
	{
	test<-rownames(sub2)[i]
	temp<-
hgu133plus2transcriptccdsannotinfo[hgu133plus2transcriptccdsannotinfo$PROBE
==test,]$ENTREZGENEID
	EntrezID[i]<-temp[1]
	}
# At the end of this step, we have an ‘EntrezID’ object, containing the
```

```
# identifiers of probe sets/transcripts selected above. This is done for
# each of the technologies (HG-U133a and HG-U133plus2), resulting in two
# lists (a and b) of probe sets/transcripts.

# Then to compute the intersection between technologies. a = HG-U133A,
b=HG-U133plus2
library(stats)
ab<-interect(a, b)
# Simple intersection between the now comparable results for the two
# technologies. This step selects probe sets/transcripts at least once DEG
# in each technology. The same can be done with biological groups, one only
# needs to adapt the definition of the a and b groups.



## 5. FAERI gene set computations ##
# Creation of the gene set list based on MSigDB
# To do in R 2.7 to use alternative cdfs

load("msigDB3.0.Rdata")
# The data can be obtained on the MSigDB website
# « http://www.broadinstitute.org/gsea/msigdb/index.jsp »
gs.list<-c()
gs.list$description<-c()
gs.list$genesets<-c(c2.kegg[[2]])
# In this study we only used the KEGG.pathways definitions contained
# within MSigDB
gs.list$description<c(gs.list$description,rep("C2.KEGG",length(c2.kegg[[2]]
)))

gslist2pslist<-function(genes,corr.vect)
	{
	pslist<-sapply(genes,gs2ps,corr.vect)
	pslist<-unlist(pslist)
	return(pslist)
	}

gs2ps<-function(gene,corr.vect)
	{
	index<-which(corr.vect==gene);
	ps<-names(corr.vect)[index];
	return(ps);
	}

bin2list<-function(gs,bin.table)
	{
	ps.index<-which(bin.table[,gs]==1)
	ps.names<-rownames(bin.table)[ps.index]
	return(ps.names)
	}
# This serie of functions allows to create gene set definitions based on
# MSigDB (in this case), which contains actual probe set names

# Conversion of genelists into probesetlists

ps.list.full<-lapply(gs.list$genesets,gslist2pslist,dataset$symbol)
save(ps.list.full,file="ps.list.full.RData",compress=T)
# Here these functions are summoned

# In R 2.12
```

```
library(pegase)
# This step must be done with a pegase library V0.9 or higher

for (i in 1:length(ls))
	{
	setwd(ls[i])
	result.full<-c()
	pvals.gs.full<-c()
	load("dataset.Rdata")
	# The dataset must be prepared beforehand. An object under
	# (dataset$design) contains the columns statuses (control or tests #
	i.e. 1 or 2), and the data is under (dataset$exprs)
	result.full<-
pegase(data=NULL,A=dataset$exprs[,which(dataset$design==1)],B=dataset$exprs
[,which(dataset$design==2)],steps=c("prepare","run","run.gs"),methods=c("st
udent","welch"),methods.gs=c("a2.fixed",
"faeri.fixed.perms"),gs.reduce=2,genesets=ps.list.full,genenames=rownames(d
ataset$exprs),faeri.nelem=5000,faeri.ngroups=1000)
# This step is the core computation step. Pvalues are computed for
# faeri.fixed.perms and a level-2 fixed ANOVA. The parameters ferai.nelem
# and faeri.ngroups are related to the number of permutations to perform
# for the null hypothesis distribution construction.
	save(result.full, file="result.full.Rdata", compress=T)
	pvals.gs.full<-result.full$pvals.gs
	save(pvals.gs.full, file="pvals.gs.full.Rdata", compress=T)
	write.table(pvals.gs.full, file="pvals.gs.full.txt", sep="\t")
	setwd("../")
	print (ls[i])
	}
```
